# A molecular hypothesis to explain direct and inverse co-morbidities between Alzheimer’s Disease, Glioblastoma and Lung cancer

**DOI:** 10.1038/s41598-017-04400-6

**Published:** 2017-06-30

**Authors:** Jon Sánchez-Valle, Héctor Tejero, Kristina Ibáñez, José Luis Portero, Martin Krallinger, Fátima Al-Shahrour, Rafael Tabarés-Seisdedos, Anaïs Baudot, Alfonso Valencia

**Affiliations:** 1 Structural Biology and Biocomputing Programme, Spanish National Cancer Research Centre (CNIO), Madrid, 28029 Spain; 2 Clinical Research Programme, Spanish National Cancer Research Centre (CNIO), Madrid, 28029 Spain; 3 Bioinformatics section, Institute of Medical and Molecular Genetics (INGEMM), Hospital Universitario La Paz, Madrid, 28046 Spain; 4 Department of Medicine, Hospital HM Sanchinarro, Madrid, 28050 Spain; 5 Department of Medicine, University of Valencia, CIBERSAM, INCLIVA, Valencia, 46010 Spain; 6 Aix-Marseille Université, CNRS, Centrale Marseille, I2M UMR7373 Marseille, France; 7 Barcelona Supercomputing Center (BSC), Barcelona, 08034 Spain; 8 ICREA, Barcelona, 08010 Spain

## Abstract

Epidemiological studies indicate that patients suffering from Alzheimer’s disease have a lower risk of developing lung cancer, and suggest a higher risk of developing glioblastoma. Here we explore the molecular scenarios that might underlie direct and inverse co-morbidities between these diseases. Transcriptomic meta-analyses reveal significant numbers of genes with inverse patterns of expression in Alzheimer’s disease and lung cancer, and with similar patterns of expression in Alzheimer’s disease and glioblastoma. These observations support the existence of molecular substrates that could at least partially account for these direct and inverse co-morbidity relationships. A functional analysis of the sets of deregulated genes points to the immune system, up-regulated in both Alzheimer’s disease and glioblastoma, as a potential link between these two diseases. Mitochondrial metabolism is regulated oppositely in Alzheimer’s disease and lung cancer, indicating that it may be involved in the inverse co-morbidity between these diseases. Finally, oxidative phosphorylation is a good candidate to play a dual role by decreasing or increasing the risk of lung cancer and glioblastoma in Alzheimer’s disease.

## Introduction

Alzheimer’s disease (AD) is a leading global healthcare burden^1^ and, while over one hundred drugs have been developed to treat this disease, only a dozen have been approved for AD treatment in the past 20 years. Unfortunately, none of these halt the disease’s progression^2^. Lung cancer (LC) is the leading cause of cancer-related mortality, with nearly 1.4 million deaths every year^3^. Malignant glioblastomas (GBM) are the most common primary brain tumors in adults and, despite recent therapeutic advances, the life expectancy of patients with GBM continues to be less than 2 years^4^. Thus, these three diseases are considered among the most challenging public health conditions worldwide, emphasizing the need for innovative approaches to deal with them.

Insight into the connections between diseases offer new opportunities to better understand their pathogeneses^5, 6^. Direct co-morbidities are common for many diseases, representing a higher-than-expected joint occurrence of medical conditions in individuals. For example, a direct co-morbidity between AD and brain tumors is currently suspected^7–9^. By contrast, inverse co-morbidities are defined as a lower-than-expected probability of a specific disease in individuals diagnosed with another medical condition. For instance, AD is associated with a lower risk of various cancers, including LC^9, 10^.

Various factors have been proposed to be involved in direct and inverse co-morbidities, such as the environment, lifestyle or drug treatments^11^, and we hypothesize that genetic and molecular factors could also play a role in these relationships. We recently studied a set of Central Nervous System (CNS) disorders and cancers known to display patterns of inverse co-morbidity. Thanks to transcriptomic meta-analyses, we were able to identify a molecular signature of deregulated genes in opposite directions in these diseases^12^. Here we aim to challenge the molecular bases of inverse and of direct co-morbidities, and the role of the affected tissues in these associations. As such, we conducted a systematic meta-analysis of transcriptomic gene expression data in AD, GBM and LC, comparing the deregulated genes in each disease to each other. Brain and lung control samples were used to detect basal tissue-associated gene expression in order to rule out confounding data. Accordingly, we identified a significant number of genes that were deregulated in opposite directions in AD and LC, inverse expression that was associated to the proteasome, protein folding and mitochondrial processes. We propose that such deregulation could represent a molecular substrate for the inverse co-morbidity observed between these 2 diseases. By contrast, we found a significant number of genes that were deregulated in the same direction in AD and GBM. This deregulation affected the immune system and the potential establishment of a chronic inflammatory state, suggesting that these changes are associated with direct co-morbidity between AD and GBM.

## Results

### Gene expression variations associated to the tissues of origin

To take into account any variations in gene expression associated to the diseased tissues when comparing AD and GBM with LC, we first compared control brain and lung samples (see Methods). A Principal Component Analysis revealed distinct basal expression of genes in the control brain and lung samples, with 66% of the variability explained by the origin of the tissue (Supplementary Fig. S1). An enrichment analysis showed that genes up-regulated in the control lung samples relative to the brain tissue are significantly enriched in immune system-related processes (e.g., “defense response”, “cytokine-cytokine receptor interaction”, “immune response”, “inflammatory response”: Supplementary Table S1). Conversely, the genes up-regulated in the control brains relative to the lung tissue are enriched in brain-related processes, such as “synaptic transmission”, “neurotransmitter release”, “neuron differentiation” and “glucose metabolism” (Supplementary Table S2). All the processes differentially expressed in lung and brain tissues will be further used in our analyses in order to distinguish tissue-associated variations from true changes in gene expression associated to the disease.

### Molecular relationships between Alzheimer’s disease and lung cancer

**Figure 1:**
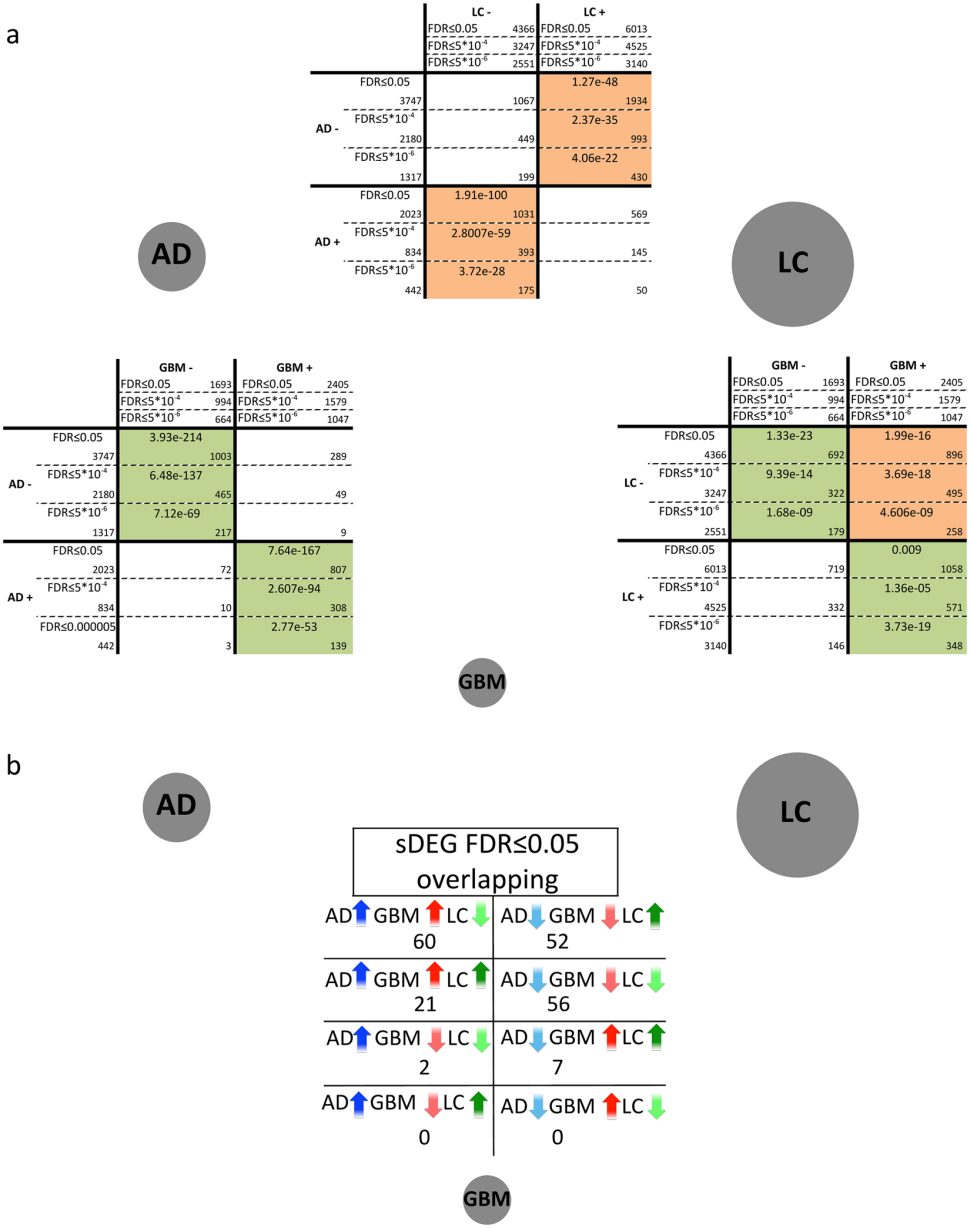
Overlaps between significantly differentially expressed genes (sDEGs) in Alzheimer’s disease (AD), lung cancer (LC) and glioblastoma (GBM). The grey circles represent each of the diseases studied and their size is proportional to the total number of sDEGs identified for each disease with a FDR ≤ 0.05. (**a**) Pairwise comparisons of sDEGs identified as significantly up- and down-regulated with 3 different FDR cut-offs (FDR ≤ 0.05, 5 × 10^−4^ & 5 × 10^−6^) after gene expression meta-analyses for AD, LC and GBM. Orange and green cells indicate significant overlaps between the genes significantly differentially expressed (sDEGs) in the same and opposite direction, respectively (Fisher’s exact test, FDR ≤ 0.05). White cells correspond to non-significant overlaps (FDR > 0.05). The number of sDEGs in each disease and in the pairwise overlaps are indicated in their corresponding cell. (**b**) Numbers of overlapping sDEGs identified jointly in the 3 diseases.

We conducted transcriptomic meta-analyses and identified “significantly Differentially Expressed Genes” (sDEGs) in AD, GBM and LC (see Methods, Supplementary Table S3). We first focused on the molecular relationships between AD and LC, two diseases that display inverse co-morbidity according to epidemiological data^9, 10^. As such, we compared the up- and down-regulated sDEGs in these 2 diseases, finding significant overlaps (FDR ≤ 0.05) between sDEGs deregulated in opposite directions in AD and LC (i.e., AD+/LC− or AD−/LC+: Fig. 1a), confirming previous observations^12^. These results persisted when more stringent FDR cut-offs were established for the detection of sDEGs (FDR ≤ 0.05, 5 × 10^−4^ and 5 × 10^−6^: Fig. 1a), as well as with an alternative meta-analysis approach (Methods and Supplementary Fig. S2).

**Figure 2:**
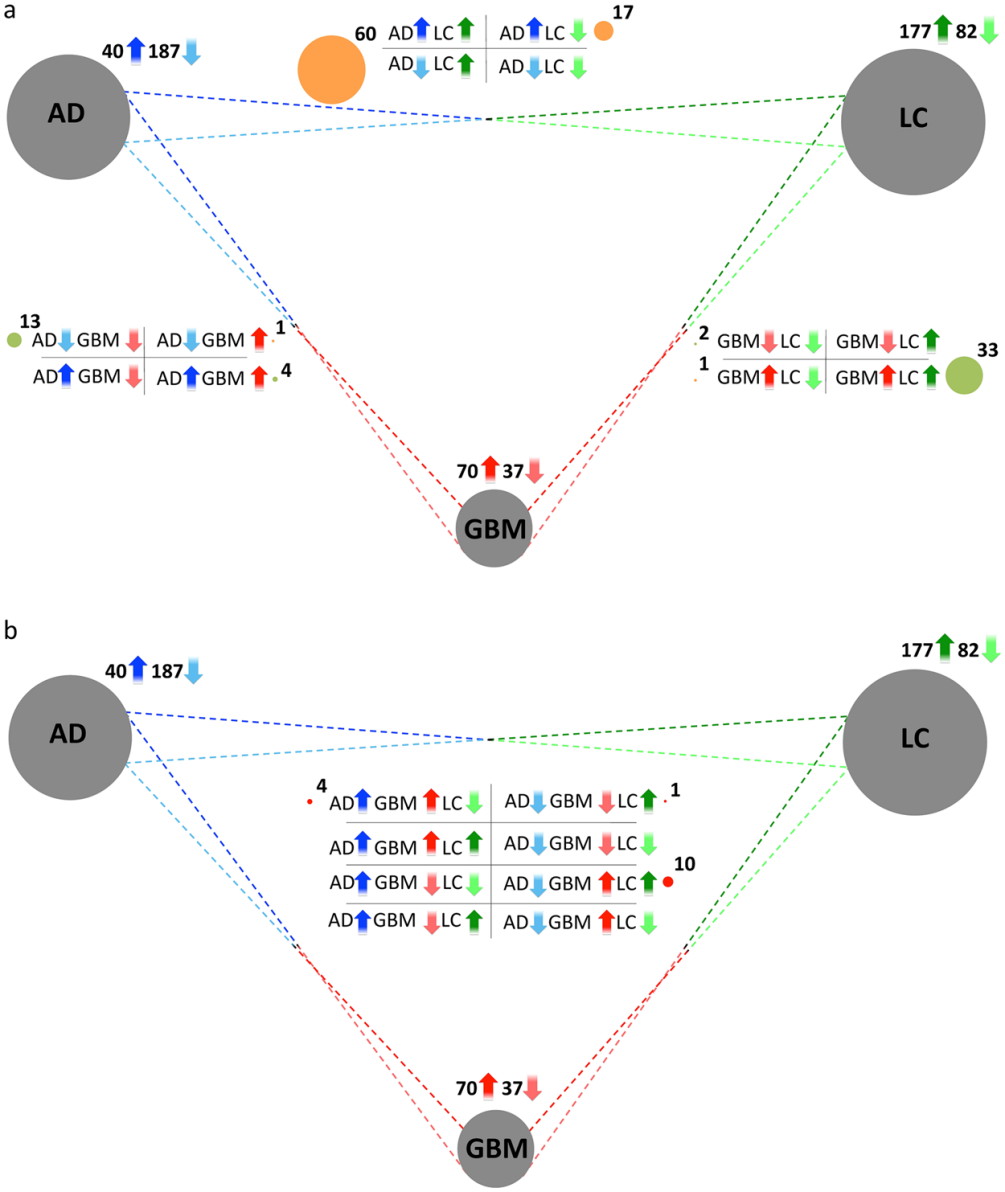
Overlaps between processes and pathways significantly enriched in Alzheimer’s disease (AD), lung cancer (LC) and glioblastoma (GBM). Grey circles represent each of the diseases studied, and their size is proportional to the total number of processes/pathways up- or down-regulated in each case. (**a**) Number of Biological Processes, and KEGG and Reactome pathways significantly up- and down-regulated (FDR ≤ 0.05) in at least two of the three diseases. Dark and light arrows represent pathways up- and down-regulated, respectively. The Processes/Pathways deregulated in two diseases are located in between their corresponding grey circles, and correspond to the 4 possible combinations: up or down in both diseases, up in one disease and down in the other, and vice versa. Green circles denote the number of pathways deregulated in the same direction in two diseases, while the orange circles denote numbers of pathways deregulated in opposite directions in two diseases. (**b**) The Pathways/Processes significantly enriched in all 3 diseases.

We conducted gene set enrichment analyses independently for each disease^13^, and we found 395 biological processes and pathways significantly deregulated in either AD and/or LC (Supplementary Table S4). Of these, 92 were common to both diseases, including 21 processes up-regulated in AD and down-regulated in LC (AD+/LC−), and 71 processes down-regulated in AD and up-regulated in LC (AD−/LC+: Fig. 2, Table 1). These common processes annotate a total of 31% (315/1031) of the AD+/LC− sDEGs and 29% (553/1934) of the AD−/LC+ sDEGs (Supplementary Table S5). No processes were deregulated in the same direction in AD and LC at the threshold selected (FDR ≤ 0.05).

**Table 1:** Overlap between the processes and pathways significantly enriched in Alzheimer’s disease (AD), glioblastoma (GBM) and lung cancer (LC). Biological Processes, KEGG and Reactome pathways significantly up- and down-regulated (FDR £ 0.05) in pairwise and three-way comparisons are identified through the GSEA approach. The Pathways/Processes also significantly down-regulated in brain vs. lung control samples, and those which then correspond to tissue-related expression variation are indicated with asterisks, while those significantly up-regulated in brain vs. lung control samples are indicated by circles.

AD+/GBM+	AD−/LC+	GBM+/LC+
ECM receptor interaction	Alanine aspartate and glutamate metabolism°	Activation of the pre replicative complex
Interferon alpha beta signaling	Aminoacyl tRNA biosynthesis	Base excision repair
Response to virus	APC C CDH1 mediated degradation of CDC20 and other APC C CDH1 targeted proteins in late mitosis early G1	Cell cycle phase
RIG I MDA5 mediated induction of IFN alpha beta pathways		Cell cycle
	APC CDC20 mediated degradation of NEK2A	Deposition of new CENPA containing nucleosomes at the centromere
	Asparagine N linked glycosylation	
	Assembly of the pre replicative complex	DNA integrity checkpoint
**AD−/GBM−**	Autodegradation of CDH1 by CDH1 APC C	DNA metabolic process
Cardiac muscle contraction	Autodegradation of the E3 ubiquitin ligase COP1	DNA repair
DARPP 32 events	Biosynthesis of the n glycan precursor dolichol lipid linked oligosaccharide LLO and transfer to a nascent protein*	DNA replication
G alpha z signaling events		DNA strand elongation
GABA synthesis release reuptake and degradation	CDK mediated phosphorylation and removal of CDC6	Double strand break repair
Generation of neurons	Citrate cycle TCA cycle	Extracellular matrix organization*
Neuron differentiation	Cross presentation of soluble exogenous antigens endosomes	G1 S specific transcription
Neuronal system	Cyclin e associated events during G1 S transition	G2 M checkpoints
Neurotransmitter receptor binding and downstream transmission in the post-synaptic cell	Cytosolic tRNA aminoacylation	Global genomic NER GG NER
	Destabilization of mRNA by auf1 HNRNP D0	Homologous recombination repair of replication independent double strand breaks
Neurotransmitter release cycle	Double strand break repair	
Potassium channels	ER phagosome pathway	Homologous recombination
Synaptic transmission	Formation of RNA pol II elongation complex	M phase of mitotic cell cycle
Transmission across chemical synapses	Formation of the HIV1 early elongation complex	M phase
Transmission of nerve impulse	Formation of transcription coupled NER TC NER repair complex	Meiosis I
	Gluconeogenesis°	Meiosis
	Glutathione metabolism	Metabolism of nucleotides
**AD−/GBM+**	HIV infection	Mitosis
Nucleotide excision repair	HIV life cycle	Mitotic cell cycle checkpoint
	Interactions of VPR with host cellular proteins	Mitotic cell cycle
	Late phase of HIV life cycle	NEP NS2 interacts with the cellular export machinery
**AD+/LC−**	M G1 transition	p53 signaling pathway
Allograft rejection*	Metabolism of amino acids and derivatives	Regulation of cell cycle
Cell adhesion molecules CAMS	Metabolism of non coding RNA	Regulation of glucokinase by glucokinase regulatory protein
Cellular defense response*	Metabolism of proteins	Regulation of mitosis
Chemokine receptors bind chemokines*	MHC class II antigen presentation	Response to DNA damage stimulus
Complement and coagulation cascades*	Mitochondrial protein import	Response to endogenous stimulus
Complement cascade*	Mitochondrion organization and biogenesis	Transport of ribonucleoproteins into the host nucleus
Cytokine cytokine receptor interaction*	mRNA capping	
Defense response*	mRNA processing	
Graft versus host disease*	mRNA splicing minor pathway	**GBM−/LC−**
Hematopoietic cell lineage*	N glycan biosynthesis	Effects of PIP2 hydrolysis
Immune response*	Nucleobasenucleoside and nucleotide metabolic process	Phospholipase C mediated cascade°
Immune system process*	Nucleotide excision repair	
Immunoregulatory interactions between a lymphoid and a non lymphoid cell*	p53 dependent G1 DNA damage response	
	p53 independent G1 S DNA damage checkpoint	GBM+/LC−
Inflammatory response*	Processing of capped intron containing pre mRNA	Response to wounding*
Interferon gamma signaling*	Proteasome	
Leishmania infection*	Protein folding	
NOD like receptor signaling pathway*	Pyruvate metabolism and citric acid TCA cycle	AD−/GBM+/LC+
	Recruitment of mitotic centrosome proteins and complexes	Activation of ATR in response to replication stress
	Respiratory electron transport ATP synthesis by chemiosmotic coupling and heat production by uncoupling proteins	Cell cycle checkpoints
**AD+/GBM+/LC−**		Cell cycle mitotic
Cell surface interactions at the vascular wall*	Respiratory electron transport	DNA replication
Innate immune system*	RNA pol II pre transcription events	G1 S transition
Response to other organism*	RNA pol II transcription pre initiation and promoter opening	Mitotic G1 G1 S phases
Viral myocarditis*	RNA pol II transcription	Mitotic M M G1 phases
	RNA polymerase	S phase
	RNA processing	Synthesis of DNA
**AD−/GBM−/LC+**	SCF beta TRCP mediated degradation of EMI1	DNA repair
Oxidative phosphorylation	SCFSKP2 mediated degradation of p27 p21	
	TCA cycle and respiratory electron transport	
	Transcription coupled NER TC NER	
	tRNA aminoacylation	
	VIF mediated degradation of APOBEC3G	

The processes in which the genes up-regulated in AD and down-regulated in LC are enriched include immune and inflammatory responses (Table 1). These functions are also expressed differentially between control brain and lung samples, and the enrichment is probably due to the tissue of origin of the diseases (Supplementary Table S1). This was confirmed by extracting information regarding tissue-specific gene expression from GTEx^14^ and by conducting a gene enrichment analysis^15^ on AD+/LC− lung-specific genes (Supplementary Table S6). Similarly, the processes down-regulated in AD and up-regulated in LC included processes related to synaptic transmission (Table 1), that were also expressed differentially between control brain and lung samples (Supplementary Table S2). As described previously, these results were confirmed with an enrichment analysis on AD−/LC+ brain-specific genes (Supplementary Table S6).

Nevertheless, other processes associated with either AD−/LC+ or AD+/LC− are unlikely to be related with the tissue of origin. These include processes related to mitochondrial activity, such as “proteasome”, “protein folding”, “glutathione metabolism”, “TCA cycle and respiratory electron transport” and “oxidative phosphorylation” (Table 1). The deregulation of these processes in opposite directions in AD and LC could be implicated in the inverse co-morbidity observed between these diseases at the epidemiological level.

Given the enrichment observed in mitochondrial-related processes, we assessed whether the gene expression signatures associated to both diseases might be related to inhibition of the electron transport chain (ETC). With this in mind, we collected a set of inhibitors of the respiratory chain from the LINCS library (http://www.lincscloud.org), and we used a drug-set enrichment approach to compare the expression signature induced by these drugs to the expression signature of both AD and LC. This approach reveals that the changes in gene expression associated to inhibitors of the respiratory chain are similar to the changes in gene expression associated with AD in patients, but they are not significantly different to those observed in LC (FDR ≤ 0.05).

Overall, using different datasets and meta-analysis approaches these results confirm our previous results from a comparison of CNS disorders (AD, Parkinson’s disease and schizophrenia) and three types of cancer (LC, prostate and colorectal)^12^.

### Molecular relationships between Alzheimer’s disease and glioblastoma

We applied the same methodology to analyze the molecular relationships between AD and GBM. The epidemiological data concerning the association between these diseases point to a direct co-morbidity relationship^7–9^. This tendency to a higher risk of developing brain tumors in AD patients is also described in HuDiNe, reporting a relative risk value of 2.946^16^. We conducted 6 independent meta-analyses using control samples from 5 different regions of post-mortem brains, and one set of epileptic pre-mortem brains (Methods, Supplementary Fig. S3). We found that sDEGs in GBM varied depending on the brain samples used as controls, as described elsewhere^17–19^, and in the 6 meta-analyses, only 35% and 26% of the sDEGs were consistently up- and down-regulated, respectively (Supplementary Fig. S4). We considered the genes that were consistently deregulated in all 6 GBM meta-analyses as GBM sDEGs (see the sDEGs and their corresponding FDR in Supplementary Table S3).

By contrast to the AD−LC molecular relationships, we found significant overlaps between the sDEGs deregulated in the same direction in AD and GBM (AD+/GBM+ and AD−/GBM−: Fig. 1a). The results were also consistent for the selection of sDEGs with more stringent FDR cut-offs (FDR ≤ 0.05, 5 × 10^−4^ and 5 × 10^−6^), and with an alternative meta-analysis approach (Supplementary Fig. S2). Since the incidence of seizures in GBM patients is between 25–50% at onset and 20–30% during the course of the disease^20^, we verified the significance of the overlaps between AD and GBM using control healthy and epilepsy samples separately for GBM. Irrespective of the samples used as controls (healthy or epilepsy), we obtained significant overlaps between the sDEGs in the same direction in AD and GBM (Supplementary Table S7).

Functional enrichment analyses revealed 301 processes significantly enriched in either AD or GBM, of which 8 are AD+/GBM+, 14 AD−/GBM− and 11 AD−/GBM+. We found that AD+/GBM+ processes were related to the immune system (Table 1). As AD and GBM develop in the same organ, these processes could potentially be molecular substrates of a direct co-morbidity between both diseases at an epidemiological level. Additionally, 10 AD−/GBM− processes denote potentially common neuronal features, such as “synaptic transmission” and “generation of neurons”. Finally, 10 of the 11 processes deregulated in opposite directions (AD−/GBM+) were also associated to LC, as will be discussed in the global comparison of the 3 diseases.

### Molecular relationships between glioblastoma and lung cancer

Finally, we studied the molecular relationships between GBM and LC. To our knowledge, no epidemiological data are available to determine the co-morbidity between these two cancers, although lung cancers often produce brain metastases^21, 22^. We discovered significant overlaps between the sDEGs deregulated in the same direction in both diseases (GBM−/LC− and GBM+/LC+), as well as in those up-regulated in GBM and down-regulated in LC (GBM+/LC−: Fig. 1a).

Among the 70 processes up-regulated in GBM and the 177 processes up-regulated in LC, 43 are up-regulated in both conditions (GBM+/LC+: Table 1 and Fig. 2). Most of these functions are related to the cell cycle, for instance “mitosis” or “DNA repair”. Conversely, only two of the 37 and 82 processes down-regulated in GBM and LC, respectively, are down-regulated in both diseases (GBM−/LC−). As expected from the comparisons between control lung and brain samples, the 5 processes deregulated in opposite directions between the two cancer types (GBM+/LC−) are related to the immune system, and they correspond to variations in tissue-related gene expression (Table 1 and Supplementary Table S1).

### Global comparison of the three diseases

We finally searched for sDEGs and processes that are deregulated jointly in the three diseases, and that therefore might potentially play a dual role in protecting against LC development and promote GBM development in AD patients. With the more restrictive lists of sDEGs (FDR ≤ 5 × 10^−6^: Fig. 1b), 112 of the 198 genes significantly deregulated in the three diseases had a similar pattern of deregulation in AD and GBM, and the opposite pattern in LC, with 60 AD+/GBM+/LC− sDEGs and 52 AD−/GBM−/LC+ sDEGs (Table 2 and Fig. 1b). Similar results were obtained with the lists of sDEGs with a FDR ≤ 0.05 (Supplementary Fig. S5). A comparison of the pathways and processes enriched in AD, GBM and/or LC revealed “oxidative phosphorylation” to be down-regulated in AD and GBM, and up-regulated in LC, pointing to potential metabolic differences between these two types of tumor (Table 1). Additionally, 10 of the 11 processes down-regulated in AD and up-regulated in GBM were also up-regulated in LC. Among these processes, the large majority were related to the cell cycle, a central activity in tumorigenesis (Table 1).

**Table 2:** Significantly differentially expressed genes (FDR ≤ 5 × 10^−6^) as: AD+/GBM+/LC−, AD−/GBM−/LC+, AD+/GBM−/LC−, AD−/GBM+/LC+, AD−/GBM−/LC− and AD+/GBM+/LC+.

AD+/GBM+/LC−		AD−/GBM−/LC+		AD−/GBM−/LC−		AD+/GBM+/LC+
NFKBIA	EMP3	PFN2	KCNK1	B3GNT1	CX3CL1	SPP1
CEBPD	EMP1	ENO2	NMNAT2	OLFM1	NTRK3	DDIT4
S100A10	TRIP10	STMN2	DOCK3	RTN3	RND1	PLOD3
CDKN1A	TNFAIP3	UCHL1	GABBR2	TCEAL2	PLK2	UNG
CSDA	GIMAP4	SCG5	LRRC20	MOAP1	PPL	PPP1R14BP3
PXDC1	MYO1F	WASF1	SEMA4F	TSPAN7	HLF	GTF2IRD1
BCL6	CD163	INA	UNC13A	RTN1	TMEM246	TIMP1
ID3	IL10RA	TUBA4A	PORCN	PEBP1	PRKCB	CHI3L1
FCGRT	S100A4	PGBD5	DLG3	PRKCZ	CD200	INHBB
HLA-DMB	DOCK6	SULT4A1	NELL1	MAST3	RP11-287D1.3	ABCB7
ANXA2P2	CTSS	SCAMP5	FBXO41	GPRASP1	BCAS3	RHBDF2
TSPO	SOCS3	RAB15	RFPL1-AS1	NDRG4	PTK2B	CDKN2C
MYD88	CCR1	HPRT1	HOOK1	NDN	REPS2	PLP2
VSIG4	TRAF3IP2	NDUFS2	CGREF1	NCALD	ELMO1	SLC35F2
VEZF1	HERC5	KIF3C	ACTL6B	SNRPN	PEG3	BACE2
PIEZO1	TCF7L1	EEF1A2	RP11-18A3.4	NRN1	PIP5K1B	FAM60A
ARHGDIB	ZNF516	CKMT1B	TTC9	REEP1	SHANK2	PPIC
CFLAR	LCAT	FNDC4	CNTNAP2	BEX4	SMARCA2	ZNF217
MSX1	TGFB1I1	CDK5	CABYR	LDB2	C2CD2L	TP53
LAMB2	FAM129A	STX1A	RAB3B	MICU1	EPB41L3	CASP6
SRGN	PLBD1	LAMP5	RGS7	CCK	TMEM35	KCNE4
TRIM22	GBP1P1	SEZ6L2	RP11-430B1.2	NAP1L2	KCNAB1	**AD−/GBM+/LC+**
DDR2	SP100	GOT1	SYNDIG1	SNCA	CLCN4	SNRPEP4
C3AR1	ANG	CDH18	TMEM59L	GABARAPL1	CHRM3	SEC61G
RAB20	ECM2	OGDHL	ATP13A2	MEF2C	HAS1	AIMP2
ITGA5	CD58	SH3BP1	SPINT2	APBB1	HSPB3	FTSJ2
STAB1	FLI1	**AD+/GBM−/LC−**		RUNX1T1	CRY2	METTL1
ARHGEF40	C5AR1	RAPGEF3		CYFIP2	TYRP1	RP11-77H9.2
SLC7A7	LYZ	SLC6A12				WDHD1
CD44	MSR1					

We applied text-mining and information extraction methods specifically to these 112 sDEGs, and the same pattern of deregulation was observed in AD and GBM in PubMed abstracts associated with these diseases, and the opposite in LC. Among the most cited genes in papers related to the three diseases we found *CDKN1A*, *NFKBIA, MYD88* and *CD44*, which were up-regulated in AD and GBM, and down-regulated in LC (Supplementary Fig. S6). For instance, CD44 is involved in cell-cell interactions, as well as cell adhesion and migration, and it is down-regulated in LC, potentially favoring escape from tumoricidal effector cells^23^. By contrast, CD44, which may favor migration, is expressed weakly in normal brain tissues and overexpressed in GBM, with even greater intensity in high-grade glioblastoma than in low-grade astrocytomas^24^. In AD patients, CD44 appears to be up-regulated in blood vessel-associated astrocytes^25^ and in lymphocytes, perhaps playing a role in the immune response in affected tissues^26^. Hence, CD44 appears to be clearly associated to the three diseases and it is a good candidate to play a role in their co-morbidity.

On the other hand, *UCHL1, ENO2* and *CDK5* are among the most cited sDEGs with an AD−/GBM−/LC+ pattern (Supplementary Fig. S7). UCHL1 is a gene encoding a thiol protease that is specifically expressed in neurons, and it has been strongly associated with a poor prognosis when overexpressed in LC as it seems to promote metastasis by abrogating HIF-1α ubiquitination^27^. By contrast, this gene is down-regulated in GBM brains^28^, increasing NF-κB activity by activating IKK^29^, and in AD mouse brains where its up-regulation improves memory deficits^30^.

Through text-mining and information extraction, we were able to confirm that some of the most significant sDEGs were among the most cited ones in each of the disease-specific abstracts for all three diseases. These genes could be good candidates to study the molecular mechanisms shared between these three diseases.

## Discussion

Alzheimer’s disease, glioblastoma and lung cancer have been widely analyzed at the transcriptome level, although studying each condition separately. Thus, to our knowledge we present here the first joint analysis of these three diseases at the transcriptome level.

### Molecular substrates for the inverse co-morbidity between Alzheimer’s disease and lung cancer

We identified 92 processes that were deregulated in both AD and LC, none displaying a pattern of deregulation in the same direction. If we increase the threshold of significance from 0.05 to 0.1, only three pathways appear to be down-regulated in both AD and LC, and none were up-regulated in both diseases.

**Figure 3:**
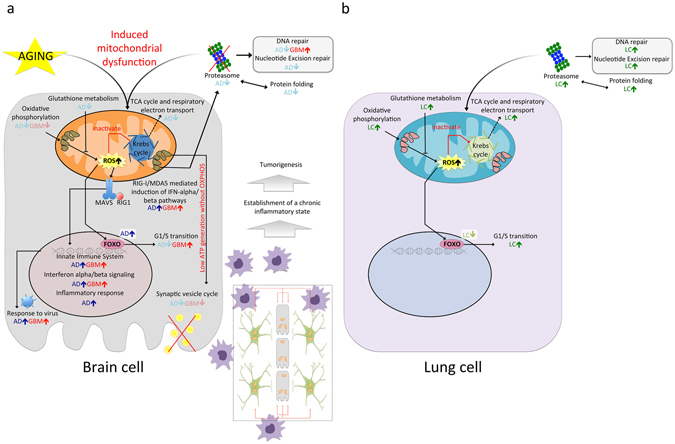
Potential scenario of the molecular pathways driving inverse and direct co-morbidities between AD and LC, and AD and GBM, respectively. (**a**) Potential molecular processes involved in the direct co-morbidity between AD and GBM. Proteasome inhibition could drive mitochondrial dysfunction, dampening oxidative phosphorylation and the TCA cycle. ROS are generated as a consequence of decreased rates of oxidative phosphorylation and glutathione levels. ROS generation activates the innate immune system, driving tumorigenesis by establishing a chronic inflammatory state through autocrine and paracrine loops. Synaptic transmission is decreased due to low levels of mitochondrial energy generation. (**b**) Potential molecular processes involved in the inverse co-morbidity between AD and LC. In this case, proteasome activity is enhanced, accompanied by increased levels of oxidative phosphorylation, TCA cycle and glutathione. As a consequence, lower levels of ROS are generated and so, redox sensitive transcription factors like FOXO are not activated, favoring the G1/S phase transition.

Many processes linked to down-regulated genes in AD and up-regulated genes in LC are related to or have been described to influence mitochondrial activity, such as “proteasome”, “protein folding”, “oxidative phosphorylation” and “glutathione metabolism”. Based on these results and previous publications, we propose the following scenario (see Fig. 3a). First, proteasome inhibition could promote mitochondrial dysfunction in AD^31, 32^, dampening oxidative phosphorylation and TCA cycle rates^33, 34^, while increasing the generation of mitochondrial Reactive Oxygen Species (ROS)^30, 31^. Oxidative phosphorylation is required for A549 lung adenocarcinoma cell line growth and thus, it might decrease tumor growth capacity in AD patients^32^. The increase in ROS is one of the first observable changes in AD brains, preceding even Aß plaque deposition and neurofibrillary tangle formation^35^, and it might be facilitated by diminished levels of glutathione^36^. Indeed, glutathione has potentially a major influence on chemoresistance in LC and a decrease in glutathione would make LC more sensitive to chemotherapy^37^. Additionally, mitochondrial ROS generation drives the activation of redox-sensitive transcription factors like FOXO, and these in turn induce cell cycle arrest in the G1 phase by repressing *CDK4* activity^38–41^. In our analyses, FOXO is AD+/LC− and it might play a role in the inverse co-morbidity between both diseases.

Finally, DNA repair is hindered as a consequence of the down-regulation of the proteasome^42^, which could make LC more sensitive to radiotherapy^43^ and at the same time, enhance mitochondrial dysfunction as a consequence of the DNA-damage caused by ROS^44^. Furthermore, proteasome and protein folding inhibition has been seen to attenuate cell migration and to increase cell death in non-small cell lung cancer cell lines^45^. Together, these distinct mechanisms are candidates to reduce the incidence of LC in patients with AD. The proposed chain of events that follow mitochondrial dysfunction as indicated above has also been described in the SK-N-MC neuroblastoma cell line when treated with the insecticide rotenone. This compound mediates NADH:ubiquinone oxidoreductase inhibition^46^, as confirmed by drug enrichment analysis using LINCS, and we found that the changes in gene expression produced in AD were similar to those observed when treating cells with such electron transport chain inhibitors.

### Molecular substrates for direct co-morbidity of Alzheimer’s disease and glioblastoma

There is little epidemiological data on the co-morbidity relationships between AD and brain tumors^7–9^. However, current evidence seems to point to the existence of direct co-morbidities between brain tumors and CNS diseases^16, 47, 48^. Additionally, a mouse model of AD displays high sensitivity to brain tumors^49^. We propose here that the significant number of genes deregulated in the same direction in both diseases could be an additional argument in favor of direct co-morbidity between AD and GBM. Interestingly, 10 of the 71 genes mutated in GBM^50^ are found in the overlap between sDEGs in AD and GBM (Supplementary Table S8).

Given the complexity of these diseases, it is obvious that these initial results will require additional confirmation with larger and better datasets. For example, the white matter control samples available in public databases (6 control samples in GSE35864) are insufficient to pair with the GBM cases, and grey matter controls were used as –admittedly imperfect- surrogates. As such, deregulation of similar sets of genes in both brain diseases might be due to different processes as a consequence of different cellular composition, with grey matter characterized by the presence of neuronal bodies and astrocytes, and white matter characterized by the predominance of axons and oligodendrocytes.

Comparing the sDEGs in AD and GBM, we found “oxidative phosphorylation”, “synaptic transmission” and “neurotransmitter release cycle” to be down-regulated in both diseases (AD−/GBM−), and “innate immune system”, “Rig-1 MDA-5-mediated induction of IFN alpha beta” and “interferon alpha beta signaling” to be up-regulated (AD+/GBM+: Table 1). The down-regulation of oxidative phosphorylation in cancer is usually accompanied by an increase in the rate of aerobic glycolysis, a process termed the Warburg effect^51^. Our finding that oxidative phosphorylation is down-regulated in both AD and GBM is supported by two independent studies proposing the use of methylene blue to treat both AD^52^ and GBM^53^ as it increases oxidative phosphorylation. As a consequence of the diminished oxidative phosphorylation, less mitochondrial energy is generated and synaptic transmission fails^54^. Finally, we propose that persistent activation of the immune system by different routes – including activation through Aß’s interaction with pattern recognition receptors^55^ and interferon alpha beta signaling – leads to the establishment of a local chronic inflammatory state. Such a state was previously proposed to be potentially related to bacterial^56^ or viral infection^57^, increasing the risk of developing brain tumors in AD patients at the epidemiological level^8, 9^ (Fig. 3b). This hypothesis is supported by associations between local chronic inflammation and cancer described previously in various tissues (e.g., chronic asthma and lung cancer or chronic pancreatitis and pancreatic cancer)^58^. In support of the hypothesis that inflammation promotes tumor growth, M2 macrophage polarization markers CD163 and MSR1 favor tumor development^59^, and they were found to be up-regulated in both AD and GBM.

Finally, the joint analysis of AD, GBM and LC, allowed us to propose potential molecular substrates of the direct and inverse co-morbidities between these diseases. These results could help to suggest drug repurposing and drug combination strategies in the future. Initial relevant examples could include combinations of proteasome and chaperone inhibitors, anti-oxidants and oxidative phosphorylation or TCA cycle inhibitors for LC treatment.

## Materials and Methods

### Comparison of control Brain and Lung samples

The LIMMA package was used to identify gene expression variations^60^ in control brain and lung samples, merging all the samples from the HG U133 Plus 2 microarray platform and using fRMA^61^ for data normalization. We applied a Principal Component Analysis to normalized expression data to evaluate the variability explained by the tissue’s origin. Gene Set Enrichment Analysis (GSEA)^13^ was applied to the pre-ranked list of genes based on z-scores to identify enriched pathways/processes. In addition, brain and lung specific genes were extracted from GTEx^14^, selecting the genes with a RPKM value of 1 or higher as those expressed in a given tissue. The genes expressed in the brain and lung were compared to each other. Those found to be expressed only on the brain were selected as brain-specific genes, while those found to be expressed only on the lung were selected as lung-specific genes. Enrichment analysis were conducted using gProfileR^15^ on brain and lung specific genes present in AD+/LC− and AD−/LC+ sDEGs using Gene Ontology Biological Processes^62^, KEGG^63^ and Reactome^64^ pathways.

### Microarray gene expression data

Raw data on gene expression (CEL files) were downloaded from the Gene Expression Omnibus (GEO, GSE* files http://www.ncbi.nlm.nih.gov/geo), ArrayExpress (EMTAB* files https://www.ebi.ac.uk/arrayexpress/) and The Cancer Genome Atlas (TCGA; https://tcga-data.nci.nih.gov/tcga/) for AD, GBM and LC (Supplementary Table S9). Studies undertaken on HT HG U133A, HG U133A, and HG U133Plus2 Affymetrix microarray platforms were selected to allow the frozen Robust Multiarray Analysis (fRMA) normalization method to be used and to reduce inter-platform differences^65^.

### Study design and meta-analyses

#### Gene expression data preprocessing and meta-analysis

Following steps defined previously^66^, disease-specific meta-analyses were conducted to increase the statistical significance and to minimize the noise intrinsic to gene expression measurements. Microarray datasets were normalized using fRMA^61^ from the R Affy package^67^. When multiple probes matched the same gene symbol, probes presenting the greatest inter-quartile range were selected. Only genes present in all the selected platforms were considered (using the *MetaDE.match* and *MetaDE.merge* functions of the MetaDE package^68^).

The P-values and effects sizes (ES) were calculated for every gene associated with each disease using the *ind.analysis* and *ind.cal.ES* functions of the MetaDE package^68^, respectively. Inter-study heterogeneity was evaluated calculating Q-statistic for every gene (assumed to follow a Chi-squared distribution under the hypothesis of homogeneity), representing quantile-quantile plots of observed vs. expected distributions. The Random Effects Model was applied to combine ES due to the existence of inter-study variability based on quantile-quantile plots (Supplementary Fig. S8). In addition, we conducted an alternative meta-analysis to double-check our results. Left- & right-tailed p-values were combined separately to identify down- and up-regulated genes using the maxP_OC function of the MetaDE package^68^ (Supplementary Fig. S2).

#### Particular case of GBM

Healthy brain samples are not available for obvious ethical reasons. To overcome this problem, we used five sets of healthy post-mortem control samples from different brain regions (anterior cingulate cortex, dorsolateral prefrontal cortex, post-central gyrus, entorhinal cortex and occipital lobe) and one set of epileptic brain biopsies as controls. One meta-analysis was performed with each of the 5 control sets (Supplementary Fig. S3). For the TCGA study, carried out on the HT HG U133A platform, epileptic samples were the only control samples available.

The sDEGs for GBM were selected restrictively as those deregulated in the same direction in all GBM meta-analyses. The small percentage of genes consistently expressed differentially in GBM (35% up-regulated and 26% down-regulated) may be explained by differences in gene expression profiles due to the delay between death and the time of tissue freezing, as described previously^17–19^, or by differences between brain regions, as indicated by Khaitovich^69^, who proposed that these variations were driven by distinct neuron-oligodendrocyte ratios^70^.

### Overlap analysis between sDEGs

Following an earlier strategy^12^, overlaps between pairs of diseases (AD/GBM, AD/LC and GBM/LC) were assessed by one-tailed Fisher’s exact tests on lists of sDEGs with three different FDR thresholds (FDR ≤ 0.05, 5 × 10^−4^ and 5 × 10^−6^), followed by the Bonferroni test to correct for multiple comparisons, and establishing the background number of genes as 14,538 (corresponding to the number of genes studied in the smallest platform, HT HG U133A). In the case of GBM, the intersection of the sDEGs found when using the different sets of control samples was selected.

### Functional enrichment analysis

A GSEA^13^ was performed separately on the ranked list of gene expression data for each disease (based on the z-scores obtained by the meta-analysis), and using annotations from Gene Ontology Biological Processes^62^, KEGG^63^ and Reactome^64^ pathways. Pathways and processes with a FDR ≤ 0.05 were considered significant. The pathways deregulated in more than 1 disease were selected for further analyses. We use the syntax “up-regulated pathways” and “down-regulated pathways” in the manuscript to refer to the pathways associated to up- and down-regulated genes according to the GSEA^13^ analyses. In addition, gProfileR^15^ was used to conduct gene set enrichment analysis when working on sets of sDEGs rather than the ranked list of differentially expressed genes.

### L1000 LINCS analysis

The 250 genes most strongly up- and down-regulated in AD and LC were used as gene expression signatures of the diseases, and compared against the LINCS L1000 library (http://www.lincscloud.org/), as performed previously^71^. The LINCS L1000 library is a large catalogue of gene expression signatures in cancer cell lines induced by drug treatment or gene knockdown. All signatures from all the cell lines were considered for the analysis. The Compute Connectivity on the Cloud (C3) environment was used to obtain the similarities between the AD and LC gene expression signatures with those of the drugs in L1000. The Weighted Connectivity Score^13^ was used as a similarity measure.

### Drug-set Enrichment Analysis

The drug enrichment analysis was carried out by applying the GSEA algorithm^13^ but using a ranked list of drugs and drug-sets instead of genes and gene sets. As in the classical GSEA, this approach reveals if a given group of drugs, a drug set, is preferentially located in one the extremes of a ranked list of drugs. In this case, the drugs were ranked according to the similarity of their L1000 gene expression with respect to the AD or LC gene expression signatures. A search of rotenone-like ETC inhibitors in the L1000 LINCS collection gave 30 different perturbagens, corresponding to 21 different drugs. We used the drug set enrichment approach to study if the drugs with similar or opposite signatures to those of AD and LC were enriched by this type of compound.

### Text mining analysis

Abstracts of papers in PubMed (http://www.ncbi.nlm.nih.gov/pubmed) associated to AD, GBM and LC were analyzed (disease-specific queries on Supplementary Information) looking for any of the genes mentioned in relation to each of these diseases. Then, the AD+/GBM+/LC− and AD−/GBM−/LC+ sDEGs (FDR ≤ 5 × 10^−6^) located over the third-quartile of genes mentioned in the abstracts of the three diseases were selected as the most relevant genes common to the three diseases.

## Electronic supplementary material

Supplementary Material
