# Supplementary material for: A molecular hypothesis to explain direct and inverse co-morbidities between Alzheimer’s Disease, Glioblastoma and Lung cancer
Source: Sci Rep. 2017 Jun 30;7:4474. doi: 10.1038/s41598-017-04400-6 (PMC5493619; doi:10.1038/s41598-017-04400-6)
Supplement: Supplementary file 1 — Supplementary Material [file 41598_2017_4400_MOESM1_ESM.pdf]

A molecular hypothesis to explain direct and inverse co-morbidities between Alzheimer's disease, Glioblastoma and Lung cancer

Authors

Jon Sánchez-Valle[1], Héctor Tejero[2], Kristina Ibáñez[3], José Luis Portero[4], Martin Krallinger[1], Fátima Al-Shahrour[2], Rafael Tabarés-Seisdedos[5], Anaïs Baudot[6]* Alfonso Valencia[1]**

[1]Structural Biology and Biocomputing Programme, Spanish National Cancer Research Centre (CNIO), Madrid, 28029, Spain

[2]Clinical Research Programme, Spanish National Cancer Research Centre (CNIO), Madrid, 28029, Spain

[3]Bioinformatics section, Institute of Medical and Molecular Genetics (INGEMM), Hospital Universitario La Paz, Madrid, 28046, Spain

[4]Department of Medicine. Hospital HM Sanchinarro, Madrid, 28050, Spain

[5]Department of Medicine, University of Valencia, Valencia, 46010, Spain

[6]Aix-Marseille Université, CNRS, Centrale Marseille, I2M UMR7373, Marseille, France

* Corresponding author. Tel: +33 0491269647; E-mail: anais.baudot@univ-amu.fr

** Corresponding author. Tel: +34 917328059; E-mail: valencia@cnio.es

Supplementary Figure S1. Principal component analysis of brain (red) and lung (green) control samples from studies included in the meta-analyses.

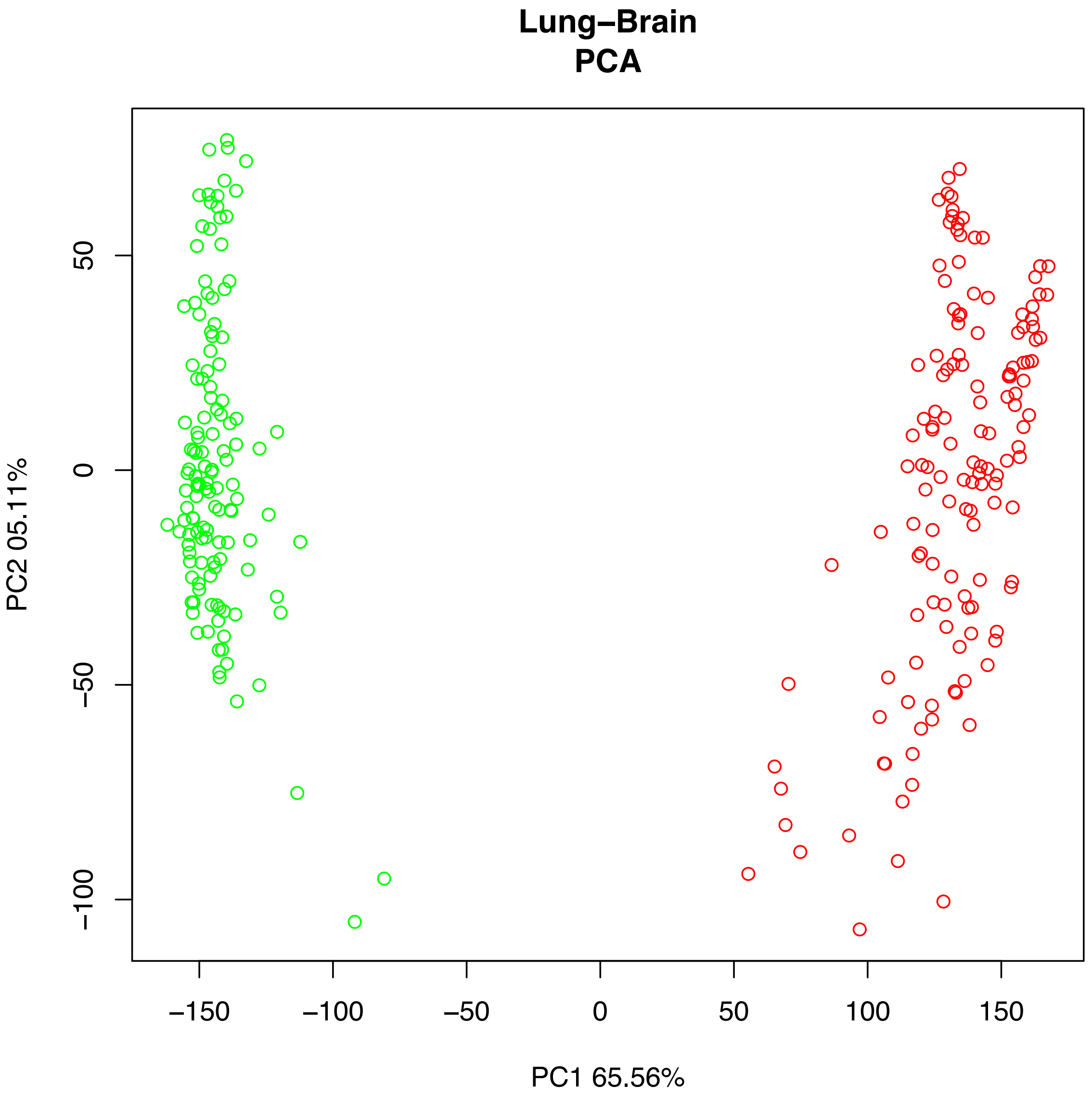

Supplementary Figure S2. Overlaps between significantly differentially expressed genes (sDEGs) in Alzheimer's disease (AD), lung cancer (LC) and glioblastoma (GBM) using an alternative meta-analysis approach that combines p-values. Grey circle size is proportional to the total number of sDEG identified in each disease with a FDR≤0.05. **A)** Pairwise comparisons of sDEGs identified as significantly up- and down-regulated with 3 FDR cutoffs (FDR≤0.05, $5*10^{-4}$ & $5*10^{-6}$) after gene expression meta-analyses in AD, LC and GBM. Orange and green cells indicate significant overlaps between sDEGs in the same and opposite direction, respectively (Fisher's exact test, FDR≤0.05). White cells correspond to non-significant overlaps (FDR>0.05). Numbers of disease-associated and overlapping sDEGs are indicated in their corresponding cell. **B)** Numbers of overlapping genes in the 3-ways comparison.

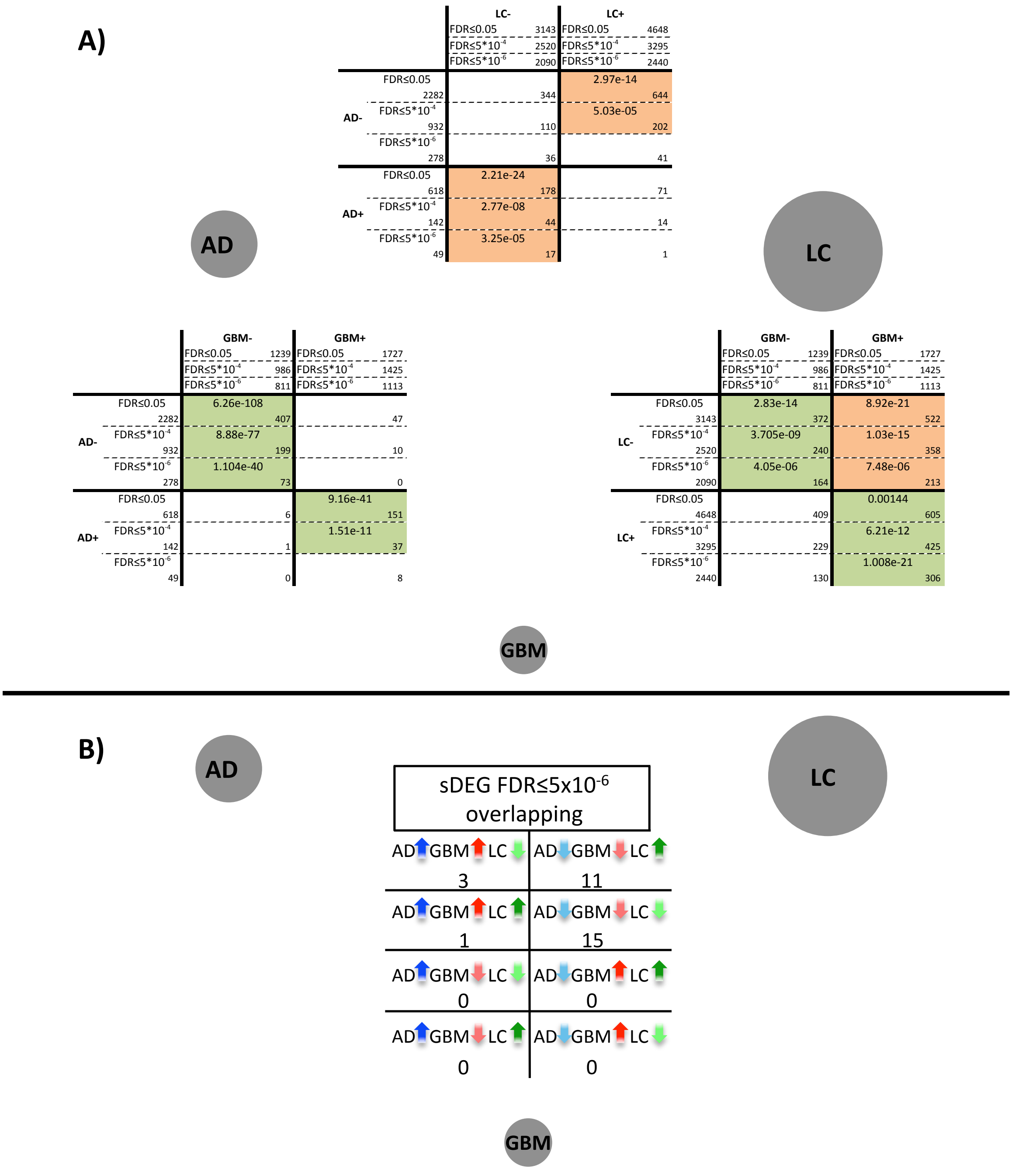

Supplementary Figure S3. **Meta-analysis step-by-step.** Meta-analyses were undertaken for each disease separately, normalizing data & matching probe names to gene symbols separately for each study, selecting only those genes common to every study (non-small-cell lung cancer in the upper side of the figure), calculating effect sizes and p-values for each gene of each study separately. Gene-specific ES and p-values were combined using Random Effects Model (REM) (in the case of ES combination) and maxP_OC for p-value combination (left- & right-tailed p-values were combined separately to discern between down- and up-regulated genes). To study GBM, 6 meta-analyses were conducted using different control sample sets, selecting as deregulated the genes that were consistently deregulated in every meta-analysis. In the special case of TCGA study, epilepsy brains were used as controls in all the meta-analyses.

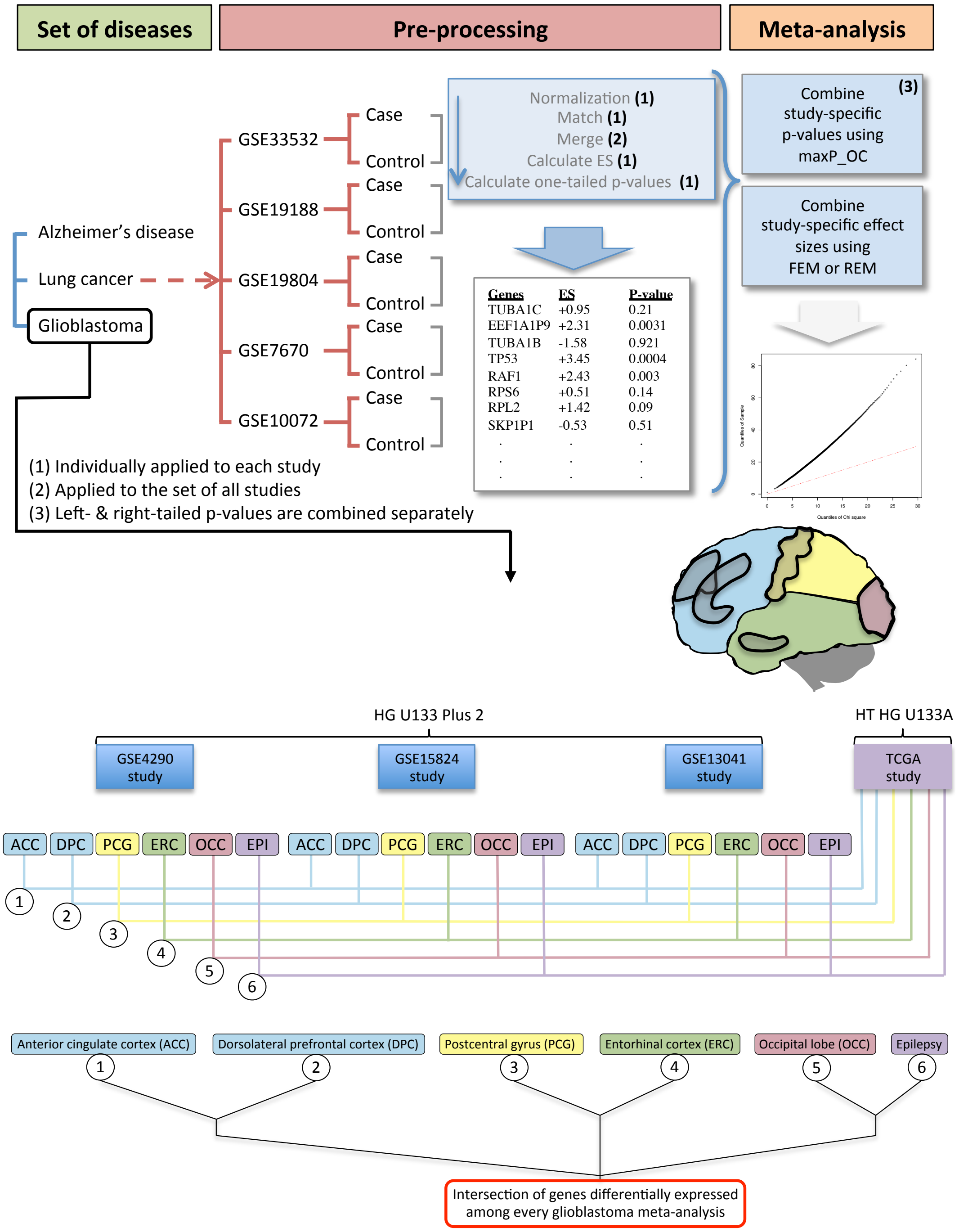

Supplementary Figure S4. Genes significantly differentially expressed in all the control-specific meta-analyses. Analysis of genes consistently expressed differentially in GBM (FDR≤0.05) using different control samples. Numbers of sDEGs in each glioblastoma meta-analysis (combining ES with Random Effects Model) and in their intersections are indicated in yellow and blue boxes (up- and down-regulated, respectively). 2,405 and 1,603 genes are consistently up- and down-regulated respectively in all the meta-analyses with an FDR cutoff of 0.05, diminishing the number to 1,047 up- and 664 down- with a FDR ≤ $5 \cdot 10^{-6}$ (numbers of sDEGs with different FDR cutoffs are indicated in Fig 1).

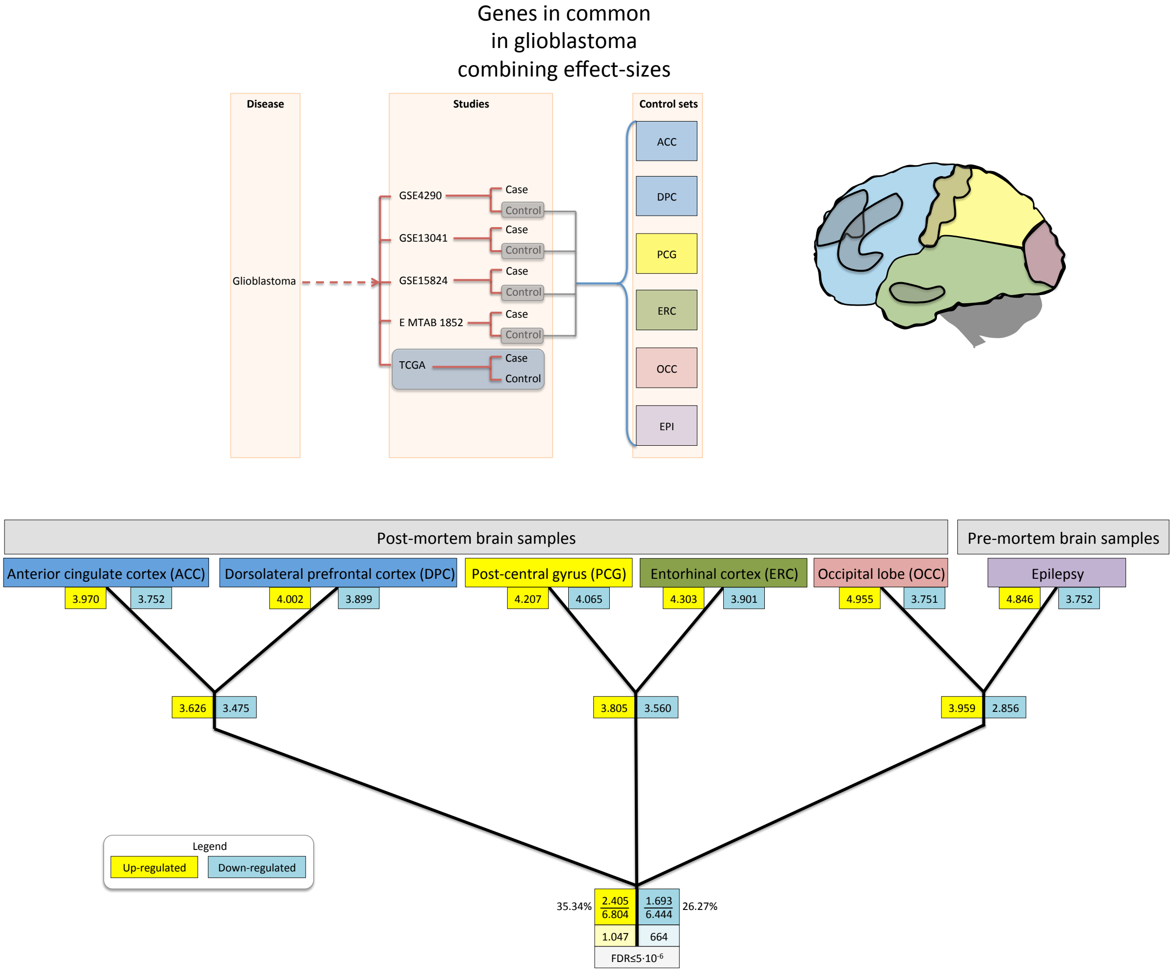

Supplementary Figure S5. Overlaps between significantly differentially expressed genes (sDEGs) in Alzheimer's disease (AD), lung cancer (LC) and glioblastoma (GBM) in the three-ways comparisons. Grey circle size is proportional to the total number of sDEG identified in each disease with a FDR≤0.05. Numbers of overlapping genes in the 3-ways comparison are indicated.

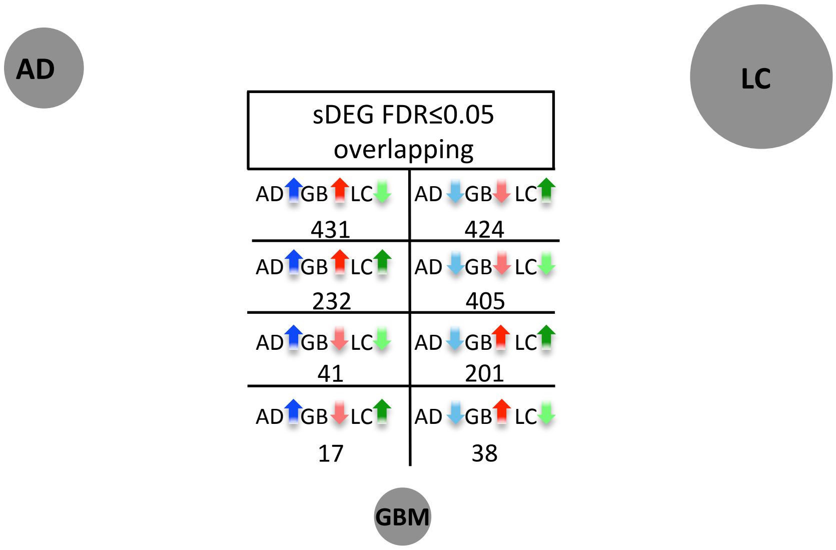

Supplementary Figure S6. Number of times each gene significantly up-regulated in AD and GBM and down-regulated in LC (FDR≤$5x10^{-6}$, see Table 2) appears in disease-related abstract based on PubMed searches. The genes located over the third-quartile of genes mentioned in the abstracts of the three diseases were selected as most relevant genes common to the three diseases.

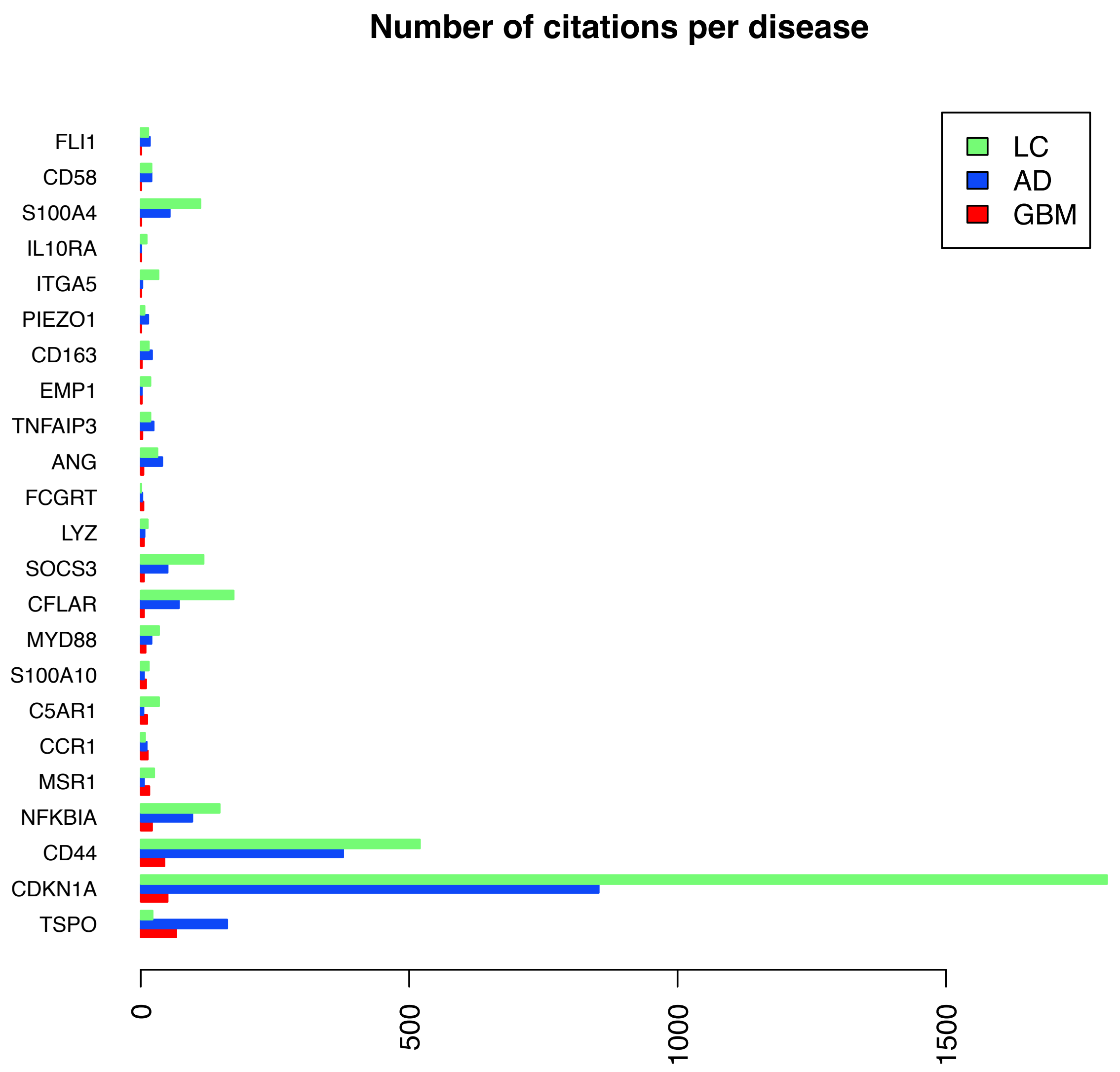

Supplementary Figure S7. Number of times each gene significantly down-regulated in AD and GBM and up-regulated in LC (FDR≤$5x10^{-6}$, see Table 2) appears in each disease-related abstract based on PubMed searches. The genes located over the third-quartile of genes mentioned in the abstracts of the three diseases were selected as most relevant genes common to the three diseases.

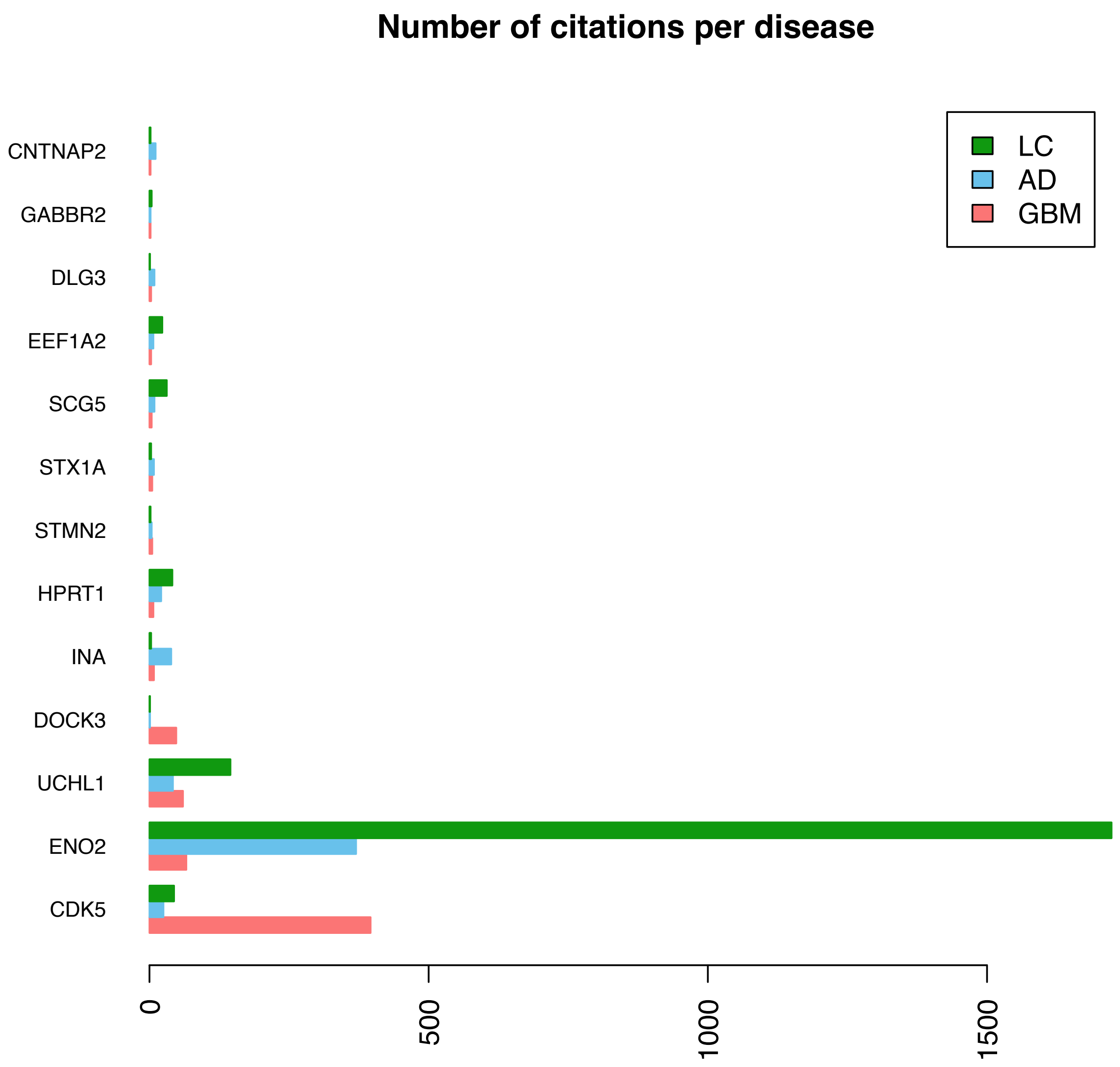

Supplementary Figure S8. Testing for homogeneity of study effects gene by gene. Overall test results are shown by the plot of the observed vs. expected Q quantiles for Alzheimer's disease, glioblastoma and lung cancer. Red and black dots indicate expected and observed Q values, respectively.

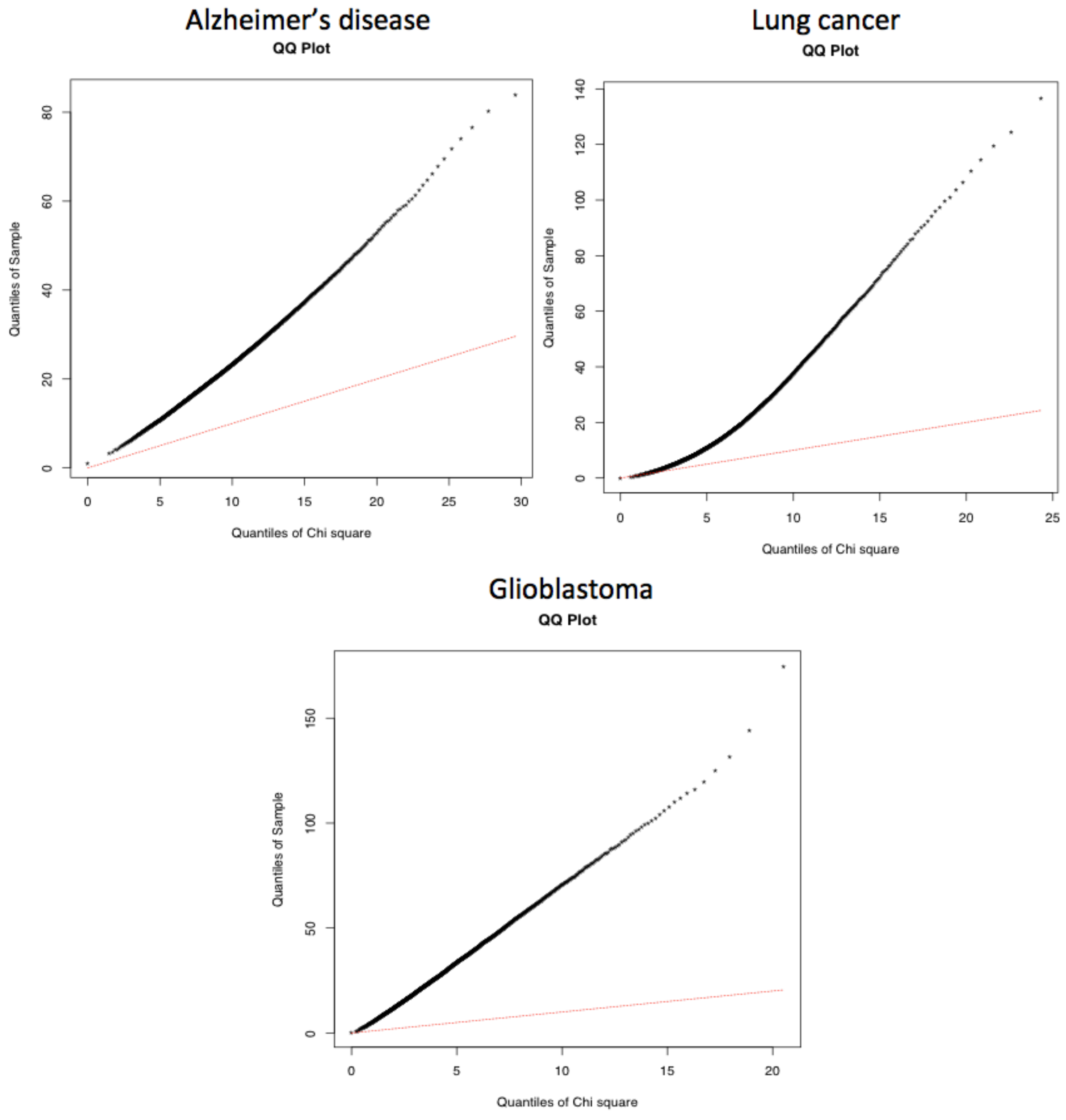

Supplementary Table S1. Pathways/Processes significantly up-regulated (FDR ≤ 0.05) in lung control samples when compared to brain control samples according to GSEA analyses.

Pathways down-regulated in brain controls compared to lung controls

Pathways	FDR	Pathways	FDR
defense response	0	immune effector process	0.003987551
cytokine cytokine receptor interaction	0	lymphocyte activation	0.00480311
immune response	0	bladder cancer	0.005413096
inflammatory response	0	t cell activation	0.0055061076
complement and coagulation cascades	0	downstream tcr signaling	0.005489926
leishmania infection	0	peptide ligand binding receptors	0.005843524
chemokine receptors bind chemokines	0	generation of second messenger molecules	0.0068624746
immune system process	0	collagen formation	0.006978542
response to wounding	0	jak stat cascade	0.007591794
interferon alpha beta signaling	0	positive regulation of translation	0.0076117185
hematopoietic cell lineage	0	rig i mda5 mediated induction of ifn alpha beta pathways	0.007891764
cellular defense response	0	epidermis development	0.008023275
response to external stimulus	0	wound healing	0.008151
immunoregulatory interactions between a lymphoid and a non lymphoid cell	0	leukocyte transendothelial migration	0.008336109
		arachidonic acid metabolism	0.008514864
viral myocarditis	0	cell proliferation go 0008283	0.008540774
graft versus host disease	0	regulation of body fluid levels	0.008599725
response to other organism	0	regulation of cell proliferation	0.009451175
intestinal immune network for iga production	0	i kappab kinase nf kappab cascade	0.010087918
complement cascade	0	regulation of lymphocyte activation	0.0099648945
autoimmune thyroid disease	0	cytokine and chemokine mediated signaling pathway	0.011112674
antigen processing and presentation	0	positive regulation of cell proliferation	0.011754328
allograft rejection	0	regulation of i kappab kinase nf kappab cascade	0.012154249
response to biotic stimulus	0	antigen presentation folding assembly and peptide loading of class i mhc	0.013633595
innate immune system	0		
locomotory behavior	0	tcr signaling	0.0148851555
systemic lupus erythematosus	0	drug metabolism other enzymes	0.015206517
cytokine production	2.1581087E-4	skeletal development	0.015872661
asthma	2.0810336E-4	degradation of the extracellular matrix	0.017060848
response to virus	2.0092737E-4	ribosome	0.017860375

integrin cell surface interactions	2.443099E-4	ectoderm development	0.01784235
multi organism process	2.8204272E-4	positive regulation of multicellular organismal process	0.018824646
interferon signaling	2.7322888E-4	positive regulation of signal transduction	0.01865462
cytokine signaling in immune system	5.395782E-4	ecm receptor interaction	0.021106495
nod like receptor signaling pathway	5.673718E-4	transcriptional regulation of white adipocyte differentiation	0.021736441
formation of fibrin clot clotting cascade	7.634044E-4	response to elevated platelet cytosolic ca2	0.023328474
primary immunodeficiency	8.275119E-4	programmed cell death	0.023638468
extracellular matrix organization	8.830396E-4	peptide chain elongation	0.023648998
cytosolic dna sensing pathway	0.0013698708	apoptosis go	0.024080545
tissue development	0.0013756277	positive regulation of i kappab kinase nf kappab cascade	0.024049316
mesoderm development	0.0013755022	natural killer cell mediated cytotoxicity	0.026526755
humoral immune response	0.0013777373	negative regulators of rig i mda5 signaling	0.026558146
cytokine biosynthetic process	0.0014174222	positive regulation of immune system process	0.028429935
small cell lung cancer	0.0015929193	homeostasis of number of cells	0.030231172
interferon gamma signaling	0.001591027	type i diabetes mellitus	0.030817948
cell activation	0.0017561248	formation of the ternary complex and subsequently the 43s complex	0.03449591
cell surface interactions at the vascular wall	0.0017825876		
jak stat signaling pathway	0.0019060413	srp dependent cotranslational protein targeting to membrane	0.03501463
regulation of immune system process	0.0019298791	homeostatic process	0.036002323
cytokine metabolic process	0.0019206477	biosynthesis of the n glycan precursor dolichol lipid linked oligosaccharide llo and transfer to a nascent protein	0.03597314
regulation of cytokine biosynthetic process	0.0019433827		
antigen processing cross presentation	0.002165943	negative regulation of programmed cell death	0.03605978
blood coagulation	0.0023812747	organ development	0.03992484
response to stress	0.0023645568	regulation of translation	0.039863244
transmembrane receptor protein serine threonine kinase signaling pathway	0.0024325717	negative regulation of biological process	0.041047987
		regulation of multicellular organismal process	0.042857684
transforming growth factor beta receptor signaling pathway	0.002952866	er phagosome pathway	0.04302385
coagulation	0.002978724	signaling by bmp	0.043864276
response to chemical stimulus	0.0029264654	lipoprotein metabolism	0.04396789
toll like receptor signaling pathway	0.0029267236	negative regulation of cellular process	0.0449526
proteolysis	0.0029794879	viral reproductive process	0.04479058
leukocyte activation	0.0034970695	cellular cation homeostasis	0.04475715

hemostasis	0.003656999	class a1 rhodopsin like receptors	0.044991665
lysosome	0.0037412765	lymphocyte differentiation	0.0498416

Supplementary Table S2. Pathways/Processes significantly up-regulated (FDR ≤ 0.05) in brain control samples when compared to lung control samples according to GSEA analyses.

Pathways up-regulated in brain controls compared to lung controls

Pathways	FDR	Pathways	FDR
transmission across chemical synapses	0	ca dependent events	0.0014552058
neuronal system	0	darpp 32 events	0.0014386949
synaptic transmission	0	system process	0.0015776071
transmission of nerve impulse	0	axonogenesis	0.0017640328
neurotransmitter receptor binding and downstream transmission in the postsynaptic cell	0	dag and ip3 signaling	0.0021478976
		neuron development	0.0029768327
nervous system development	0	olfactory transduction	0.0032582425
gaba receptor activation	0	transmembrane transport of small molecules	0.0033403481
neurotransmitter release cycle	0	cytoskeleton dependent intracellular transport	0.005424392
ion channel transport	0	glycolysis	0.0058042174
neurological system process	0	brain development	0.006085304
activation of nmda receptor upon glutamate binding and postsynaptic events	0	neurite development	0.0060263583
		g alpha z signalling events	0.006817662
central nervous system development	0	activation of kainate receptors upon glutamate binding	0.0067721517
gaba synthesis release reuptake and degradation	1.0558233E-4	phospholipase c mediated cascade	0.006887275
trafficking of ampa receptors	9.8040735E-5	secretory pathway	0.0075761024
glutamate neurotransmitter release cycle	9.150469E-5	monovalent inorganic cation transport	0.008362685
long term potentiation	8.578564E-5	metal ion transport	0.010949573
potassium channels	8.073943E-5	amyotrophic lateral sclerosis als	0.011583868
ligand gated ion channel transport	7.6253906E-5	long term depression	0.0139110405
unblocking of nmda receptor glutamate binding and activation	7.224054E-5	transport of inorganic cations anions and amino acids oligopeptides	0.013908565
regulation of insulin secretion	6.862851E-5	generation of a signal involved in cell cell signaling	0.013884332
ras activation upon ca2 infux through nmda receptor	1.2997272E-4	oocyte meiosis	0.013802813
creb phosphorylation through the activation of camkii	1.5599813E-4	gap junction	0.015670598
post nmda receptor activation events	1.7942587E-4	nitric oxide stimulates guanylate cyclase	0.017142821
inhibition of voltage gated ca2 channels via gbeta gamma subunits	2.2878876E-4	amine catabolic process	0.018208954
opioid signalling	2.7551333E-4	signaling by robo receptor	0.021170063
voltage gated potassium channels	2.6491669E-4	amino acid catabolic process	0.022108197
gaba b receptor activation	2.5510494E-4	microtubule based process	0.022088395
integration of energy metabolism	2.697323E-4	cation transport	0.023137355

interaction between l1 and ankyrins	3.067987E-4	axon guidance	0.02309424
generation of neurons	2.9657208E-4	signalling by ngf	0.025800014
cell cell signaling	3.5307676E-4	axon guidance	0.030235026
neuron differentiation	4.065564E-4	ngf signalling via trka from the plasma membrane	0.034563936
calcium signaling pathway	4.1569516E-4	inhibition of insulin secretion by adrenaline noradrenaline	0.03794163
neuroactive ligand receptor interaction	4.0346885E-4	glucagon signaling in metabolic regulation	0.037487157
inwardly rectifying k channels	3.9194117E-4	taste transduction	0.037176404
neurogenesis	4.189271E-4	regulation of insulin secretion by glucagon like peptide1	0.04036897
alanine aspartate and glutamate metabolism	5.3724484E-4	gluconeogenesis	0.040932
exocytosis	6.4900506E-4	axon guidance	0.044443894
l1cam interactions	9.830337E-4	amino acid derivative metabolic process	0.04481504
plc beta mediated events	0.001043489	g alpha1213 signalling events	0.044360034
creb phosphorylation through the activation of ras	0.0011682987	proximal tubule bicarbonate reclamation	0.0444665
cellular morphogenesis during differentiation	0.0012887896	glucose metabolism	0.04737697
ion transport by p type atpases	0.0013841481	phosphatidylinositol signaling system	0.047361452
cardiac muscle contraction	0.0013996053	recycling pathway of l1	0.04728522
insulin synthesis and processing	0.0014135403	g protein coupled receptor protein signaling pathway	0.047915023
potassium ion transport	0.0014868407	amino acid transport	0.048897058

Supplementary Table S3. Significantly differentially expressed genes (sDEGs, FDR ≤ 0.05) in Alzheimer's disease, lung cancer and glioblastoma.

Alzheimer's disease		Lung Cancer		Glioblastoma	
**Up**	**Down**	**Up**	**Down**	**Up**	**Down**
MT-ND5	CALM2	AC091304.2	EEF1A1P9	RPS2	CALM2
RPS29	TUBA1A	RP11-234A1.1	RP11-290D2.4	B2M	RP11-50D9.1
AC107983.4	NGFRAP1	RP11-475C16.1	EEF1A1P19	RPS19	MT-ND5
RP11-761N21.2	BASP1	RPL13AP5	B2M	RP11-603J24.18	NGFRAP1
PTMAP5	TUBB2A	AC016739.2	AC004453.8	H3F3AP4	RTN4
RP11-282O18.3	MTCH1	TMSB10	TMSB4XP8	VIM	TUBB2A
GJA1	CHN1	RP11-425L10.1	EEF1A1P6	AC011737.2	RP11-677N16.1
RP11-396K3.1	PPIAP29	IGLL5	FTH1P5	HMGN2P5	ATP5A1
MT2A	UBC	RPL39P3	RP11-50D9.1	HLA-C	HSP90AA1
RP11-403P17.4	RP11-121L10.3	RPS29	FTLP3	CD63	COX6A1
RP11-771F20.1	SKP1P1	CTD-2192J16.15	HLA-C	HLA-A	MTCH1
RPS19	RP11-887P2.3	RP11-464D20.2	AC107983.4	SPP1	COX6C
FAM107A	STXBP1	RPL9P25	FTH1P20	PTPRZ1	NDUFA4
MT1E	RTN4	IGHGP	UBC	YBX1P1	DYNLL1
RPS15AP1	TUBA1C	AC007969.5	VIM	SDCBP	RP11-887P2.3
AIDA	TMSB10	RPS4X	HLA-A	HIF1A	TMEM66
PABPC3	HSP90AA1	RPS2	CALM2	LAPTM4A	SOD1
RP11-36C20.1	BEX1	ACTG1	IFITM3	DBI	PARK7
MT1P2	GAPDH	PPIAP11	ANXA2	RPLP0P6	PFN2
PLP1	TUBA1B	PPIAP29	RP11-603J24.18	SEC61G	COX7CP1
CSRP1	CLSTN1	RPL27	EEF2	BTF3	CCNI
RP11-118B22.3	PPIAP11	RPS17	S100A10	PTTG1IP	SERINC1
MT1H	LDHB	RPL38	RPL11	UBE2E1	HIGD1A
PRDX6	HK1	RPS19	RPL34	SPARC	MLLT11
RP11-347C12.2	NDUFB8	IGHA2	NPC2	PPP1CC	NPTN
LAPTM4A	COX7A2	IGLC2	FAUP1	RP11-193F5.4	PEBP1
MXI1	CLTC	RP11-526L22.1	A2M	RBBP7	ATP5B
PFKFB3	ALDOA	AC011737.2	HLA-B	GPX1P2	RTN3
RPL13A	COX6A1	RPS15AP1	ITM2B	RAB31	COX8A
ANP32B	TMEM66	FAM153C	RPL3	A2M	BEX1
AC011498.1	PARK7	RPL15P3	HLA-DRA	PRDX6	RP11-169K16.7
HIF1A	ARL6IP1	RPS7P10	CTD-2287O16.1	EIF3E	CPE
HLA-C	PI4KAP1	GAPDH	CEBPB	ANP32B	GABARAPL2
AL162497.1	ACTG1	RP11-466H18.1	EIF3L	PSMB1	COX5B
IFITM3	NPTN	AC079250.1	LAPTM4A	ITGAV	AC073869.1
RP11-146N23.1	ATP5A1	RP11-110C15.4	MT2A	BZW1	UCHL1
PTTG1IP	DNM1	AC018462.3	CTSH	S100A6	PLP1
AL592188.3	NDUFA4	RPS16	SLC25A3	NDUFS6	EIF1B
AL021977.1	DYNLL1	H3F3AP4	RP11-677N16.1	SNRPEP4	NDUFA1
NDRG1	CYCSP55	RPL8	TOMM7	CYFIP1	SEPW1
HSPA1A	HIGD1A	PABPC1	CD81	PMP22	ISCU
ALDH9A1	NSF	AC144530.1	CEBPD	RP11-367J7.4	CLSTN1
IST1	PFN2	UBA52	MYL12B	HMGN1P36	GDI1
PTMAP2	B3GNT1	RPL35	TUBA1A	EIF3FP3	BASP1

TNS3	MLLT11	RPL13P12	GABARAP	TCEA1	NDUFB8
DBI	SOD1	RPS5	TSPAN13	PMP2	PJA2
CASC3	TSPYL4	RP11-36C20.1	CD9	EEF1D	UQCRFS1P1
MT1G	NARS	RP5-1068H6.3	OAZ1	RP11-466P24.2	MGST3
PABPC1P3	HIGD1AP14	PTMAP5	NFKBIA	HLA-B	QDPR
HLA-A	OLFM1	LDHA	SPARCL1	BIRC2	PEA15
RP11-87C12.5	BLCAP	RPL10AP6	MTCH1	C3	CHN1
MT1X	ATP6V1E1	RP11-408P14.1	SDCBP	APEX1	NDUFAB1
YBX1P1	BNIP3	ALDOA	MSN	CCT3	WSB2
DENND5A	ENO2	PABPC3	NGFRAP1	SSR4	HK1
NOTCH2NL	SLC25A3	RPL29P11	CNBP	HSPB1	OAT
NFKBIA	RP11-365D23.4	RPS26P47	RTN4	IST1	ATP6V1E1
EFHD1	NDUFS5	PTMAP2	GNG5	SNRPGP10	DYNLRB1
SLC6A8	TCEAL4	RPS7P1	ANXA2P2	ANXA5	CSRP1
CEBPD	FAM127A	RPLP0P6	SLPI	RP11-111K18.1	ATP1B1
PTMAP3	LDHA	GAPDHP1	C3	GNG5	RP11-20I23.1
BMI1	ATP6V0E2	TIMP1	ZFP36	TAF7	YWHAB
RP11-466P24.2	TSPAN13	COX6A1	CCNI	YBX1P10	MXI1
SYNM	SLC9A6	S100A11	PLS3	RP11-973H7.2	USP11
AC005035.1	OAT	CHCHD2P2	ANXA1	PFKFB3	ATP6V1B2
ACADVL	CCDC92	CLTC	NCOA4	NFKBIA	PNMA1
DVL1	MAGED1	PRDX1	RNASE1	RPN2	GABARAPL3
PTPRZ1	COX7A2L	EEF1B2P2	S100A4	WDR45L	TERF2IP
SPP1	RTN3	RP6-11O7.2	ANXA5	DEK	MZT2B
RP11-79D8.2	MYL12B	SSR4	LTA4H	PGM1	ATP6V0E2
ZCCHC24	AC040173.1	HNRNPKP4	CAP1	PLS3	UQCRC1
RP11-518D3.3	CUTA	IFITM2	ARPC2	SSR2	RP11-697E2.6
GLTP	TUBB4B	COX6C	NPTN	HLA-E	B3GNT1
ADD3	YWHAQ	LDHB	PPT1	SCP2	TSPAN7
EIF4BP6	TCEAL2	RP3-507I15.1	RPL36AL	SRSF9	NSF
PAQR6	SNX3	EEF1D	DSTN	RP4-604K5.1	BLCAP
RP4-545K15.3	EIF1B	EIF3E	MT1P2	HERPUD1	PI4KAP1
EEF1D	NELL2	SUMO2	ATF4P3	NPC2	NEDD8
MT1F	AC073869.1	PABPC1P3	ALDH2	CEBPD	ALDOC
NACAP1	MOAP1	SLC25A5	HMGN3	CAPZA1	RRAGA
CYFIP1	COX6C	COL6A3	SAT1	RP11-977G19.10	PTGDS
SDR39U1	WSB2	PARK7	RP11-466P24.2	RP11-603J24.7	ALDH2
SETD1B	PPT1	RP11-467L20.9	HOPX	S100A10	TSPYL4
SUMO4	TPI1P1	RPL36	LPCAT1	DDR1	ACO2
HMGB1P5	DYNLT3	PPA1	FTH1	WDR83OS	PPP3CA
METTL7A	TSPAN7	COX7CP1	RGS2	KARS	SCG5
LGALS3	COX5B	PTGES3	RGCC	WBP5	STXBP1
PURA	SLC35E2B	AP000350.10	PSAP	HNRNPAB	ATP9A
HLA-B	NDUFA1	COX7A2	NOP10	CEBPB	ATP1A1
CTD-2341D24.1	ARNT2	RP11-887P2.3	MT1E	PPIB	DYNLT3
UBA2	COX8A	SOD1	MT1H	EIF3D	PFKM

RGCC	PTPRK	NDUFA4	TMEM66	SEC11A	MOAP1
KAT2A	COX6B1	HSPB1	CDIPT	EIF4A3	ENO2
XPO6	NEFM	DYNLL1	HEXB	HEY1	BCL2L2-PABPN1
ARHGEF18	USP11	IFITM1	PDLIM1	MSN	ATP6V0B
PUM2	TBCAP1	HMGN1P36	ISCU	SAT1	SYS1-DBNDD2
PRRC2C	BTBD1	JTB	CAPNS1	ATRAID	GOT2
IFITM2	ATP9A	SRP9P1	HLA-E	ATP8B5P	AKAP11
CLDN11	RPS26P47	TPI1P1	LIPA	RP11-700P18.1	SNRPN
PMP2	SH3GL2	HSP90AA1	SDC4	AKR1A1	CS
RP11-700P18.1	CHCHD2P2	HIF1A	DBI	EIF3G	SLC9A6
SEMA4C	EID1	PFN1	CD59	SNRPB2	GAP43
SUN2	TUBB3	PTMAP3	IER2	SRSF6	FAM107A
SNRPGP10	NDUFS6	KRT18	STARD7	SGK1	MAP1A
PLEKHB1	NDUFA3	TUBB	SGK1	TOP1	FAM108A7P
ITGAV	DCTN1	COX6B1	DPYSL2	YTHDF1	BEX4
PNISR	RP11-169K16.7	TALDO1	VAMP8	DHX15	PLEKHB1
DDIT4	ITM2B	COX8A	RP11-20I23.1	SOX9	SERINC3
H1F0	NUAK1	SNRPD2	RP11-231C14.4	QARS	ARNT2
RAB31	STMN2	PSMA6P1	IFI27	HNRNPM	GUK1
SGK1	SPOCK1	EIF3CL	HLA-DQB1	RP4-545K15.3	PNMAL1
CSNK1A1	ATP5B	IGLL3P	SRGN	ZMPSTE24	NDUFS2
DUSP8	RP11-799B12.4	YWHAQ	RHOA	TMED10	MLF2
ATP1A2	GPX4	TRMT112	GLTSCR2	MEST	KIFAP3
RALGDS	GPM6A	RPN2	CSRP1	COPB1	YWHAH
ARHGEF6	UCHL1	TUBBP2	TNS3	SHFM1	RP11-799B12.4
KLHL21	NDUFC2-KCTD14	UQCRQ	RP11-796E2.3	KRT10	CCDC92
HSPB1	EIF4H	SSR2	ATP1A1	HNRNPA3P5	PINK1
CEBPB	SERINC1	GPX4	GRN	IMPDH2	PCMT1
ATP1B2	RTN1	NDUFS5	CREG1	RP11-796E2.3	CMC2
SAT1	PSMD2	ADAR	RP11-367J7.4	RP11-514O12.4	USP32P2
FBXO2	NAP1L3	ATP5B	RPL22	RP11-59H1.3	MRFAP1L1
GAK	SCG5	TXNDC5	PCBP1	CCT6A	MAP2K1
CTSH	AP000350.10	ERH	SFTPB	KCTD12	NRN1
APOD	PPA1	CYCSP55	S100A8	RP11-721G13.1	NDRG4
SLC7A5	ISCU	TXN	CYFIP1	HEXB	RTN1
VGLL4	TERF2IP	RP11-282O18.3	STOM	ODC1	AL021977.1
WBP1L	STMN4	PHB2	FOS	NOTCH2NL	POLR2I
SOX9	ATP1A1	LGALS3BP	SLC38A2	FBL	VDAC3
KTN1	ATP5J	ATP5C1	EIF4B	ARF4	APOD
KIAA0930	CCT8	CUTA	MYH9	SERF2	TMEM14A
TRAK2	SERPINI1	SEC61G	SERPING1	CSRP2	WDR47
HSPA2	NRGN	RP11-118B22.3	SNX3	RP11-553L6.5	KLHL2
RP11-603J24.7	APMAP	RP11-169K16.7	SPARC	RASSF2	NELL2
PHF11	GLO1	CCT8	SERINC1	SEC13	PLEKHB2
VIM	TBCB	NDUFA1	GJA1	TWF1	SPOCK1
ZBTB16	PEBP1	ATP1B1	TAGLN	GLTSCR2	DCTN1

CERS1	CAP1	CCT4	DHCR24	ANXA2	EPS15
GOLGA4	AP000304.12	TUBB4B	HLA-DMA	TMEM123	DNAJC6
DEK	PSMC1P1	ATP5I	CD55	TIMP1	MAGEH1
PLEKHM2	GOT2	RP11-603J24.7	ASAH1	COPB2	SV2A
SDC4	SNAP91	SPCS1	RP6-218J18.2	HLA-G	TF
RP4-775C13.1	SLC25A5	IGKV1-39	PEBP1	MFSD1	APP
SECISBP2L	AC007318.5	MDH2	SEPW1	GADD45A	IDI1
VAT1	PFKP	GSTP1	HLA-J	TMEM147	PPP2R1A
RP11-255B23.1	STX12	RPS10L	GSTO1	SNRPD3	R3HDM2
HMBOX1	PRKCZ	PSME2P2	C1QB	LAP3	GPM6A
GNG5	EPS15	RP5-977B1.10	METTL7A	PPP1R8	AASDHPPT
PIK3C2B	FKBP3	XRCC6	GPX3	FSTL1	CALM3
CLASRP	WASF1	NDUFC2-KCTD14	JUNB	PSME1	BTBD3
USF2	PFKM	TGFBI	ATP6V1E1	MAFB	ATP6V1H
SASH1	NCKAP1	AC007318.5	APOC1	ARHGEF6	CRMP1
HIP1R	SYNGR3	NARS	LAMP3	TIPARP	APLP1
DTX2P1	PNMAL1	PSMB1	HADHB	PRDX4	TTC19
ZFP36	RP11-93O14.2	SNRPEP4	RP11-403P17.4	UBE2L6	KIF5C
A2M	SLC12A5	ST13P4	TCEAL4	DDIT4	CBX6
MKNK2	HOPX	KARS	WDR83OS	SPG21	NAP1L3
SALL2	INA	ATRAID	HBA2	VAT1	OSBPL1A
RP11-553L6.5	THY1	GOLPH3	IST1	VGLL4	TCEAL2
TIPARP	IDI1	PPP1CC	CYB5A	CTSL1	SLC25A4
STOM	TAGLN3	DAD1	CSDE1	TXNIP	ENC1
PLOD3	MAST3	EIF4H	PGM1	WTAP	MGLL
HP1BP3	PPP3CA	UQCRFS1P1	MLPH	IDH1	NUAK1
BAG3	RP11-198M15.1	HINT1	SCP2	SND1	PPP2R2B
ATG2A	TALDO1	GLO1	CSDA	BZW2	EVL
LRP4	AC067852.1	AKR1A1	PMP22	UXT	CISD1
RP11-316E14.6	PINK1	SPINT2	MAT2B	ILF2	PAQR6
PLXNB2	RRAGA	SUMO2P1	MT1G	CCNG1	AC067852.1
ABLIM1	ATP5L	H2AFZ	DNAJA1	SRGN	WASF1
C3	HMGN2P5	RBBP7	EIF3G	HMGB2	ENSA
sept-08	ATP6V1B2	BTF3	C1QA	TXNRD1	P4HTM
TOB1	AKAP11	S100A11P1	FABP5P2	HSPA5	ATP1A2
SON	UQCRQ	SNRPGP10	YPEL5	MLH1	PLD3
TERF1P5	HINT1	CAPZA1	ACTN4	ROBO1	FAM171A1
DDR1	GARS	SHC1	SH3BGRL3	UNC50	SIRPA
LMF2	DDHD2	EIF2S2P4	BIRC2	RDBP	ANKRD46
MT1M	DRG1	NDUFAB1	RAB31	PSME2P2	HPRT1
AMOTL2	APP	UFC1	GABARAPL2	SERPINB6	EFHD1
DNMT1	RPL10AP6	EIF3D	TRIM22	ERBB2IP	MAP7D1
NEAT1	NDUFB3	RP11-365D23.4	APLP2	NAGK	STK39
PHGDH	ACO2	NSA2	FAM127A	CORO1C	SUCLA2
CALCOCO1	PJA2	CTSA	HBB	TXNDC5	NDEL1
PCBP4	PSMA4	BZW1	CMTM6	DFNA5	HSPH1

BAZ2B	TCF25	RP11-111K18.1	TYROBP	IER2	COX7A2P2
CLIC4	PSMD14	NDUFS6	VAT1	BUD31	WDR7
SNTA1	PNMA1	ARL6IP1	CERS2	STAG2	OLFM1
FAM193A	ENC1	ATP5J	IGFBP4	RCN1	INPP5F
ANKRD36B	ECHS1	BCAP31	VWF	LSM14A	GABBR1
UNG	XRCC6	IER3	C1R	CD99	PPME1
RBMS1	NDUFA8	PPIB	HLA-DMB	DECR1	MYCBP2
PLCG1	TUBA4A	NEDD8	ARL6IP5	CDK5RAP3	APBA2
RIN2	CISD1	APEX1	MYD88	CNN3	PKP4P1
ABL1	MAT2B	RANP1	LAPTM5	PDIA6	EXTL2
TMBIM1	SPCS1	AL592188.3	AC104698.1	CDK4	SYBU
ERBB2IP	MYL6B	RP11-680H20.1	ANXA4	OXA1L	EFR3A
DUSP1	EDF1	MMADHC	RARRES2	VCL	RNF44
PTP4A2	AARS	CCT3	LAP3	TH1L	NPDC1
SPN	NHP2	CSNK2B	EIF1B	KIAA0907	MAPRE2
UBN1	VPS51	TCEA1	ARHGDIB	EIF2B1	PRPF19
NR2F1	MAP2K1	MAGED1	MRC1L1	ZC3H4	ATP1B2
TXNIP	CCNI	COPB1	PTGDS	MANF	CLASP1
PLCD1	KIAA0528	OAT	CTSL1	RIN2	TCEAL1
RP11-738E22.2	CCT4	TBCAP1	HLA-G	PLOD3	OXR1
LPAR1	GPRASP1	EIF4A3	EMP2	RND3	KIAA1279
PODXL	ST3GAL5	PSMB3	CAV1	GPN1	SH3BP5
SLC3A2	BCAP31	PSMC1P1	CTGF	SHC1	COPS7A
HMGB1P4	ACTR10	PSMA4	TSPO	PLTP	ACYP2
DAAM2	UQCRC1	DEK	CCND3	SNRPD1	HSPA2
RBM6	LRRC47	FBL	SQRDL	HSD17B10	NECAP1
FNBP1	RP11-526J3.3	IGHM	AC073869.1	LPCAT1	THY1
ERCC5	PTDSS1	NDUFA13	ATP6AP1	PRKRIR	ARPP19
TWF1	MEA1	COX7A2L	LMBRD1	NGLY1	KLHDC3
USP47	TSPYL2	UQCRC1	APOC1P1	MRC2	PSD3
XKR8	ATP6V1H	PSMD2	SRSF6	ECI2	KIAA1598
ZNF384	FXYD6	PSMB4	C4BPA	DNMT1	DDHD2
KANK1	NEDD8	HNRNPAB	PRPF8	RRAGD	RP5-1187M17.10
CHD8	RP11-38L15.6	MRPL33	CRIP1	FKBP9L	OMG
RGL2	WRB	IMPDH2	SFTPA2	HMGN4	CORO2B
KIAA0195	AL139385.1	COX5B	PPP4R1	OS9	DNM1
BCAS1	NDEL1	PRDX4	RFTN1	ID3	DENND4B
CCNG1	GALNT11	RP11-514O12.4	ILK	PSRC1	MKRN1
PSAT1	PSMA6P1	IGLV2-14	RRAGA	NCOA6	PI4KAP2
NBPF12	EIF3CL	GUSB	VPS51	PCNA	CAP2
FAT1	UBL5	PSMB6	REEP5	SERPINA3	B3GAT1
FASTK	CAP2	RP5-1053E7.1	RIN2	RP11-386M24.4	SNAP25
MSN	YPEL5	RP11-977G19.10	SARS	SNRPB	SKP1
PNRC1	RUNDC3A	COL3A1	ALDH9A1	ACTA2	RP11-94I2.1
CRYBG3	SEPW1	RP11-365O16.1	ALOX5AP	GPR137B	LDOC1
KCTD12	RBFOX1	NDUFB5	TUBB6	CMTM6	NCALD

MYH9	PRPF19	PSMA1	HSD17B11	PELI1	F8A2
IFITM1	TUBBP2	SEC31A	SLC35E2B	PDIA3	MDH1
PHLPP1	MORF4L1P1	BCL2L2-PABPN1	GPRC5A	BBX	ARF5
APOLD1	NPTX1	TOP1	TIMP2	CCDC47	TEX2
FBXW4	PHYHIP	RP4-545K15.3	PAPSS1	HLA-DRA	ARFIP2
ANXA5	IARS	DDIT4	ACADVL	SLC35B1	WBP2
SLC12A7	NOMO1	EIF4BP6	GADD45B	CHI3L1	RP11-287D1.3
COL16A1	VBP1	TMED9	RAB1B	RAB9A	MICU1
APOC1P1	TM2D3	TMEM123	FCGRT	NXF1	GPD1L
TMEM106B	BZW1	HBXIP	ID1	HADHA	AAK1
F3	UFC1	NHP2	APP	LAMP2	PCSK1N
CTDSP1	SYBU	PRICKLE4	AKR1B1	GUSB	CTNNA2
S100A10	SNRPD2	RP11-38L15.6	CD14	PPP4R1	TULP4
HIPK2	PSMB5	TUBB3	ETF1	AQP1	TAGLN3
KIAA0355	NDRG4	DHX15	OSBPL9	ALG5	SPOCK2
DNAJC1	HARS	NPM1	MT1F	GBE1	KIF3B
AC009469.1	MDH1	SERPINF1	NEK7	DHX40	AP3M2
CLDN5	SDHA	IGKV1-13	BTBD1	RUFY1	GPRASP1
PELI1	ATP5C1	PLP2	HSD17B4	MTHFD1	ARL2
CERS2	MRPS35	EDF1	ATP6V1B2	POLR2H	IQSEC1
KLF15	PSMC5	ECHS1	CTSD	RHOC	RNF220
LZTR1	CCT7	NDUFA3	VPS28	EPS8	ACTR1B
LHPP	AP3M2	CCT7	SRSF4	CTD-2643K12.1	AMPD2
GNA12	DCTN3	ATP6V0B	YWHAB	OSBPL9	GRIA2
SAFB2	ASNS	RP11-518D3.3	CRISPLD2	ABL1	FAM134B
KIF1B	DDX1	RBX1	SERPINB6	ANXA1	DPP6
KCNJ10	DYNC1I1	UBA1	AC007842.1	CKAP4	RAB6B
JUNB	NDUFA13	SRSF9	FIS1	YIF1A	MBP
YBX1P10	GRAMD1B	GPI	SERINC3	TRAM1	USP14
GS1-111G14.1	UQCRFS1P1	EIF3I	PSMB9	LONP1	FN3KRP
RP4-781L3.1	APLP1	ZMPSTE24	CECR1	MAPRE1	ATP6V0D1
NME4	CRYM	IGKV3-20	Z82188.1	JUN	NDN
TULP3	RP1-228P16.7	RNPS1P1	FOLR1	POLR2F	RGS5
NOP2	NDUFV1	AP2M1	DRAM1	LGALS3BP	TSPYL2
RHOBTB3	BTBD3	TMED10	MAFB	PODXL	CCDC85B
PLSCR4	SIRPA	UBL5	PFKFB3	APOC1	EEF1A2
CHSY1	CMC2	CAPZB	MGLL	SYPL1	MARK4
REV3L	ATP5G1	HTRA1	MYLK	RER1	STMN4
RAB11FIP3	NDUFB5	DDOST	HEBP1	FAT1	TSR2
MAP3K5	YWHAB	PDIA6	TOP2B	HLA-J	GLUD1
SUZ12P	PRPF8	HNRNPA3P5	KLF10	ADNP	PPP1R3C
POLR2M	RIC8A	IFNGR2	CCNG1	ID4	SLC6A1
CTDNEP1	COX7A2P2	UXT	BNIP3L	CRELD2	PIN1
P4HA1	SRSF6	SEC13	TIPARP	PLXNB2	AC034102.1
TST	PGBD5	SEPHS2	PI4KAP1	NUP153	RUSC1
SH2B2	MLF2	BAG6	ABLIM1	AIMP2	CDK5

CSK	DROSHA	APMAP	CD163	KIF1B	PIP4K2C
MID1IP1	HMP19	HSBP1	CYR61	NPM1	HAGH
MGAT1	PPME1	MZT2B	UBE2L6	UTP6	OPTN
NEK7	AP2M1	NDUFB11	CAPG	RAB8A	KBTBD11
LBR	SEPHS2	TMX2	BLVRB	SH3GLB1	KIAA0513
RP11-793H13.8	NDN	RP4-595K12.1	CDKN1A	CRY1	PIP5K1C
ERN2	SLC25A6	MRPL3	TAF7	KIAA0247	PQLC1
AC005013.1	SLIRP	EPCAM	EPHX1	PLSCR4	ARHGEF9
CRIM1	CRMP1	RABAC1	LITAF	CPSF4	RAP1GAP
FXR1	SLC22A17	RP1-228P16.7	MXI1	NOL11	CELSR2
COL5A3	STK39	SNRPD3	FKBP9L	APOC1P1	LETMD1
PRODH	MKRN1	HMGB1P5	SRRM1	ID1	ASRGL1
TMEM43	CS	NDUFB6	OS9	MPV17	NRGN
GPR56	AC009948.7	PSMC5	HECA	AC026271.5	DCAF6
DAZAP1	OAZ1	WDR45L	KIAA0247	ISG15	NELF
GMEB2	GGCT	IDH1	GDI1	CDC25B	CAMK2N1
CRY1	NDUFAB1	TMEM147	CHMP3	AHCY	MCF2L
WWC3	SCN2A	HSPE1	PHF11	POP5	ITPK1
CIC	ENOPH1	COPB2	SFTPD	IVNS1ABP	GOT1
USP16	NDUFB6	CIB1	ACP5	CIB1	SERPINI1
SLC9A3R1	PSMB6	GUK1	FBP1	AUP1	DNAJB2
PAK4	RP11-262M14.2	CS	PNRC1	SLC3A2	CXCL14
ZRSR2	IQCJ-SCHIP1	SF3B5	GNAI2	LITAF	SLC22A17
KDM2A	SULT4A1	UBE2L5P	MSL1	DDX39A	PRKAR2B
ZCCHC8	MTMR9	SDHA	CTDSP1	PTTG1	TUBA4A
STX10	PGRMC1	ITGAV	MYL9	HLA-DRB6	ATP2B1
WDTC1	ANKRD46	CTD-2643K12.1	PKIG	CLIC4	ARPC5L
NACC2	SERINC3	RP4-604K5.1	IDI1	ATP6V0E1	RNF11
RHOG	P4HTM	PSMD8	VSIG4	FAM35A	ATP6V1G2
ZNF573	MAGEH1	RP11-93O14.2	EMP3	CHSY1	NDRG2
GLTSCR1	RP11-193F5.4	SHFM1	NBL1	KDELR2	FAM216A
SCRIB	TUBB	MARCKSL1	ZNF611	RPS2P46	RCAN2
CHD4	SH3GL3	IGKV1OR2-118	PLIN2	FAM208A	REEP1
NFASC	RP11-697E2.6	SNX6	RP11-347C12.2	SNX6	PCDH9
SERTAD2	PFDN2	VMP1	MSMO1	GALNT1	KIF3A
GPRC5B	ATP5G3	HNRNPA1P3	TMBIM1	POLD2	MAST3
KCNJ2	GABARAPL2	RP11-21J18.1	TMEM109	TRIM22	EFHD2
ANKZF1	GUK1	PDCD10	PLBD1	KEAP1	DKK3
PLSCR3	KARS	S100A13	DECR1	RP11-142L4.3	HOMER1
PHF2	ETF1	SND1	SELENBP1	ACAA1	PDP1
CDKN1A	NCALD	SDC1	TOB1	ABCF1	BAI3
TOB2	AKR1A1	AC016745.2	SH3BGRL	JAM2	CA11
TMEM80	SARS	NDUFV1	MPC1	TSEN34	FBXW7
AC092839.1	RP11-977G19.10	TRAM1	TXNIP	VEZF1	RAB4B
CSRP2	RCAN2	MANF	RARRES3	RNASET2	RP4-788L13.1
METRN	RP5-977B1.10	ARF4	ZDHHC7	PSMC4	USP6

CROCCP2	SNRPB2	BASP1	IER5	SF3B3	ACTR1A
ZC3H7B	GNAS	PSMB5	MKNK2	UNC119B	GABRB1
TMEM47	FAIM2	RP11-386M24.4	TNFRSF1A	SNF8	CAMTA2
HDAC1	CBX7	FAM96B	SCPEP1	HDAC1	ATG13
SLCO4A1	ZNF706	HDAC1	DYNLT3	LGALS3	SLC25A11
SLCO3A1	TSG101	RP11-721G13.1	FOXO3	IFNGR1	CTSF
MUM1	CAMK2B	NDUFS2	SFTPC	ANXA2P2	RIMS3
FAM193B	NDUFA6	LRRC59	CD74	GRN	ANXA7
CNOT6	RUSC1	GARS	AC011498.1	HSD17B11	ADSS
DIP2A	PUF60	COL1A2	CXCL2	CTR9	STMN2
MBD3	SCAMP5	PSMD14	NXN	RBM3	SMARCA2
PLEKHA5	SCN3B	SNRPC	CFD	RPS27L	FNDC4
MFHAS1	ZNF365	POLR2B	NUPR1	CHCHD8	EHD3
CSDA	GS1-257G1.1	BZW2	RTN3	PNN	CLTB
MAN2A2	MRPL49	SNRPB	FAM8A1	HDAC3	DHCR24
UNC45A	TMEM14A	VDAC1	PARP4P2	VEZF1P1	DAAM2
AC008731.1	PSD3	RCN1	CES1P1	CAV1	SEC31B
CYP27A1	CETN2	SNRPB2	POLR1D	DNAJC2	PNPLA6
VWF	UBP1	HSP90AB1	KANK2	P4HA1	ADAM22
PECR	MAPRE2	NDUFB3	DDX5	NME4	PAK3
SERPINA3	TRAP1	ARCN1	DCTN3	TMEM248	UQCR10
AMT	FIG4	TXNRD1	ARHGEF18	SNRPF	MPHOSPH8
TMEM123	SNRPN	CLSTN1	HNRNPH2	DDAH2	DOCK9
CEP164	RAN	ISG15	SPAG7	KANSL2	PAIP1
MRC2	EMC7	CNIH	EDEM1	CPNE3	PRKCZ
NFAT5	GNG3	ARPC1A	FAM108A7P	TAGLN2	EFNB3
ZNF324	PDE2A	ATP6AP2	SH3GLB1	VKORC1	SCAMP5
KIF13B	PHB2	RP11-59H1.3	CDKN1B	GNL3	PLEKHM2
KDM4B	POLR2B	PPP1CA	FCER1G	ABCE1	DEAF1
SOX2	RP13-514E23.1	NOMO1	FLNA	FJX1	MYO18A
EFEMP2	AASDHPPT	PTDSS1	CCL18	PPP4C	PLLP
PAIP2B	GSTA4	GS1-257G1.1	CD52	RP11-632F7.1	NAPA
PXDC1	GPI	IARS	LYZ	RAF1	NDUFAF4
HLA-E	CSNK2B	XRCC5	WBP1L	SNX7	DNM3
UBXN7	RAB15	TBCB	SIK1	KLF10	ABHD14A
NINJ1	ARL3	DRG1	FBXO7	TMBIM1	IDH3A
IER3	APEX1	PRDX2	MGAT1	CTC-425F1.4	DEXI
SLC7A2	SNRNP25	GGCT	TRIM44	RP11-15K19.2	BIN1
MYBPC1	BNIP3L	GANAB	CASC3	NUP54	HMOX2
ZNF592	NDUFB11	YTHDF2	LEPROT	SUCO	KIAA1467
MXD4	FBXW7	PUF60	RAB8A	RP11-310H4.5	FBXW4
MIIP	SIPA1L1	IARS2	CD151	LYPLA1	RP11-229E13.2
SOX10	SSR4	MEA1	PPP3CA	TMCO1	IPO13
RHOB	SNX10	PDCD6	ARHGAP29	HILPDA	KIF3C
N4BP1	OPTN	RDBP	IK	SCAMP3	ELMO1
SIRT1	HPRT1	MZT2A	RP11-613H2.2	NMB	LDB2

BCL6	ISCA1	ILF2	PEF1	NECAP2	PTPRN2
CCDC90A	GRM3	YTHDF1	TERF2IP	RP4-781L3.1	LIMCH1
FAM63A	SLC25A4	KRT8P3	CD302	RNF114	ANK3
GRAMD3	MAGEF1	DPM1	RP11-802E16.3	GLB1	CYP46A1
ZNF423	CELSR2	TRIP12	HCLS1	HAT1	MPP1
RPS21	HAGH	CETN2	DENND5A	ANKRD10	HCFC1R1
ARHGEF1	NRN1	CRELD2	FXYD5	EXOSC10	TARBP1
PPP1R14BP3	PFDN1	CDK4	ETS2	AEBP1	WAC
TJAP1	DDB1	HEBP2	GOLGA8IP	CECR5	DTX4
CRLF3	ATP6AP1	AP2S1	RHOG	PGLS	CBX7
RP11-561C5.4	MADD	COPZ1	IFNGR1	ADSL	MRPL48
HLA-G	RP4-788L13.1	DDX1	WARS	GMNN	REEP2
TNRC6B	ARL6IP5	UBA2	GSTA4	TPRKB	NBL1
RGS19	APBA2	SUMO4	NXF1	RACGAP1	MADD
GOLGA8IP	ARHGEF3	GHITM	FAM32A	NUP205	ARHGAP5
ID3	RGS2	VBP1	ADIPOR2	TPST1	sept-04
EFNA1	GPD1L	BANF1P3	GPR116	S100A11	CYFIP2
BBX	SC5DL	OXA1L	RBM23	GAR1	AGTPBP1
RP11-98J23.2	DNAJA1	COPS6	AC012379.1	INTS8	FAIM2
INPPL1	GAPDHP1	MRPL49	ID3	SLC2A1	CRYM
KAT6B	REEP1	HN1L	BRD2	RGL2	CNTNAP1
ZBTB38	BEX4	PLOD3	NFIL3	NFIL3	PLEKHA1
HLA-J	NDUFS2	ATOX1	ATF3	CDKN1A	NBEA
EMX2	PI4KAP2	RP4-614O4.11	ESYT1	NAMPT	MAPK3
ACACB	LCMT1	FAM134C	PCNP	RP11-468E2.2	RAB11FIP2
THBS2	HSPA12A	AC019178.1	DMBT1	ITGB1	TOMM34
DHRS3	CALM3	MRPL18	SIDT2	USP1	GABARAPL1
ENGASE	CNTNAP1	ALG5	SNX17	THOC1	AACS
TBC1D17	TSC1	SLC12A7	STX12	DYNLT1	LARP1
TNIP2	LDB2	AMZ2	PALM2-AKAP2	LAPTM5	UROS
CDC42BPA	HBXIP	NDUFA6	TRAPPC8	LBR	DGKZP1
ZNF444	IGBP1	GORASP2	ZC3H4	SH3BP4	SLX1B-SULT1A4
PLOD1	SSR2	DDX21	MID1IP1	MTCP1NB	CLASP2
KIF5B	RP11-305B6.1	TMEM70	CCDC92	ST5	LARP6
TPP1	MRPS22	F8A2	IL7R	VMP1	GNAZ
QSER1	CTSA	GS1-124K5.12	GPD1L	GFAP	PALM
PTPN1	RP11-365O16.1	HSD17B10	SLC43A3	ABCA1	TCP1
RRP1B	PGP	PFN2	MYO1C	BTG3	AMPH
NFIC	CUEDC2	METTL9	ZYX	REV3L	ROGDI
MAP3K11	RFK	ADIPOR1	MYC	NAT10	SYNGR1
ZNF358	COX4I1	AHSA1	MAN2B1	MTX1P1	NPTX1
ARHGEF10L	KLHL12	PFDN2	RND3	DVL3	NISCH
FAM189A2	TSR2	POLR2H	RGL2	RBP1	TUBA3E
TAGLN2	AKR1B1	ORMDL2	SETD1B	DARS	EPHA4
ARPC1B	PJA1	TMEM14D	UBP1	NUP107	HABP4
RP11-74E24.2	IMMT	GOT2	DUSP6	CYBA	MAPK8IP3

BCL2	MICU1	TRIM28	DCTN1	C1QB	TUB
RCBTB1	URGCP	CACYBP	SEC16A	ADM	RUNDC3A
MAP7	UBA1	MRPS35	IL32	ALG8	DIRAS2
LDB3	SNAP25	NCKAP1	SHOC2	IP6K2	BAI2
PALLD	ZC3H15	RIC8A	FDX1P1	DUSP12	KCTD2
APOC1	ATP6V1G2	NME2	DAZAP2	ZHX2	ZNF365
ZFP36L1	OXCT1	RER1	WTAP	TAX1BP3	KLC1
FOXO1	EIF4A3	NDUFA8	SLTM	CALCOCO2	SFXN3
MED13L	CREG1	PIGP	RNF13	HEBP2	CLDN11
STAG2	SLC25A44	GNPAT	DHRS3	ARPC1B	NCS1
ADM	EIF3I	COX17P1	ICAM2	PXDC1	RAPGEF5
TENC1	FAM82A2	HYOU1	KAL1	ALDH3A2	PGBD5
PLEKHO2	TTC19	DDB1	COL6A2	CTGF	MGEA5
MUTYH	KIF3C	RP5-1042K10.13	AK1	TMED7	UPF2
DDAH2	NRD1	TMEM176A	CDC37	NAV2	GABBR2
HYAL2	RANBP6	RNF4	CRBN	TGFBI	NPTXR
PDK4	SKP1	AARS	GYG1	PIGT	HSPBP1
FOXC1	PARL	PSMB8	LMCD1	CTSO	CIC
DIAPH1	BACE1	DIABLO	ST3GAL5	WLS	CDK5R1
TRIM26	TMX2	TMEM176B	RGL1	THBS2	HERC1
ASAP3	TXN	H1F0	FMOD	CTDSP1	MAPK9
IL17RB	CA11	YIF1A	LCP1	KIAA0355	MAL
SELENBP1	NPDC1	CTSK	TMEM50A	SCRIB	DYNC1I1
MAP3K3	SEC31B	IGKV1-37	MYO5C	MSRB1	VAMP2
IRF3	PIP4K2C	RP11-298C3.2	AC009469.1	AC009469.1	GNAI1
HIST1H2AC	FAM32A	VKORC1	GLRX	CREB3L2	SH3GL2
LHFPL2	ABCA3	IGKV1-17	USP11	PRCP	GRAMD1B
FCGRT	IK	YWHAZ	FBLN1	RCAN1	NIPSNAP1
SORBS3	ASH2L	HMGB2	NDFIP1	TDG	SPATA2
CPNE3	CALM1	TMEM248	YWHAH	FUCA1	CX3CL1
GTF2IRD1	RBX1	AL021977.1	CAV2	EIF2D	RNF144A
HEG1	H2AFZ	MAPK6	DCTN6	ARHGDIB	CLCN6
DAP3	SNRPEP4	SERPINH1	SLX1B-SULT1A4	CLK2	LY6E
SERPING1	DNAJA3	VDAC3	AC007347.1	SAP30BP	PRDM2
AD000090.2	NME2	IGBP1	TMEM41B	RNF138	SHC3
FN1	TMEM147	TUBB2A	FOSB	PPP1R14BP3	SLC17A7
S100A1	RABAC1	MAGED2	KCTD12	SERTAD2	MLST8
AXIN1	SV2B	GDI2	UNC13B	SERPING1	SLC48A1
AHCTF1	NOP10	P4HA1	CYP2B6	DAP3	PHLPP2
ITPKB	SHOC2	ENO1	MRFAP1L1	DERL2	MEF2C
RHOC	MRPL3	MSRB1	NDEL1	RP11-173D9.3	TPPP
KANK2	METTL9	COX7C	SYS1-DBNDD2	DERA	SNTA1
UBE2Z	SS18L2	CTC-554D6.1	JUN	ASUN	UBB
E2F4	COMMD3	HDGF	RAB20	FDX1P1	RUSC2
KCNN3	POP7	ATIC	FAM82A2	PLIN3	HMP19
UNC5B	ARPC2	UFD1L	CHSY1	SEC61A1	RAB15

MPST	EEF1A2	MTCH2	PPL	DHX29	PLK2
ANTXR1	NDUFA9	RNPEP	FAM21A	FNBP1L	RAP1GAP2
PRKAG1	VPS45	AUP1	GTF2B	EMP3	KIAA0284
TMEM176A	PSMB1	NDUFA9	ADD1	SRPX	BTBD2
PCF11	FAM192A	MLF2	VPS4B	TRIP6	SH3GL3
ARID3B	ERP29	COX7A2P2	MAP7D1	SNRPA	FAM20B
RXRA	AACS	NANS	WDR13	HEG1	OPCML
ROBO3	NAE1	FAM50A	RAB11FIP1	RBM42	TPPP3
TNFRSF1A	KIF3B	IFT20	ETF1P1	ADNP2	ANK2
MLC1	CYC1	FKBP2	AC008731.1	LEPROT	HSPA12A
TIMP1	DCTPP1	VOPP1	LTBP2	YES1	MDN1
HEPH	FIS1	NAE1	RP11-87C12.5	WDR12	ENPP2
RBL2	NRIP3	AFTPH	MLH1	PKD2	NCDN
DAPP1	PCBP1	DUS1L	RGS5	C1QA	PRKAR1B
DDX11	PRKAR2B	HSPA1B	EFHA1	IGFBP2	KCNK1
ECHDC2	DIABLO	JUP	MINK1	BCL10	SH3BP1
ANXA2	PYGB	RP11-468E2.2	GEM	CETN3	SORBS2
KLF6	MAP7D1	DDR1	VEZF1	ARFGAP3	ENTPD6
IQCA1	AMPH	TMED3	NR4A1	LMAN2L	RP13-608F4.6
HLA-DMB	GNAZ	MPV17	TTC19	CCNL2	CEND1
NOTCH1	TCEAL1	LSM1	NACAP1	UNG	FOCAD
ZNF692	CCK	CYC1	MFAP4	RBM22	PDE2A
SPR	IQSEC1	RNF44	CCDC28A	CCDC86	ACTR3B
NFE2L2	TTC1	AC063976.6	ALAS1	TAP1	COX7A1
AC135048.13	DPP6	SMYD2	COQ10B	IGFBP3	KIF21B
NECAP2	KIAA0319L	KDELR2	FEZ2	LHFPL2	APBB1
TAP1	SPAG7	TSEN34	CTNNAL1	ZNF266	LHPP
GFPT2	CKMT1B	CHST15	AKAP11	GNA12	CHST1
RAB3IL1	PIN1	LAMP2	CTDNEP1	HMGXB4	NKRF
NIPBL	BAI3	LBR	HERC2P2	CSDA	PRKCE
BMPR1B	NAGPA	VPS52	CCL2	HMGCL	LANCL1
CSF1R	NAP1L2	ITPA	TGFB1I1	OXSR1	RBFOX2
SDHAP2	TSPAN5	ZC3H15	RAN	HIST1H2AC	PTGES2
IGLL3P	SMYD2	PGD	ANXA6	MIS12	GNG3
HAPLN2	ADO	EI24	SUN2	POLR2M	EPB49
SCAMP2	PITRM1	RP11-446E9.1	CNN3	FTSJ2	ATP6V0A1
CHI3L1	SACS	RUVBL2	NDNF	LAMC1	FXR2
VEZF1P1	CORO2B	PIH1D1	PARP12	ZZZ3	ASPHD1
CNNM3	NGLY1	TPD52L2	SERPINB1	MYD88	TUBB4A
CACFD1	HABP4	FKBP3	TNFAIP1	TYROBP	AGXT2L1
AEBP1	HSBP1	GPN1	CPSF7	ETF1P1	BTRC
EP300	VPS28	PARL	AOC3	TRAF3IP2	TOM1L2
NXN	CRBN	RBCK1	TM2D3	TEX261	CACNA2D1
SPEN	EPB49	DCTPP1	BTG2	TNFRSF1A	HIP1R
CIT	PSMD7	TCEB2	SYNPO	XPC	NPTX2
SLCO2B1	ATP5D	PUM2	CPA3	FCGRT	TLN2

ZNF395	CRELD2	MXRA5	AL162497.1	SSRP1	PSMD11
TEX261	PRICKLE4	CTSB	AL139385.1	APIP	SNAP91
CARHSP1	ARCN1	NRD1	CYP27A1	DAP	INA
HN1L	CHMP3	ADRM1	QDPR	DYRK4	NNAT
LIMS2	FGF13	LSM7	TMEM9B	TMED3	KIAA1549L
RAD54L2	SUCLA2	PRPF19	PELI1	GMPSP1	TUBG2
LTBP1	MAEA	GADD45A	PIK3C2B	AIMP1	CALM1
DDX17	MDH2	MRPL9	AQP1	C1R	ME3
CBFA2T2	CTC-554D6.1	MEST	GAK	CD14	CHRDL1
AEN	CAMKV	SLC35B1	IL4R	SLC12A7	RALGPS1
SYPL1	RNF4	DAP	ARL2	DDX23	PEG3
EBLN2	SLC30A3	BUD31	CTC-479C5.12	RPL10	SYNGR3
CEACAM19	NDUFAF3	NDUFS3	MICU1	NINJ1	OTUB1
EML3	DDRGK1	HMGA1	FMO2	WWC3	TMEM183A
MYO1C	PARP1	COMMD3	ACSL1	DDX18	MAP2K4
IQGAP1	EXTL2	PARP1	DNAJB1	MRPS14	INSIG1
PBXIP1	DDN	UAP1	ABCA3	CBY1	SLC25A28
MAP4K4	NARF	BNIP3	PFDN1	NOTCH1	ADAP1
PTPRU	ATP5H	KRT10	SACM1L	MTHFD2	SREBF2
ICAM2	ATP6V0B	MRPS33	CD83	ECI1	PET112
NASP	CHGA	NDUFS4	PIEZO1	RALY	ATP13A2
TRIM25	PDHX	EIF2AK1	G0S2	TYW1B	NDRG3
UGDH	CHMP5	MRPL24	DTX4	EXOSC8	ACOT7
AC099668.5	UNC50	EIF2B1	CD97	UBR5	SLC25A22
RPS17P5	RP11-785H5.1	COPS5	PAPSS2	TGFBR2	TMEM59L
RNF122	COQ10B	HIST2H2AA3	MOAP1	PLOD2	ZBTB7A
DND1	CTSF	PPP1R8	CSTA	PPM1G	LAMP5
CTBP2	ETFB	NSF	AC115617.2	APPL2	STX1A
DVL2	EIF3D	VAMP7	ACAA1	PARP4P2	SCN2A
PRPF38B	SNX17	RPS17P5	TMEM30A	RARS	NIPAL3
RP11-57A19.3	CTNNBIP1	CMC2	HYAL2	RCBTB2	RP11-566E18.3
EMID1	RP11-20I23.1	NOL11	TIMP3	CTSD	GIT1
TCF7	GNPDA1	PCNA	ENG	ATP13A1	RAB11FIP5
EHD2	TPI1	CDC123	VPS4A	GLT8D1	RPS6KA2
CYBA	CAPNS1	C1QBP	CBX6	SDF4	GRM3
SLC22A5	LONP1	USP14	MAFF	VSIG4	SCN3B
PHLDB1	F8A2	AHCY	LEPROTL1	DKC1	MAP6D1
STARD3	RALYL	SEMA4C	TMEM204	TARS	LPHN1
STK11	SNCA	FAM60A	CD47	NXT1	FAM49A
SZRD1	EIF2S2P4	RP11-310H4.5	PJA2	WDR3	PISD
TRIM38	VPS11	IDH2	ABL1	ERCC1	TOLLIP
CDKN1C	EXOC1	BZW1P2	NISCH	AC008731.1	CELF2
EGFR	SHFM1	RAD23B	NCF2	LMAN2	AP1G2
NOP14	NECAP1	PLTP	TPCN1	PRKRIP1	TIAM1
MKNK1	ITGAE	ASS1	LHFP	COMMD4	ITPR1
PHF10	RANP1	TECRP1	RAB7A	PHC1P1	EGR3

RBCK1	PCP4	PPP4C	HADHA	INTS12	CAMK2G
POGZ	BAG6	POLR2I	GAS6	STX2	GNAQ
IQCK	PSMB3	AC009948.7	STXBP1	PLP2	LPPR2
AQP1	RP11-315C6.3	PGRMC1	SCGB1A1	RRP1B	CRY2
IFNAR2	IMPDH2	IMMT	DENND4B	AC019097.7	SH2B1
TFEB	RP5-1042K10.13	SC5DL	AC040173.1	DPM3	BAP1
EP400	FNDC4	PSMD7	SDR39U1	BBOX1	FBXO41
RELA	RCN2	GALNT1	SCAF8	TRIM24	TTLL7
CDK13	GAP43	IGKV2-28	EXOSC7	CD151	COX7B
HILPDA	TMEM70	RP11-317N8.2	WWC3	IFIT3	NRSN2
BCAT2	TRIP12	HSP90B1	RGS19	TCTN1	SULT4A1
THRA	PEX11B	CBX1	ANKS1A	ZBTB5	FGF13
CYB5R3	LGI1	SOX4	GMFG	FBXL12	EPB41L1
GAL3ST4	FOCAD	MRPL15	ADD3	BCHE	GNAO1
PIM1	SEC13	STRA13	MIR22HG	TRIB2	SEZ6L2
NUPR1	ABHD14A	GBAS	RAB9A	CKS1B	CHD3
RBM4B	TOMM20	ARFIP2	USP33	PSMD5	MT1P3
MTF1	COX7A1	FIBP	ITGA5	PPATP1	AATK
USP1	MRPL24	IVNS1ABP	SPOCK2	BFAR	NAP1L2
SELPLG	DPY30	POLR2K	PPAP2A	RBCK1	GSTM3
LINC00341	PRKRIR	ATP5G1	DTX2P1	FERMT2	ZC3H13
TGFBR3	BZW2	ARF5	PKD1P1	HLA-DMB	LGI1
BTG2	PIH1D1	ARPC5L	MEPCE	RFC4	CAMK1D
ZNF451	LYRM1	RAI14	PION	KRR1	CCP110
MAPKBP1	ACTR1B	LAPTM4B	SLC7A7	VCAN	ADCY1
SPTBN1	EIF2B1	PPP1R14BP3	LPXN	TOR1B	PPP2R5B
PTPN18	BRD9	EIF2D	GYPC	RNPEP	TBC1D9
ANXA2P2	FDPS	TUBA4A	ASNSD1	SCAMP2	ERC2
SMARCD2	HPCA	KXD1	GABARAPL3	NUP85	FAM131B
NOTCH2	SNRPD3	SCAMP3	CTSO	SEMA5A	TMEM160
ALDH4A1	GORASP2	UGDH	PRKCZ	PLOD1	KCNAB2
HIGD1B	PRAF2	PAICS	PEAK1	ZBTB33	DUSP7
MEIS3P2	RUVBL2	CTCF	SPRY1	COL4A1	NAGPA
CBFB	PSMD1	NGLY1	TMX4	MRPL42	MAN2A2
ZIC1	DHCR24	P4HB	CLTB	DHRS3	FBXO2
PDGFRB	GLRX2	DROSHA	PPAP2B	PSMB9	FSD1
ZNF446	SUB1	YY1AP1	CSF1R	GADD45B	AK5
PPFIBP2	UBXN6	GPAA1	HEG1	RASSF4	EPB41L3
KLF2	AFTPH	RNF5P1	EID1	GTF2IRD1	SCN1B
QPRT	WBP2	PPP2R1A	LHFPL2	VPS37B	CAMK2A
RUFY1	YTHDF2	DNAJA3	GBP1P1	KDM3A	ETS2
PHLDA3	PAPD7	PIGT	CSRP2	CRYBG3	NRXN2
LAMC1	ASNSD1	AAMP	SRSF7	CSK	SYNDIG1
ZHX2	RP3-352A20.1	GTF2H5	ARL2BP	CTNNBL1	PRRG1
C4A	NPTXR	PGLS	GIMAP5	ALOX5AP	ELAVL4
RPS6KA5	PDCD10	TTC3P1	LETMD1	ADI1	CHGB

RASSF4	CDK5	HPRT1	GAA	TMEM176B	NACAD
SFN	TBPL1	TRIAP1	MYLIP	PRPF38B	FAM5B
PER1	SCAP	THBS2	SASH1	MATN2	PRODH
FLT1	STX1A	PIP4K2C	TREM1	MPPE1	SPINT2
PODNL1	PCSK1N	PCOLCE	TPSB2	EFHC1	B4GALT2
SRRM2	POLB	THOC7	CAT	SUCLG2	SYN1
HNRNPUL1	RNF111	UTP18	UBXN6	CD302	SLC30A3
EIF4EBP2	XRCC5	MLEC	CHN1	DESI2	PIK3R2
TSPO	NAT10	EMC6	RPS6KA2	SPTSSA	RP11-526J3.3
ITGA6	GNB2	TH1L	SEC24B	NUP93	CCBL1
CREB3L2	GPN1	RP11-231C14.3	AES	CXCR7	BCAS1
MXRA8	CDO1	CISD1	ATP6V1H	FTSJ3	KLHL3
AFF1	GABARAPL1	SF3B1	PODXL	EFNA1	NME5
CD82	CTBP1	CCT6A	TNFSF10	GPSM2	RPH3A
ABCA8	ENSA	GOLGA5	PPP1CB	RARRES3	NRIP3
PLTP	MEF2C	DAP3	H3F3B	GLA	COX5A
MYD88	MRPL18	ETFB	BRD3	CNOT2	HPCAL1
GRIN2C	PPP2R1A	MTX1P1	RXRA	LRP10	NSG1
FDFT1	KIFAP3	SLC38A1	CALCOCO1	CD53	NECAB3
FCHSD2	CNIH	CSNK1A1	CTR9	WWTR1	SULT1A2
UCP2	SLC17A7	CLDN7	RRAS	GATAD2A	CLDN10
NEIL1	CDC123	RPA3	AXL	TCTN3	PNMA2
TGS1	GRHPR	TMEM214	SLK	XPO1	SLC25A12
IGKJ5	RDBP	PSMC4	ANXA3	AP1S2	FHL2
NAV2	ASTN1	ABCF1	GNAQ	CECR1	RBFOX1
VSIG4	SH3BP5	RPL26L1	NDUFAF3	CTNNAL1	VSNL1
CTDSPL	BABAM1	MORF4	NES	TAF2	ERN2
GDPD2	REEP2	ACTN1	MPPE1	LPL	PACSIN1
FOLR2	RASGRP1	RALY	GNG11	PBX3	SLC25A23
LAMA5	FN3KRP	MAGEF1	TBC1D9	RGS19	HPCA
TTC38	RP11-21J18.1	COPE	HNRPDL	UIMC1	KIT
PLIN3	PEF1	PSMD1	SH3BP5	CPNE1	CAMK2B
VEZF1	ANKMY2	SF3B4	MCCC1	PAF1	SEMA4D
SRPK3	KIF21B	POLR2F	COLEC12	MGP	SNPH
FOXO4	AGPAT1	AIMP2	ACTR1B	RALA	SEMA4A
RAPGEF3	AP2S1	PLSCR3	CTNNBIP1	MCAM	RALB
SSPN	DDX41	IMP4	ELF1	MCM7	SLC4A3
WSCD1	ARFIP2	NME4	CFH	TSPO	KCNB1
PARP4P2	UBR7	SLBP	DAPK1	HN1L	CAMK1
INO80D	LETMD1	CKS1B	F13A1	TMEM176A	PCNXL2
GNA13	RP11-446E9.1	COASY	PXDC1	EFEMP2	NR3C2
LRP6	COPS4	PDIA5	HBEGF	NOC3L	CD200
RPL22P22	CAND2	IFI6	SLCO2A1	SMAD4	PC
AC093734.1	SCN3A	LTBR	SLC9A6	NOL8	ZNF324
GSN	WDR61	CRYZ	GALNT11	DNAJC1	HIVEP2
MYLK	PLD3	SCNM1	CCL5	ZCCHC8	KIAA0930

IGF1R	BAALC	DDX41	NUAK1	NUCB2	DDN
TCF12	HEY1	RP1-40G4P.1	PIK3IP1	ZNF259	DYSF
MID1	BSN	SLIRP	GUCY1A3	NES	HDAC5
CAPN2	EFNB3	SLC3A2	PLAC8	COTL1	RASL10A
MTMR10	MRFAP1L1	BCL10	ARHGEF6	C1GALT1C1	DUSP26
GPSM3	PPP1R7	SCAND1	SCAMP2	PIGF	KCTD13
RARRES2	TMEM50A	AC026271.5	WBP2	ELK3	GAD1
RHBDD3	KIAA1107	POSTN	FBXW4	WDR73	DBC1
HSD17B14	DPM1	CTC-524C5.2	TPSAB1	GTF2H2B	SYT13
TINAGL1	COPS5	MFAP1	AKT1	FLNA	CYB561
ABHD3	COPS7A	IMP3	INPP1	TMEM43	USP5
GFAP	SYT1	ATP5H	ITGB2	NUPR1	CRTC1
ANGPTL4	NHP2L1	EMG1	CNOT8	G6PC3	FKBP8
APOC4	DUSP26	SPP1	PALLD	EXT2	DUSP8
DAG1	CD83	IMPA2	UBBP1	CCDC59	PAIP2B
NINL	NBL1	RPS2P46	CTDSPL	PSENEN	CHGA
EPS8	NBEA	GOLPH3L	KIAA0528	TIMP2	ZMIZ2
VASP	BCAS2	ZNHIT1	PDXK	TREM2	CTIF
CUX1	PSMD8	DHX32	SH2B3	UBE2G1	GSK3A
TJP2	OAZ2	KIAA0907	ZMIZ1	FCGBP	KLHL26
CD99	ATMIN	GSS	EPS15	RBBP8	CACNB3
PLCB3	SEC61G	UTP6	TMEM43	TTI1	NLK
NAA16	LAMP5	NOL7	LRRC32	ANXA4	FBXL2
RNPEPL1	APBB1	ADSL	HNRNPA2B1	PION	RANGAP1
RARRES3	DAZAP2	RP4-775C13.1	PRELP	MAGOH	TEF
MAFF	COMMD1	CCDC90A	SON	KRCC1	HUWE1
LEPRE1	NEFH	HN1	HCFC1R1	STYXL1	SLC12A5
MITF	AC007842.1	DNMT1	ISCA1	RP11-488C13.7	PCDH8
FLNA	TM9SF2	RAB25	H2AFV	MCM6	AVPI1
EZR	PSMA1	CCDC47	LTBP3	SGPL1	RAPGEF4
S100A11P1	OPCML	SF3B3	CYB5R3	INPPL1	TSPYL5
HK2	PSMB4	IGFBP2	PPP2R5A	CCDC109B	NEDD4L
MYO10	AC009052.12	IMPA1	TCEB3	PLAT	UBE2O
BAZ2A	LAGE3	ENOPH1	IFIT3	TULP3	RNF123
SMARCC1	SCAMP3	POP5	CLDN5	TNIP2	RP13-514E23.1
FGFR2	TMX4	RP3-352A20.1	NFKB1	GNS	PPP1R16B
LRRC32	HPCAL4	IGLV3-10	KANK1	PMF1	ATP8A1
XRCC2	CTNNA2	NARF	NDN	E2F3	PCP4
SLC39A1	COMMD9	SMS	FLII	UCP2	PGAP3
CUEDC1	NUDT11	TMEM208	PDLIM2	PLEKHO2	GPR153
NMB	DCTN6	IGKV4-1	TPST2	TUBB6	NOS1AP
PARD3	NIF3L1	VPS72	TGOLN2	GSTK1	TBC1D17
FOXN3	CCNH	COA3	AVPI1	RMI1	FAM13B
CEP350	IDH3B	SS18L2	FARP1	DPYSL3	SNCB
ID4	PNMA3	SNF8	DOCK4	TMEM251	PHYHIP
XAF1	MSL1	NOSIP	CES2	KCTD3	S100A1

PLXNB1	NUDT9	YIPF3	CCNL2	TOB2	TMOD2
LRP10	TRIAP1	TMEM14A	ZSCAN18	COMT	GNB5
DCHS1	UBE2L5P	PDIA4	PLEKHO2	FEM1C	CADPS2
STK17B	PDHB	PLIN3	ACO1	MAFF	ANXA11
SLC35E2	CHST10	SCFD1	INTS3	PAICSP4	SOX10
ZNF609	USP14	BRD9	FAM208A	DENND4C	PTPRF
TAZ	ARMCX1	MCTS1	FASN	PLXND1	GUCY1B3
SLC1A5	VAMP7	PFKP	ALDH1A1	GYS1	RPS6KA4
ATN1	MMADHC	NUP153	NUDT9	SEC23B	ARHGAP33
ANKRD40	PLEKHJ1	SNRPF	SNX10	LPAR6	KCNS1
MYO9B	FAM136A	GMPSP1	PDCD6IP	AAR2	CAMKV
MAP2K3	FBXL2	RP11-651P23.4	AMOTL2	RUVBL1	CCK
PLCG2	FSD1	PSMD3	PINK1	RPS21	GAS6
NPAS3	ATP6AP2	PAPD7	MRC2	TSPAN6	HPCAL4
TCEB3	ROGDI	BANP	IRF8	MCM3	BAIAP2
REEP4	THOC7	RBBP4	RAB32	ORMDL2	HDAC11
CENPB	FAM131B	EIF2S1	YY1	DCAF13	SEZ6L
LSM12P1	FAM216A	AC009052.12	AL118508.1	PUS7	C2CD2L
SKI	AC012379.1	HDAC3	STX7	ALG6	GSTM5
OTUD7B	DGKZP1	TWF1	USP16	C4A	SUPV3L1
LILRB3	MAGED2	MRPL16	SLC15A3	SSR1	DPF1
PLIN2	OPA1	PTTG1	MARCO	MAGT1	MPP2
GSDMD	SF3B3	POLR2G	EGFL6	ZNF395	GNAS
ELL2	ACTN1	DDX39A	GSTM2	ITGB3BP	RP11-382A20.3
TMEM104	ATOX1	CKAP4	PYCARD	ABHD3	DOCK3
FBXL7	CDC34	ACP2	SLC39A8	MID1	INPP4A
KAT8	CLCN6	SNRPA	TBK1	DTNA	SSX2IP
MTUS1	ARMCX2	TRAP1	CTSS	PDIA4	NECAB2
ZNF768	AK5	TRIP6	ENPP2	RFXANK	AP2A2
AC005943.5	OXA1L	UBE2A	PPM1F	SMARCA5	NET1
SLA	SLC25A22	RCN2	LPL	AC100791.1	PPP1R12B
SETP14	MRPS27	PWP1	EFEMP2	ZNF274	ULK1
ZNF652	MRPL28	MTHFD1	LYRM1	UGDH	RPS6KA5
SURF1	GTF2B	NIT2	PQLC1	CHFR	L1CAM
PIEZO1	TRAPPC12	ACOT13	FBLN5	CNOT6	CELSR3
PRKAR2A	AAMP	ICMT	DMTF1	PXDN	KCNJ4
LIFR	RP11-317N8.2	NUP107	SNX2	NOP2	ACSBG1
SMO	SF3B5	RP11-632F7.1	CRIP2	HIST2H2AA3	THRA
PGF	TMEM14B	PLAT	CALCOCO2	ZNF337	SNCA
GOLIM4	NANS	POP7	LY86	EMC8	TACC2
TRIP6	MPPE1	TRAPPC3	CUL1	ENAH	sept-05
PKD1P6	ESYT1	EAPP	FIG4	HCLS1	RAB33A
sept-02	ERC2	CXCL9	PIP5K1C	ZWINT	RAB3A
CCNT1	SYN1	MRPL40	GRK5	UFL1	GAMT
PARP16	CDK5RAP1	MRPS27	ZCCHC24	HK2	FAM50B
PLEKHF1	MRPS33	INTS8	LMO2	RGS10	SYT1

MVP	LPHN3	NEU1	UXS1	FADD	CAB39L
ITGB5	TOMM34	RGS10	HLA-DRB6	ITGB5	ABLIM3
ARHGDIB	ITPA	SUPT5H	PPP6R3	PLIN2	EXOC6B
ZNF500	VSNL1	RUSC1	HELZ	ZNF83	PARD6A
CFLAR	PTS	SCO2	PROS1	CASP3	SV2B
SERBP1	SEZ6L2	IDH3G	ACTG2	GNG12	ABR
CRISPLD2	CRY2	METAP1	ZNF266	LY86	ATP6V1C1
FLCN	HYOU1	XKR8	SULT1A2	TRIT1	PTPRD
WDR55	KCTD2	PTOV1	CCNH	PRRC1	NEFM
EIF2C3	RPS6KC1	LYPLA1	NAMPT	GFPT2	RAB40C
IL4R	CTC-524C5.2	COPS3	CCDC85B	BST2	RASSF7
CNDP2	TXNRD1	DDIT3	PTPN1	PHC2	RHOBTB2
RGPD5	GOT1	LMAN2	MAOB	POR	ARHGEF4
USP3	POLR2C	PERP	PEA15	IFI44L	SATB1
CLNS1A	FIBP	ITM2C	ST5	HNRNPF	VAMP1
PRELP	CDH18	AP000275.65	NRP1	CHIC2	UGT8
GAS1	ACYP2	CHCHD8	IRF1	RRM1	UNC13A
ENG	CCBL2	DGCR6	MAP3K11	ITGA6	FAM13C
RP11-727A23.4	ATG9A	CAPN1	LDB2	ADAM9	COA3
PTPN2	BEGAIN	DSP	DUSP22	ZNF211	DMXL2
PDE4DIP	CADPS2	NXT1	B3GNT1	LTBP1	AF011889.5
LONRF3	RP11-680H20.1	ZNF384	ADH5	TMED1	CRELD1
sept-10	NDUFAF1	ALG8	KIAA0182	WDR77	TTBK2
IGF2BP2	SS18L1	AZIN1	COMMD9	TRIP4	THRB
SLC5A3	PDHA1	GNPDA1	LIMCH1	CDKN2C	SBF1
JUP	BRE	B4GALT3	VASP	TSR3	CDK20
CYTH1	PSMA2	EMC2	DGKD	WSCD1	MAPK8
IGFBP5	CYFIP2	NOP56	KIAA0355	KIAA1033	PPP6R2
PDE8A	GLOD4	SSB	VPS37B	FAM111A	BCAS3
FCGBP	PNMA2	RP11-700P18.1	ITM2A	EHD2	HLF
MSX1	CHMP7	FAT1	DYNC1LI2	TNFRSF12A	RALYL
IFIT2	PAM	CCBL2	FXYD6	BLMH	SPTBN1
ACADS	RAB11FIP2	BCAS2	MPST	CKS2	RUNDC3B
AKR1C3	AUH	CTC-435M10.3	LAMB2	CAPG	KCNF1
PHF3	MPP1	TNFRSF12A	SNRPN	ETHE1	EDIL3
ACP6	WTAP	LSR	OXSR1	EGR1	CPNE7
ARRB1	SMAP1	FAM136A	CCDC53	CNIH4	LRRN2
MAN2B1	PLEKHB2	MCM6	RP11-29G8.3	ACTR3	LMO4
INHBB	XPA	TSNAX	FAM53C	MCM2	MOBP
RORA	TAOK3	MRPS7	XPC	ISG20L2	KIAA0232
JMJD6	RAB26	SCRN1	COL4A2	PRC1	KCNA2
ITPKC	EFHD2	IFI16	KIT	POT1	LY6H
IL11RA	OGDHL	APRT	PNPLA6	NBPF1	ABCA2
BTN3A1	GS1-124K5.12	CCT2	MTMR14	PRPF3	MAP3K10
OSBPL11	RP4-614O4.11	MRPL17	ACTR1A	TBC1D2B	PIGZ
PCBP2	RAB11FIP5	THYN1	ECI2	SLA	MAP7

FGFR3	AGK	XPO6	MAGEH1	THBS4	ITGB1BP1
CPT1B	ACOT7	LCN2	RP11-315C6.3	GNL2	LINC00599
VSIG10	DGCR6	CSK	SPECC1L	FAM70A	TAC3
PHF21A	NDUFA7	MRPS22	MYCBP2	CKAP2	IPCEF1
LCP1	BZRAP1	KDM1A	SLC25A4	AFF1	KIAA1107
KIF1C	UNC13B	PEX11B	ARHGEF3	PALLD	FOXG1
RBM38	RAD23B	SUPT4H1	MNT	HADH	SST
PTH1R	CHGB	RNASEH1	PER1	USP3	TPD52L1
FKBP5	SH3BP1	UBAP2	AC006978.6	FBXL7	PNMA3
ZDHHC14	NKRF	TRADD	NR3C1	MXRA8	BZRAP1
APBB1IP	HSPA8	HIST1H2AC	SLC27A3	GEM	OVGP1
GNG11	CX3CL1	CREB3	FTSJD2	TCF12	NOL4
ROM1	BAI2	PSMD12	KDM3B	HLA-DMA	BSN
PIAS4	NCDN	DHX40	KCNS3	TINF2	CTD-2527I21.4
HES1	SLC4A3	NONO	JAK1	ELP4	NR4A1
TCN2	UBE2N	CDK2AP2	LEMD3	TPM2	MEF2D
F11R	PRPSAP2	ADSS	RPL10	MTR	JAG2
GLI3	ABCC8	TMCO1	PLA2G16	PROS1	GNPTAB
GPR125	ODC1	COPG1	PRRC2B	TNPO1	EGFL7
TTC28	TARBP1	PSMB2	SCAP	FEN1	SIRT3
SSH3	IDH3G	ARFGAP3	TMEM2	STAB1	NEFL
RP11-347C12.1	STAM	ECM1	EHD2	SPRY1	HS3ST2
BGN	RPL26L1	LONP1	EFNB2	PEX12	AFF3
BST2	UBE2L3	KIAA0664	TENC1	PLSCR1	ARHGAP32
TREM2	GUCY1B3	FHL2	TRAPPC12	SERPINH1	SSBP3
RP11-574K11.16	USP9X	ARL3	FBXL12	KDM5B	MBNL2
LLGL1	SPINT2	BSG	TYK2	AC098614.2	SELENBP1
HAUS4	ODZ4	COG4	FCN3	GNAI3	MINK1
LAMB2	EBNA1BP2	NET1	FZD4	SALL1	NPY
GPNMB	REEP5	LCMT1	PLCG2	LUC7L2	TMCC2
RBM39	PMPCA	EIF2B4	FPR1	GAL3ST4	PPM1H
LITAF	RDH14	AC005740.3	CACNA2D2	RFC1	TMEM35
MGA	HLTF	STOML2	CLK1	AKAP8	LRRC20
MAPKAPK3	KLHDC3	LAMC1	EGR1	SLMO2	STIM1
ZFHX3	IARS2	PARN	UBR2	DHRS4	CKMT1B
SYNGR2	MTMR4	TDG	CRIM1	SYNCRIP	KCNAB1
PRB1	POLDIP2	NDUFA7	N4BP2L2	PLSCR3	PSD
TCF3	ERCC3	SMAP1	MARCH7	BTN3A1	RP4-791K14.2
ATP10A	CACNA2D3	RPA2	PLLP	CXCR4	CDKN1C
ERF	PDCD6	PSMC3	CXCL12	CALU	FGFR2
SPTSSA	NAA10	VPS37C	PLSCR4	PRMT3	PRKCB
SOX12	PSMB2	LY96	CCL4	RAB32	GAD2
RAMP3	PSMD10	SNRPD1	PEPD	ARHGEF10L	RND1
NFKB1	CDS1	HNRNPF	CD2	SRPRB	EFNA3
TGFBI	ASS1	IGFBP3	IL10RA	PTBP1	AP3B2
NES	SKIV2L	SDF2	AC008738.1	CHD7	SLC7A8

STAT5A	FAM5B	LRRC8D	MMS19	E2F6	KB-1460A1.5
RTEL1-TNFRSF6B	FAM134B	DUSP11	EDN1	TBL2	BDH1
CHST3	BUD31	TIMM23	SMARCA5	UBE2D4	TMEM246
FAM120C	CLTB	GRB2	TMEM222	APOBEC3C	AGPAT4
FXYD5	FRG1	HMGB1P4	MAP3K4	GCN1L1	KLK6
FERMT2	NTRK3	TAF6	GCH1	PRSS23	WDR37
RORC	CD2BP2	RBBP8	TLR2	MYC	GPR27
DNM2	ZDHHC6	TIMM13	FBXL5	ZNF408	SRPK1
TCF7L2	SYNDIG1	AKR7A2	DEGS1	LEF1	GAS7
HOMER3	SPOCK2	GTF3C1	ICAM1	FPR1	PORCN
KHNYN	AP000275.65	KDELR1	UBAC1	NOTCH3	ADARB1
MTRR	SUMO3	SRM	AC067852.1	PAICS	MGAT3
RP11-802E16.3	AGTPBP1	TAF9	CYBRD1	MED1	GTDC1
LDLRAP1	PKIA	RAB1A	TEX2	DAG1	LMTK2
PRKAB2	ORC3	MCM7	SERPINA3	DPYD	KCNN1
ZHX3	LINC00094	MRPL23	ANXA8L1	LSM2	SEC61A2
COL11A2	RASL10A	NIF3L1	PTPN6	NAA38	CDKN2D
MED12	UNC119	PMF1	F3	MMP2	ST8SIA3
RASL12	CHMP2A	WDR61	CPVL	OSBPL11	FUT8
YES1	MLST8	ZNF146	TSPAN14	ATF3	SYN2
MSANTD2	RNASEH1	GFPT1	MITF	CTD-2008A1.2	ARL4D
CHD1	VPS39	RP11-255B23.1	ADCY9	ARPC5	CRABP2
ARAP1	COPS6	PFDN4	ANKRD27	RP11-552M11.4	PARM1
XPNPEP3	ASB13	PSMD10	AC034102.1	HMGB3	ALDH1A1
SLC2A1	SLC35B1	GNL3	CBX7	CDKN2AIP	WNT10B
TMX1	RP11-231C14.3	RNF220	FAM107A	ZNF330	SH3GLB2
GLI1	MMS19	SNAPC5	HAGH	TMEM5	MAPT
KIF13A	GRIN2A	SNRNP25	CRYBG3	DTYMK	RYR3
SLC4A2	TCP1	FN3KRP	GBP1	IFIT2	FBXL15
MTSS1L	APOO	CD46	HMOX1	CD163	CNTN2
BCAM	RAB3A	DPM3	N4BP1	ZBTB20	GDAP1
CXCR7	PHYH	ZNHIT3	IFI44	SAP30	BEGAIN
GIMAP5	PDCD6IP	PHB	CGGBP1	DPY19L4	RMND5B
HEXA	ATXN10	RAE1	PDGFRB	ZNF22	TMEM8B
NRIP2	TSNAX	YIPF1	IFIT1	ASAP3	PTK2B
KANK3	PRKAR1A	LMAN2L	CTSE	EIF4EBP1	NIPAL2
SRGN	TUBA3E	PPME1	MED15	RIPK2	PPP1R13B
FRYL	PCNP	DHX16	WASF2	PSMB8	BCL7A
C1QTNF3	PCMT1	PTS	AAR2	BCL6	LGI2
LUC7L3	PARD6A	LAMB3	SREBF2	NNMT	GJB1
TTC31	ZNHIT1	SLC39A14	CORO1A	EFEMP1	MFSD2B
KCNJ16	PIGP	KIAA0196	COL4A3BP	TIMP4	HSF4
RRNAD1	CAPZA2	SERF2	MCAM	TIA1	PDK2
ADAMTS1	HSPBP1	IRF3	PITPNA	POLD3	TSPYL1
CCP110	FAM206A	RP11-397A15.4	TMBIM4	C3AR1	SNCG
TBC1D2B	CSNK2A2	PMVK	VEZF1P1	OFD1	BAIAP3

ITIH5	MTHFD1	ERBB2	SELPLG	MFSD5	RGS4
MAF	RND1	CKLF	SERPINB9	LAMB2	SYP
Z82188.1	PSMD12	HIST1H4C	RAMP3	HYAL2	TMEM151B
AC093668.3	RP11-111K18.1	MOB4	RP11-181C21.4	ADAP2	PLCB1
SAMHD1	TMOD1	VDAC1P1	FCGR3B	PRR11	KLC2
CAV1	SNCB	ATP13A1	ATXN2	RSU1	COBL
KLKB1	TMEM59L	MX2	RBL2	RNF40	NRXN1
GMPR	SCG2	PRAF2	EVI2B	DCHS1	STMN3
EHMT1	ANXA6	STAU1	UTP3	HAUS4	ATP8A2
DNALI1	PISD	PSMD5	LPAR6	CRTAP	EPHB6
PARP12	CACNB3	DNAJC2	MAP2K3	AC004941.5	KLHL18
LMCD1	DNM1L	MCM3	HSPB8	SLC35D1	OTUD3
TRIM22	SLC25A12	DNAJC9	TNFSF12	NUP37	GNL1
DTYMK	SLC8A2	C1GALT1C1	CD48	GAS1	OGDHL
ABCB7	MED24	GIPC1	SLC31A2	MAN2B1	DBP
HCLS1	PEX19	VPS45	TGFB1	PHF10	RAPGEFL1
TRAF4	FUT5	MPC2	FHOD1	MIOS	AHI1
ENTPD2	FAM69A	RAP2B	KIAA1279	SEPHS1	LIN7B
RP11-96D1.10	WNT10B	PPM1G	DPP8	PRPF31	FLRT1
TMPRSS5	CHST1	PTRH2	RP1-37E16.12	CAPN2	PTPRU
PDE9A	MAPK9	PEX19	HEY1	NRAS	WIPF2
GRAMD1C	RP11-142L4.3	RARS	GCHFR	PUS3	ARF3
TBC1D16	RNF5P1	PPP1R2P3	TSPAN7	CLP1	NMNAT2
DDR2	PITPNA	CPNE1	MPP1	NVL	DDX25
TMEM63A	TBC1D10B	MPHOSPH10	CD82	VEGFA	EXOSC7
LFNG	FBXO34	DVL3	LRP1	MUTYH	RAB26
GAB1	VPS33B	COL5A2	APOD	SQRDL	KCNC1
WWTR1	TOLLIP	FTSJ3	CHRDL1	NMI	PKNOX2
PHACTR4	CDH10	NUCKS1	TXNDC15	VAMP5	B3GNT4
PAK1IP1	SYT13	PRKCSH	PURA	QSER1	ABCC8
IRF8	GRINA	RP11-173D9.3	WDR11	ZNF136	PPP2R2D
SFTPB	KIAA1467	ABCE1	UBL3	EML3	NAV3
GLTPP1	ISG15	GOLM1	KRCC1	FLNC	SLITRK5
POLA1	ATP5J2	SLC1A5	TAOK3	MSANTD2	ENPP4
NOMO3	TTLL7	PDHX	C3AR1	IFI16	CDC42
WASF2	PTGES2	MRPS18B	SF3A3	IFI44	RAB36
ADAP2	PPP2R2D	S100A14	ECHDC1	BRMS1	PSIP1
LYL1	SOGA2	RP11-785H5.1	CD4	POU3F2	RPRM
HSPB2	PTRH2	HAX1	MYO1F	DENND2A	ZDHHC11
AC013461.1	VDAC1	EIF4EBP1	PCDH17	CAD	GJC2
RP11-661A12.7	CCDC85B	SLC25A1	THAP11	IFT81	RIMS2
TMEM204	FAM134A	ATP5J2	SFXN3	SERBP1	CLCN4
STK3	PPFIA4	SFN	POR	RPIA	LPCAT4
C3AR1	AZIN1	CXCL10	ARRB2	LIMA1	RELN
HIST1H2BD	QRICH1	EMD	UBA7	TXLNA	ARHGDIG
RAB20	PLK2	NME7	RPS4Y1	ITGB2	PITPNM1

PXN	ARL4C	NUP85	GNA11	RAI14	GPR162
C1R	PPL	TARS	IL2RG	ZNF232	SLC36A1
Y_RNA	ARF5	NAT10	TNFAIP2	MDC1	PPL
TRPS1	BCL7B	SKIV2L	PHF10	TMX1	MICAL2
CYP3A4	CLCN3	CKS2	HMBOX1	EGFR	LHX2
PLEKHG3	KPNA3	MRPL48	KLHL2	GBP1P1	CSDC2
NBR2	SKIV2L2	UBXN4	FRG1	HSPG2	PTPRT
HIP1	SUMO2P1	RP11-142L4.3	VPS11	GALNT2	ME1
POU3F3	YWHAZ	ATP9A	SGCE	ILVBL	MAPK10
GJB4	UBE2D2	HLTF	THEMIS2	SLC4A2	DGKQ
AKAP10	KCNJ6	SH3GL1	COX7A1	ABCB7	NTRK2
SHB	GRPEL1	TPI1	FOLR2	DAB2	CPLX2
ZBTB20	IFIT1	DDX27	TRAK2	SEC14L1	ERBB3
RHBDF2	CUL1	IRF7	ABCA1	MVP	FASN
SMC3	MUL1	MTIF2	HCK	PRKD1	SLC8A2
SHPK	CSE1L	SUMO3	BTN3A2	ASCC3	RP11-566K19.6
FYCO1	GSS	DDT	TNFAIP3	NFKB1	PKP4
FADS2	EIF4ENIF1	SLC39A1	SKP1	REC8	NTRK3
CTC-203F4.1	LSM7	MTX2	PACSIN2	LSG1	SEC14L5
SPON1	BAIAP2	COMMD8	BAZ2B	TEFM	RP11-411B6.6
RP11-183E9.3	MT1P3	BCL7C	IQSEC1	LIPT1	RP11-164J13.1
FOXJ1	PPP1CA	MRPL42	ZNF395	TMEM115	IQSEC3
MMP14	HNRNPH2	FTSJ2	MUL1	RP1-37E16.12	AC010336.1
KB-1896H10.1	UTP18	SDF4	TSPYL2	OXSM	RNF8
SDCCAG3	GCSHP5	LSM4	NPDC1	MITF	WFDC1
TBL1X	THAP4	PREB	TSPAN4	SMURF2	ELOVL4
ATP11A	HRAS	LYPLA2P1	DSE	TTC28	SIRPAP1
TUBB6	SAP130	EBNA1BP2	PLEKHM2	PIEZO1	AC005895.4
RYK	PPP1R13B	ACVR1	DDHD2	LFNG	FGF14
RBM5	IMP4	FASTK	AGAP5	DDR2	EMX2
PPARA	SDHD	SUCO	RP11-799B12.4	SMARCC1	ATP2B2
RPL37	RPA2	USP10	TCTA	TM9SF1	KCNJ6
PPP1R2	CTSB	S100P	FILIP1L	MANBA	MYRIP
EDN1	NDUFS3	NUP93	DHRS4	GRB10	ARHGAP35
GATA2	UBE2B	RP5-1187M17.10	PKN2	STT3A	CDH22
SIRT2	PNPO	RP11-164O23.5	CALM1	IGFBP4	FOLH1B
KRI1	NDUFS4	TSR2	RALGDS	CDC73	BCL11A
S100A11	PARN	NUP54	GAPVD1	ZNF292	GABRB2
RAB30	LY6E	MRPL11	SLC25A46	CHD1	RASL11B
DDB2	MAP1S	TMEM189-UBE2V1	STAB1	EFTUD1	PIP5K1B
ZC3HAV1	COX7C	TSTA3	HNRNPA3	SWAP70	NTSR2
ATP10B	RP11-395L14.17	ABCC10	SPRY4	EMP1	SCAMP1
TSPAN6	ME3	STT3A	NSFL1C	RAB7L1	LHX6
PTK2	APLP2	GLRX3	CRY1	CDYL	FBXL18
SLC6A12	KIAA0319	HMGXB3	USP32P2	PAXIP1	PAK1
HAUS3	KIAA1279	MRPL22	MAPK3	FAF2	ACBD4

NOTCH3	ALAS1	LAMTOR2	PCTP	INHBB	CNTN1
UBA7	GSTO1	NUP205	MANBA	NEDD9	NRXN3
RPL31	TUBA3C	RP11-15K19.2	CDC42BPB	RRBP1	FAM155A
LILRA4	CAB39	CHD8	LAMA5	PLGRKT	SLC2A6
PPP2R1B	SREBF2	HDHD1	ALDH3B1	PDLIM1	SLC1A2
CHST11	GPKOW	GAR1	CLOCK	TBRG4	R3HDM1
S100PBP	TWF2	SETP14	PGC	HPS5	GDPD5
PCM1	MRPL9	AC064843.2	RNF111	CBFB	ZNF536
AHNAK	MRPS34	ALDH18A1	ZFP106	VASP	CDH18
TNFAIP2	PCYOX1L	POLDIP2	CYP51A1	KATNA1	CALB2
UBE2D3	HMG20A	HAT1	AC005035.1	UMPS	STXBP6
SLC25A20	GAD2	BCKDK	TCEAL1	PNP	VIPR1
ITGA5	PTPRT	TMEM45A	SMAD7	METAP2	NEIL1
ACSF2	RPH3A	AGRN	DNASE1L1	GINS1	POU6F1
MICALL2	MBTPS1	PCBD1	GIMAP4	ATP10D	MLXIPL
ZFP36L2	OXR1	RP11-542C16.2	PLCG1	FCGR1C	FAHD2A
ZNF207	FAM179B	MRPS28	ZNF274	GMFG	DLK2
U6	ATP13A2	EIF2B2	NAA60	APRT	JPH3
HLA-F	PWP1	SEC22B	ATP8B1	CPQ	NOVA2
PGPEP1	RTF1	CDR2	KLF2	COL4A2	PCYT2
CD34	CAPZB	HNRNPL	FABP4	BGN	KIF5A
PRKG1	RHOBTB2	TRIM39-RPP21	VIPR1	RP11-435I10.4	NGB
ATHL1	ALG8	TMEM134	TSPAN31	RARRES2	TRANK1
CDKN2C	GMPR2	ECI1	CAPRIN2	HMOX1	PLA2G6
SIPA1	SNRPB	CHI3L1	FAM111A	ASL	ARHGAP26
CTNS	POLRMT	SLC39A4	CDC16	FAIM	KCND3
DENND4C	HLF	AGR2	MAP3K3	NCAPG2	PPFIA2
RRAS	MRPL16	PLS1	PCDHGA1	CKLF	ADAM23
DAPK2	UBL4A	SPR	DYRK4	UTP14A	SLITRK3
PIEZO2	POLR2K	RRS1	NTAN1	HLA-F	ELAVL3
LGALS9	TMEM246	TARBP1	CRK	SMC4	LARGE
ZNF37A	ATIC	BACE2	APIP	PPFIBP2	BCL2L2
RASSF8	SRM	TMEM161A	NR2F1	TYMS	LRRC8B
RAB33B	ZNF629	CCZ1	C7	METTL1	FGFR3
TNFRSF10B	NOP56	PRRC1	KIAA1598	BTBD7	PNMT
TAF1	CREB3	ISG20L2	PRNP	NBPF3	MCTP1
CD302	PMM1	GALNT3	GORASP1	RBM7	ABCB9
ZFR	BCR	NAA10	MICAL1	FICD	PRX
BTBD7	DDX10	BMS1	FLRT3	NKIRAS2	DLG3
TEAD1	AC010170.1	DERL2	PI4KAP2	SLC25A24	ODZ3
GALNT10	MAP4	SNX7	SLC19A2	DDX19B	PPFIA4
PKN1	SLC25A38	YME1L1	TACC2	PRKD3	PRSS3P2
KCNQ1	STX18	MRPS16	LAIR1	DOLK	PTPRN
MCC	PSAP	SMYD3	PLXND1	TMEM8A	SETD3
SCAF11	TIMM23	CTAGE15P	SECISBP2L	VPS54	SPTBN2
GADD45G	NOL4	CCDC86	CYBB	BLM	UNC5C

EMP2	NDUFA2	RNF113A	RAB4B	IQCG	ATP1A3
MS4A6A	STK25	TIMM10	SLA	FKBP5	FRS3
SLC43A1	MRPS18C	COL18A1	MED24	ELF1	CACNG3
RCC1	TRAPPC3	MYL6B	PLCD1	PTPN13	TUBGCP2
AIF1	TIMM10	VGLL4	PPP1R12B	POLA1	MAPK12
ZNF701	CACNG3	UBE2M	KLF4	EIF4E2	CCDC64
PLP2	TUBG2	SRP72	PLEKHA1	HMG20B	KIAA1644
CPQ	ELMO2	NOP2	DHX29	PSMF1	CNNM4
ABCD4	EIF2B2	ARL1	CTNNBL1	F11R	RP11-571M6.8
MAP3K1	MRPL46	CKAP5	UFSP2	GOLIM4	AF238380.3
BLM	RSL24D1	PSMA7	DNAJB2	MED28	MYH11
CYR61	DLD	PLVAP	FAM193B	METTL18	DAGLA
ACVR2B	CCT6A	RALA	LGALS9	PDPN	FAAH
NDE1	ARPC1A	BUB3	SPN	RFC2	ARHGEF7
NUMA1	PRKCB	GUSBP11	TMEM127	ITGA7	NUDCD3
LAIR1	CSRNP2	GLRX2	CSF2RB	PIAS3	TRPM2
RPL17	UBB	POLD2	DYSF	IRF8	MAG
SLC4A4	DDX50P1	STYXL1	GGT3P	MSX1	DTNB
PCSK5	CLIP3	PPIH	ELN	LRRC42	ASPA
SERPINH1	PCDH8	PAIP1	CTSF	BMP2K	HIF1AN
PRKY	ITFG1	GTPBP4	AKAP13	SNCAIP	POLG
SBF2	AIMP2	TTLL12	GAB2	FAM57A	CACNA2D3
MTMR11	KIAA0753	MRPS34	CYTH1	DND1	FAM131A
RHBDF1	MEST	SIRT7	PBX1	LAMA5	CHRNB2
HLA-F-AS1	ATP2C1	VCAM1	PCK2	FMOD	B3GALT4
MAPK7	RANBP2	ASNA1	HLA-DPA1	SLC35F5	SUOX
CIDEB	ELAC2	AP003419.11	STXBP3	NUBP1	PRKCQ
RARG	CIRBP	AL162458.1	RPS6KA1	HEXA	TUBA4B
ZFHX2	RFX5	PSMD4	RASSF2	CDK5RAP2	STAT4
GTF2H2C	TMEM127	PLOD1	ARMCX2	ITGA5	ZMAT4
NXT2	HOMER1	TUFT1	FGFR3	HRH1	ADRB1
CD4	TBC1D9	FNBP1L	SLC9A1	CHPF2	MAPK11
UBTD1	SLBP	COL15A1	AAK1	P2RX4	ZNF385D
KLF4	LANCL1	CD2BP2	CYP4B1	ACP6	HTR2A
FXYD3	THYN1	DDX23	OLFML2A	CD2AP	TMEM180
STAB1	DUSP14	CHCHD7	RHOB	SLC25A20	MYT1L
DHFR	LRRC40	EMC8	KIF13B	TMEM168	VGF
ITPK1	UBE2O	ARMC1	FAM193A	SNX4	RNF208
FRMD4B	KCNAB2	CPSF4	FSTL3	RBM28	TBKBP1
TGFBR2	TCTA	PLGRKT	MAN2B2	HLA-DQB1	NELL1
FPR1	RPN2	GRPEL1	ERCC5	DPP3	ENTPD3
WHAMMP3	EHD3	HSPH1	MCOLN1	AK2	IDS
FSTL1	RP11-44F14.11	TMEM106B	LAMB1	ERO1L	CACNA1A
MYO15B	MTX2	CECR5	AHNAK	COL1A2	NEFH
TMEM176B	AC159540.1	TBC1D10B	PHF2	METTL8	CLSTN3
H6PD	COPS3	BCAT2	GTPBP6	CUEDC1	SYNE1

STARD8	PSMA5	PRMT5	SIRPA	CAV2	SIGIRR
PPP1R13L	PSMD3	VRK2	RSAD1	CNPY3	SPTB
FANCC	CLOCK	MORC2	ARHGEF17	HIST1H2BK	FA2H
HPS5	TMEM14D	HIST1H2BK	BSDC1	NT5DC2	ARHGAP44
PTCH1	TRIM9	CNPY2	TSPYL4	FXYD5	FGF12
PTPN6	NECAB3	SNRNP27	VAMP5	CCNK	PPFIA3
USP6	KIAA0513	CBR1	GOLGB1	POLR1E	PITPNM3
LMOD1	PARP2	THAP4	IL10RB	MCMBP	CAMKK2
PLGLA	ANAPC15	SLC9A3R1	GSDMD	CTSK	THEM6
ITGA10	METAP1	CCT5	DIAPH1	TRIM14	SYNJ2
GEM	NECAB2	CPE	RGS16	FAM114A2	ZBTB44
EPB41	STOML1	SCRIB	SPRY2	RAB20	CPNE6
IL3RA	ARFGAP1	CTD-2323K18.1	NR1H3	TNC	MAP3K9
ARHGEF40	MCM6	PHLDA2	CABIN1	CCDC94	MAPK8IP2
COLEC12	RP11-574F21.3	TYW1B	TSPAN8	REEP4	GALNT14
CDK19	BNIP3P1	TMEM8A	NCOA1	TARBP2	PPP1R1A
LRCH3	KCNK1	R3HDM4	ALDH3A2	ERI2	SLC22A14
SALL1	PSMC4	CANT1	DNM2	NCAPD3	SH3PXD2A
HIN1L	HTT	EBP	PMM1	VAMP8	ACSL6
EVI5	IDH2	CFB	RBM6	SLC31A1	DLGAP1
DOK1	OCRL	SLC22A18	SLC25A38	HIBCH	GRIP2
PSMB9	SYT11	TPRKB	RP4-753P9.3	YIPF6	FAM188A
SLC43A3	TPPP	RP11-395L14.17	NRGN	LTF	CHN2
ABHD4	RALGAPA1P	ZNF259	BRD1	ALG3	WIF1
LRRC23	YWHAH	ZNF593	VAMP3	STK3	OLA1
RP11-192H23.4	DEF8	AC016292.3	ZZEF1	PGAP2	TESC
SLC27A3	TIMM9	TIMM9	TSC2	ACOX1	GDAP1L1
EWSR1	DMXL2	MFF	LAMA3	IFNAR2	PVRL1
RP11-804A23.4	PSMD11	MPDU1	QRICH1	RP11-738E22.2	TFAP4
SLC7A7	ADIPOR1	MRPL12	BCL7B	TGFB1I1	P2RX5
DDX39B	PKNOX2	ABCC3	FAM192A	PCBP2	INPP5J
SLC38A3	PCCB	TSPAN6	SERPINE1	LIMS1	KIAA0319
ARHGEF10	AP2A2	HMGB3	SRPX	TP53I3	ZER1
SGSM2	TXNDC15	CPSF1	STX2	ZNF140	PRSS3
OSMR	TAF9	PMPCA	STX8	TTC31	SEMA6C
RASGRP3	ZNF428	ABHD3	ATG9A	SMAD9	RTN2
IKBKB	TMED3	TOMM34	CCL21	RASL12	NEBL
LEPREL1	MED15	PPIC	AC091171.1	S100A4	FOLH1
TLE4	NMNAT2	NARS2	WDTC1	HES1	PEX5L
G0S2	TOPBP1	CCDC109B	SIGIRR	MORC2	ZNF204P
BMP7	LARGE	RP11-454H13.6	TMEM140	LMCD1	MCHR1
IPO8	PWP2	BNIP3P1	FOXN3	SLC2A5	GFOD1
PLEK2	KDM1A	CHPF	IDO1	TMEM45A	ITPKA
LTBP2	PRKRIP1	DPAGT1	SYNM	GLIPR1	CELF3
FRMD4A	HN1	PXDN	CTD-3074O7.11	PDE9A	ZNF280B
RPL13P5	PRPS1	GTF2E2	MARCH2	LMNA	RASAL1

TGM2	CELF2	AP2B1	MED13L	GMPPA	EFNA5
GYPC	NIPSNAP1	IGHV3-9	IL1RN	PYCR1	RIT2
KIAA0494	MORF4	ARL4C	PBX3	STK38	TMEM86B
STAT3	USP20	DAZAP1	EBAG9	SLC43A3	CHST8
LSS	LRRC8B	ZNF532	MIS12	SLC2A10	DHRS11
CD44	DDOST	RAD21	PPP1R15A	SMC5	CACNB2
ELF1	CDC37	RPIA	ASAP2	SOWAHC	KCNJ9
TBC1D2	VPS37B	ASNS	ABI2	CEP192	MOG
SLC44A1	PPIB	FADD	CA2	OSGEPL1	CDS1
PTPN21	DUSP22	THOC1	PIAS1	C1S	CHRM3
MDM4	THRB	NSUN5P2	DDAH2	PLAUR	ELAVL2
MAN2A1	HMOX2	CLP1	VPS39	GALNT7	DOC2A
CD2AP	VDAC3	CLPP	OBFC1	IGF2BP2	DLG2
LAT2	B3GAT3	MKKS	PRF1	HEATR1	CRLF1
ZMYND8	PRNP	TSKU	RP11-287D1.3	TRAM2	GABRB3
OFD1	BECN1	GOT1	DEF8	PIM1	TGFA
RBM7	ECSIT	EIF5A	SLC46A3	TMEM209	PDE4A
SMAD5	IFT122	CDK5RAP1	SOD3	SMAD1	PDE1B
ASXL1	GCAT	FAM206A	ROCK2	SUCLG2P2	KALRN
SERTAD3	SCAND1	FASTKD1	NECAP1	VCP	ADCY2
CCDC85C	EPHB6	RP11-29H23.5	UNC119B	ACY1	VPS53
NAGA	OXLD1	TMED1	BCL2L2	STAG1	EZH1
GJA4	KCNA1	TMEM11	WDR7	PARP12	MUC3A
NCOR1	PIGH	KIAA0284	IRF2	PKN1	CDC42EP2
ABCA1	DYRK4	GYS1	APOL3	PCDH12	CNTNAP2
DOK2	AP3B2	ESRP2	CITED2	CFLAR	IQSEC2
TMEM51	KIAA1644	ZNF217	NCKAP1L	TGS1	CA10
IFI16	SNAPC5	TXNDC9	CCL3	PIPOX	CRYBB2P1
ARHGAP29	EGR3	WDR12	USPL1	RBM15	B4GALT6
CBS	POP4	DPP3	ITGAM	HS1BP3	GABRA4
TMEM106C	sept-05	MRPL28	KAT5	DSE	ABHD6
COPZ2	RAPGEFL1	NUDT3	AUH	SETDB1	MATK
EMP3	PRKCE	NRBP1	DNAJA2	NRP1	DNAAF1
EMP1	SMS	EEF1E1	CELF2	LAMA4	RIMS1
YME1L1	CDR2	HIST1H1C	CTC-479C5.10	SMCHD1	INPP5A
SH2D4A	HMGCR	BRMS1	WLS	PCTP	ATRNL1
SP1	SEC22B	MTHFS	TOM1L2	RRAS	EIF4E
LST1	RP11-831H9.16	MEN1	PKD2	C1GALT1	FRY
FANCE	PDXK	CDC34	FAM117A	TTC38	RFPL1-AS1
CXCR4	DLST	MAN1B1	DCP1A	RP11-170L3.8	NUDT18
MTCP1NB	PRDM4	DPY19L1	ARAP2	COQ2	RBP4
ROBO4	MFF	GPR89B	SLC1A1	PYGL	CACNA2D2
STAMBP	CTD-2323K18.1	TXN2	RP11-553L6.5	ELK4	CHAC1
GCFC1	CCNDBP1	PPCS	SVEP1	FAM114A1	SNN
SPHK1	NEFL	FAM127B	CX3CL1	CHI3L2	CACNB4
ZNF20	PSMD5	GTF2IRD1	CDKN1C	ZFYVE26	ADRA2C

QKI	PID1	ABT1	GPR137B	RGS1	FGF9
THSD4	LIN7B	LYPLA2	FYCO1	TGDS	PRSS53
ELN	CRELD1	SRPK1	MNDA	WDR43	NKAIN1
SMC1A	COPZ1	HES1	GOLGA8A	IGF2R	KLK1
MYO19	AC074182.1	SIL1	BEX4	S100A8	PAK6
LIMK2	TBCC	CYP1B1	EXT2	VCAM1	DBNDD1
SMAD6	PPP2R5B	GCLC	TBC1D2B	PRPF40A	GRIA4
CERS4	RP11-298C3.2	MGAT4B	CTD-2270L9.5	CLEC2B	GFRA2
PELI2	CCT2	UBD	BCL2A1	RREB1	PNOC
RHOD	DNAJC11	TCF20	TIAM1	GLI3	RP11-445H22.3
ACSM5	TSSC1	ATP5D	FAM65A	HMHA1	CRTAC1
SLC15A3	CAPRIN2	SEC61A1	GCA	LAIR1	RIMBP2
TAF4	RIMS1	LAGE3	MAN2C1	SPATA5L1	STS
MACF1	PRKAR1B	PMEPA1	FGR	POLG2	ARPP21
PLOD2	GDI2	DUSP12	EMC3	FYCO1	SLC9A5
SEPP1	TDG	AMPD2	HMG20A	ANGPTL4	SLIT2
MED13	MAN1B1	SART1	CRYAB	LMNB2	GABRG2
SMURF2	AGPAT3	TFB2M	NECAB3	NADSYN1	PDE1A
RAI14	FBXL15	FAM18B2	ADARB1	FAM60A	B4GALNT1
EPB41L4A	SLITRK5	NKIRAS2	FEZ1	DNMBP	TRPM3
PDLIM1	TMEM248	FKBP11	CDH5	UBE2C	SIDT1
GDPD3	PIGZ	PSMG1	LEPREL1	TCN2	GABRA1
KLHDC4	TFPT	ESRP1	BTAF1	Z82188.1	CNTFR
RBMX	CCDC25	MRPL13	GFOD1	RBMS1P1	GABRD
IL2RG	RP1-40E16.8	OXCT1	LIMS2	IFI35	DDX54
HMOX1	SHROOM2	RPS21	RP11-44F14.11	TM4SF1	KCNA1
CTC-338M12.4	FTSJ2	TOPBP1	UQCR10	SSBP1	NEUROD6
GNG12	ARF1	COX7BP1	PPP6C	CD248	PIGQ
SRPX	CD320	BET1	CTNND1	COL6A2	DARC
IRF7	NDUFA5	TRIM27	RP11-79D8.2	MAP3K2	CDK19
PSME4	RNMTL1	CPSF6	TINF2	TGIF1	SHANK2
SLC14A1	FBXO7	STAMBP	LST1	OAS3	SCN2B
CAST	RABGGTB	ARHGEF5	UIMC1	NAA40	HR
ELK3	MAN2B2	KAT2A	AL163636.6	HERC5	PYGM
BEST1	NQO2	NIPSNAP1	IGFBP6	RUNX1	REPS2
APOL3	GNAI1	ACY1	PLK2	ELN	VILL
IRF2	ANO3	TRAC	APOC4	ADORA3	ASXL3
ITPR2	ARMC1	RP11-229E13.2	TRIP10	EIF4A1	PIM2
NEK1	UAP1	SERPINE2	ST6GALNAC2	RHBDF1	KLK7
POLD1	MTCH2	ACP6	STXBP2	SLC7A7	FAM189A1
ID1	RP11-29H23.5	RAB22A	EIF3A	GBP1	ABHD8
ARHGEF5	RNF10	GTF2A2	SLIT2	SLC26A2	ADAM11
TCL1B	BCS1L	NRIP1	INPP5K	ATXN7	RHOF
VCAN	RPF1	POLE3	EXTL2	ECT2	OCA2
SLC2A5	RP11-255N24.4	SRPRB	KIF1B	PCNXL4	CNNM2
RPS6KA1	KIAA0368	FURIN	ACVRL1	RYK	RAPGEF3

GGT5	CAMK1D	REEP4	ZNF331	ZNF282	MMP17
DLEU1	ACTR1A	NOC3L	IFFO1	NOP14	FCHO1
RECQL	RIMBP2	TBCC	LPIN2	AL603926.1	PPIP5K1
PAPSS2	KLHL26	SMPDL3A	DDX60	SP110	SEMA4F
PITPNB	CASD1	PLOD2	ANKRD46	LAT2	ZFYVE9
CAPG	ZNHIT3	DARS	KLHL21	LOXL2	NEURL
RP11-235E17.2	TOR1B	SSRP1	IL6	LAMB1	TAC1
ITGA8	GLS	UBL4A	IL2RB	SHMT2	ASIC2
NFATC1	EMC3	HIST1H2BD	IFIT5	ANP32E	RP11-57H14.4
IL13RA1	ABCE1	RWDD2B	HLA-F-AS1	LEPRE1	CWF19L1
DGKG	EGR1	HMGXB4	LAMP1	NAGA	STAT6
MCM5	LRRC49	DDX18	WFS1	CPVL	ARHGEF11
P2RX4	NME5	RPP38	ECHDC2	AHR	DAPK2
MARCH3	DUSP12	DDX56	ZNF580	FCER1G	CCKBR
RHOQP3	ARPP19	AP1M2	USP3	LYN	GFRA1
CSF1	RBM42	EIF2B3	SF3A1	DONSON	CEACAM3
PERP	PEMT	HARS2	SLC2A14	ZNF799	RAB40B
IDI2-AS1	PDIA6	LOXL1	MARCH8	G3BP1	ADAMTS8
USP36	LGMN	TCEB1	ZC3H12A	UBA7	SPTBN4
SAMD4A	SNRPA1	LTF	EVI2A	TRAF5	EXTL1
POU5F1P4	AHCY	CD320	CSNK2A2	DCPS	CACNA1I
NR2F2	SATB2	TMEM251	ZNF189	RNASEH2A	ACTL6B
ZEB2	YIPF1	DRAP1	TMEM164	PRIM1	CGREF1
FAM111A	DYNC1LI1	SDF2L1	BTBD3	ALG13	RUNX1T1
TRIP10	ZSCAN18	AASDHPPT	RP11-458I7.4	FBN1	CHRD
TRGC2	NACAD	AGL	LMBR1L	SERPINE1	NEUROD2
KIAA1551	LONP2	BCR	ARHGAP25	TMEM164	PLEKHG3
OCLN	RAP1GDS1	EIF2B5	VRK3	F13A1	FAM155B
RP4-753P9.3	PIGT	UNC119	HOXA5	PCOLCE	CYP26B1
TNFRSF1B	ADSS	FAM114A1	GGTLC1	WNT5A	TESPA1
PRR14	DOCK3	ENO2	OPTN	CCDC101	CACNB1
BLOC1S1	ULK1	WDR70	SLC34A2	COL18A1	ICA1
HEYL	WDR37	LOXL2	AGER	TMSB15B	ANKS1B
CNTRL	DNAL4	MAPK13	CREBL2	MARCH3	GRM2
CYP1A2	ACOT13	FAHD2CP	CIRBP	ARHGEF40	DUSP2
CD22	TRPC4AP	TERF1P5	WDR73	SNAP29	CADM3
GFRA1	PIK3R4	SCCPDH	LAT2	LGALS9	SPRYD7
EIF4EBP1	FAM173A	PRRC2A	ECHDC3	ASCC2	ST6GALNAC5
CASP7	SLC25A11	AC016732.2	EML3	MAPKAPK3	NMT2
SLC35F2	NDFIP1	IGLV3-19	GAS8	ARID5A	PODXL2
MLL	NRSN2	ALG3	SLC25A24	PPIC	GRIN2C
NAGLU	MRPL40	EIF1AX	PGRMC2	HAUS3	FAM174B
SLC7A9	BTN2A1	SAE1	PRKD2	WIPI1	B3GALT2
ITGB2	DCAF6	ESRRAP1	ROBO4	PAPSS2	GRM5
MX2	B4GALT3	ALG6	NAPA	NIPA2	TMEM74B
SBNO2	ATPIF1	PCCB	ITPKC	CEBPG	TRIM8

ADA	DNAJA2	UTP11L	RB1CC1	ATM	SYT12
RGR	GNL3	RP11-255N24.4	F8	GIMAP4	SLIT3
FBLN1	SRSF2	FAM203A	ZC2HC1A	IL17RA	CACNA1B
ZXDC	KCNF1	MCM2	ARHGEF1	PRKY	GRIN2A
HMG20B	KIF3A	APOO	WDR37	ACTL6A	PCSK2
ARHGEF26	TACO1	DECR2	SGSH	TPX2	GLT25D2
PLAUR	EDEM1	SAP130	EGFL7	SLC39A7	SSTR3
LEF1	FSCN1	DDX10	TTC31	TTC27	BCL11B
NCAPD2	GABBR2	POLR2E	PRKCDBP	TMEM51	CAMK1G
CD180	MRPS18A	GRB10	TSC1	BTN3A2	FABP3
MAP3K6	SDF2	DBN1	TRIM38	MCM5	CA7
RIN3	NAPG	SEC23A	GABARAPL1	LIMK2	ZBTB7C
ADAMTS9	ABCF1	IBTK	FOXO1	CTNS	ATF7IP2
WNK1	CORO1A	CETN3	EZR	TUG1	DOK4
ZFHX4	IFT46	ATP6V0E2	MMP19	CDK6	AQP7P3
TGFB3	SGSH	TNPO3	RANBP2	KLHDC8A	MXRA7
LIMA1	PPM1G	UBN1	RHOBTB2	C2	STXBP5L
ACVRL1	EBAG9	COL5A1	RNF38	STK17A	RAB3B
TWSG1	ING4	CEACAM5	DOCK9	FAM129A	RGS7
GJC1	FAM214B	ERN2	MADD	TTI2	PPP3R1
ALOX15B	LY6H	MRPL39	EFEMP1	DLEU1	LDB3
FNDC3B	PSMD13	STK39	TGFBR3	CD4	CSF3
NPFF	TMEM41B	MTMR4	C5AR1	NUPL2	CAMK4
SMC5	DYNC1H1	RP11-54K16.2	TSTD2	POFUT1	SLC4A8
PSMB8	AGGF1	DKC1	ARAP3	AL118508.1	MVD
PPFIA1	SLC27A5	GDF15	THUMPD1	TMEM140	CPEB3
TNFAIP3	CCDC51	SLMO2	LDLRAP1	HSPBAP1	CALY
PCOLCE	USO1	PSME3	TCN2	SLPI	PCBP3
ANP32A	CLPP	HTRA2	GPSM3	sept-10	ZNF467
MTMR3	CTR9	MAD2L1BP	WDR6	PTPN6	HUNK
CLK3	SYP	UBE2S	DARC	COL1A1	RP5-955M13.3
FKBP10	RER1	BAZ1A	CSGALNACT1	RP4-564F22.2	ZNF839
ITSN2	TSPYL5	MRP63	KLC1	MICALL2	GNAL
IRF1	SET	UMPS	PPIG	PTP4A3	RGS6
COL21A1	ITPR1	CTD-2116N17.1	PRKCD	CFHR1	P2RX2
PRF1	FHOD3	IGHV1-69	FOXF2	STEAP3	GRIK5
FCGR1C	ITPKA	OAS3	FCGR2A	ALOX5	LMO3
BHLHE41	MAK16	UBE2G1	SNX1	SLC27A3	B3GNT3
MKS1	EIF2S1	ICT1	PCDH12	SDF2L1	NXPH4
RBBP6	SCN2B	RP11-552M11.4	FCGR1C	HIST1H1C	DGKI
ETS1	CDK20	RACGAP1	MACF1	MTMR11	GABRA2
HSPB6	OSBP	PSMD13	SLC29A3	TRA2B	PENK
EIF4A1	AC083899.3	GMNN	TPPP3	ASTE1	MEPE
OR7A5	GANAB	TNPO1	VWA5A	DHFR	RP11-18A3.4
SNCAIP	TSC2	DPM2	TRIM25	PDGFRA	MPPED1
PACSIN3	MCOLN1	LYRM4	SECTM1	STAT3	FRMPD4

CTD-2555K7.2	TEX10	RPN1	FAM129A	RP11-802E16.3	IL1RAPL1
PTPN13	TYW1B	RNPS1	ENOSF1	JAG1	CA4
CEP290	SNRPF	ZWINT	TBC1D5	SNAPC1	TBC1D30
SNAPC1	SGCE	FPGS	PGAP2	MED17	RGS14
GIMAP4	NDUFAF4	UBAP2L	ATP2B4	CDH11	ABCG4
TLR5	WDR12	MRPS18A	ZNF862	STK17B	DLG4
ACSL5	ACD	SLC41A3	SUPT6H	IFRD1	CDK5R2
HIF3A	POLR3B	DDX11	CYP3A4	MAPK7	CABP1
IFI35	TARS2	BRE	MARK3	LSM5	KIDINS220
ALPK1	HMGN4	USP21	RAP1A	CCND1	POPDC3
RP11-429B14.4	NAPA	LBX2	NOTCH1	NUDT1	PBX2
HMHA1	BRWD1	ITGA2	SESN1	CD44	MFSD6
MMP2	ECHDC1	POLR1B	KIAA1551	ITPR2	CACNA1H
PCSK7	RNF113A	RFXANK	FAM53B	CALD1	AL590369.1
AUTS2	MGST3	SSSCA1	SIPA1	GALNT10	KIF17
RFX4	LRRC20	RFNG	WWTR1	S100PBP	MAPRE3
DCLRE1C	BZW1P2	RRP1B	APOLD1	SOX11	PDE8B
IGLL5	MYOM2	NUP37	IL17RA	ZNF217	ZFR2
SWAP70	RANGRF	USP39	WFDC1	PROM1	PLIN1
ST18	DHPS	RP11-77H9.2	MAP4	EIF3B	TTR
OLFML2A	HMGCS1	HOXB2	ZNF32	METTL3	XK
DAB2	ETS2	COMMD10	IL15RA	ECM2	RP11-414J4.2
FBRS	CRNKL1	NENF	PLEK	TNFRSF10B	TXNRD2
MCM7	P2RX5	GNL2	REV3L	ARAF	CALB1
DYNLT1	SAP30BP	TIMM17B	SCMH1	LY96	HECW1
ARSD	GDAP1	CTD-2008A1.2	ARMCX1	TCEB3	SEMA3G
DPF3	SCFD1	NDUFB7	RP11-574F21.3	RNASE6	RAX
NPC1	FAM50B	USO1	CHMP1B	CBLB	PDZD7
LGR4	G3BP2	AACS	CD34	BRIX1	YBX2
TM4SF1	ADORA1	PTP4A3	KIAA0195	GCFC1	MAST1
HIST1H2AI	WDR3	IGHV4-4	CD8A	HEATR6	PDIA2
HLA-DOA	RP11-382A20.3	INTS5	PACS2	RGS16	FAM153B
GPR4	FAM155A	TARBP2	CYP51P2	TCF3	RGS11
FZD7	RAB27B	SIAH2	STAT5A	TGFB1	ALS2CL
MYO1F	STAT4	ELAC2	MS4A4A	RECQL	PRRG3
PCDHGA10	CANX	DGKZ	ASRGL1	SLC33A1	GLS2
LPP	DHX29	SLC7A5	CCDC130	AD000671.6	PRPH2
MUC1	FRMPD4	PRPS2	CRTAP	IL10RB	CUX2
FOXN2	RWDD2B	SSNA1	ACSF2	URB2	HRH2
ZBTB40	DEAF1	ERAL1	CLIC3	DDB2	TRIM46
ARAP3	EIF2AK1	ECD	SLC25A28	CD300A	KIAA1045
ELK4	CD200	CPNE3	EZH1	DDX56	PLEKHA6
CARD8	HIVEP2	TEX10	DHRS7B	OLFML2B	CSRNP3
BCL2L11	HTRA2	EDEM2	ZRSR2	MYO1F	DLGAP2
LPAR6	PREB	IRAK1	TNFRSF1B	HNMT	TSC22D2
BRD8	ASB6	AC019097.7	COMT	FAM64A	CD79A

CROCC	KAT7	VCAN	RPS23	RBM38	MADCAM1
BTK	APRT	PLAU	TERF1	SLC38A6	FUT1
MFNG	SNCG	TULP3	DENND3	EIF2S2	CLSTN2
PHKA1	GABRA1	ERO1L	CST7	TMED5	PPP1R37
RBFA	FJX1	SLC50A1	ADRB2	NACA	NTNG1
DIAPH3	COPE	MMP7	VTI1B	PHLDA1	CHRFAM7A
STAG3L1	MTERFD1	HNRNPUL2	RCAN2	PANX1	PRKCG
VAV1	SCCPDH	CHPF2	FAM46A	RB1	TNNT1
USH1C	ADCY1	PPA2	RNF10	LPCAT3	RFPL2
PRDM16	UTP3	UBE2NL	AHCYL2	SIPA1	DGCR5
FAM114A1	R3HCC1	DLG5	UVRAG	ANGPTL2	CACNG2
HIST1H1C	SIL1	WDR3	ZNF142	SLC35F2	ZNF804A
FMO4	FUCA1	E2F4	EIF4ENIF1	ZWILCH	CDHR1
CFHR1	GPS1	RTCA	MPHOSPH8	BACH1	SPOCK3
PDZD2	DPH2	HILPDA	CRY2	SLC16A1	ICAM5
CHPT1	PAM16	SMC4	IQGAP2	NR1H3	ZNF238
USP53	MATK	FUT5	MEF2A	BAZ1A	PTPN4
PARP11	HTR2A	METTL13	KLF11	SAYSD1	LPIN1
EPOR	ODZ3	G6PD	SFPQ	BMPR1A	GPR21
PLEK	SCG3	PPAN	KLF9	KIAA0020	APBA1
SMTN	RP11-15K19.2	SLC7A1	SETX	PLVAP	ZDHHC18
PROP1	RRM1	TMEM165	TMEM47	HNRNPA1	TNFRSF25
CD163	ATG4B	USB1	RNF123	CASP2	SLC6A12
ANP32E	SYN2	ACIN1	LDLR	UBIAD1	LARP4B
RGS1	EMC9	EXOSC8	ETV5	TRIM38	SMPD3
SP110	DRAP1	SMG8	ATXN10	LEPREL1	RASGRF1
BIN2	MRPL15	LDOC1	TCF7L2	POLR1B	LIPA
PLXNB3	MTR	SAR1A	SLC25A20	PSPH	HSPB3
IL18BP	ANK2	AIFM1	BAMBI	NLRX1	HSD11B1
CAMK2G	CPNE6	BCS1L	NKX2-1	ZKSCAN5	PLCH1
SERHL	SMYD5	ARF6	TBC1D13	TMEM38B	RPP25
MYOF	SERF1B	NIPA2	CRYL1	MYOF	AC124309.1
HEY2	WARS	TRPC4AP	NR1H2	CTBP2	MYH14
IL10RA	CIAPIN1	TRAPPC6A	TDRD7	WEE1	COLQ
RENBP	RTCA	DHCR7	SIRT1	ZNF394	AC012065.7
PHF15	KCNN2	TAF2	CALHM2	SOX4	PCLO
BACE2	ASB8	ASUN	CASP7	CD97	RLBP1
DENND1C	TSPAN3	TMEM106C	MSRA	IRF1	ARRB1
TRAPPC10	AKIRIN1	RFX5	GPR153	NUP88	FAM49B
ACSS3	JAK1	ZNF574	MZF1	IFT52	ANK1
BCL3	RNF123	GPKOW	UPF3A	ITGAM	ALDH1A3
AL773572.7	INTS5	CNDP2	STAT5B	QPRT	KCNMA1
BCAR3	ARGLU1	TRAPPC2L	ATRIP	LRRC1	DRP2
CP	C1DP1	TRIT1	AMT	TLR2	NRIP2
SERPINA5	DPY19L1	LEPRE1	MT1P3	AL162458.1	SLC16A8
ADORA3	RP11-651P23.4	PPT2	CNNM3	ABCC3	KCNK3

STAG1	ANAPC1	HSPBP1	PTGER4	SLC35C2	RAB17
DOK3	SLC35E3	MBOAT2	HDAC5	PGM3	CACNA1F
CILP	SRPR	SEPHS1	HNRNPA0	NFATC1	GABRA5
RGS10	NDUFB7	IGSF3	LPHN2	DOCK6	DGKE
KCNJ8	NSG1	CMPK1	HEXIM1	TIMELESS	BDNF
CLDN15	R3HDM4	PTPLAD1	FAM171A1	ZNF606	FABP6
YAP1	MRPL17	TUBG1	SECISBP2	FILIP1L	TTC9
CTDSP2	RP11-13N13.2	ZZZ3	NINJ2	UBFD1	SNHG14
STEAP3	ABCC5	ZNF212	NMRK1	PDIA5	HOOK1
GPC4	HSP90AB1	SLC5A6	NACC2	LIG1	NPY1R
TUG1	SRPK1	OSBP	CXXC1	SMAD5	CRHBP
RREB1	SELRC1	PPP1R13L	PTPRM	SCARA3	ANKH
LYN	EPHA4	POLR1C	BNIP2	PKN2	ANKRD2
TBX19	PET112	PHGDH	IFIT2	TWSG1	KCNC4
PLSCR1	MEN1	TBPL1	RNPEPL1	TMEM100	DYRK1B
ZC3H11A	RAB22A	ZNF629	TFEB	CA12	GCK
SLC2A4RG	TMEM11	MTHFD2	FAM13B	KIF2C	KDM8
BACH1	SPRY4	TM9SF4	CD58	APBB1IP	CATSPER2P1
ITGAX	LYRM4	MRPS15	TMEM80	IL4R	TUSC3
TLR2	F8	TAF15	KCNQ1	SEMA6A	SGCD
CCR5	TMEM208	GCSHP5	TCF7L1	MYNN	CARTPT
S100A4	UXS1	MTERFD1	BRD4	ZFHX3	RFPL3
IL16	SEMA4F	PIAS3	ITGA6	LYPD1	NOS1
RP11-261C10.3	ETF1P1	LSM2	AATK	STAMBP	GLRB
LINC00623	RRAGD	WIPI1	NOTCH4	NCAPD2	PCDH7
DCLRE1B	HNRNPK	PRR11	DOK2	DRAM1	CROCCP3
LEPREL4	SDAD1	PQBP1	DDB2	SERTAD3	CRHR1
DOCK6	ACTR3B	MOGS	SETD3	TMEM187	SYT5
ANXA1	RP11-287D1.3	FLNB	AIP	MAPKAP1	HBQ1
MCL1	MET	GRWD1	GLUL	OLFML2A	PART1
CD84	HPCAL1	ACP1	WDR19	SKIL	GRP
DLC1	HERC6	TUBA3E	TMEM50B	LIFR	CNNM1
JRKL	CCDC53	NDUFS8	ATP8A1	BTN2A2	CCNA1
FERMT1	OTUB1	PFDN6	ARHGAP15	PIGN	GRM1
ADRB2	ZFPM2	KLHDC3	LGALSL	RP11-264B17.3	KLHL35
CTSS	ATG5	R3HDM1	HK3	GJC1	CRH
PTRF	GTF2E1	TSR3	LIMD1	ROBO4	SLC17A6
IL1R1	GOLGA5	COQ2	PRKAG1	LRRC17	PPEF1
RP11-680F8.4	ENDOG	MRPL2	RGPD4	SLC25A13	CYP26A1
ADAMTS2	FAAH	FAM102A	CISH	EHD4	SPEF1
RBPMS	SF3A3	CARM1	MDN1	IPP	GRIN1
PSMC3IP	GAD1	ANKZF1	DUSP1	APBA3	AC021066.1
ADARB2	BCKDK	COX10	ARID5A	PCK2	HRH3
SREK1	PYROXD1	STEAP1	PISD	ZMYM6	SSTR2
NXPE3	PMS2CL	ATMIN	JAG2	NUSAP1	PPARGC1B
SOCS3	SUPV3L1	IGHV3-7	SERPINA1	ANTXR1	EPB41L4B

DPYD	GTPBP4	AC005280.1	CD37	PHKA1	FRAS1
IL32	SUPT4H1	NVL	OPN3	PAK2	MIR600HG
FGR	EBP	MOSPD3	PNPLA2	TMEM104	HTR5A
LSP1	C1QBP	TUBA3C	SAP18	TCF7L1	KCNC3
PRKX	PRSS3	CTC-425F1.4	FGL2	IL10RA	MAGI1
NBR1	EIF2B3	MIIP	HPCAL1	TSPAN12	TP53TG5
RBM41	ARL4D	EHMT2	RGP1	LAMA2	SEMA6B
FAM46A	TUSC2	KDM2A	RASSF1	FAM82B	KIAA1324
CD33	KIAA1045	RP11-737O24.3	CHD1	UTP20	KRT17
ARID5B	INPP5J	CASP3	FHL1	CTD-2116N17.1	CABYR
AL118508.1	TRAF3	KDM5B	SPATA2L	CDCA4	RYR2
BMP1	FADS3	MRPL4	RANBP6	PLEKHF2	LINC00574
CXCL12	TTC13	CD24P2	SLC35A5	MEIS1	KIAA1462
KCNJ5	ACP2	ALDOC	ARAP1	CFI	RNF128
KAT2B	SSX2IP	RAB15	ZNF337	MS4A6A	CHD5
RB1	PFDN4	MAGOH	CAMTA2	RBM34	NTSR1
LY6G6C	ARHGEF7	TTC13	ANK3	CTBS	CYP2E1
KDM5A	MRPL23	RFC4	GIMAP6	SLC35D2	CACNA1D
FZD9	PPP1R11	PKMYT1	PHC1P1	SAMSN1	CDHR2
CALD1	PSMB7	CSNK1G2	HIGD1B	SMC1A	BRSK2
CCR1	GSPT2	SRP72P2	ROCK1	AC006011.4	ANKRD26
POU5F1P3	EIF3A	SLC16A3	SORT1	EVI2B	MST1R
CASP10	RMND5B	SSR3	MAPRE2	CARD8	RP1-240B8.3
SCARA3	TRIM28	KLF5	NPR1	PRKD2	SLC6A15
PTCD1	PCCA	GALE	ANKRD40	DNASE2	ZDHHC8P1
ST6GALNAC2	FARP1	RUVBL1	EPN2	KHNYN	CASR
UBE2C	ZNF259	AC083899.3	XPA	IL13RA2	ATP2C2
SVIL	ATRN	SHARPIN	BACE1	HAMP	TRIM17
SCAF4	DPM2	ACOT7	NAT6	CASP7	GNA14
FCER1G	ORC6	UBE2K	PSIP1	TRMT13	SYN3
SOS2	KALRN	PAFAH1B3	COQ4	RIT1	TRIM3
MYOM1	MYH10	VPS54	BIN1	ABHD5	PVALB
RHOQ	EEF1E1	UPF3B	CCND1	BACE2	BCAS4
RFX2	ARF3	HDAC2	KCTD2	SMC2	PPP4R4
ALOX5	MCTP1	ZNF76	CNN1	FHOD1	PCDH11X
LAMA3	FAM13A	ABCF3	CCDC101	PES1	CHAD
ECHDC3	GRSF1	SHQ1	CCR5	MLF1IP	PLCL1
CFDP1	NEUROD2	IGHV3-30	TSPAN9	RP11-395B7.7	KCNH4
HOXB2	KIT	GPS1	CIDEB	AP4M1	PREPL
TNXA	ADSL	MUTYH	BIN3	CRYGS	CDH8
PHKG1	BCAS3	HIST2H2BE	ADCY7	CYBB	CRYGEP
LLGL2	sept-07	SRRD	USP13	MYBL2	ADRA2A
ITGAM	UNC13A	ZNF783	NDST1	VAV3	HAS1
CTGF	DDT	SSR1	OSBPL1A	STON1	TMEM121
TRAF3IP2	MRPL22	ANO10	MID1	CPT2	TAF4B
SLC12A4	GABRD	CLCN7	CYTIP	BARD1	AFF2

KDSR	CHST2	MLST8	OGT	CCDC22	DUSP9
LCN2	AP2B1	MAGT1	NR4A2	COL3A1	CNKSR1
FAM160B2	GHITM	METTL5	INPP5E	MAF	RP11-616M22.5
AC055811.1	DCUN1D2	GLTPD1	RP11-267N12.3	CYBRD1	KRT83
CFH	CLSTN3	DEPTOR	EPB41L2	ACSS3	PRSS3P1
HERC5	KPNA4	ISOC2	PILRB	CLCC1	RP11-560G2.2
RP11-499P20.2	EXOSC9	ACLY	AQP9	CCR1	PROZ
PILRA	PORCN	ZNF408	RCBTB2	GUSBP3	ACHE
P2RX1	UBE2K	UBE2H	SOCS3	AP5Z1	TBR1
P2RY6	COG5	PRKRIP1	GLT8D2	SLC15A3	PAK7
FBXW4P1	SMYD3	PUS7	SH2B1	PIGA	DRD5
FER	AARSD1	EIF4G1	VEGFB	KCNJ8	HTR1E
NAIP	PAK6	FAM49B	ZNF394	SRBD1	PRMT8
RPS20P22	TLN2	CAD	RASIP1	DNALI1	OSBP2
FAM60A	FAM162A	GTF2E1	TPGS2	PLA2G5	ART3
MORF4L2	RCOR3	ITGB3BP	RAMP2	WDR91	GREM1
TES	EXOC3	SRSF1	DUSP7	IRF7	SV2C
KIAA0754	FAM184A	NQO1	FXC1	PYCARD	ABCA4
VPS13C	TRAPPC4	CLDN4	SEC14L1	ELTD1	GRM7
RNASE3	NFU1	SPCS3	ECM2	RHBDF2	AGMAT
RP11-1319K7.1	SCMH1	ISLR	NCALD	PLEK	AJAP1
HBP1	SEC23A	UNG	CXCL1	TACC3	TNNC2
CA5B	TMEM8B	MRPS2	CHFR	MX2	CRYGD
SMAGP	ADARB1	FANCL	NUCB1	ITPKC	LAMA3
TCF7L1	RPP14	PWP2	MEIS2	GINS2	CRABP1
TLN1	HMGA1	ACTL6A	SCD5	FZD1	KCNQ3
U73169.1	PANK4	OLFML2B	THTPA	METTL21B	PTPRR
PIK3R5	CHMP2B	UBE2J1	TJP1	DBR1	BSPRY
RP11-395B7.7	PPP2R5E	PYCR1	NEDD4L	TNFAIP3	KCNK12
SPSB1	SERGEF	NAT9	HLA-DOA	RCC1	SLC6A7
SLC26A6	SUPT7L	CCDC51	ACOT1	CDKN3	KCNJ3
OGFRL1	CALY	RBM15	RFC1	POSTN	IDI2-AS1
DCN	ATP9B	SNRPA1	OR7E5P	AGPS	SLC7A10
SLC9B2	MRPL35	TRIB3	FBXO31	TGFBR1	MLLT4
PMFBP1	VRK1	SLC2A10	REC8	MDFIC	TRHDE
LRP2	SERPINF1	AC005943.2	TAPBPL	PLA2G2A	CCNO
PARP6	UBA5	GMFB	HCAR2	ZNF639	FPGT-TNNI3K
CST7	VAMP1	CHD4	DNAJC15	NUP98	GPR143
TAL1	POLR1D	FXR1	GPC3	AR	DBH
PRKD3	CCS	TRIM29	FLT1	SYNRG	GPR12
OR2N1P	GPX3	VDR	IL1B	EDEM2	ATP2B3
PYCARD	RP11-263K19.6	TRMT61B	ITGA8	RP11-77H9.2	CDH7
DDX6	RAE1	FAM3C	ASF1A	MELK	CASQ1
TNS1	CYB561	SULF1	RAD54L2	FCF1	HCN2
ABHD5	MBOAT7	SAR1B	TOE1	GGT5	BAAT
SUCLG2	GNL1	TMED2	USP12	IMPACT	FNDC8

PRRX1	TRAPPC2L	TMEM184C	TIE1	ZKSCAN4	RCVRN
CLU	PRMT5	CDCA4	KPNA3	MASP1	SSTR1
PSPH	MRPS7	PIGF	IFT88	G0S2	EPHA5
SLC11A1	NCBP2	TRAF5	PIN1	ABCC1	SLC1A6
MECOM	LRPAP1	PNKP	RSBN1	ZNF20	SLC22A8
PLCE1	TMEM160	TIMM23B	KATNA1	TTLL4	RP1-101G11.2
AVIL	ZNF419	SPHK1	CADM1	OXTR	WNT11
PPIC	IDH3A	ZNF544	CUTC	FCGR2A	ADCYAP1
MYBPH	TPM3	TFF3	HERC2	FER	MCF2
KIFC3	RAB6B	SNX15	HMGCR	NUP160	ALDH8A1
OASL	UROS	DFNA5	RASL12	CLPB	P2RX6
FGF2	UTP11L	EIF5B	OCEL1	AP3B1	CRYGC
CAV2	GMPSP1	AD000671.6	TSC22D3	THEMIS2	FEZF2
TOP3BP1	TSR3	AL136419.6	TTC17	PQLC3	NPY5R
SIX5	GTF2F1	DGAT1	ABI3BP	CPM	SLC6A20
FOSL1P1	PTPRN	COL16A1	DUOX1	CHCHD3	EPHA7
RBM47	HNRNPA0	DNM1L	LINC00341	ESPL1	SLCO1A2
HEMK1	PRSS3P2	TOR3A	PHF1	FAH	INHA
MYBL1	PARM1	BAK1	SGMS1	PTPRC	OVOL2
SOX13	ELOVL4	TGDS	ITGAX	HLA-DPA1	KCNQ4
SNAP23	SMG8	TOMM40	TLN1	NUP43	SCN8A
GOLGA2P5	PSME3	NUP88	IVD	GPX7	WNT7B
KIAA0040	GRIN1	ZNF292	CLU	FAM48A	FZD9
SIGLEC15	PHB	RP11-74E24.2	MLLT4	TIPIN	MYO15A
CD300A	RP4-791K14.2	IPO13	STARD3	TSR1	EGR4
BNIP2	DOC2A	MED8	CHMP6	MXRA5	STAR
CPM	HS3ST2	CSE1L	FAM189A2	PINLYP	SHANK1
EYA2	GPR89B	MORC3	PPP1R3C	MIS18A	CDH12
CD2	MED9	COG5	BCL6	MBD4	KCNK10
RETN	TBP	SMARCB1	CLIC2	ADA	TDGF1
CLCN2	ACTB	EPHB4	INSIG2	SLC2A4RG	CTRL
RP11-612B6.2	PUS7	LAD1	TMEM115	MORC4	SFTPD
EVC	TRMT61B	TPST1	DCN	SERPINB1	FSTL4
NBEAL2	NUP93	SYNCRIP	GSTM5	ALMS1	RHBDL1
TSC22D4	PTP4A1	TACO1	LSP1	UST	HPSE2
HLA-DOB	SPIN2B	RCC1	GOLGA6L5	MFNG	CORO2A
RP11-370B11.1	UQCR10	GPN3	ACOT9	CA9	CDKL2
GART	ALDH5A1	AMPD3	GNE	PSPHP1	NLRP2
CDC42BPB	PTPRN2	IMPAD1	MBP	TNFRSF1B	RHO
SYK	CDKN2D	EXOSC5	TNFRSF14	BICD1	SLC7A4
SEMA6A	APEH	NUAK2	KLF7	RIN3	CYP1A1
ZNF516	SF3A1	THAP7	GBP2	BNIP2	OPRL1
ZNF562	MKKS	PTP4A1	FRMD4B	FAM46A	LMX1B
DEF6	TRIM36	PRKDC	ANKRD11	CENPF	FRMPD1
LCAT	ABI1	CDC42SE1	SOWAHC	RND2	AC012074.2
AL356740.1	COQ4	AC074182.1	WAPAL	NPL	KRT6A

APAF1	UBE2G1	COG8	PANK4	TNFAIP6	SOSTDC1
IGHG1	TAF9B	PAM16	PPP1R26	TPRA1	VIP
SOWAHC	SETBP1	BATF	SRI	DOK1	RP11-783K16.10
SLC2A9	LAMTOR3	CD164	HERC5	DSCC1	CIDEA
EFHC2	NMT1	CDK5	BTN2A1	ERMAP	TECTA
AGFG2	NIT2	INHBB	STAC	LYZ	GLRA2
IL6R	NUP54	MRPS10	ME3	NIP7	FAM163A
ZMYM5	TESC	TMX1	TTC28	POLD1	BEAN1
CD177	PTK2B	ANKRA2	WISP2	ZNF227	KCNA10
TGIF1	NAA35	S100A2	RP11-94I2.1	STK10	GREM2
TRIOBP	PPIH	GPR56	CD69	HIST1H4J	KCNJ12
NUAK2	NUP85	RAP2C	TBC1D2	COL6A3	OVOL3
ICAM1	CRIP2	LDB1	DGKZP1	PGPEP1	ADTRP
NKTR	SEC61A2	SLC39A7	TLE2	BAX	LGR5
SH2D2A	ELP3	GID8	IFT122	FANCF	MC4R
ACOX2	CACNA2D1	PTK7	MFNG	GADD45G	FAT2
NKX2-2	BEND5	LTB	RAB11A	FZD5	FIGF
CSPG4	TSTA3	CRABP2	ADCY3	INSM1	KCNA4
CYTH4	ZMAT4	AC004797.1	BRPF1	GYG2	BHMT2
FMO2	COX7BP1	CIAO1	KCNAB2	FN1	RXRG
ATF1	DOK5	NMB	RP11-488C13.7	DBF4	SLC22A13
EPHA2	YIPF3	GOLT1B	PPP1R12A	DDX60	CCDC68
SMEK1	TMEM97	GTF2H3	APPBP2	HEY2	KRT31
PGAM2	NME7	DCPS	HSPA2	IL8	ADARB2
MAPKAPK2	UFD1L	GMPPA	PCYOX1L	SGSH	PTH2R
IKZF2	MPPED1	PIGH	DOLK	NT5E	HTR4
SLC29A2	INSIG2	MAP2K2	NEO1	POLA2	DNAI1
IL1RL1	KPNA6	APTX	SMARCD3	REST	NDST3
CTRL	PRMT8	AGPAT5	RP11-685I11.1	OGFRL1	CACNA1E
AURKC	KIAA0196	DNAJB9	SIN3B	EPB41L4A	IRGQ
PON3	ICT1	GSK3A	ARIH2	TRIP10	ASIC3
DRAM1	COX10	ATP5G2	SAV1	ABHD4	DNAJA4
TGFB1I1	B3GNT4	NUDT15	PLXNA2	BTN3A3	CACNA1C
MKLN1	FKBP1A	IGLV3-25	CLIC4	TMEM39A	KCNMB2
ACAN	SLC24A3	EXOSC4	ATP10D	AC099522.1	CDKL5
B4GALT1	ATP6V1C1	PAAF1	RAF1	SLC10A3	SSTR4
NLRP1	NPTX2	AK2	OR7E12P	CCNB1	HTR3B
IGHGP	RAB11A	DR1	SPEN	SNAP23	IL1RL2
WDR5B	ZNF133	PTGES2	SELL	SNX5	SOGA3
ZC2HC1C	COX5A	TMEM39A	EPB49	ADAM17	MYH7B
C1GALT1	HCCS	SMARCA4	AGTPBP1	CTSC	HERC3
ISG20	GNB1	MGAT2	UBE2L3	RRM2	CLUL1
MICAL3	REPS2	DCK	TCF12	NFATC3	ACTA1
RP4-673D20.1	METTL13	PIGCP1	WIF1	EYA2	TNFSF9
TNFRSF10D	PSMC3	RGS1	CPED1	PDSS1	GABRA3
MICALL1	MAD2L1BP	SERBP1	LCMT2	HOXB2	KCNV1

RIPK2	ELMO1	TMED7	ZBTB16	DIDO1	ALOX12B
CCDC69	LCMT2	RPL28	MARC2	FOSL2	RP11-706O15.1
HSD17B3	DLG3	MAST2	RALGAPA1P	ZNF200	INSRR
TAAR5	CTPS1	TUSC2	GATA6	DHX57	GRIP1
COL1A2	CHRNB2	FBXO34	NFIC	CCNB2	HERC2P3
OR2H1	ZFAND1	DCTD	SUV420H1	FZD7	GNG13
ANXA3	DDX25	DOLPP1	GPR183	AC007362.1	FLT3
NFATC4	TMEM30A	ZW10	SNX4	GIMAP6	ANKRD34C
CLEC1A	IPO5	RPS6KC1	FOXF1	QTRTD1	KCNA3
EMILIN2	NIPA2	LARS	CPQ	AL163636.6	PAH
SRP19	CHPF2	RFK	CHST10	ING3	MPO
ENO3	PSMD4	KIAA0100	DDR2	PLBD1	MTUS2
DST	UFSP2	RTF1	ABHD5	NID1	SFTPA2
KIF25	CTD-2527I21.4	MXD3	MT1M	ODF2	KCNIP2
SENP3	HS6ST1	GNA12	CASD1	GART	WNT8B
FAM129A	CAMK2N1	AC135048.13	CHD3	BCAT1	RP11-707M1.1
NPL	MICAL2	NMD3	LARGE	ASF1B	FSHB
NANOGP8	THAP9-AS1	MBOAT7	UNC45A	DCLRE1B	CLCA4
HAP1	ELAVL4	SMG5	EVL	IL1R1	PRKG2
THOC2	MGAT4B	SH3YL1	AREG	YAP1	ENDOU
SLC2A10	MAMLD1	SUPT16H	PPP1R13B	HLA-DOA	PRSS16
CALML4	TACC2	YEATS2	MCC	HP	ZNF702P
S100A8	EXTL1	RAB38	HYI	S100A9	CST5
ZNF217	RALGAPB	GRAMD3	NFATC1	TAF1	GPR22
GUSBP11	SENP2	POLR3K	HS3ST2	INTS7	CBLN1
DNAJC3	BCCIP	CTAGE9	ADPGK	MTFR1	MTMR7
PAWR	MEAF6	TSSC1	MXD4	TDP1	RBPJL
HSD11B2	E4F1	PSENEN	LTBP4	PPP1R2P4	PGR
HSPA6	SNRPD1	AAGAB	SLC6A14	AURKA	LOR
DNASE1	NRBP1	KCNK1	CCR1	SP100	OPRK1
LTF	IDS	NFKBIE	MTR	SLC11A1	FBXO40
TRGV5P	COPG1	THY1	SELP	ALPK1	CRX
EMCN	RIMS2	CHCHD3	MSL2	LCAT	MUC6
SPRR2D	PEG3	KDM4A	RCAN1	SCUBE2	ANKRD55
FCN3	RP11-49C24.1	PUS3	MYO15B	GMPR	RPS6KA6
FGL2	LDOC1	PNPO	HPS5	DLEU2	SMPX
PSG7	AC005280.1	MCM5	MAL	PLK3	GPR63
UNC93B1	DSTYK	SLC2A1	PPWD1	IFIH1	GDF10
FCGR2B	RAD51C	CEBPA-AS1	FGD2	CDCA8	KCNB2
OR2C1	NEDD4L	CNTD2	FAM179B	RHOQ	DPYS
OR1D2	IFI6	UCHL1	RUNX3	ENTPD5	CDH20
ITGA1	SSB	TBRG4	KIAA1033	H2AFJ	AKAP5
GID8	FGF12	MED1	IQCJ-SCHIP1	CENPO	ADRA1D
PLBD1	TOPORS-AS1	SMYD5	PCGF2	CHST11	TLL2
PDLIM5	ERI3	ZBTB33	CREBBP	PILRA	UTF1
METTL21B	PDZRN3	SLC35A2	ASAP3	NOL10	IL12RB2

DISC1	MPC2	NCAPD3	WNT2	WIPF1	AGAP2
RP11-134P9.1	ARHGEF9	PYGL	GLUD1	CTSS	N4BP3
NRG2	MAP2K2	VAC14	KDR	IGLL5	INS
CDK2	ARF4	IGHV3-23	DYNC1LI1	LXN	CDH9
CEP135	HNRNPA3P1	FAM3A	RND1	RRAS2	GPR6
LY6G5C	SDHB	ZFAND3	WDR47	MAGOHB	EMX1
SLC1A7	TRPM2	MTPAP	INPP5D	FXN	HCRTR2
PCDHGA2	CAMK1G	AAAS	UBR5	PTX3	CACNA1G
HIST1H2BI	SURF2	GNA15	BCAS3	MYBL1	FOXI1
RNASE6	KIAA0317	AC100791.1	KCNJ15	ZBTB1	LMO7
ANGPT1	IVD	TOMM70A	CYTH4	APOL3	TUBBP5
TSPY2	LSG1	MAN2A1	KCNJ8	CDK1	TSKS
LSR	ARHGDIG	TIMM8B	TSSC4	CASP6	HTR1F
SERPINA1	ABCF3	SLC6A8	ARID3B	HEATR2	CNTN6
ZNF273	CITED1	VCP	GATA2	LEPREL4	GUCA1A
ROR1	CCDC91	RHBDF2	HSD11B1	NID2	DRD1
SERHL2	AAGAB	FASTKD3	MAPK7	PSMC3IP	SPAG6
CSF3R	VIPR1	TLK2P1	TSPAN3	SLC25A37	AC110619.2
SUZ12	ZNF331	HOOK2	ANAPC2	IPO4	LPAR3
EPX	RBFOX2	RPS6KA4	UBOX5	KRI1	TYRP1
PDLIM4	UBE2D4	POU2AF1	SMTN	CTA-204B4.6	PAX7
SUCLG2P2	MAPK8IP2	B3GAT3	GPR126	PDGFD	POU6F2
SLC1A3	MAP2K5	TSN	LAMTOR3	BUB1B	PTPN20A
H2AFJ	LAPTM4B	RP11-406A9.1	FAM214B	CFD	CYP2C8
LIMD1	EXT2	GGCX	HEYL	FCGR3B	SLC9A7
SOX5	TCF4	LRRC42	SRSF11	CTPS2	GAPDHS
CHCHD2	SLC25A46	ING2	MAN1C1	CDC20	RP11-451M19.3
PTPN7	HMGB3	ERBB3	KIAA0240	TAPBPL	RS1
VAMP8	CGRRF1	CBR3	MEF2C	CUL4A	ZNF391
TEP1	GTF2E2	AKR7A3	PSMD11	EMILIN2	KCNJ13
ADPRH	TOX4	HIST1H2BF	LZTFL1	ATP7A	GABRG3
IL10RB	FAM20B	EFTUD1	R3HCC1	SVIL	TRAV16
ZNF124	RNF24	TNFRSF21	PNISR	TBX2	RP11-430B1.2
SNTB1	UBE3A	MED6	FGG	RIPK1	ABCG5
EMR1	PTPRD	TPBG	INSIG1	FNDC3B	MYL2
ASXL2	CAMK1	ARSA	HDAC11	PLCE1	RP6-91H8.1
TP53	TPGS2	SMARCA1	RUSC2	APAF1	SERPIND1
MCM3AP-AS1	CDC42	BGN	B3GNT2	HCK	BMP8A
IL22RA1	RP11-510M2.10	CCL19	SETBP1	HSPA6	IMPG1
ELF4	WIPF2	OXSM	SEMA5A	MAP3K6	
AOC3	ZNF184	CAMKK2	LYL1	SYNC	
GBP1P1	CHMP6	ABCB7	MEIS3P2	KDELR3	
AKAP3	SNX24	HRSP12	ZNF211	SAP30L	
HPSE2	ADAM23	SGSM3	PRX	CD84	
NUP188	MOB4	E2F6	DCAF8	MICALL1	
FOXF1	SLC2A14	PDAP1	ENGASE	ARSD	

MICB	USP39	POLD1	CRMP1	FKBP14
CDA	TSN	FBXW7	DIAPH2	CYP20A1
AC008394.1	PSEN1	TOMM22	ZNF44	LPP
HCP5	ASAH1	NT5DC2	BIN2	WDR5B
CLEC7A	CHMP1B	CPNE7	RAB17	KAT2B
VCAM1	TMEM39B	DERL1	ITGA9	BTK
ABI3BP	TAC3	FTSJ1	SPSB3	MANEA
REST	ATP6V0A1	VARS	EPHA2	CENPN
SP100	MIR22HG	TAF12	GDPD5	ZNF516
TIE1	HINFP	RPL22P22	KCNMB4	CCNL1
MBNL3	TCEB1	NOTCH3	SP110	SCO2
TGIF2	DNAJC12	MINPP1	RBM22	RAD54B
CHI3L2	SETD4	EMC9	PPP2R2A	HOXC4
SELL	MRPS28	SLC52A2	MAST3	APEX2
PLA1A	PGD	FARSA	FAM174B	SEC24A
CXCL9	FHL2	METTL1	SIGLEC1	FANCE
CARD9	TPMT	ARFGAP1	STX16-NPEPL1	THADA
CPT1A	TMEM151B	SIGMAR1	CST6	BLNK
SLC23A3	TMEM126B	CDH3	CASKIN2	KIAA0040
TFAP2C	COX7B	REXO4	MKNK1	TMBIM4
ADAM28	RP11-571M6.8	TRAF4	DMXL1	ADAM28
CYP4F2	PCSK2	ASCC1	TNS1	MRE11A
NUP160	MRPL12	OXLD1	STARD13	TP53
FAM53B	EAPP	SNX27	TBC1D4	CDCA3
NEDD4	TMEM183A	RNMTL1	PAMR1	GPR107
CEP104	RP11-566E18.3	SDHAF1	MAP4K3	MOB1A
PAX6	USP13	SNRNP40	SEMA3B	MAP3K1
RAB27A	BET1	FAM57A	COX16	FDXR
BAZ1A	SST	HCCS	RNF14	TRIM21
P2RY14	PFDN6	ACTR2	PDPR	ADAMTS9
CEACAM1	PPWD1	HIRA	CSF1	ACOX2
DSC2	RNF115	SUPV3L1	FADS3	TP53TG1
RP4-724E16.2	SRA1	UBE2C	PIGA	NTN1
CYTL1	SLC1A1	UBA5	CD1C	NCK1
EPB42	SIAH2	TGFB3	PNRC2	CD58
ELTD1	CYP51P2	OTUB1	ZNF143	PGGT1B
CDH5	RPA1	TAX1BP1	EIF4E2	B4GALT4
NBPF20	ILF2	SEZ6L2	RAPGEF1	FBXL4
SLCO1A2	KIAA0232	ANP32E	HP	ITGA2
MAP7D3	ECD	SPDEF	RAB11FIP2	MALT1
VNN2	CIDEC	HOMER3	GLI3	CTDSP2
TLR4	NABP2	RBMX2	ELTD1	FAS
DOCK2	AP3S2	YRDC	SMAD6	FANCI
RUNX2	GABRG2	PIGC	S1PR4	TMEM194A
CASP6	NT5DC3	FAM222B	RNF128	KIF18B
LMAN1	SEZ6L	DVL2	H2AFY	NARG2

DNAI1	CEP41	IGHV3-20	ZDHHC3	SYK
RP1-302G2.5	SRP72	TRIM2	PEX3	SOCS3
WEE1	SLIT2	GTPBP3	USP47	IL1RAP
U4	B4GALNT1	LRBA	RABGGTA	PLEKHA4
S100A2	RBP4	APEH	UBE2D3	TMSB15A
AKAP9	RAB33A	NID2	RAPGEF5	MYO1E
RRN3P2	TUBG1	MED27	PIK3R1	AASS
CXCL1	SLC2A8	HEATR1	IL18BP	ATAD2
BRD7	TXNDC9	IQCB1	RNF122	ANGPT2
CER1	RP11-392O18.1	ZNF282	HOMER1	OSMR
KANSL1L	DZIP3	NOP14	DNAJB14	SLC16A3
STON1	WDR7	RP4-710M16.1	SP2	SFRP4
FCN1	AQR	ARHGEF16	HTT	ZFP112
CEP152	ACACA	ZDHHC24	SLCO3A1	MFAP4
HAUS5	PPCS	XPO1	TBX2	DPF3
EHD4	CKAP5	DESI1	BTK	LMO2
XPNPEP2	EMG1	SAC3D1	HNRNPR	GPC4
FAM205B	RNF121	DNAL4	DHX38	ABCC4
CD1B	CDK16	SEC23B	SVIL	GCFC2
IFIH1	RP11-227J5.3	ACAD8	KBTBD2	RPS20P22
PTPRC	AIP	TAF11	LYST	SSR3
SLC6A9	DEPDC5	PTPLB	DOCK2	PALMD
SULT1B1	PIP5K1B	VRK1	PRKG1	SNIP1
ANG	OSGEP	PPID	ADCK3	PPP1R13L
KCNE4	UBE2M	XRCC1	DAAM2	CLN5
DNMT3B	MAP3K12	RP11-345J4.8	PLXNB1	TMPO
ECM2	SHANK2	TFPT	ELMO1	SYNE2
FAM106B	NELL1	RAD1	DENND4C	ACVRL1
RBMS2	ETHE1	PAICSP4	SLC26A2	ZNF253
GBP1	COA3	NUDT21	NDRG3	CYTL1
H2AFB2	MCTS1	SPINT1	U2AF1	MRPL19
CD58	AKAP12	AC005895.4	MICA	LARP7
IL1RN	M6PR	WDR46	FCGR3A	IQGAP2
GABRE	TTLL1	ACBD3	BAG1	TLR5
CGA	FBXO21	GLB1L2	GAS7	BIRC5
ACOT11	RAB3GAP1	GPR78	FZD5	DDX58
MYOT	RPA3	RAB3IL1	MFHAS1	CXCL2
PSTPIP1	TBC1D30	TCFL5	GZMA	ATF1
TBX6	RTN2	PKP3	TOLLIP	MNDA
PHF16	ABR	PARP16	ANKRD12	PDLIM4
FGA	PCID2	HIST1H2BE	SLC48A1	HJURP
IL2RA	CAPRIN1	GGH	LAMC3	GBP2
HMGN5	FBXO41	PLEK2	AP001062.8	VANGL1
HFE	KIF2A	SLC1A4	TINAGL1	FOXM1
HGF	OTUD3	MPG	RP11-727A23.4	EGLN1
GIMAP6	CHTOP	MYCBP	TMUB2	ASPH

CEACAM5	DRG2	PMS2P3	DPYD	SOD2
VEGFC	COQ3	BCCIP	CHST12	KDM5A
PAFAH2	PPM1H	YIPF5	RRAD	TROVE2
CXCL10	NDUFAF5	SUGP1	CX3CR1	KDR
CXCL2	MAP4K3	IGFBP5	GPR124	CALCRL
TRAF1	NUDT21	TRMT2A	ABLIM3	RBM47
BPI	ZNF204P	CIT	ATP6V0D1	LIF
RP11-872D17.8	CHL1	TIPRL	KLHL9	E2F5
RRAGC	BCL7A	SIRT6	SDC2	PLEKHA8P1
CPED1	PSIP1	CEACAM1	TRMT1	FCGR3A
TNC	IGFBP6	RPL18AP16	PPRC1	C1RL
RALGAPA1	GTDC1	ZNF232	MON1B	FGL2
TLR6	KDM5B	SLC36A1	GNS	ASXL1
PHF8	TCEA2	TARS2	KLK11	TMEM185B
LILRA2	DLK2	TTC27	CFLAR	SERPINA5
CLCA4	CCZ1	ST14	MYH10	FBXO5
ACSBG2	PPFIA2	RMI1	MBIP	MTHFD2L
RND2	SMARCA2	RPL35P8	EFHD1	ETNK1
CFI	FAR2	FNDC4	VAPA	LUM
NAALADL1	AC016732.2	CEP350	APH1B	NUAK2
AIM2	MED27	GEMIN6	ELF4	ZNF193
PYGO1	FAHD2A	STAP2	PHACTR4	CEP135
TNFAIP6	POR	PIM2	DCTN4	USP18
ART1	MRPL39	TGS1	PKN1	HPGDS
CCNB2	SUV420H1	CD38	DKK3	CASP4
HPGDS	VRK3	MID2	SHC2	FZD6
LRP5L	BATF3	RP11-13N13.2	PRKAR2B	MDM2
CD40	CAMKK2	CCDC90B	SLC2A3	ERGIC3
CCL2	RYR2	ATRN	RRAGD	RP11-397A15.4
LYVE1	GOLT1B	DHTKD1	COL6A1	TFPI
COL8A2	SMCR7L	WRAP53	ALG13	SLC26A10
FYB	ABT1	LRP3	FUBP1	TAF1B
APOBEC3G	PUS1	DPH2	ANXA11	MYCBP
EVI2B	CSTF2T	TMEM39B	COL5A3	SERPINA1
AC099522.1	COPS2	DFFA	CSF3R	LOXL1
AL163636.6	RAB36	PDIA3	MUT	CD86
ACTL6A	NEUROD6	ERGIC3	POGK	ZNF473
RTP4	LAMTOR2	PTPRF	ASMTL	KNTC1
CLEC4A	GBF1	CP	MTMR9	KIF4A
PCDHGB7	PRDM2	PYGO2	ADH1B	TEAD1
UBE2I	ACP1	PTBP1	LAMA4	SLC22A4
ANGPT2	SPTBN2	DLGAP4	RCBTB1	ACTG2
HERC2P3	MBTPS2	RP11-170L3.8	RPS27L	ABI3BP
DPEP2	C2CD2L	TCTN2	SCNN1B	MNS1
FPR3	CCKBR	H2AFY2	RNASEL	RBPMS
LGR5	MARK1	CDK16	EIF4EBP2	IKBKB

FLI1	SLC17A6	LHPP	ZNF324	ZNF167
UAP1L1	NOLC1	WDR77	SEMA3G	RAB27A
STK19	MAP2	NCBP2	TBP	APOBEC3G
PLAC8	GDAP1L1	MORC4	ZC3H13	CCR5
PTPN9	ZBTB11	WASL	BTN3A3	KLHL20
SDPR	GFOD2	COMMD4	MS4A6A	C5
PDE5A	ASNA1	USP1	TRAPPC2	ST7L
SCARF1	EPB41L3	USP5	LRP6	ICAM3
GZMK	FAM35B	AKIP1	VAV1	FGR
CUBN	ACAT2	PDP1	KCNJ2	KIAA0922
SP140L	ENO1	CDKN2C	JAM2	COL5A1
RUNX3	RARS	WDYHV1	WWP1	SNAI2
CYP11A1	MPI	IRS1	FOXA2	F2R
ADAM5	TSPYL1	MAP3K10	HPGD	HK3
ERG	MAPK6	NABP2	ABCD4	EZH2
KDELC1	SLK	IGHV3-21	VPS13C	LMNB1
STEAP4	NRXN1	EPPK1	SPG11	MICA
TEX14	MAP2K4	ERI3	SCARB1	AC013461.1
PHEX	RP11-445H22.3	MAFG	ZNF665	MTM1
CALCRL	GMPS	TRMT12	HLX	ARFIP1
MPZL2	FH	NELF	GSPT2	CXCL10
PA2G4P2	WFDC1	TMEM159	CAB39	BCL2L11
LILRB1	COMMD4	B4GALT2	PALM	STC2
CASP4	EXOSC5	GRB7	HNMT	ZMYM1
PTMAP1	DALRD3	RP4-564F22.2	MRAS	IGFBP5
ZSCAN12	RPP40	KIF5B	USP20	PHF16
SYF2	RFPL1-AS1	IGHV4-34	CTNS	ARHGAP15
ANXA2P3	NUDT15	RNASEH2A	TOB2	CENPM
GPR183	ACLY	H2AFX	sept-10	DHODH
GBP2	RPE	MTMR11	ANG	PRKX
RP11-84C10.2	SIDT1	OVGP1	KLF15	ZNF45
GML	SMARCA4	TIMELESS	VEGFC	SLC16A10
PGR	DAGLA	KIF2A	CLIP4	CDC25A
FMO5	TMEM186	PIK3R2	TAF1C	EFCAB2
APOL6	SRRD	ASCC3	CGRRF1	MS4A4A
KCNJ15	TOR1A	IPO4	NAAA	KIRREL
IRAK4	VIPAS39	OAS1	IGF2	CDK2
HIST1H2BH	ZC3H7A	QPRT	PARVB	RPL10L
CCDC170	GABRB3	SUZ12P	ATP2C1	HLX
PIGR	AHNAK2	RP11-18A3.4	BMP2	LIMD1
NAT2	FXC1	DGCR2	LY75	VSIG10
PAQR5	CSNK1G3	FAM69A	MEGF9	FLI1
LINC00115	SPRED2	CYB561	RP11-872D17.8	CP
BHMT2	LANCL2	ZC3H11A	SAMD4A	TOP2A
TP63	TUBA4B	AVEN	TLR5	FBP1
ZIC3	GPHN	PPP3CB	PPIP5K2	GCNT2

FKBP15	SEMA6D	PALB2	BAP1	NCF2
ITGAL	MFSD5	TSR1	ABCA8	BMP2
ANPEP	SUOX	FAM86A	MIER2	CCT6B
CYSLTR2	CABP1	PDCD11	TEF	SP140L
FHL5	IMP3	IRF2BP1	FCN1	KLHL7
HERC2P5	UBE2A	WBSCR22	MED22	ISG20
CA6	SSBP1	IFNAR2	SNTB2	EMILIN1
SEC61B	DLGAP2	BSPRY	MAP2K5	CHEK2
ENTPD1	NUDT2	CRTC1	MTMR10	HLA-DQA1
RP11-727A23.5	RWDD2A	PPIF	MAPK1	ZNF350
RGS9	MCAT	ACTBP9	HSD17B6	RAD54L
CASP8	RGS17	SLC35F2	GMEB1	PLA2G4A
SNHG3	NRXN3	RHOH	PIK3R5	RFX2
RP11-451M19.3	MFSD6	TNK1	KIF1C	FYB
C6	FAM174B	GGT5	ASAP1	SUV39H2
NID1	SETX	POLA1	sept-08	PGAM2
CD72	POLR2E	SNTA1	STK11	QRSL1
LAX1	ATP6V1A	GCDH	SNX24	TK1
LEP	TBC1D8	NOC4L	RP11-395B7.7	NDNF
GLYAT	EXOSC4	MMP11	CWC25	SPSB1
IL18R1	SETD6	PGF	KAZN	RFX4
PDCD1LG2	RNF219	CALU	AC093734.1	MCM9
AZGP1	TRAPPC6A	RRM1	STIM1	IRF5
TMPRSS3	GPR176	THEM6	EP300	ANGPT1
CPB1	SDHAF1	GALNT7	MTF1	ZNF124
SIM2	ME1	LSG1	CTD-2527I21.4	TEP1
IL1R2	WDR46	RCN3	KIAA0922	FZD2
AL645730.2	TOMM70A	HEATR8	LIPT1	PRRX1
MNDA	SHQ1	APOL2	STAT4	BRCA1
GRTP1	TOMM22	TMEM185B	USP8	PTPN21
COL4A6	TESPA1	ABHD10	ZC3H7A	SAMD9
RNASE2	CPSF6	GPR125	PCBP2	SCIN
ANO2	TMEM35	MCAT	ST3GAL1	LRBA
GLI2	SAMM50	RANBP9	FIGF	CRIP1
HHEX	RBBP4	AARSD1	TNXA	EVC
PRRG4	DOLK	ZNF281	FAM115B	ZNF468
CLTC-IT1	PLCXD1	ARVCF	BATF3	TFAP2A
F2R	SOCS5	ANGPTL4	NEMF	GPR183
RAD51B	TM2D1	CD27	NCF4	PTCD1
IQGAP2	ZMYM3	CDC5L	TNRC6B	MBNL3
EMR2	ZDHHC24	SPAG16	RHOQP3	TTC23
TMSB15A	TBRG4	SYT2	FNBP1	PLAU
SOAT1	SLC25A32	NOL3	SYBU	ANG
PPM1F	METTL1	PADI2	APPL2	ENTPD1
7SK	RND3	PRRX2	ELP4	PRR5L
ITGB8	COA1	IRF6	DCHS1	ZNF107

NPHP1	FAM149A	KRTAP5-8	CAMK2N1	BCKDHB
CDC14A	ELP5	TTI1	BTN3A1	THG1L
C5AR1	PRDX3	TOM1L1	CCRL2	LCP2
BMX	SIRT3	HSPB11	PRPF40A	MAP7D3
HPR	CLPTM1	NSDHL	TEK	NEDD4
SLC6A16	B4GALT7	NRAS	NFIX	IL13RA1
TBL1Y	PNKP	KIFC1	SEC62	EGLN3
MS4A4A	NISCH	STIP1	RP11-152F13.3	LINS
CYP39A1	ISOC2	RPS6KB2	TBC1D17	PIK3R5
EIF3C	ZNF8	IGHV1-2	CNPPD1	EDNRA
IL20RA	GTF3C4	YWHAE	CD86	WRN
SAMD9	FASTKD5	PRCC	CHIT1	KRT8P12
ALOX15	ICMT	ELP5	RP11-82L18.2	IL6R
MCTP2	PTPRO	NDOR1	FAM47E	DOCK2
OR52A1	YARS2	AP5S1	NDUFA4L2	IGF2BP3
OR2F1	ACVR2A	ENSA	CHKA	ADAM12
ERAP2	EIF1AX	RBM28	PKD1	SDC1
F2RL1	GALC	B4GALT7	FAM134B	PCOLCE2
TRIM5	ALG3	WEE1	ZNF419	GINS3
AC008103.5	KLHL18	CTPS1	PTPRN2	GDF15
SLC19A3	TOM1	GEMIN7	DIDO1	MPZL1
HIST3H3	TCTN3	TRIM24	PER3	CD37
AL359757.1	NTNG1	SMARCAL1	TESK2	OAS1
LYZ	PBX1	TMEM132A	SEC31B	PTGER4
MSR1	BCL11A	ADAMTS12	PID1	TBXAS1
OMD	TMEM135	GOLGA3	CRTAC1	ATF5
IGSF6	COIL	LRRC1	DYRK3	IBSP
ZNF345	UBFD1	SFT2D2	FEM1C	SFMBT1
ABCA6	TMEM19	TMEM186	EMILIN2	SRPX2
COL1A1	MECR	FHL3	CCDC94	ELF4
OR7E14P	TTPAL	EIF2S3	NXPH3	SMC6
LAMP3	GPATCH3	JAK3	CRELD1	HOXD4
CXCR2	GABRA4	NARFL	CHI3L2	CPED1
TLR7	ABAT	ARF1	RPL15	TBX19
ADH1B	EGR4	YARS	KLF6	SKP2
LPAR4	CCNE1	C2CD3	NR1D2	ACAP2
TLR8	MARCH5	AC004692.5	FAM134A	TNFAIP8
RP11-714L20.1	DCLK1	SLC2A4RG	ID2	POLQ
ARSJ	FHL1	NOL10	BAHD1	KCNE4
FZD10	DHRS7B	CLPX	RELA	LARP4
CAPN6	U2SURP	BPGM	APPL1	HCP5
CYP4F3	CNNM4	DAXX	LAMA2	RUNX2
WDR78	ABCA5	LPAR2	RHOBTB1	STIL
TEX15	SLC25A14	CLCF1	FLRT2	IL7R
ZIC4	BHLHB9	PRRC2C	PTGS2	KDELC1
S100A12	EXTL3	NPM1P22	SKI	THOC2

SPTLC3	CADM3	ATAD3A	PTCD3	CASP1
IRAK3	KDM5C	TPMT	HEPH	AIM1
SLC16A4	RP11-552M11.4	PTER	DPT	THBD
COL8A1	AKR7A2	GAL3ST4	CCDC25	DTL
RP3-522P13.1	ZMYM4	COQ3	GLIPR1	NRP2
ATP5E	CMAS	RNF121	MAP2	ICAM1
CNTLN	KCNAB1	MTMR2	RAI2	PBK
ZNF117	NPY	COX5A	PHF17	CCNA2
LY75	CCND2	S100PBP	FBXO42	SPATA6
PCK1	PALB2	TMEM135	ZC3H7B	PAWR
ELL2P3	RAB3GAP2	RRN3	TGFBR2	NCAPH
CLEC5A	CACNG2	RAMP1	PLK3	ETAA1
GNRH1	ATP1A3	PAXIP1	ALS2CL	RTP4
RFPL3-AS1	TBL2	SLC30A1	PLAGL1	CENPE
CYTIP	LMTK2	ATP6V1C1	PDCD4	ZCCHC4
IL18	RP11-243J18.3	PPIE	LCK	SLC24A1
IL15	EIF4E	ATP13A2	WDR60	SOAT1
GABRP	GADD45GIP1	SELRC1	GABBR1	SLC16A4
CYP4F11	GNPTAB	DDA1	SCML2	CSPG4
EDDM3A	ENOX1	SLC17A9	RABGAP1	RAD51AP1
CASS4	PCGF3	LRFN4	KLRB1	TREM1
SLFN12	DDX18	MAP3K7	ATP10A	TLE3
RBL1	MRPS2	ZMIZ2	CARD8	MSH5
PPBP	TUB	ATP2A2	PLEKHF2	EPHA2
SERPINI2	ZNF14	RPP40	TRANK1	RBMS2P1
NSUN7	ENTPD3	CEBPG	STON1	ZSCAN16
FCER1A	VEZT	CHTOP	CTSW	HOXC6
MLLT10P1	AIFM1	METTL18	EFHC1	ZNF430
CD226	PAFAH1B1	ARL4D	EPS8L2	ASPM
RP1-181J22.1	MOXD1	BLNK	CTDP1	GPNMB
RP11-69I8.2	VGF	CILP	GAS2L1	ZNF224
TFEC	METTL21D	LARS2	MYO9B	DENND2D
HMGB1P3	BCRP7	GRIK5	ACYP2	ETV6
CCRL1	ZNF282	NAA35	ACTR3	HOXA5
SELE	PPIE	DTYMK	EDNRA	ZFP37
AL136115.1	XK	MECR	PILRA	FAP
RP11-488L18.4	OXSM	RP11-1280I22.1	DLC1	RPL37A
CCDC102B	GNL2	GSTZ1	ATP2B1	ETS1
LRAT	PSENEN	USP18	SMG6	ZBTB10
STATH	RNFT2	DUX4L9	TSPAN12	ZNF79
TIGD6	MIA3	REEP2	HDAC7	LILRB1
SPATA1	WDR70	NUP43	PICALM	CDKAL1
ZNF257	TMEM9B	ENDOG	PRKACA	APOL6
PRG4	TIMM17A	EPN1	PTPLA	UAP1L1
GYS2	ARIH1	KLHL25	ACE	RP2
UGT2B17	POLR1C	MLLT11	SOBP	KIAA1009

ADGB	SYT12	HSPA14	MAPKBP1	ZNF764
	USE1	TACC3	CD36	CDC45
	COQ6	NUDT4P1	PYROXD1	ERLIN2
	RFC5	CD24P4	MPDZ	IKZF1
	ASXL3	TIMM17A	NDRG2	TXNRD3
	LIMCH1	FKBP8	GZMH	MKI67
	ZNF415	CACNB3	FRAS1	PLK1
	ISCA1P6	FBXO46	NLRX1	RFWD3
	PANK3	HINFP	LMOD1	C5AR1
	NOC4L	COA1	PPP1R10	KIF15
	TRPC1	AC118138.2	RBM26	CSTA
	SMARCAL1	ASB6	ASXL1	CALML4
	TPM1	UBE2O	TIMM22	ZC3HAV1
	NARS2	MMP15	PRKCQ	LILRB2
	SPAG16	THOC2	NOTCH2	KCND1
	KCNH2	NIPBL	KLF13	ZBED4
	RP11-73M18.2	RBM34	CCBL1	ATP6V0A2
	DPP8	FKBP4	TRIM21	LILRA2
	ASIC2	SCAMP5	PSKH1	CENPA
	PLA2G15	FAM173A	ATP2C2	ZNF548
	SLC35C1	GPN2	MAP3K12	HIST1H2BH
	FAM49A	SHMT1	KAT6B	ITGA1
	KPNA1	FEN1	AP1S2	LOX
	HSPA9	SLC37A4	NR3C2	TRIM34
	PSEN2	CREB3L2	DOK3	TWIST1
	ESD	GPATCH3	CMAHP	FAM105A
	XPNPEP1	GADD45GIP1	MLYCD	IKZF2
	PCNXL2	DUT	EPAS1	NABP1
	BMPR2	CBFB	CTNNB1	KYNU
	SSSCA1	OGFOD1	OASL	MTF2
	LMO4	PARP2	OSBPL11	DEF6
	HOOK1	RANGAP1	KAT8	ZNF225
	SYNJ1	LMNB2	EMCN	ATAD2B
	CIR1	PRIM1	NBEAL2	THAP9
	TMEM74B	TMEM187	THSD4	MSR1
	WI2-1896O14.1	CCDC9	ARHGEF7	EYA4
	NUCB2	ARPP19	ESD	ASPN
	STOML2	SLC25A22	TMEM100	CENPQ
	RABGGTA	TESC	CTNNA1	RP11-492E3.1
	VARS	TMEM104	USP9X	DOK3
	PRR3	NOL9	RP11-64P12.8	KANSL1L
	KLK7	SH3BP1	JMJD1C	NCF1B
	ABCG4	GBAP1	TRGC2	ADAMTS5
	PDK2	RIT1	ZNF264	POLE2
	PPP6C	AL590369.1	ARHGEF15	CEP152
	KIAA0430	E2F3	CH25H	TMOD3

PPP2CA	ARPC4	AP3S2	AVIL
PSMA3	RRP7A	AOAH	ENPP1
TRAF5	HSPA13	DOK4	ABCC6P2
MED7	PITPNM1	LPAR1	PHEX
RAB40C	ZNF428	PDHA1	COL8A2
MRTO4	SUV39H1	RIPK1	OAS2
GLRX	COL1A1	LINC00312	HOXC10
POMP	TTI2	LPCAT3	S100A2
ZNF544	PLCB3	ACACB	HOXA7
UCHL5	DENR	UBB	RP11-340I6.6
RASA1	CCDC64	RETSAT	PRKAA1
FZR1	CEP85	SP100	CARD10
MAP3K9	MFSD12	PIP5K1B	PDE3A
ARFGEF2	METTL21B	PTGER1	TGFB2
FDX1P1	ABCB6	TBC1D1	MICB
DCTN4	ALG12	ZNF467	F2RL1
GAS7	RBKS	TRAF6	BORA
LRRC6	SBNO2	PELI2	NCAPG
FASTKD3	C1QTNF3	KCNMB1	LILRB4
GAPVD1	AKAP1	NYNRIN	PLAC8
PCLO	LYPD3	NRN1	MR1
SLC25A17	LEF1	MAOA	NDC80
TMEM120B	HIP1R	KIAA0556	ZNF551
PDIA2	ELK1	GSTM3	STC1
OBSL1	IL13RA1	NCF1B	SHCBP1
AC004941.5	TIMM44	RECK	MMP7
METTL22	CEND1	SLCO4A1	CPS1
RPRD1A	LSM12P1	PLA2G10	MCPH1
DENR	KCTD5	GCOM1	FUT4
STXBP6	DNAJC10	PLA2G4C	PRKCDBP
MRPL11	DUS2L	S1PR1	MTHFSD
RNF146	RRP12	TOX4	DDO
RABL2B	EFNA5	ZCCHC2	TTC26
MED4	ABCD1	FERMT2	PLK4
ZKSCAN1	SERPINI1	ZNF148	BNC2
SRPK2	TGFA	NHEJ1	UBD
NEU1	SLC35C1	TBC1D22A	ALPK3
PREP	MNAT1	POMT1	GALNT3
TM6SF1	BYSL	TJP2	ERAP1
RANGAP1	MST1R	SORBS1	KIF11
FBXO17	BCRP7	SEC63	IRAK4
F12	MTFR1	PPP2R5B	CCL5
ACOX3	LRRC14	PTN	TLR1
ALDH18A1	REST	RTP4	HOXD10
ATRX	AC007229.3	STAT6	GOLGA2P5
PDZD7	WDR4	TM6SF1	TANK

CDC27	FSCN1	SKAP2	SPICE1
CRADD	FAH	CD247	PI3
ADD2	ME2	MCMBP	PIK3CG
NOV	COPS7B	MTRR	TRIM5
DCAF4	FAIM	PDZRN3	KIF20B
CDC7	NHP2P2	SLC24A3	KIF20A
TMEM242	DGUOK	SDPR	LEFTY2
MFSD2B	ARMC6	QSOX1	CBLL1
COL5A2	GEMIN4	CPM	TGIF2
EGLN2	DSG2	ADAMTS1	STAM2
FRAS1	MED13	WDFY3	SOCS2
FZD3	B3GNT4	SSPN	ZNF701
NUP133	POLG2	STARD8	CDCP1
HPS6	RABEP2	CLCN6	TNFSF4
ICAM5	NACA	AHI1	HAS2
AC145291.1	SHMT2	SOX13	RNASE2
ZW10	LGI2	SYNE2	XRCC4
RPUSD2	ASF1B	PARP3	SIGLEC9
OGFOD1	FAF2	TPP1	TAF5L
LPHN2	PRR3	ANPEP	ELAC1
HSPA14	LPGAT1	DHRS9	LRP5
ST6GALNAC5	TMEM206	ATP1B3	GSTCD
PRPS2	CHD7	CYTH3	USP53
GSK3B	SNAPC1	BMP4	CLEC7A
AGPAT4	SMCR7L	EOGT	TRAIP
AMIGO2	RRP1	SFI1	CSF2RA
AC002398.9	COX15	ACADS	PROCR
BDH1	SMARCC1	IL11RA	S100A3
VDAC1P1	CDC73	KCTD9	TAF1A
CHCHD7	CDYL	KANK3	NCF4
FOSB	NCSTN	LILRB3	CLEC4A
NMRK1	BIK	PRKCE	RP11-357H14.19
GPR85	RP11-227J5.3	HLA-F	SIGLEC7
LSM4	RBM38	WDR48	PTBP3
SH3GLB2	SMG9	KCTD15	RAD51
ANKS1B	PRSS3P2	FGFR1	POC1B-GALNT4
PPID	ZNF133	HPGDS	CLEC4E
LRRC59	MET	CYFIP2	ERAP2
ALG6	MYBL2	PTK2B	SNTB1
NPY1R	PRB1	VLDLR	DPEP2
SRD5A1	GMDS	ZNF451	PPP2R3B
OSTF1	ADAM8	SIRT3	NEB
UTP14A	PPAP2C	RP11-710M11.1	ZNF85
PPA2	ADAT1	PTGIR	TLR3
PPARGC1A	PHC2	DDX19B	IGHA2
AKIP1	MGA	EIF4A1	CSF2RB

RABEP1	RAD17	RBM7	FPR3
ZDHHC4	FANCC	FAM190B	TLR7
UBE3B	SAYSD1	PTPRS	MDM1
GEMIN4	AEN	KLHL3	HELLS
PSMG1	CCL22	CASP1	HHEX
ANXA7	DUSP10	MTRF1L	NAT1
SPATA5L1	WDR43	MMRN2	SLAMF8
NTHL1	OVOL2	ZBTB40	VAX2
BCL2L13	HDHD3	PRKCH	ENO3
ZNF593	KCNS1	DAPK2	TTK
GNG4	TGIF1	HSPBAP1	HPSE
RAD23A	NDUFS7	SCML1	AIM2
DUSP2	BPIFA1	TNKS	RUNX3
MLX	RP3-334F4.1	MTUS1	ZIC1
RP11-164O23.5	PMS2CL	FBXL7	LRRC48
TEX264	TFB1M	CXCR1	ST8SIA4
DDX49	SMC3	EVC	TFG
TM9SF4	PDK1	PNMA2	LIPG
THEM6	FAM46C	CXCL3	HS3ST3B1
THOP1	UCK2	DIXDC1	DNMT3B
EIF4E2	PLA2G7	LRRC16A	PCDHB2
FOXK2	RNF141	FOXO3B	CNGA3
B3GNT2	GPX7	STX5	EXO1
PSMD9	PDE6G	EBLN2	FCN3
ULK2	TFAP2C	HNF1B	ZFX
PRDM10	ELMO3	NKG7	COL4A6
MOGS	CXCL14	PPP2CB	CHRNA9
CACNA1B	FUT3	OLR1	HMMR
MRPS15	RELB	MYL6	HEATR3
BLMH	UTP20	DES	KCTD14
SPATS2L	SMO	PTCH1	FLVCR2
STX6	SREBF1	BAHCC1	EFNA4
HDHD3	TRMT2B	PHLPP2	TTC30A
NCAPH2	PRPF4	DDX46	OIP5
THSD7A	GCAT	FSTL1	CLEC5A
AC007560.1	ATG12	NAP1L1	DSC2
NHP2P2	AHCTF1	ATG7	AURKB
ERCC2	SEMA7A	WASF3	ITGB8
NKAIN1	SLC35D1	SLIT3	GPR65
SLITRK3	HTATIP2	AP2A2	HOXA3
COMMD10	ESRRA	PITPNB	NOP14-AS1
DET1	NUDT1	CDK11B	ZNF334
MYO5A	PRC1	RABL2B	COL8A1
RPP25	HLA-DOB	TK2	TNFRSF11B
USP19	LACTB2	ITPR1	SHOX2
ATG7	KCNN4	CORO7	HOXA10

GPC1	AZGP1P1	EIF2S2	JHDM1D
AVL9	NPM3	TPRA1	TMEM48
CASK	XYLT2	TMED5	BCL2A1
MNAT1	HAUS3	TUBA4B	DLGAP5
DBC1	AC068533.7	COL4A5	IL1R2
PTPRA	SMN2	RALB	ARSE
TUBGCP2	SLC25A17	SHROOM2	RP11-678B3.2
PGK1	MED20	MPP5	SIM2
RGS4	SNX5	ACSL4	LY75
MPP3	TNXB	LRRFIP1	ATAD5
NREP	MAPKAPK5	CMKLR1	ENPEP
SLC16A2	APOL6	OLFML1	IGSF6
NUPL2	NCAPD2	FRAT1	MAP3K8
AMIGO3	NUPL2	ATXN7	NOX4
MED21	TCOF1	DYNC2LI1	HOXA1
PCBP3	SEMA4F	APBB1	FST
SLC25A23	PTDSS2	RPS6KA5	SOCS1
UQCC	NCAPG2	CTF1	BRIP1
MEMO1P1	BID	PRDM2	HIST1H3D
KCNMA1	ARF3	ACVR2A	PEX13
CDK14	MMP14	UBE2B	TNFSF8
HSPA4	PRPS1	KDM5D	RP11-473I1.9
BPGM	RRM2	CLEC1A	AZGP1
IGSF3	HIST1H2AI	PTPN13	KIF14
B3GALT2	FAP	CAMK2G	BUB1
PIGK	NRSN2	RNF146	HOXD11
FAM65B	DEF6	HIPK2	SIX1
RAB1A	CD180	COTL1	MIR548N
POLR3F	THUMPD2	ARGLU1	PYGO1
PELO	NKRF	BMPR1A	ARSJ
TIMM23B	TMPRSS4	PTRF	MEOX2
ATP2B3	XAGE1B	LILRB1	CCDC15
SLC39A6	CSTF3	ZNF83	CARD9
BAD	TBL1XR1	COL21A1	SIAH1
TKT	NOLC1	PRPF38B	CCL20
OCA2	ITGB4	RAB11FIP3	CCNJ
NFS1	ZNF512B	CDKN2AIP	ROR1
SNN	SURF2	CTB-89H12.4	CELSR1
NUDT18	PRRG4	DIP2C	GALK1
ZNF329	APEX2	MUSTN1	ADAMTS6
TRMT12	NFKBIL1	RGN	TEAD4
TBCD	CCNF	PRKAB1	CHEK1
TAC1	EPS8L1	REL	ANPEP
STAU2	CD79A	DPEP2	CYTIP
SLC35A2	TYMS	NEIL1	FABP4
DCAF8	ERGIC2	ZCCHC14	EMR2

POPDC3	CIDEC	TMEM180	COL15A1
RASL11B	BBC3	FBXL15	EN1
PEX3	AC145291.1	PLA2G15	AC112502.1
ASMTL	WNT10B	HNRNPH3	PSTPIP2
CSNK2A1	COG2	EHD3	NPHP1
CYB5B	FUT8	RP11-20I23.8	CBX8
NFKBIE	AC004941.5	KIAA0040	IL18
GFRA2	WDR18	SLC16A2	BST1
STS	GTPBP8	CTIF	UROD
POLR1E	DPYSL3	DNAAF1	GNLY
ENTPD5	RP1-40E16.8	ITGB1BP1	TTF2
NDUFS7	AP001055.7	KLHL26	DEPDC1
STAT1	ILF3	SLCO2B1	ITGBL1
ZBTB24	RPL10L	GNAI1	CCDC102B
MVD	ICA1	JMJD7-PLA2G4B	CNTLN
CYB5R1	POLA2	PDE2A	RARRES1
GLT8D1	CXADR	PTAFR	CXCL3
TMEM101	KIF3C	GJA5	CDC6
DYNC2LI1	YARS2	MEIS1	ANXA2P1
HGSNAT	F2RL1	ACOX2	AC008103.5
SLC22A18	FLAD1	IL3RA	RP11-405O10.2
MRM1	ZGPAT	ITGAL	MYB
OPN3	DLEU1	KCNJ5	GLI2
HDLBP	RPS6KB1	ACBD4	NOD2
RABIF	PTPN7	ATN1	IL15
DDA1	HPX	MECP2	EN2
CBFA2T3	MMD	ZNF767	FAM46C
OGT	ZNF444	ST7	IRX5
EIF5A2	TEX261	RENBP	MAK
MPPED2	NUP210	MSANTD2	CDC25C
ASL	GPX2	KLRC4-KLRK1	FLT4
SLC39A9	TELO2	PLA2G2A	PHC3
ATRNL1	DLAT	RBBP6	ERCC6L
LIMD2	SIPA1L3	UBTD1	CD72
CARTPT	PANX1	CD3E	CDKN2B
ANKH	MIS18A	CIR1	ST14
ABL2	TNFAIP6	TMEM74B	RP11-84C10.2
UBE2W	NNAT	CD33	CENPI
FAM86DP	NTHL1	RSAD2	CEP55
MRP63	TEAD3	ZBTB48	TFEC
SRSF3	CLSTN3	ATF6	ST20
FAM175B	OSGEPL1	BICD2	RBL1
UQCRC2	RHOD	ALOX5	ITGAL
MCM2	RTEL1-TNFRSF6B	TRAPPC11	HOXD3
RIC8B	ITGB7	SIVA1	KIF23
MTMR2	GRHL2	PIGZ	RP11-313D6.4

SLC16A6	RPS10P2	ELF2	CD36
SGPP1	SLC33A1	GALC	ARSB
MFN1	BAIAP3	VILL	APOBEC3B
DCTD	TRAF2	IRF5	PRRG4
WHSC1	RNF43	PPP1R7	HS3ST3A1
BPHL	MOK	MATN2	NKX3-2
PTPRF	DLK2	RSU1	NEIL3
MAPK8IP3	MOB1A	KIF21B	IRAK3
MKRN2	STK16	NID1	HIST1H2AE
GSTZ1	SLC38A10	ADAMTSL4	TLR8
FBXO9	BTNL3	DOCK10	MCM10
RIT2	WNT5A	RASA4CP	POPDC2
PIAS2	KIF2C	GOLGA1	WDR76
LZTFL1	RORC	DENND1C	MPZL2
B4GALT6	GGPS1	HEXA	OLFML1
DLAT	LRRC15	FAM184A	CTD-2224J9.4
CWF19L1	ZNF552	ARHGAP4	LILRA1
PTPLAD1	GALNT14	NELL2	PPBP
FARSA	NAP1L4	PDE4B	WDR78
TPRKB	ZNF768	MEGF6	ATP8B4
FEN1	NOP16	TBXA2R	HOXA9
GEMIN6	POU5F1P4	REM1	HESX1
ACOT8	SLC30A9	BMP6	SLFN12
RP11-29G8.3	LRRC20	ZDHHC17	POU3F4
TAF4B	SEL1L	HBG1	CLCN5
UBAP2L	ADORA2B	MME	NFE2L3
ATR	HSPA4	ZFYVE26	HOXA2
RP11-762H8.2	MRPS12	CD44	EGF
SLC35G2	NBN	PCYOX1	GALR1
HUWE1	AGK	PPARG	CD69
AP5M1	MYO19	MLF1	ORC1
NPR2	FNDC3B	DMPK	ADAMDEC1
CYP2R1	UBE2Z	ASTE1	ASB9
PRCC	TRIP13	IL6R	CLEC2D
FABP3	GSDMB	ZBTB43	PIGC
KIAA1324	NEK4	AKAP17A	CXCL5
NUCB1	DCTN5	PTPN21	MAB21L1
PRC1	RMND5B	AL356740.1	PRDM5
DNAJC8	POT1	TFPI	PRIM2
APTX	ECT2	ACAP1	SPC25
CACNA1D	RAD51C	ZNF638	COL14A1
GCN1L1	UBE2W	NR2C1	RP11-769O8.3
GGH	TK1	TGM2	ZNF345
ARL8B	ZNF16	LINC00094	PARPBP
MTERFD2	CASP6	MOB3B	WDHD1
CACNA1E	RP3-406P24.1	ITGA7	CD163L1

KIF17	PPATP1	TBC1D12	CA3
ADCK2	KHSRP	IGSF6	OSR2
MEGF8	DCUN1D1	RP11-264B17.3	GZMA
ZNF134	MSH2	GLS	HIST1H2BG
GBAP1	AVPR1B	CASZ1	E2F8
ORC4	PUS1	SYT17	RP3-497J21.1
CGREF1	DHDDS	PRMT2	ESM1
AP1G1	PFAS	NUP214	CCDC81
TASP1	SLC39A6	ROBO3	KIF18A
ARFGEF1	LSM5	SLC9A8	NUDT13
DNAJB9	ZMYM3	PEX14	MGAM
MRPL2	DDX54	TLE1	NR5A2
RP13-608F4.6	KRI1	ANKRD28	GPR39
FBXW2	WRAP73	RIN3	AGTR1
FUT1	AC005943.5	PSMC2	PTPN22
SAE1	TXNL4A	MAN2A2	IGFBP1
PPIF	GALR3	ZNF136	CD1D
CFL1	TRPM2	MAP2K4	PCDHB8
TRAPPC2P1	KIAA0947	IGF1	MAGEA12
ABCF2	RP11-526L8.1	DET1	F2RL2
ANKLE2	RP4-669P10.16	SLC47A1	ITK
MARC2	THNSL2	PRDM11	CYP39A1
SPRYD7	GCLM	TICAM1	EBF2
RAD17	AQP5	AC080125.1	NSUN7
GNB5	AMMECR1	CASP9	AREG
RUVBL1	MED9	CDC42EP2	TRPM8
ARHGAP35	MYO1E	JUND	MTTP
ZNF510	MOSPD1	TSC22D2	MSTN
PSMA7	ESPL1	CCDC22	AGBL2
MYL5	MPZL1	TARDBP	RPE65
ICA1	PORCN	PECR	LINC00472
WDR25	UGGT1	PDE8A	PDZK1P1
SNX4	AC010170.1	KCNK3	HMGB1P3
RPL35A	DOM3Z	RAB21	BRCA2
ARC	PRR5-ARHGAP8	C5	ITGA4
ACAD8	SLC22A14	SLC35G2	TFAP2B
ATP8A2	B4GALT4	PARP8	HIST1H2AG
GRB14	BDH1	RREB1	SKA1
DNAJC16	CD209	IKBKB	MMP1
MLF1	TUFM	NOD1	PRDM13
TRHDE	PDLIM7	FOXJ2	VNN1
ODZ1	FER1L4	ATXN1	IL7
DNAJA4	PDXDC2P	ANKHD1	CXCL6
MGAT2	HMGB3P1	HGSNAT	CXCL11
DBNDD1	EPRS	TRIB1	TMEM156
DHRS7	SDS	TMEM168	GPR18

PDIA3	FBXO2	ATP6V0A1	ARHGAP11A
NDUFB4	AD000090.2	TMEM183A	SLC34A2
NSDHL	NR2F6	ATP9B	TIGD6
MSH3	RP11-263K19.6	EHBP1L1	GATA6
SIK3	ELL2P1	KDM6B	IGJ
PDCD11	PC	ARHGAP5	AC005754.1
EFTUD1	ME1	FES	TPH1
WRAP53	GPC1	FGFR4	MMP10
TFB2M	GSR	EDNRB	MMRN1
ZWINT	SLC6A10P	FBXO3	HNF4G
PCSK1	URB2	ERAP2	VGLL3
FGF14	UBE3B	PRUNE2	FOXD3
CDC37L1	LPPR2	SYNGR1	PLEKHS1
IMPAD1	PAQR4	GUCY1B3	PITX2
WDYHV1	TRABD	PIK3C2A	RP11-116O18.1
TFRC	TWF1P1	AC141846.4-001	
FAM188A	CYHR1	IHH	
RASGRF1	WDR55	NFAT5	
FAM45B	ZDHHC13	PGM5	
WDR67	CNKSR1	TMOD1	
ATP7B	HIST1H4J	DHRS12	
ALKBH4	PICK1	PLA1A	
MAPK10	RALGAPB	HIVEP2	
HNRNPC	CTSL2	RANBP10	
ACOT1	ACVR2B	AKAP7	
ZNF711	JHDM1D	AL603926.1	
PPRC1	CCNB2	KRT4	
FAM57A	LRRC61	DHX58	
GLMN	JRKL	KB-1896H10.1	
GFPT1	RRNAD1	MTHFR	
SDF4	KPTN	COX4I1	
NDRG3	PPCDC	FAR2	
GRM1	DIMT1	MOSPD2	
PRPH2	ASPN	DMD	
PPP1R2P3	SLC25A14	MAP3K14	
TXNL4A	TRIM62	BAIAP2	
LIG4	PDE9A	FDFT1	
SENP3-EIF4A1	TUBGCP4	MGAT3	
CLCN4	LIG1	KDSR	
SLC7A1	FICD	LONRF3	
CAMK4	USP48	GADD45G	
GLRX3	OSBPL3	CHST7	
FAM110B	NLE1	SPATA2	
RRP7A	CUEDC1	FUT1	
POLR3K	FGFBP1	EXOC3	
GEMIN7	PDGFRL	GALNT12	

RANBP9	RP5-886K2.3	GNLY
DERL1	RHBG	SRGAP2
CNR1	STMN1	SH3BP2
ACTL6B	TBKBP1	PRKD1
PRKAB1	SPSB1	COL13A1
DCAF13	MRPL19	SOCS2
TBC1D19	PPP5C	GSPT1
STXBP5L	RFC2	DUSP2
ATP6V1G1	GATM	SGK3
AKAP6	ZMAT5	EXOC2
DEGS1	RIN1	RP11-96D1.10
CEP192	PODNL1	DNALI1
WDR18	CARS	ZFYVE9
TBL1XR1	AGA	CAB39L
ADCY3	PDGFD	HSDL2
WBP4	POLR3D	CTSG
VTI1B	MYBPC3	NBPF12
DBP	G3BP2	LRRC36
MGMT	FKBP10	PHKB
CACNB2	NDUFS1	CHPT1
PFAS	CFL1	ZBTB20
ERO1L	CRLF1	MAP3K8
ARHGEF4	SRPK3	RP11-574K11.16
ITGA9	SDAD1	REV1
ST8SIA5	CRIPT	SRSF8
PRKCI	RINT1	IL33
MAPK8IP1	A4GALT	GFOD2
PDE1A	POLR3C	GNL1
FBXO28	CUL5	RABGAP1L
ADAM22	SIRT5	ABCA5
PVALB	TTC22	EPB41L4A
RP11-390F4.3	AC079780.3	NR0B2
MAPK8	MLH3	PHACTR2
KCNJ9	SLC38A7	KIAA0513
RBM8A	MRE11A	PDGFB
GDE1	APH1A	HSPB2
FAM190B	METRN	USP7
PRMT7	ERCC2	GRK6
CELF1	DOHH	DUSP8
CA10	CA12	MAP3K6
SUMO1	TPX2	PLEKHB1
RNGTT	RNF40	PEG10
COLQ	MEX3D	CSF3
PAK1	PCGF3	NEDD9
GRM5	CCNG2	CEP104
ORC2	RNF219	SNHG14

PTPRR	MGAT5	FZD7
MBD4	CSRNP2	THRB
FABP6	HMX1	PPP3CC
NDUFA10	SRCAP	TNIK
BOLA1	EGFR	SPTBN1
TM9SF1	CST4	CLDN11
ENTPD6	CDK1	CLDN18
CYP26B1	E2F1	TRPV2
SEL1L	BMP1	RUFY3
SNTG1	GPR176	ADTRP
RP11-18A3.4	CDC27	NEDD4
DGUOK	PBX2P1	MTERFD2
NNT	TKT	RPS28
ME2	BPHL	PTH1R
TRIM32	MED14	TMPRSS2
DHDDS	PARD6A	NUDT18
LRRC41	ADA	CDC14B
ZNF304	GOLGA2	SYNJ1
IFT27	BOLA1	CA4
CHML	MARK2	SF1
SRPRB	MUC5B	SLC25A12
RNF14	SFXN1	PLK1S1
PPP2R5D	PMAIP1	AKAP12
ADAT1	BARD1	CST3
UBQLN2	CHERP	CD53
SMURF1	NPPA	CAMP
POLE3	RWDD3	P2RY14
GRM7	CAMK1D	RFX2
DUS2L	CLEC11A	RAPGEF3
MAN1A1	TCF3	CDK10
MAGOH	ATP13A3	VAPB
RAD1	AK4P1	LRRC23
YWHAE	RP11-216M21.2	EXPH5
SCYL2	TPSG1	RAB36
PPP2R2B	TFAM	CHIA
LIN7C	GJB1	TNNC1
ZNF385D	CBX3	SREK1
CHRM3	MEAF6	DOK1
CDH3	DARS2	GPA33
SLC37A4	GDPD2	KLK10
WBP11	NADK	SMURF2
SLC37A1	ZNF195	GAS1
RRP9	SAP30	GSK3B
CHM	BRIX1	MYOZ1
FAM208B	NUP155	ZFPM2
EDC3	TMEM62	IKZF5

TTC9	AP4M1	GULP1
RNF7	AGGF1	LHX6
PGAP1	DCAF13	OLFM1
PIM2	ENC1	APOBR
SLC6A15	KRT15	KIDINS220
MAPRE3	EDEM3	POLR2L
GSTT2B	TMEM59L	STX4
TRIM17	KLC2	TPPP
SLC4A8	CSTF1	SMAD9
PNPLA4	SRPR	RP11-617F23.1
BTRC	TROAP	PDPN
TRO	F12	EHD1
NPM3	RCE1	ANK2
CDC5L	TPD52L1	PPP6R2
CACNB4	POP4	ARID4A
AC124309.1	AP1G1	DOCK6
CENPO	KCTD13	PIGQ
SORT1	STX1A	TACC1
SDF2L1	RPE	PGS1
CDK5R2	HSPD1	DAAM1
JMJD4	RBM19	RP5-874C20.3
GLS2	SOX9	ZNF14
FKBPL	NCLN	GJC1
CNIH4	SMG7	MSLN
RQCD1	ZNF93	SMARCE1
RRP12	PRR5	CENPC1P1
UBE2S	SLC27A5	DNASE1L3
COPA	KIAA0930	ST6GALNAC4
KIAA1549L	TP53	PHLDB1
ATF7IP2	CSTF2	HIRIP3
HSPA4L	GPATCH1	PIBF1
MREG	PDGFC	WIPF2
TIMM22	CPOX	EMP1
VLDLR	LLGL1	GPD1
CDC42EP3	SORL1	UPK3B
API5	TP53TG1	SACS
ZNF747	MFN1	CDO1
CACNA1H	RPL39L	BCAR3
PPP3R1	CCDC6	ID4
SMPD3	TNFRSF4	EFNB1
TPD52	RPL18	CLIC5
CDKL1	SRPX2	OSGIN2
TM7SF2	ZBED1	ENPP4
WDR26	NUSAP1	KIAA1109
DCUN1D4	CHRNA2	CYP20A1
ATP2B2	TCF7	CYP1A2

YRDC	IPPK	MYH11
TNFRSF21	MMP12	CCDC69
CACNB1	ENTPD6	ARNT
ANKRD6	ZNF354A	ZFYVE21
PDYN	PLA2G4A	LMO3
YIPF5	TPR	FRMD4A
GMCL1	GTF2H4	RASAL2
SBF1	KIAA1199	CSDC2
STK16	NETO2	P2RX1
ZFYVE21	GTF2H2B	NDRG4
CTD-3074O7.11	EFNA3	ATP6V0E1
ARL1	LRIF1	TRIB2
GRIA3	SMC6	FYN
RP3-406P24.1	CRCP	EXT1
SUPT3H	CHAC1	STXBP6
KBTBD4	NT5DC3	MFSD2B
ADK	KREMEN2	EPB41L5
PRR5	CHEK2	MUM1
SIVA1	GLRX5	THRA
UCK2	CALR	SAMSN1
RNF25	SLAMF7	RPH3AL
UBE2V2	ZKSCAN4	NEK9
PNOC	SRP54	WBP4
ZNF239	ZNRF4	TNIP1
EXOC2	PGM3	TPM1
OLA1	SPATA5L1	NEK11
INPP4A	AC126365.1	UBE2D2
GLRB	GART	NDUFAF4
SEC23IP	SEH1L	ARHGAP44
CARS	STMN3	THBS1
PROSER1	GRIP2	ANGPT1
ARFRP1	ARTN	ALOX15B
DGCR2	MYO7A	ZNF423
PIGCP1	NDUFAF5	RASGRP3
CHRFAM7A	SNAPC2	MYH14
RPL39L	EPHB3	KLHL29
SKAP2	TRAPPC4	MOCS1
SNX2	CDCP1	MSRB2
PAICSP4	SOX10	PER2
PREPL	JOSD1	GPRIN2
AMACR	CTPS2	FMO3
ST3GAL2	NIP7	CAP2
NUDCD3	INTS1	PPM1D
BCLAF1	RP3-522P13.2	ZFHX3
DOK4	UQCC	TCF4
JOSD1	RGS4	FAM125B

DHX8	PRMT3	OTUD3
ACTBP9	MKS1	TSPAN32
RP11-57H14.4	P2RY6	ARHGEF28
AP001055.7	ST3GAL4	PAIP2B
PCDH1	CKAP2	PARD6B
TAF11	NFKBIB	ADAMTS8
PDE6D	MARCKS	PLA2G6
SLC2A3	PEX7	CDKN2B
SPEF1	TUT1	ZC4H2
IL1RAPL1	MTG1	PHLDA1
KLHDC8A	NAA40	SPG7
MRPL4	RPRD1A	PRKAR1A
DDX19B	TXNRD3	EHD4
SLC30A9	ZFP91	RBMS2P1
AL136419.6	OSGIN1	ABCG1
ATP5F1	COL11A2	HOXA3
MTOR	BLM	SMARCA2
TFCP2	ADCK2	SLC15A2
BRIX1	RP11-29H23.6	ING3
PRPF4	CTA-204B4.6	TM7SF3
PIGF	MAD1L1	FARP2
ZNF407	PAK2	PTGS1
B3GNTL1	RASAL1	LUC7L3
PHF20L1	SLC5A5	RBM25
COX6B1P3	CEACAM3	ARRB1
FGFR1OP	TAC3	COPS2
CDH12	RP11-243J18.3	PCIF1
CLUAP1	PEX6	TEAD4
TRIM24	LARP1	ARHGAP10
SIGMAR1	SHPK	FZD1
DCX	TBL3	GDPD3
PQBP1	CHRNB2	RP11-15J10.1
SDHAP3	TRAF3	ZNF226
SSTR2	MAPKAP1	RPL31
FAM5C	CTD-2089O24.1	METAP2
RNF128	SPAG4	ABR
SMARCE1	STRA6	TMCC1
SRD5A3	CD3EAP	SSFA2
LIMK1	KPNA4	TOPORS
FECH	APBA3	CLEC10A
PODXL2	QPCT	SIK2
RAB4A	JMJD4	ALPL
TTC33	SRD5A1	USP15
CNKSR2	B3GNT3	COL4A3
ACSL6	SCYL3	HSPB6
CKAP2	TSPAN5	CIZ1

UBXN2B	PIGN	ACAT1
TSR1	CIB2	TBX5
MSANTD3-TMEFF1	AGT	P2RY13
NUS1	LHB	CLIP2
SNX11	MLXIPL	RP11-382A20.3
OXCT2P1	ARHGAP1	PF4
COQ7	GALNT6	IL1R1
ADAM19	SLC22A18AS	FLVCR2
MAPKAP1	KIAA0317	RRAGC
MTMR12	CEP57	TTC33
FANCF	PAK1IP1	THAP1
HTR1E	SNPH	CDKN2D
FAM92A1	IGF2BP2	MLL
PASK	AHNAK2	PAFAH1B1
COQ2	GPSM2	IQCG
KDM8	KLK1	CLDN15
IFT81	P2RX5	NR4A3
CAND1	TNS4	KMO
TRAPPC11	TNFRSF13B	F2R
CLEC16A	PVT1	MECOM
HRH1	ALAD	EIF4A2
PPIP5K1	CELSR2	FAM178A
TP53TG3C	ZNF652	VAMP2
SLC16A8	CDC42BPA	RBP4
CHAF1B	C1GALT1	RAPGEF6
PDK1	STARD5	CCR2
NOVA1	TGM5	CAPN7
SCYL3	SF3B2	FGFR2
MBNL2	MAP2K6	RP11-411B6.6
TRIM27	CDCA3	SRSF3
ZC4H2	RPUSD2	BHLHE41
INPP5F	TMEM33	TBCEL
GRIK1	ADK	ZNF573
TRIM16	AC005822.1	STAT3
POLR3D	AOC4	NT5C2
BAP1	PRSS53	COX7B
BYSL	EXOSC2	AL021707.2
PTPN3	GLI1	THBD
PKD1	BIRC5	MBNL1
SYNCRIP	COQ7	SEPP1
YPEL1	FKBPL	ZHX3
PTCD2	CPD	ADCY6
OSBPL3	TDP1	RGPD5
EIF5A	AQP7P3	ABCC6
TRAPPC2	HTATSF1	PPP1R16B
SIRPAP1	FGL1	ZNF20

PYCRL	CMAS	GPRASP1
ARHGAP32	SYMPK	RETN
TRPV2	ASGR2	LINC00667
HAS1	KRT5	ACOX3
DCPS	UBE2D1	SLC16A5
PPIP5K2	DCAF4	STK10
SYT5	PDLIM4	FRZB
RAB3D	PRPF6	SETD4
TFR2	U2AF2	TRIM5
ATF2	PRSS22	MYL5
MRPS30	FJX1	PDSS2
ZNF804A	FGD1	DNAJB4
PRUNE	RP11-357H14.19	NIPAL3
LARP4B	AMHR2	GJA4
TMEM48	CHAF1A	GNG7
STYK1	AC007389.3	ZNF91
TYRO3	STAT1	PRRG1
OBFC1	DONSON	ARHGAP26
GULP1	DBR1	CCL14
FLOT1	NNT	LMF1
NLK	ZNF184	TAOK2
CDK12	PANK3	ANKFY1
RASAL1	RARG	UBE2I
ZDHHC13	PFKL	RAB8B
GPR21	TMEM5	AC092839.1
ABCC1	AURKA	CLK4
PTPRM	FDXR	HHEX
SLC30A5	MRPL35	GLB1L
PACSIN1	THAP3	PRKX
TMSB15B	LIPE	ETS1
MYO16	CLPB	FKTN
NETO2	ADRBK1	EPB41L3
PROSC	FGGY	FDX1
SQLE	FBXO41	SIGLEC7
PRKCG	EAF2	GIT2
HNRNPU	TFAP4	LUZP1
TIMM8B	CENPF	SNCAIP
RINT1	AC010336.1	FPR3
ZNF323	CDCA8	PDGFA
LGALS8	LANCL2	BNC2
GPN2	THG1L	NFATC2IP
DEDD	PCNT	LRRN3
ADAMTS8	ECE2	TOR1AIP1
TMED2	STX6	PHLPP1
UBE4B	TMEM249	ATP6V0A2
ZDHHC17	LTN1	MED4

NR4A3	SEMA6C	EGR3
MED20	UBIAD1	PIGG
GTPBP8	SQSTM1	SORBS3
APBB2	DNM1	PNMT
LHX6	PRODH2	RALGPS1
NAB2	OGFRL1	SHC3
SLC23A2	HEATR2	RP11-125K10.4
ENTPD4	PAK6	CDK9
TMEM53	RP1-142O9.1	NPHP4
FKBP11	ERI2	ZNF673
NF1	RECQL4	SEMA4A
DDX51	EFNB3	FNBP4
ARHGEF11	PHKA1	SWAP70
HSPB11	PSAT1	MAN1A1
CNPY2	TRMT61A	MATK
UHRF1BP1L	IGFALS	RIMS3
FAM47E	TXNL4B	FAM49A
CALB1	PVRL1	TBX3
PRR4	RGS14	GGA2
CRKL	FAM86DP	PCDH9
AFF3	RNASET2	MCTP1
COX6CP1	B3GNTL1	HMHA1
FEM1B	PRMT7	GPR171
LEPROTL1	AL133458.1	HOXB5
NAA50	WDR5B	CLEC4A
MRPS12	THOC6	CAMK1
DCAF10	RAD23A	PIK3C3
MAPK1	CTC-203F4.1	CCNL1
SLC31A1	CLDN3	APBB2
PSPC1	DUSP4	GSTK1
KLHL3	GPR68	MAPK11
PIGL	ATXN7L3B	SUPT7L
PIGB	GJB4	RP11-492E3.1
SPCS3	ZNF7	ARHGEF12
RP3-437C15.1	TBCD	FRY
CHCHD3	SIT1	ARHGEF2
RP11-77H9.2	AP3D1	NPR3
B3GALNT1	OPA1	INPP5B
DYX1C1-CCPG1	FBXL4	DCUN1D2
DIMT1	RHBDD3	HUWE1
GRAMD4	RP11-49C24.1	TMEM231
PVRL3	ISYNA1	MRPS14
GGCX	RBM12B-AS2	ALDH1A2
UBE2Q1	CYB5R1	NCOR1
PRKDC	PGLYRP4	PLCB2
RBM12	MRM1	SETD5

ZMAT5	ALOX12P2	TRAPPC10
EHBP1L1	HMBS	ABCG2
IFT52	PPP2R5D	PCOLCE2
ALG9	CYP2J2	UBXN8
NEURL	GRM4	SUGP2
HUNK	RPP25	VNN2
KCNS3	ENTPD3	RTN1
TUBGCP4	IKBKE	SPON1
TXNRD2	PAQR6	RASA3
KIAA1199	ZFP64	CCNT2
MAPK13	TEX30	DCP2
MED28	COIL	SGPP1
KIF26B	T	BMP2K
GUCY1A2	BDKRB2	TYMP
ARHGAP26	FGFR1OP	COL14A1
SMUG1	FANCF	SOD2
CNIH3	CXCL13	HLF
SIRT5	RRP9	HIPK1
MED8	CORO2A	SPI1
EXOG	DYRK2	RNF41
RP3-425P12.4	MELK	KCTD14
ZNF839	MC1R	PLA2G3
GSR	GAMT	MYCT1
ZKSCAN4	YKT6	GPR65
STRAP	SPATS2	WWC1
MYT1L	ELOVL5	COBL
EIF3H	RNF25	SLC22A17
DHRS11	AHDC1	RGS3
PRPF18	LAMP5	IL12RB1
RPN1	NOS1AP	SPTAN1
TUBGCP3	ZNF576	TLR1
CYP2E1	AP001324.1	BZRAP1
ATP5S	TGFBRAP1	NTM
SPA17	CHD1L	TXNL1
BLVRA	NCDN	CTC-428G20.3
PENK	PCYT2	NCK1
CCDC22	PAQR3	PDE5A
EIF3J	POLE	SLC12A4
POP1	HIST1H2BH	PCSK7
UBXN8	MAP6D1	ZFP36L2
MCHR1	CDH22	NUPL1
ARL10	DCLRE1A	INPP4A
DGKE	MLF1IP	DDX3Y
KATNB1	APBA2	MXRA7
PRPF31	KRT17	PTPRD
IKBKAP	NDE1	EMR1

TUFM	ADAM28	LRCH3
DDX28	CERS6	HIC1
RNF34	ATF5	KCNMA1
CUL3	MAGOHB	RP11-510M2.10
GTF2H4	GTPBP10	OGG1
USP46	PLA2G12A	USP25
AP1S1	MRPS30	MGP
TSFM	CNOT1	RP11-1136G11.7
RP11-77K12.7	KCNN1	RAB28
SCPEP1	CEP70	DNAJC8
AC021066.1	CLTCL1	SLC16A4
DIDO1	RPL27AP	ZNF589
FOSL2	COL7A1	RAB33B
RNF8	TBL1X	BTRC
RASGRP2	ACAA2	CHN2
AFF2	KCTD17	AOX1
ULBP2	GPT	ITGA10
SEC63	PLA2G2D	CLEC2B
BCAT1	MDFI	NIT1
SCN8A	NCBP1	ST13
MSH2	SLC5A2	TP73-AS1
CDADC1	ATAT1	ITIH4
ZNF682	CNR2	RBM8A
ADRA2A	LIMD2	DENND2A
TXLNG	RQCD1	TUB
BDNF	CLCN3	SLC4A4
P4HB	BAZ1B	IRAK3
RABL3	PRB4	DUSP26
RNF103	FANCE	SCN7A
QPCT	AC127496.1	HABP4
HRASLS	PHF16	PZP
ALDH1B1	SRPK2	PDE4D
SAR1A	DSCAM	SH3GLB2
SUSD4	FAM208B	ARNT2
CA12	MSH6	AQR
HIVEP1	OPLAH	SIPA1L1
ANKRD34C	PAK3	TUBG2
RP3-468K3.1	EXTL3	ZNF248
ACTC1	PASK	CASQ2
DRP2	KLK12	ABAT
CDK8	CUL9	ATP5F1
POLR3C	TMEM30B	CNTRL
SREK1IP1	CLDN10	GRSF1
NRBF2	CA9	SIGLEC5
RP5-1028K7.3	OVOL1	SLC2A9
ACTR8	MAGED4B	MARCH3

GINS1	PLEKHG6	DAB2
DGKZ	AC007967.3	RP5-1052I5.1
COX15	ZNF821	FAS
CERS6	VWA1	TRIM13
SLIT3	GRPR	HDAC4
POTEKP	MUC3A	TRAF3IP3
PIK3CA	MIA	KIAA0232
PIP4K2B	SPTB	RASGRF1
ELOVL6	RP3-425P12.4	PIEZO2
TRH	TMSB15B	DTWD1
PEX5	TM9SF1	NEK1
CREM	TP53BP1	AR
DNAJC10	RHOBTB3	GPR27
DUSP3	METTL8	ARHGEF10
PGA3	EGLN1	CALML4
TIPRL	POLG	PRDM12
FLAD1	ZNF8	GAB1
ATP6V1D	ORC6	MAMLD1
WDR4	ING1	IL18RAP
GRIA1	WHSC1	TRAK1
GFM1	RP11-407N17.3	SLC30A4
RPP30	BCL2L13	XCL2
PA2G4	AIM2	CUX1
PDE4D	IKZF2	TMEM242
UMPS	ARHGAP33	CCDC48
NDUFC1	SMAD5	EPB41L1
CEP72	RP11-240D10.2	GSN
MAD1L1	HCFC1	HSPA1L
ARMCX5	GFER	RORA
ST3GAL6	NCAPH2	PROSC
PLCB4	ACTC1	CENPT
STMN1	SMOX	HIVEP3
DACT1	ZNF646	PARP6
IMPACT	RAB3A	METTL3
UGGT1	STOML1	PRKCB
SLC9A1	TFDP1	RDX
PFKL	FRS3	CEP63
NLE1	IRF4	AC007362.1
RIC3	NPFFR1	RP11-98J23.2
RGS14	CBS	TMEM143
BRF2	SPTBN5	GNPTAB
CUL4B	YIPF2	TNFRSF10D
PHLDA2	NUP62	BBS4
FAM182B	DHX34	KALRN
CRYBB2	PSD3	ARPC5
TEX30	ZMYM1	FAT4

KLHL22	LMNB1	WIPF1
SCRN3	SMARCD1	C8B
TRIB1	MTA1	GPR4
TBCEL	C1QL1	NDUFC1
SEC22A	RHBDF1	CD300C
CPD	AGFG1	RHOQ
HBQ1	NDUFB2	GSTT1
DSCR3	FAM64A	MAGI2
KCNJ3	GTF3C2	SYT11
CTSL2	PPP2R3A	SLC2A6
DESI1	TTPAL	SRRM2
CRHR1	TTC39A	PIAS2
NCLN	LGR4	SURF1
PAFAH1B2	MYOG	ARHGAP19
METTL2B	DENND2D	MPP3
CNTNAP2	FXN	IL6ST
MRS2	GLMN	TBC1D15
KCNB1	RABIF	NRF1
BCAP29	PEX10	CD200
CRYBB1	HIST1H1D	RUNX1T1
DZANK1	ZNF692	BAG5
BAG5	RAB7L1	SYDE1
PTPN4	HPSE	sept-04
JAK3	TFCP2	PCDHA1
NRAS	CCDC99	INPP5A
MOCS2	CCNE1	ERMAP
RNF6	KRT6B	IKZF4
DYRK2	UBFD1	GALK2
ZNF7	CD70	SLC44A1
DAK	HOXC11	ACSS3
MARK2	HHLA1	CERS4
TOX	SGK2	GNA14
CANT1	CD1A	FAM149B1
SCN5A	PTCD2	VEZT
HSPA13	THOP1	TBX4
PRKAA2	ATPAF2	EIF5
ACTN2	PTBP3	PTPRO
IFT57	RNF208	ELAVL1
BDKRB2	CSNK1G3	RP11-195E2.1
LAMB3	DTNB	RAPGEF2
KCMF1	TPD52	CTDSP2
ABHD10	NOC2L	HDC
FBXO16	PYCRL	CEP68
FHIT	KCTD20	GSTT2B
CDC40	RPAP2	ROR1
DNAJC6	KHDRBS1	USP19

CCNA1	GDF2	SPOP
SNIP1	ZNF259P1	FBXO21
CSTF1	MYCL1	CCDC68
GNAL	PSRC1	PNPLA4
NAV3	BAX	USP34
DSCC1	CENPN	PFKFB2
DHODH	NUFIP1P	TSPYL1
SLC12A8	HPS1	ANXA7
RAP2B	ATF7	sept-11
MMACHC	ATP10B	ZAP70
ZFPL1	CDK2	TLR3
SEC62	HDLBP	SATB1
POMGNT1	CDC20	VPS13A
DNAJC17	EDC3	IQSEC3
TRIP13	SOX12	RWDD2A
BBS7	ARSF	ZBTB38
ARPC4	GNB3	PTPRE
BRSK2	GINS2	DOT1L
H2AFY	SOGA2	ELK3
NAA38	DDX49	VPS13D
CRYBB2P1	MB	IFT57
NLGN1	RNF187	CACNG4
ABHD6	SDCCAG3	REXO2
MRPL44	ZDHHC18	PINLYP
ENY2	ACVR1B	NRCAM
QTRTD1	CENPM	HOXB6
ARNTL2	ARFRP1	MN1
SPATA7	PDSS1	PRKD3
OSTM1	URI1	HGF
POM121	CACTIN	ADRB1
RAB8B	RNF39	SLC24A1
NRP1	H6PD	CTD-2514K5.2
CCR10	CDH1	AFF4
IFRD2	DNAJC3	NOMO3
PLCL2	FOXM1	RRN3P1
FRY	ATP11B	ARHGAP6
SEH1L	LIMK1	CAND2
DCLRE1A	TDO2	MCL1
ZKSCAN5	YBX2	GPER
AP4M1	BCL7A	ZNF302
IFNAR1	FCHO1	PSTPIP1
PTPLA	HJURP	NPC1
RAB14	SETDB1	DSTYK
MBD1	SV2A	sept-07
DLL3	KPNA1	SIRT2
RCHY1	B9D1	TDRD3

ZNF222	TLN2	GMPR
CA4	ATF2	HECTD4
GLCE	IDE	ITK
ARMCX4	TLE3	COLEC11
HSPD1	PEX1	PLEKHA2
PXMP4	KIAA1644	DTNA
KIF22	LGALS8	N4BP2L1
DGKI	CEP250	URM1
CHRD	FADS2	GPM6B
EPB41L4B	FAM168B	GNAZ
ARHGAP19	NECAB2	LEPR
NNMT	LEPREL4	AADAC
B9D1	ZC3H3	AGPAT4
RNASEH2B	RNF24	KB-1460A1.5
PTPRG	SPTBN4	CPB2
MED6	MAPK1IP1L	RP11-1319K7.1
HSPB3	PDZD7	SLC11A1
LINC00667	HIF1AN	CAPZA2
SLC52A2	EPHX3	MAP1LC3B
BCAS4	ACACA	AKAP10
NOS2	RP11-445H22.3	RIC8B
ZNF238	APLP1	MED23
GMEB1	THBS4	PCSK5
SSR3	INSR	FRYL
FBXO3	GNB5	UST
FGF9	PAOX	PTPRB
PANX1	SH3D21	EPHX2
PEX1	ELL3	PLN
DUSP5	PTCRA	RNF125
TDP1	OSBPL10	GK
CHAF1A	RP11-831H9.16	PDCD2
ABCB8	PIGO	EPHB6
SAA4	POLD3	TSNAXIP1
DGKQ	DUS4L	CDON
PRSS3P1	MRTO4	NFKB2
DLGAP1	MAT1A	NUDT4
ANKRD26	TCAP	KIFC3
CCNT2	KIF26B	RELN
KCNK7	ZDHHC4	ZDHHC11
MCF2	MAN1A2	IL16
RAD50	CTTN	RGS12
CEP70	RRBP1	ARL5A
CDHR2	FH	CBFA2T3
AP006222.2	SFRP4	WSB1
ARG2	CLDN9	LCAT
UBTF	BECN1	LGALS2

VPS53	PCSK1N	MBNL2
SLC6A7	ADRA2C	CA5B
GS1-164F24.1	RP11-22B23.1	ANO1
PLXNA1	PSME4	SLN
ACSL4	HOXD9	ZBED2
MAGI1	NAA50	ULK2
MRPL13	MAPKAPK5-AS1	VCL
INHA	GJB3	YTHDC2
PTENP1	IL13	MMP28
ERCC6	JARID2	SENP6
UST	TBX19	SOSTDC1
NUDT4	HAUS5	ZNF304
VPS33A	SMUG1	PTPN12
PDE8B	ASPH	MLLT10
CABYR	GALNT8	TMEM86B
GNS	GEMIN2	PKNOX2
RHBDL1	DSCC1	NR2F2
RP11-72I8.1	STRADA	TRAF3IP2
CSNK1D	ACTL8	HIP1
FLRT2	CD3G	ZNF329
SSBP2	OR1F1	SPG20
TAF12	VCY1B	ZEB1
HDAC9	OSMR	IFNAR1
FNBP4	MAD2L1	TMF1
GCLM	TMEM38B	SH3GL3
STAG3L4	SLC9A5	RP2
ZNF473	RSRC1	RAP2A
RNASEH2A	PEX16	RASL10A
FARS2	DDX6	HSPB7
GMDS	MOCOS	PRKY
CCNO	RBM4	RNH1
SSTR1	LRRC17	AC005013.1
VPS16	BNIP1	ESR1
GRP	MVD	PIK3CA
DNAH9	AMDHD2	MSR1
PRKCD	CBX5	LIMA1
PTH2R	MAP3K9	PIK3CG
SAFB	CHST3	KBTBD11
RUNX1T1	CYP11B2	ELMO2
SFTPD	MCCC2	CTC1
CHP1	LIN7C	SMAD2
ECT2	SEMA3C	PPP2R2D
AUNIP	PRKAB2	TBX21
ODF2	PGBD5	LRRC6
EML1	ATXN2L	ADPRH
PCIF1	PNMA3	ZNF548

KCNJ12	WDR62	FECH
NDUFB2	DDI2	BEX1
PDLIM7	RAB40B	PXMP4
ASGR1	UBE2G2	AKT3
GTF2H1	IMPACT	RBMS3
TSEN2	PACSIN3	CLASP2
TRIB3	CUL7	MCM9
RFC2	PDCD1	RPS20P21
BCL9	BRF2	UTRN
PRR7	FOXRED2	CIITA
ZXDB	CBX4	HIC2
AC006011.4	GJD2	MAST4
PTGER3	MAPK12	CASP4
BNIP1	ODZ4	TRIM66
BAX	CAMK2A	S100A3
TFIP11	ZNF35	GTF3C5
SRMP1	TJP3	NPR2
TMEM177	AP1S1	MASP1
GFOD1	EFCAB11	AFG3L2
TOX3	PSD	NME5
PHTF1	DDX52	HEMK1
IQSEC3	AMN	HERC1
SOCS2	MPP2	DNAJB12
KIAA1456	RP13-1032I1.10	EPM2AIP1
ZNF10	PDE6D	FAM110D
TUSC3	WIPI2	WWC2
GABRA2	BCL11A	SAMHD1
FPGT-TNNI3K	TNNT1	RERE
FBXO22	DAGLA	MSX1
SHMT2	SSTR3	ZMYND11
MYNN	WNT5B	EIF3M
LSM3	RDH16	ZNF276
ANKRD17	RUNX2	ZNF134
FARP2	CA7	TECPR2
DEM1	RNF126P1	TRIM52
SLC30A4	COX6B1P3	CLEC4E
PLS1	ARFIP1	ZMYM6
ZFYVE9	CHAF1B	CDS2
PPP4R4	HMGN2P9	MBD4
DYNC2H1	TFAP2A	TOX
CDON	ZNF131	PAQR5
PVR	FANCI	TLR8
KLHL35	TAF1	BMPR2
RRP15	NUS1	TRIM34
GRIA4	AURKAIP1	CRYZL1
ING1	MMP17	SGCD

PTER	DNAJC12	CLTA
AGBL5	PRRG2	REPS2
BAG4	FERMT1	BANK1
RASAL2	GPR35	LTK
ZNF324B	CCDC93	OSTM1
RWDD1	SLC12A8	KLRG1
ZNF180	CDC7	IL18
PACRG	KDM4B	ZNF516
PDK3	TBC1D9B	NSMCE4A
NRL	UBA6	TGM1
NOP16	DDX51	ALCAM
ZNF45	YAF2	PDS5B
PDE10A	RALGPS2	SIK3
KLF8	ORC4	ZNF34
ETNK1	SAA2	RBM5
DLG2	DPF1	IDS
PPCDC	TBL2	ITSN2
SLC25A15	DPEP1	ZNF280D
LIN7A	TMPRSS15	DPH1
ABCA11P	NSUN3	ZXDC
STAR	PI3	WDR91
RPS20P21	ZNF764	HIF3A
INPP5A	RAPGEFL1	VPS53
AGPS	TERF2	ALPK3
CAPN5	MASP2	CALCRL
CYP26A1	AMACR	RP11-738E22.2
UBE3C	HIST1H2BI	WNT11
KCNQ3	EFNA4	PDE7B
NT5E	CKMT1B	PPP2R5C
GCNT1	TMPRSS11D	ERAP1
RALGPS2	CBX2	CCDC19
LIAS	FZR1	ATP1B2
DAPK3	SEC24A	RPA1
EXOC6B	SLC26A6	SCTR
RAB3B	NFYA	BTD
GPD1	KNTC1	SLC22A4
OPRL1	CD79B	GVINP1
MAP3K13	CCNT1	ABHD6
TTC39A	CD19	HMGCS1
PEX26	DOK5	TMSB15A
OPRK1	DIEXF	USP36
TCP11L1	SPOCK1	PARVA
CDH8	KHK	KIAA1462
GPR107	KPNA6	PTPRC
MAPKAPK5	AP1AR	PLA2G1B
TIMM8AP1	ZNF468	GZMM

WNT5B	POC1B-GALNT4	PKD1P6
MAZ	NPTX1	ACSM5
CKLF-CMTM1	DNAJC16	SDK2
ZNF215	SLC6A2	PPP2CA
COPS8	GCNT3	LPIN1
TMLHE	B3GAT1	SFRP1
QSOX1	EML2	SPRED2
VWA8	NHLH1	IL1RL1
DUSP4	SPIB	MAML1
EXT1	DBF4	SLC9B2
MSC	GS1-115G20.2	CD80
EPHA5	ZBTB7B	SRSF5
AC007229.3	NR1I2	EPOR
COCH	CD2AP	SORBS2
RLIM	RHD	CPPED1
DNAJC9	DAK	SYNC
RP11-397A15.4	KDELR3	CFP
LEFTY1	PPAT	ZEB2
SLC7A6	MPI	ADAMTSL3
RNF2	RMND1	MFN2
LSM5	DNAH2	PALMD
CITED2	GRM2	LIN7A
CHRNA7	ANKRD36B	FAM212B
GUF1	SETD1A	LYVE1
TSTD2	COMP	CEACAM21
PCDH11X	ALPPL2	MAP4K2
TAS2R14	ZNF550	UNC5C
NOL6	FGA	ELF5
MAPKAPK5-AS1	MREG	FAM13C
CORO2A	NOX5	MAP7D3
SLC29A1	CCDC28B	QKI
GPR6	PBLD	TNFSF8
MCM4	XRCC2	LRP4
RHCE	DHODH	CFDP1
AURKA	MAPK8IP1	KDM5A
GPR88	NXT2	FLCN
ACADSB	MMP1	VWA8
PPIAP21	SLC17A7	SIGLEC9
CCDC132	ZNF507	GHR
LAMB1	SAMM50	CFTR
MTMR1	RP11-192H23.4	PANK2
VCPIP1	WASF1	EXOC6B
TFDP1	LIG3	RASSF8
ZNF277	PIK3CD	LRRFIP2
SPAST	ARID3A	BST1
RNASEL	HR	PSMD9

TERF2	FEV	APOH
RAD54B	SYNJ2	OFD1
ARMC8	TWSG1	AGTR1
TMEM121	EPS15L1	PIK3R3
BET1L	TP63	THAP10
ARMC9	MAPK8IP3	SLC19A1
LNPEP	SOX15	BCHE
NIP7	TRIM37	ERP44
WRNIP1	ARF1P1	PKIA
DHX35	MTOR	PITPNM3
PPP1R37	CPNE6	MYOM2
CTH	BIRC7	CLN8
SLC7A4	ZFHX2	SGCA
FGGY	EZH2	ZNF175
PCDHAC2	AVL9	SYK
GABRA5	COX6CP1	RP11-488C13.5
MAGOH2	SBF1	TMEM246
ZNF75D	MEX3C	AC093668.3
RP5-1052I5.1	HOXC4	DAPK3
HNF1A	ADORA3	ENTPD1
KLHL7	CEP72	CLEC7A
CDHR1	GALNT2	NOD2
FKRP	WHSC1L1	ZFAND5
SMPD1	VANGL1	LIFR
CRHBP	AL928768.3	GFRA2
CDC23	TMPRSS6	RPS20P22
ADCYAP1	TMPO	KLK7
NFRKB	THRAP3	CD244
GPR68	MNX1	DZANK1
YKT6	S100A1	MARCH1
KCND2	TACR2	CHP1
NIPSNAP3B	B4GALT1	SREK1IP1
FASTKD2	CD6	NEBL
TUBGCP5	RP11-571M6.8	ST3GAL6
SENP5	XDH	HCFC2
ANAPC10	GUCA2A	MYO10
GTPBP3	PPFIA3	DCSTAMP
AZI2	RP11-457M11.2	RWDD1
ARPP21	GTPBP1	ZNF510
NUBPL	ASPHD1	SAMD9
EMC1	GINS1	YPEL1
KIN	MPZL2	SCAPER
KCNV1	AL773572.7	GPR162
PEX7	CDK12	BTNL8
NCBP1	DEFB1	FAM65B
GCC1	TRIM45	MYOC

GREM2	ZBTB25	LUC7L
ZNF223	MYLPF	NFYB
WARS2	P2RY2	BEND5
AMBRA1	ZNF200	ITSN1
GPR173	CENPO	MKLN1
TPX2	PIGL	TMEM120B
CRH	VRTN	LSS
TRMT5	URB1	BCLAF1
CCDC99	NKX2-5	ZNF204P
HERC3	PKP1	PML
MIS18A	LEPREL2	SLCO4C1
DDI2	FGF6	NLRP3
ORC5	SPICE1	KLRD1
RIOK2	RAB11B	RYBP
RMND1	STAU2	PLCE1
ZWILCH	PXMP2	DEFA1
TBR1	MAPK8IP2	FOLR3
DGKB	ZSCAN16	EVI5
SDC1	DTX3	ARPC4-TTLL3
MFSD9	IGLV4-60	ZBTB44
PES1	ADAM11	ICAM4
SBNO1	CDKN3	KIAA0368
ADPRM	PPP1R3D	ITIH5
ZFP2	NAPG	GRAMD1C
TRIM3	EDAR	FMO5
TPK1	SMC2	LRMP
TWISTNB	NUP50	ETFDH
NLRP2	TRAV8-6	EIF1
ALOX12B	ALKBH4	IL18R1
TRAIP	MLL4	GOLGA8C
TLK1	FXYD2	TMEM87A
GTPBP10	GRAMD4	VSIG10
AIG1	POFUT2	RNF103
FBXO11	CA14	ST7L
ZNF764	SIX1	RANBP17
RAC3	NT5M	PGLYRP1
RBM3	PDE1B	FAM188A
BRAF	BRF1	EML1
DTNB	HS3ST1	S100A12
MIPEP	NCAPH	ST6GALNAC5
TSHZ2	WRN	ARHGEF40
PPEF1	HOXC6	PLCL2
SLC16A10	RASL11B	ZNF654
SLC18A3	AC021066.1	CALD1
PLAA	LILRA4	KIAA0664L3
RRN3P1	PAX8	ZNF222

GATC	KRT8P12	STRN
HTR4	ALDH1L1	XAF1
TDRKH	POM121L9P	APC
YAF2	ICOSLG	DHX30
RFC3	BAI1	TRPC1
TMEM223	PPP1R14D	LRRC49
XPO4	PIP5K1A	ZNF407
IGF1	TREML2	IFRD1
RHO	DYNC1I1	REEP1
SORCS3	ECEL1	NMT2
FAF1	CDC45	LIN7B
ARHGDIA	PDS5A	JAM3
RBBP5	CHST8	SLC35D2
CCNB1	AK4	CCDC102B
DUS4L	POLQ	PXN
RFXAP	LETM1	FMNL1
GLT8D2	CHRD	NBEA
TRIM46	IVL	DGKE
ATG10	ABCC5	GOLGA2P5
JRK	DKKL1	SNRK
LRCH1	TAB1	SNAI1
MXRA7	SLC6A3	KCNAB1
DPH5	SP140L	ANAPC13
LRP12	ZBED4	MCOLN3
NRG1	LOX	LSAMP
GIN1	CSNK1D	FGF9
STEAP1	TRAM2	SETMAR
RSAD2	MC2R	C2CD2L
CDKN3	FKBP14	DYNLT1
POLRMTP1	INTS7	TRIM8
PRSS16	DNAAF2	RASGRP2
RPAP3	FUT2	PEG3
AC004381.6	PACSIN1	SPRYD7
CCT6B	ENTPD7	ETV1
ZNF667	WNT7B	MTM1
POLR3G	TRAPPC9	MAF
SHH	ATAD2	CD101
MAGOHB	KCNF1	CUL2
CEP76	NRIP3	DACH1
RNLS	FMO1	NOV
ZNF585B	GLE1	SEBOX
PVRL1	ELOVL6	RP13-608F4.6
INSM1	CAMKV	HAS1
MAN1A2	RP11-375F2.3	HMGCS2
PCDHA5	RSG1	MTO1
PPIL2	GCNT1	DMXL2

RP11-706O15.3	PPP2R1B	RAB14
BEAN1	SEC24D	GLT25D2
KIAA0895	MYL3	GTDC1
KCNB2	DDN	SOS2
RPGRIP1L	KIAA0586	ARHGAP24
ADAM17	ZNF205	HSPA12A
SOSTDC1	PKM	PLA2G5
KCNH1	IRAK4	GPHN
FLT3	APOBEC3B	AC013461.1
WNT2B	PNOC	ZCCHC6
CLGN	CITED1	RNF11
PELP1	ZNF193	RPGR
MTF2	CENPJ	ZNF263
GOSR2	TMEM209	RASA1
SPAG4	KDELC1	FCER1A
RXRB	ASIC4	FUBP3
LXN	TCF15	ARHGEF4
LRMP	CRKL	PDK4
MCPH1	PTPRA	ADAMTSL2
KCNIP2	SPINK1	GPM6A
RNF19B	GRTP1	MYBL1
GPATCH2	TIPIN	DIS3
RRAGB	PSG1	FBXO9
TMOD2	CCR7	DICER1
ROPN1	PDK3	PENK
AP4S1	ETV4	OGDH
TSC22D1	MRS2	SEC14L4
RNF170	CDA	SCAF11
ASCL2	RPRM	B3GALNT1
ZNF287	MYL10	SOX5
RBBP9	TNFRSF25	ADAM17
KLK5	IFNA10	ATP7B
CCDC41	SUPT3H	PVRL3
AGA	ALDH1B1	IDH3A
EDN3	TBCE	LIMS1
GLRA2	TCL1B	C1RL
NEUROD1	EEF1A2	NTRK2
ARL15	RNLS	LGALS3
ZNF200	RPL21P2	ZFYVE16
PARG	CASK	TPK1
RGS7	RHPN1-AS1	KIF17
SLC12A6	RABEPK	CROCCP2
RAD51D	CLCNKB	SNED1
IRGQ	APC2	RFX3
PPP1R17	CHRNB1	PTPN2
NMU	TMCO3	NSMAF

SLC1A6	SULT4A1	KAT2B
KLHL4	RFC5	MYO1B
BFSP1	ZWILCH	CSF2RA
EPCAM	RABL3	TAB2
NPY5R	CRYBB2P1	SAP30L
VAMP4	KIAA0125	SLC26A10
CCDC68	WIZ	LRCH1
QPCTL	SLC25A13	F10
TBCE	RPL30	SPAG9
KRT17	TOP2A	NOS1
CADM4	CDKAL1	FAM105A
LRRTM4	MARK1	LMO7
RNMT	ZNF669	CD40
CDH9	CRYBB3	PPFIBP1
STMN3	ZNF239	PPBP
UBE2D1	IGKJ5	PARP11
TGM3	NCS1	TCF21
TNFSF9	RAD54L	ADAMTS9
VIP	ZNF3	SZT2
GUCA1A	PCBP3	PDE4A
TECTA	MYOD1	RP11-164J13.1
HEATR3	SOX2	SEMA6D
RP11-583F2.3	COPA	PDLIM3
GRIK2	SLC2A5	ACOXL
CUL2	CACNG1	CORO2B
SRR	ENTPD4	ADI1
PLCB1	SCIN	WWP2
RNF187	TAGLN3	FBXO38
RNF31	FAM155B	FAM63B
HLCS	HABP2	LY6G5C
IL1RL2	POLE2	NEK3
OPA3	PRDM14	ROS1
BRCC3	SBNO1	NRXN3
ERCC8	TAF4B	NFASC
FAM163A	PHF13	FLI1
DSN1	PBX2	ATXN7L1
PTGS2	AP3B1	NFIB
ASLP1	LY6G6C	GDF10
PCDHB2	ZNF473	ZNF24
BLZF1	MCM4	AFF3
RAB35	LINC00652	LEFTY2
ST8SIA3	PNMAL1	PHACTR1
TOX4P1	FAM118A	GYPB
SPP2	SLC30A5	ZBTB7A
PPARG	SPRR2D	PTGER2
CACNA1C	PRKCI	CYP26B1

UGGT2	GPRC5D	NFATC4
WDR52	KIF22	ATP8A2
PDGFD	RAX	SLC6A4
RET	PSG3	IKZF1
TUBB7P	B4GALNT1	RIOK2
TEC	GNRH2	ARHGAP28
MSANTD1	GFRA3	KCNA5
GPR63	NXPH4	AC012065.7
HCN4	GNB1L	VNN3
LRRC2	MKI67	MAPT
N6AMT1	CXCR3	ABCF2
ITIH3	IQCC	DYNC2H1
ZNF259P1	NCL	ARC
TUBBP5	PEX26	RAVER2
DBF4	TRPV6	CLDND1
SFTPA2	TDRKH	APBB1IP
INPP4B	DENND1B	EXOG
LIG3	SH3PXD2A	LINS
DOPEY2	ZNF787	GRIA1
PDS5A	DRD2	SGCG
KCNA4	COL10A1	FLT3LG
IL13RA2	DNMBP	ANKRD6
HTR2C	POU2F1	ERG
COX11	SLC29A2	C6
CCNF	GNL3L	UAP1L1
SPINK2	THOC5	PRPH2
TTC21B	BCAN	ZNF37BP
MCF2L-AS1	C4BPB	PRSS21
CLUL1	ZNF215	LILRB2
SOGA3	NCOR2	ACADL
KCNMB2	SCYL2	ASXL2
FAM189B	RSPH6A	SPATA7
PRKACB	CPSF3L	TTBK2
ZFP37	NUBPL	IFT74
RASA3	BUB1B	FLT4
ADAMTS3	MBTPS2	NUMB
RP11-707M1.1	PYY2	FGF18
CCDC70	HOOK1	RFXAP
EMX1	TNFRSF17	STAB2
CYP2C8	SMPDL3B	NADSYN1
VAX2	RIPK4	TBXAS1
HSF2	NREP	NAALADL1
SLC3A1	SCN10A	SEC22A
PCNX	NDST2	CYTL1
GRIK4	GLP1R	MYO9A
SMPX	SAA4	LIG4

AGAP2	ARHGDIG	GUCY1A2
KNTC1	CST2	CEP112
ACTA1	ETV6	RBPMS
KRT31	TLK1	PHEX
CASQ1	BCORL1	BMPR1B
PRG2	CDH2	TAL1
DNAJC13	PDZD3	CHD9
N4BP3	CEP76	GFRA1
KCNK10	GAST	NTNG1
MAD2L1	PPIL2	ZXDB
ZNF135	RP3-468K3.1	MMP23A
ATP2A3	CHURC1-FNTB	ITIH3
ELAC1	RPL3L	PLCB4
THNSL1	RP1-130G2.1	NLRP1
FBXO40	APITD1	SGCB
AL603926.3	GCGR	TSPAN2
CRYBA4	ADORA1	USP53
TBC1D3P1-DHX40P1	DEDD	DYX1C1-CCPG1
ADTRP	SPTBN2	COL4A4
TMEM62	ORC5	P2RX7
CUZD1	RABL5	ZC2HC1C
BUB1B	SMURF1	OTUD4
TOM1L1	DLG4	CSPG4
POSTN	UBR4	LILRA2
ASB1	AZI1	CTD-2540L5.5
RFWD3	KLK8	ZNF426
PLEKHA8	LUZP4	CDK17
SLC27A2	RP11-259K21.3	NAA16
DDC	FTCD	ARAF
ZNF780B	IZUMO4	WDR33
MST4	FAM189B	ZC3H14
TAF7L	FBXO5	EIF2C4
REN	PPARD	PI4K2A
BTN3A3	TFF1	MCPH1
TDO2	POP1	ALDH6A1
GPR1	KLHL35	CAST
AFP	MRPL44	DENND4A
PAX7	PLEKHA6	NAB1
CHRNA4	GLS2	GGA1
YTHDC1	ETNK2	NUMA1
DIRAS3	BCKDHB	ATG2B
LMO7	TACSTD2	PDE12
FHP1	EDN2	DDC
CDK1	KDM8	TMOD2
FDPSP5	TCP11L1	JPH2
PAH	HPCA	MR1

GPLD1	MEPE	SCARA3
RP13-210D15.1	PMFBP1	AC022532.1
INSL3	WHSC2	PPP1R2P4
INSL5	DPF2	TNFSF14
GJB5	KIF18B	SMAD4
DNAAF2	EIF2AK3	SYNJ2BP
E2F5	LIAS	TNF
ZBBX	DCAKD	KATNB1
GEMIN2	IL23A	LARP4B
NR2C2	DLG3	CAMSAP1
SCLY	FA2H	NAIP
TCEB1P3	VGF	ACTN2
TLL2	ZKSCAN3	SH2D1A
HTR7	INO80B	LIMK2
SGCG	ASMTL-AS1	C2
SLC12A1	KCNMB3P1	INTS6
ZBED4	FAM98A	MYL12A
YEATS4	MICALL2	PHF7
COL13A1	DHFR	UBXN1
ACN9	GABRR2	KIAA1704
DHRS2	TSFM	LILRB5
RP5-916L7.1	SLC4A3	RP11-499E18.1
RARRES1	GTF3C3	ZNF236
TBC1D22B	EVX1	NOS3
COMP	ZNF202	RAB2A
KIAA0020	CTD-3126B10.1	MFAP3L
MYBPC2	SYN1	GPR20
POU6F2	CHRNA6	AGRP
MYBBP1A	ARSE	SFRP5
SCN11A	CD177	CETP
GPR22	EPN3	YTHDC1
FN3K	PART1	NACAD
CCL19	SLC16A8	RNASEH2B
RPS6KA6	RP5-1119A7.11	CSPP1
BAG2	MAPKAPK2	BMP5
PEX13	SLC25A15	MMP24
CRYBA2	FRMD1	ZNF160
RAB11FIP1	SLC39A2	LARP7
AZU1	PAK1	PDE3B
PHF14	E2F2	NPFF
PCDHAC1	LRFN3	FKBP15
RAB40A	ZBTB17	TSEN2
CCRN4L	ZNF710	PGR
HCG4P6	ZFR	CXCR2
MTMR6	SLC2A11	SNCA
PCOLCE2	CAMK1G	ZSCAN5A

NPY2R	RAD51	TNNI2
ALS2CR8	APBA1	TESPA1
DPYS	RTEL1	RARA
MTMR7	ELAVL3	GPR64
DNMT3L	RAI1	TRIM33
RP11-473I1.9	SPANXC	KANSL1L
TACSTD2	ICK	FHL5
KMO	NFS1	ERBB4
NOL10	ZSCAN2	PPP2R3C
FBXL8	VPREB3	FAM110B
GTF2F2	E2F5	KLF8
SFMBT1	CGB	CRTC3
RNFT1	ZNF79	HBP1
KIAA1024	SYT13	TLR7
EPHA3	PSORS1C2	MDM4
MLLT1	OR10H5	FAM149A
CYB5RL	ZNF668	STX11
HTR7P1	GBX1	WSCD1
DCAF17	TAPBP	FER
TNFRSF11A	BORA	CEPT1
PTTG3P	CCNA2	FAM48A
PRR16	F5	SYNRG
CSTF2	PTGER4P3	TEKT2
MPP6	SLC8A2	ALAS2
KLRC2	YEATS4	DNAJC6
AC004478.2	ZKSCAN5	PDE4C
EGFL6	RP13-221M14.1	KIAA1009
IL12RB2	EHF	ITGB6
HAPLN1	TMEM177	SLC6A12
AFAP1	ST6GAL1	FAM204A
CNTN5	COL11A1	ZDHHC14
INCENP	PRUNE	CYLD
ZNF702P	KLHDC8A	PPM1A
NAT8	OXCT2P1	ABCB4
SLC9A7	NGF	CXCL5
NF2	LILRB4	PPARGC1B
CASC1	ACOT8	CLUAP1
CBLL1	ASH1L	SLC12A6
CNTN6	KCNE4	GGNBP2
ARHGAP28	GNAS	ZNF177
LGALS13	CEP41	USP2
GLP2R	AC006011.4	PRDM16
PLK4	TTLL4	SOX17
RP11-292B8.1	KIF4A	ALPP
PTHLH	ABCA4	COBLL1
MPHOSPH6	CUL4A	NHLRC2

ZNF334	CYP27B1	CLCN4
GUCY2F	ITCH	SEC61B
ZNF551	NYX	ASB1
ZNF780A	HUS1	SLC18A2
RS1	CSPG5	MOCS2
ZNF132	BRCC3	STEAP4
GYPE	TRAV27	U73169.1
SNTG2	ACAN	AQP4
CROT	ANGPT2	BAALC
BBS5	PLCH2	SMPD1
PTPN20A	EPHA1	JAKMIP2
NEK2	PSMC3IP	ITPKB
ICOS	PAFAH1B2	SFSWAP
TAF1A	PNO1	ICAM5
AL928768.3	TMEM48	LRP2
RP11-277P12.6	OR7A5	PREX2
ADRA1B	CYP2D6	TCEAL2
SCRT1	DTX2	NAV3
TRPC3	MAGOH2	PTPN22
AANAT	ETNK1	PYGM
ABCG5	GNAO1	TLR4
HCG4	TMEM126B	PGAP1
WDHD1	RBM41	CYP46A1
WT1	SUV39H2	ABCA11P
TYRP1	DKK1	ZXDA
CSN1S1	RP13-98N21.3	JAK2
TTC30A	RP1-179E13.1	PRRG3
MTNR1A	MPZ	RP11-612B6.2
SCN9A	FKBP6	ASB9
LCE2B	LHX3	FGF14
NPPC	GTPBP2	RP11-57H14.4
ZNF154	HMMR	LCP2
RP11-770J1.4	DNASE1L2	AVIL
ZNF643	PMEL	CDC37L1
DNAJC28	RRP7B	Y_RNA
SPCS2	APOA4	RP11-35N6.1
HSD17B12	EPO	LSM3
TMA16	NUDT11	ZNF224
ADAM3B	NT5C	EIF1AY
LPA	BPTF	IQCK
RP11-430B1.2	PTPRH	CRYGS
SEMA3E	KEL	SYF2
PARPBP	UCHL5	BTN2A2
ZNF165	MAX	PPOX
PCDHA6	ROM1	CREB5
BTF3P13	AL590762.11	EPM2A

ZNF492	PTK2	NFATC3
RP11-325N19.3	IPP	WNT2B
NUS1P2	PITX3	IKBKG
CRTAM	SERPINA5	THSD7A
CCR6	APOBEC2	SIGLEC6
OR2W1	STK32B	TMPRSS3
KIF11	TBC1D8B	STAM2
GABRA6	EYA2	SEMA3F
BTC	ZNF623	PDE4DIP
PDE6H	HOXB1	PTPN9
INHBE	OAZ3	PDZD2
ZNF677	HECW1	AASS
RP11-645C24.2	ACTL6B	MED31
DKK2	HOXD4	PRR5L
PBK	EPHB2	PTX3
PNLIPRP2	EIF2AK2	OMG
PPIAL4A	KRT75	NOVA2
RP11-170N11.1	BET1L	ZNF235
ANKRD7	DOPEY2	CD300A
TXK	MAP2K7	CARD9
GLUD2	KCNC3	MPHOSPH6
TRPC5	UGT1A8	RALGAPA1
PCDHB3	BMP8B	ABCA6
	MLN	KDM4C
	THEG	MEOX2
	CUL4B	GPR132
	TRPV4	KIAA0141
	G6PC2	SLC22A3
	RAB26	ARNTL
	DTL	CREBZF
	FSCN3	CLIP1
	LRRC8E	CACNA1D
	RCOR3	BEST1
	CACNA1F	EFCAB1
	UROS	MFSD6
	CCHCR1	POU6F1
	CRYBB1	FAM76A
	TULP4	SYNE1
	FOXK2	ATP1A2
	ARMCX5	TM4SF1
	NCR1	KIF13A
	OR2N1P	POLI
	SAG	SV2B
	HOXC13	VGLL3
	PFKFB1	PELO
	CDKN2A	KPNA5

TRPS1	SRR
COX6A2	DLEC1
ADM2	CD1D
HAMP	ATP5S
SCG5	SPAG6
ARG2	DKK2
AC007952.5	ASPA
PFKFB4	ACSL3
RP11-616M22.5	TFE3
GINS3	ABHD2
SPTLC1	IFT140
KRT9	TRDC
LARP4	CD22
CEBPE	DZIP3
TAF4	NCAM1
TBC1D29	ACTR8
RP11-181G12.2	CYSLTR1
KCNK10	LPHN3
HHIPL2	CDH6
CCNO	RRAGB
IFNA13	TRIM23
TRH	CACNB4
MEF2BNB	NLK
DPY19L4	U6
ESF1	DNAI1
TOX3	SLC6A13
PCNXL2	HSPB3
CRYBB2	AF011889.5
STEAP3	SNAP23
PRR4	UBBP2
PPP3R1	FABP3
RIF1	COL4A6
BRCA1	BRWD1
PLK1	MON2
ASIC1	IL15
SLURP1	PLCL1
RP11-661A12.7	CR1
METTL2B	ATF6B
RP11-65G9.1	SLC5A1
ENTPD5	LDB3
GREM1	KLKB1
SHBG	ASGR1
WDR74	CASP5
AC027612.1	TRIM3
DUSP13	OGN
KAZALD1	CCDC170

POU5F1P3	KIAA0754
ABCB9	YSK4
CENPE	PKD2L1
RAD51AP1	NAP1L3
DSCR4	PPARGC1A
F2	FZD8
RP11-374M1.3	RP11-566E18.3
ACN9	HNRNPK
USH1C	IL7
HIST1H3G	MNS1
IL37	VPS8
RP11-783K16.10	KCNC4
KIF20A	TMEM212
BARX2	MAP1LC3C
IL19	ZNF365
ADAMDEC1	EIF3F
XIAP	WNT7A
ADAM12	LRRC48
INE1	CLN5
MKL1	SOX18
KCNQ1DN	ICOS
DOC2A	NGDN
IFRD2	HEY2
CNPY4	CRTAM
CDH15	BACH2
SS18	CYP3A5
EXTL1	TRPC6
SMCP	RB1
ANGEL2	PCDHA3
NLGN3	KBTBD4
ATL2	RCHY1
VPS33A	AC000120.7
CARD14	RPP30
MPPED1	DNPEP
TTC3	SIRPB1
PMPCB	EWSR1
CDC25A	RP11-499P20.2
PTCH2	MGST2
EGLN3	MED17
CEP97	CTRL
MORN1	RGS13
SIGLEC15	RP11-2E17.1
HHAT	RP1-240B8.3
CDT1	GEMIN8
HTR3A	DIRAS3
MINA	ABCB1

CD72	RBPJ
GP1BA	PAPPA
GPR21	NMUR1
R3HDM2	LMO4
SLC16A7	IL20RA
TCL1A	CHRM3
POFUT1	HBD
NFE2L3	SPATA6
BCAS4	MAPK10
TMCO6	SELE
RP11-235E17.2	KLRC2
ETAA1	ZMYND10
FUT7	AFF2
PRY2	KCNN2
DHRS11	GALT
GID4	DDO
AUNIP	IKBKAP
TSPO2	MRPL20
TMSB4Y	CDKL1
PRSS3	ATP8B5P
TERT	ARHGEF26
NEUROD6	SYN2
SLC4A1	SHANK2
FAM120C	ITGA1
RBM12B	SLC8A1
UBE3A	HAS2
DSN1	DGKG
INPP4B	TTN
PRRX1	ZNF45
RP11-510M2.9	USP12PX
FBXW11	MTMR6
PREP	SMC5
CELF3	EYA4
BHMT	CTA-134P22.2
IPO8	SBSPON
GUCA1B	LYPD1
LINC00599	SPEF1
AC009237.1	PRSS12
ARHGAP32	SCEL
DGKA	RTDR1
RP11-340I6.6	CNOT4
RAB3D	EXD3
PCDHB2	CNR1
ABO	ETV3
SUSD4	NR5A2
NT5E	VPS26A

CHML	RNF185-AS1
POMGNT1	WI2-1896O14.1
KIF3A	GSTA3
BCL2L14	HSD17B3
ASB7	BMX
LY6D	ITGA4
CLEC16A	APOBEC3A
GJA8	CST5
IGF2-AS	SSBP2
RFC3	STK4
SPRR3	FAM70A
GREB1L	RP11-72I8.1
ARR3	FAM124B
ANGPT4	PDE8B
PDZD8	TEC
TRIM17	STX3
ABHD11	VPRBP
MEF2BNB-MEF2B	SMEK2
MMACHC	KCNA3
AL137067.1	CYP39A1
CTH	ZFP112
ACTR3B	NXF3
TRPV5	HMGN5
SLC25A16	SCARF1
CNOT2	CACNA2D3
TRGV5P	PLAA
AQP2	TFEC
SIKE1	HIVEP1
SNCB	SCAF4
GCKR	CAPN9
LAG3	GNMT
AC003989.4	ZNF536
SRMP1	FLT3
BHLHB9	ARMCX4
CYP2C9	RP4-788L13.1
EEF1DP5	GATA3
IL2RA	PLA2R1
SEC61A2	SNAPC3
APOL5	RP11-583F2.3
SLC16A1	MFAP5
TBX1	ZNF75D
PSG4	SIAH1
SGPL1	CYP3A7
SLC7A11	DNAH7
MOCS3	KHDRBS3
HAPLN2	TPM3

FBXL18	CCL23
RP11-566K19.6	WNK1
SLC25A23	DPP6
ERN1	DZIP1
SERPINB3	DNAH6
GATC	ADCYAP1R1
IL22RA1	TAPT1
PGA3	BCOR
LAX1	ZNF667
GLDC	RP11-645C24.5
SLC5A3	SUCLG1
USP22	BTC
PRMT1	TYRP1
MUC2	AKAP6
CCBP2	TIMP4
STAT2	FPR2
MYBPH	NDUFA5
SERHL2	KLRF1
RHEB	RAPGEF4
IL21R	PDCL
ASIC2	FASTKD2
KCNQ2	ZNF10
RARB	EPHA3
KCNH4	DNM3
NUDCD3	TMOD3
PSPHP1	SMC1A
NAA15	FAM82B
STIL	SPTLC3
ENO3	NFE2
HS2ST1	PACRG
ZMYND8	TEX14
FOLH1B	RIC3
SYT12	MLC1
HOXC5	PTPN4
SMPD3	TTC12
ABTB2	BDNF
BCAT1	KCNJ12
GPR143	DNAI2
ZNF696	TNN
RASA4B	TTTY14
CACNA1I	H2AFJ
SSX2IP	THAP9
ZFPL1	RP11-313D6.4
PROP1	DOPEY1
PADI3	F11
CLCNKA	UTY

SKP2	RASSF9
NMU	GNAL
RP1-149A16.17	CDKL5
SYNDIG1	ENPEP
sept-09	PDE1C
AP5M1	POPDC2
RAP1GDS1	AC068039.4
FASLG	MYOM1
SLC38A3	OMD
CENPA	SENP3
PROZ	IREB2
LINC00574	GABRB2
SEMA3A	SEMA6A
RFWD3	CDADC1
ZNF253	HBS1L
LBP	FAM189A1
COL9A3	NLGN4X
GNG4	SSTR1
PLXNA3	RP4-791K14.2
ASCL3	ZNF493
CHRNG	TRDMT1
RIMS2	SLC31A1
NSG1	RHOT1
FAM120A	TGFB2
ABCG4	MAK
AIRE	OPA3
CACNB1	NXPE3
OTUB2	TAS2R14
RP11-293I14.2	EMR3
SLC1A6	AGTR2
TTF2	ARMC4
KIAA1609	SLC6A16
SLC13A4	RPAIN
AKR1B10	PCSK2
CHAD	CUL3
BCL9	RGS11
ABCB8	MEOX1
NPAS2	CD160
FBXO22	ZBTB11
CAMK2B	TMEM35
ANK1	ZNF197
DENND1A	SCRG1
AC131971.1	NAP1L2
HRH3	MLLT3
EBI3	RP11-680F8.4
CCDC106	CLEC4M

KIF20B	HBZP1
DBNDD1	LILRA1
EHMT1	RP11-405O10.2
MYL7	HYDIN
KCND1	PLAG1
PEX13	ZNF571
ANKRD2	KCNQ4
PRSS50	ZNF271
INA	CCRL1
TRIM32	GCC2
OR10J1	KIAA1456
VCX3A	OR2A20P
KRTAP5-9	MST1P9
TNFSF4	USP9Y
DRD5	TNNT2
DCBLD2	DEFA4
Z83844.1	FGF2
UTP14A	HIPK3
PHOX2A	SLC1A2
EHHADH	ZNF682
GNG3	ANKMY1
ASPM	SLC14A1
KCNG1	CLEC1B
GPATCH2	GRK4
RAB6B	PCDHGC3
PI15	MMRN1
PROC	TTLL7
AP003068.6	DUOX2
RAD54B	CATSPER2P1
ZNF528	CACNB2
SENP5	CCK
RND2	ADAM22
OGFR	PYHIN1
AC004381.6	CAMK4
FAM83E	SPAG8
BCAS1	EIF4E
SERPINA4	CHD2
LMAN1	ODAM
MTCP1NB	ST8SIA1
ZG16	SCN3B
RP11-813N20.1	UPB1
PACS1	NFX1
HHLA3	PRKG2
GPR52	KCNE1
NGB	SEC14L1P1
COL17A1	IL1A

MYOZ3	CPEB3
PSPH	CD5L
KRT16	SLC9A3R2
DRP2	TRPC2
EIF4G2	STAG1
RHO	LNPEP
GSTCD	WDR78
HIST1H4I	MBL1P
KLK14	CDH19
NDUFA2	PRM1
S100B	SCN1A
WWOX	ELANE
CA5BP1	PRMT8
SLC27A2	ZNF529
BAI2	HPCAL4
AGBL5	GIPC2
CELP	WDR52
ASCL2	MINOS1P1
CDC40	YIPF4
KCNQ3	SCN2B
CELA3B	FBXW2
ENOX2	INADL
ZMAT4	DNASE2B
CYP26A1	RXRG
FANCA	TAF9B
NAGLU	TKTL1
CELSR3	FGF7
CRYAA	SNAP25
AL357673.1	ADCYAP1
PUS7L	CDC14A
MPHOSPH9	ZNF141
HAP1	ITGA2B
DNMT3B	UQCRB
GFRA4	NUDT13
LGALS7	HTR7P1
NPHS2	AOC2
DIP2A	ZBTB3
RDH8	LRRK1
MUC13	WHAMMP3
PRR14L	PRDM5
NTSR1	PDCD1LG2
RHCE	CNKSR2
GCC1	GFM1
ZNHIT2	ATXN3
SPANXB2	CTNND2
UPK3A	HERC2P3

TF	CRHBP
RP11-390F4.3	ELAC1
CCR4	GPLD1
TANC2	MYRIP
RECQL5	BAI3
SCGN	LRP2BP
ATP5G2P1	KCNMB2
RPS4L	KCNA4
NR1D1	LILRA5
BMP15	ANO2
PHTF2	PCNX
OR7C2	KCND3
TAAR5	PVALB
A1CF	AGBL2
NOX1	NTRK3
ELK4	ACSBG1
MST4	MYNN
TG	ALOX15
RP11-261C10.3	ALDH7A1
GP2	SLC19A3
TP53TG5	VGLL1
PADI1	MAGI1
CDHR2	KCNB1
CERS1	ANGPTL7
ARFGEF2	NPY1R
RNFT2	NABP1
RPS3	RPA4
ART1	PPP1R17
EXO1	ZFP2
HOXC8	HOXA2
GAL3ST1	TRIM36
NMNAT2	CD1E
TMEM40	TRAT1
TAS2R1	PELP1
SYNGR4	ASXL3
ODF1	L1TD1
CELSR1	MS4A2
AC105020.1	ZNF471
KLK6	SEC14L3
GCFC2	ART4
CBX8	SLC6A1
N6AMT1	RP11-617D20.1
GALK1	AJAP1
CDC23	FKRP
TRIM15	CNTN6
RNGTT	WBP1LP2

DOCK1	TTC21B
MUC16	EDN3
OR3A3	PTPRG
SYP	UTF1
SLC24A2	CD163L1
NKAIN1	GPR85
SSX7	RP13-514E23.1
WDR59	RYR2
FFAR3	KL
ZMAT3	ZNF506
AP000350.7	SLC17A3
OGDHL	FAM153B
SSX3	PYGO1
CYP2W1	NME8
SIX2	MRPS18C
TRBV5-2	HMGB1P3
SYT1	TUBB1
PSG7	NPY5R
LRP8	CMA1
DPH5	PADI4
TTC26	GP6
ITIH1	NIPSNAP3B
RP11-435I10.3	ZNF440
HKDC1	WDR96
ACVR2B-AS1	RUNDC3B
CRHR1	COLEC10
TCN1	RGS9
MFSD9	MBNL3
CASR	TLE4
CYP21A1P	CNTLN
CTD-2269F5.1	ZNF442
UBE3C	DNAH9
ANXA2P3	OTC
PPP2R3B	CTAGE5
MAZ	ENDOU
UGGT2	MLLT4-AS1
CTRC	CCDC30
RP11-414J4.2	NANOGP8
PRICKLE3	CCDC81
PAK7	PTGDR
LTA	CYP2F1P
GLYR1	P2RY1
CCDC57	RPS20
NOX4	GYPA
ITIH2	MGAT4A
OXT	CLTC-IT1

LRIG1	SPATA6L
CALML3	SLC6A20
MUC4	ZNF385D
RP11-195F19.30	MGAM
TAF1A	PTGFR
RPL12P11	RRP8
RGR	KCNJ16
DUSP9	DPYS
CELA3A	HCRTR2
NSD1	SH3GL2
DGCR5	ZNF135
RLBP1	RP11-769O8.3
PAX4	CASS4
IRGQ	BBIP1
CBLC	TTC40
RGS6	SLC3A1
NEU3	GAS2
CCDC134	ANKRD1
PLXNB3	EBF2
CAPN5	ZBBX
FZD9	ZNF132
DSC2	SLC4A8
WNT6	ZNF345
DIO2	CD8B
PRSS3P3	TNIP3
FLRT1	CA3
F7	DLGAP1
CCNB1	FPGT
CCKAR	ZNF287
RNF186	SLCO1C1
RNF7	NRXN1
AMBN	GREM2
SH3BP4	RS1
FHOD3	SYNPO2L
AQP6	EPPIN
NEUROD2	TRHDE
CABYR	ANKRD55
SPINK2	GRIK4
NEK2	ABCC9
CTLA4	ANKS1B
FOXH1	PLGLA
PRAME	EFCAB6
CDH16	RERGL
GRIN2C	CACNA1C
COL9A1	IL10
CST8	CCDC121

CEMP1	SLCO1A2
BTNL2	ODZ1
VIL1	CASC1
ERCC8	ZNF804A
XRCC3	ZNF334
CHODL	PTPRR
LDHC	ALS2CR8
BEST2	SEMA3E
CABP5	ADH1A
SPINK4	SCN3A
IL9RP3	CASQ1
RP11-450I1.2	ADRA1A
SCN5A	B3GALT2
HTR3B	CELA2A
VAV2	MATN3
NPAP1	RYR3
SCAND2	ANO3
GPR19	ALDH8A1
CLDN16	GPR17
RBM48	CDH10
PIN1P1	MYH3
RBBP5	RP11-451M19.3
CLCN2	LRRC2
FNDC8	LINC00472
SLC22A8	PPP4R4
PSPN	ARG1
PARG	SENP7
ARNTL2	TXK
ODZ3	PRG4
UCN	MAB21L2
LINC00675	CRISP2
CDK19	HPR
NDC80	FPGT-TNNI3K
TGM3	PDZRN4
SLC1A7	CWH43
PCDHGA9	PLP1
MCHR1	SLC27A6
UPK1B	KLHL4
ENAH	ZNF154
DNA2	RP11-292B8.1
KRT83	GYPE
EPS8L3	TSGA10
TMEM194A	NAALAD2
TYRL	SERPINI2
ACTL7A	RORB
IL1RAPL2	ASTN1

TBX6	CEACAM8
PCDHA10	RPS2P45
CD207	AC007405.4
BMP7	GBA3
GUCY2D	TRPC3
RP4-796F18.2	FCAR
HGFAC	SOGA3
MSC	DEM1
STRN3	GPC5
MAST1	PCDHA2
KRT85	MYOZ2
SP140	RBMXL2
NOL6	TRIM58
TM4SF5	GNRH1
AGXT	MYH2
TMEM131	VIP
ZSCAN12	BBS5
GPD2	SULT1E1
RNASE2	IQCH
PIK3CB	MAB21L1
IL25	RPS29P28
MVK	FAM5C
PRAMEF12	MYOT
KIF11	CPA1
THADA	CYP2C8
INHBC	ZNF549
KIF5C	DRD1
TOP3A	KLRAP1
ITPKA	SNX29P2
ZNF771	ADGB
GABRD	CCDC144A
TBX10	TAC1
P2RY4	UGT2B4
GRIN2A	LRRTM4
CYP4F2	IFNA1
KRT23	RP3-391O22.2
CDSN	SPATA1
ZNF701	MYH1
ENTPD2	LRRC19
PCP4	AGBL3
RP1-256G22.1	TPH1
THNSL1	RP11-549J18.1
KLHL7	RAD21L1
AMPH	MGAT4C
CACNG5	
IGSF9B	

CACNA1G
RP11-404P21.8
KRT32
FAM90A1
S100A5
HOXB9
GYG2
HSF2BP
GDF11
VPS16
ACRV1
GMCL1
ACTN3
GTF2A1
CNGB1
HOXC10
IBSP
CETN1
FGD6
KLK13
ZNF74
MAGIX
SRC
RPAP3
AP001029.1
PPIAP21
DCAF16
FOXP3
KCNE2
GRIN1
OR2S2
CLDN1
KIAA1614
MTMR8
EDA2R
RGS17
FOXG1
ARHGEF38
SLC10A1
PAX6
STEAP1B
MATN1
HSF1
HBE1
WNT4
TRAIP

CNTN2
LRP5L
PLD1
POU3F1
TTC9
BAAT
CDH4
LRRC41
ZNF557
LPO
GPR12
RUNX1
NPTX2
AIM1L
IGF2BP3
NKX2-8
GTSE1
TFR2
SULT1A3
PRSS3P1
HNF4A
SIX5
FAT2
TAF1B
DEFA6
NPC1L1
BHMT2
CXXC4
RP5-845O24.3
CLCN1
TFDP3
PNLIPRP1
CCDC87
SCNN1D
TM4SF4
GS1-164F24.1
DAO
TNFRSF11A
SFMBT1
HAND1
CSPG4P2Y
GPR137
ZNF324B
RP11-560G2.2
KLK2
RP11-12J10.3

CR2
DSG3
RP11-414H17.5
INHA
MATN4
CRYGEP
P2RX6
VIPR2
ETV7
CER1
MTA2
RP11-134P9.1
PET112
ZNF124
AC015936.3
TOR1A
TNPO2
PHC3
AMBP
PAX5
CYP2A13
COCH
PLK4
CRYGC
HTR4
DLGAP5
PLAC1
DLX4
FAM210B
NOP9
POMC
PSG6
GRM5
ALX3
LIPG
DBF4B
RBFA
KLK3
ENOX1
WT1-AS
UPK1A
XRCC4
ALKBH1
CEP55
AURKB
TIMM8AP1

OR1E3P
P2RX2
INSL3
OR2C1
NCR2
MAGEA9B
TNK2
RP13-362E11.2
SYNGR3
FSTL4
RGSL1
RP4-673D20.1
GRIK2
RHCG
GTF2F2
TRGV5
SLC26A1
TLL2
KRT33B
INPP5F
SIRPG
PKLR
CYP17A1
FOLH1
RNF2
GIGYF2
RP5-1184F4.5
RUNDC3A
RHAG
BUB1
SPP2
PTPN14
LRRC3
PRKCG
KCNC1
KCNB2
PRKACG
STK3
RCAN3
KIAA0895
GCNT1P1
CTD-2587H24.4
KCNG2
DFNB31
SLC17A4
CRYGD

SEZ6L
PRB3
TFF2
ERCC6
BCL2L11
TMEM223
GPR31
NCAPG
ANKRD5
SULT1B1
SLC13A2
RNU6-73
GH2
CXCR5
LSM14B
MYT1
MFI2
NPAS1
GLRA2
ACOT11
EPX
FGF21
PCDH7
EIF5A2
TRO
GLRB
TNFRSF9
PSG9
CGREF1
TRIM10
CCNE2
PPP1R1A
APOOL
HGD
FOXJ3
NPY
WNT3
CHRM2
AMH
ANKRD26
RP11-429B14.4
TACR1
GCK
HTR5A
SLC6A11
PTGDR2

ATOH1
VTCN1
SEC14L2
SATB2
CARTPT
AKAP3
TNFSF9
OPRL1
ALDH3B2
TAF13
PRR7
DHX9
EPB41
SSX2
RAB33A
FOXN1
ZNF107
CTB-31N19.2
GDNF
CCDC88C
SLC6A7
POM121L2
OIP5
NMRK2
HAO2
GDAP1L1
EIF4G3
ZBP1
ALLC
SLC6A6
CTRB2
AQP8
PODXL2
CIDEA
H2AFB2
PCDHA5
KRT33A
SERPINB5
PBK
FGB
PDIA2
GUCY1B2
NEUROD4
MAS1
GRK1
ADRB3

CENPQ
OVOL3
CACNA1S
CDX2
TLE6
CGA
WDR67
TRIM46
TCL6
GUCA2B
KRT2
CCDC70
QSER1
IL36A
ZNF587
CDX4
SLC13A3
SPAST
HOXA11
OR10H3
CHRNE
SLC22A6
HCN2
LUZP2
NR2E3
RNMT
POLRMTP1
KRT76
CTNNA3
ZFHX4
ATP2B3
IL1RAP
HELLS
DKK4
RAC3
ADAMTS7
TRAV10
IFNA21
MCF2L-AS1
TSPY2
CNGA1
BPI
OPCML
ABCC2
GRIN2D
SCLY

GUF1
TMEM121
SCT
HOXD13
SCN4A
GJB5
L2HGDH
ZFR2
NR5A1
NEU2
HIST1H3D
GPR3
FGF16
AC009299.3
HOXA1
PDYN
OSBP2
NTSR2
DEPDC1
FBXO11
RAB27B
GDF3
SLC28A1
TTK
LHX5
DLX5
HMP19
CHD5
RBFOX1
RP11-930P14.2
PAEP
CSAG3
KCNAB3
FGF17
MEGF8
ZNF816
FAM169A
KRT14
HLCS
CTAG1A
ERVMER34-1
SERPINA6
NEUROG1
RP5-886K2.1
MKRN7P
UMOD

RFX7
ZNF85
OTOR
POU2F2
PIPOX
NPPB
PDE6A
TWIST1
OR1D2
KIAA0913
LECT1
OXTR
GABRQ
HLA-DPA2
RBP3
MIR600HG
SHCBP1
SLC4A7
PPP1R37
RANBP1
CACNG2
DLX2
MAML3
ZBTB32
OPRD1
MSANTD3-TMEFF1
RP3-470B24.5
MTHFSD
LMX1B
BARX1
CYP2C18
OR7E14P
INSRR
RP11-79P5.2
DNAJC22
MYCN
HTR1A
RET
CST1
PRG3
ZNF174
OR1G1
ALOXE3
LRP12
CRHR2
TMA16

LIM2
RP11-517A5.7
HNF1A
PTPRN
CDHR1
ANXA9
HSPA4L
BLZF1
HIST1H1A
MCM3AP-AS1
CCKBR
ZNF273
DLL3
FAM198B
UPK2
HFE
RP5-955M13.3
ADAM30
PRL
CCL1
FCER2
MAGEA10
GSN-AS1
AIPL1
ERCC6L
FRMPD1
BRD7P3
NRXN2
REG1P
FUT6
SLC22A13
IL11
ATAD5
GPR37L1
KIF15
ELSPBP1
PDE11A
AC002310.7
NELL1
RP1-223E5.4
MYH7
FOXL1
KIF1A
HIST1H4E
SSX5
CCL25

KIAA1045
GATA4
ITGB3
RP1-101G11.2
SLC28A3
NEFH
GPR173
SSTR4
CUZD1
CRH
AC012074.2
MYH13
YOD1
LY6G6F
DNASE1
CLPS
MCM10
PRLR
LCN1
CASP2
GJA3
AC013271.3
QTRTD1
BMP3
HIST1H2BC
FAM131B
INCENP
HOXD10
TLX1
PRDM8
ERO1LB
FAM182B
PHF8
SHOX
PLA2G2F
CPLX2
KRT6A
ATP4A
ZNRD1-AS1
TWISTNB
SLC22A2
VWA7
HCG9
PITX1
SOX21
NXPE4

AC092653.5
KRT19P2
BLK
HRK
CA6
RP1-190J20.2
COL8A1
AGMAT
SLC22A11
TDRD12
GUCA1A
KISS1
OR2H1
IL1RL2
CINP
OR12D2
ROR2
HCRT
CNTNAP2
PTK6
CLCA1
PGAM2
ANTXR1
GHRHR
KLHL11
FCRL2
SLC18A3
CACNA1B
ZFP30
VAX2
TULP1
DCT
FOXD4L1
PPARA
RP6-24A23.6
NEURL
AC124309.1
LCT
PMS1
PTMAP1
SLC17A1
KIF25
HTR1B
PLEKHM1
HS3ST3A1
HAND2

CASC5
CKM
KCNJ14
HTR2B
EYA3
TP53AIP1
TRIM31
ANXA13
QPCTL
ALX4
CHRNA4
UGT8
BRIP1
TECTA
GHRH
ESM1
FBXO24
PIWIL2
SLC12A5
HTR1E
FGF23
GAL
DPF3
SSX8
HES2
NF2
WISP1
UNC13A
FAM163A
CACNG3
REG1A
SERPINB4
ALPI
ADAM5
MMP13
HSD17B1
TNFSF11
GAGE4
AC011385.1
UGT2B15
GLYAT
GNL3LP1
CROT
GPR37
GINS4
OR1A1

KRT18P38
CRNN
ACE2
SPRR1A
ELOVL2
CDY2B
PLG
MS4A5
CCL7
EPHB1
GOT2P1
CDHR5
CEP152
P2RY10
TPP2
RP11-217B7.2
CDH18
HIST1H2BG
CEL
GPR87
HOXD11
ALOX12B
TUBBP5
SRD5A3
FOXC2
SCG2
GPR1
SYT5
DUTP1
BEAN1
KCNJ10
RP1-136J15.3
PTPRZ1
HIST1H1E
SLC34A1
PGGT1B
ASLP1
CFHR2
SSX6
RIBC2
CDH7
KCNA10
KCNA2
MBTD1
HIST2H4A
LMO1

FDPSP5
DPYSL4
ATP6V0A4
TRMU
PON1
ABP1
NEB
TEX28P2
ZNF223
MPP6
ALOX12
CRX
IGSF1
SLC12A3
OR10C1
CDRT1
CNNM1
CXCL6
PPFIA4
SLC52A1
BCL2L10
CABP2
TNP1
SSTR2
CLDN14
CRYBA4
KIF23
HIST1H3J
PLA2G2E
WNT8B
DENND5B
PRIM2
RP11-84C10.2
HOXB13
RP13-221M14.5
POLR3G
DLG2
SSTR5
C8A
KRT38
AVP
CHGB
NEIL3
SLC9A3
ALDOB
PAGE1

SARDH
ORC1
MANEA
ZNF280B
ANKRD17
GABRA4
ZDHHC8P1
CCNK
PCDHA9
OPN1SW
SST
AC099522.1
SLC7A10
LOR
CHEK1
ADAM29
IGKV1D-8
PTTG3P
SLC22A7
RP11-44F21.3
GML
MYBPC2
AK5
TMPRSS11E
SOCS7
ATP2A1
PGK2
FAM5B
CRABP1
ZNF280A
CALML5
HOXA10
ESR2
GRM7
LIPC
ACSM1
WT1
AL645730.2
HRG
DNMT3L
PAX1
STMN4
ONECUT2
TUBB7P
TEX11
MMP16

DESI2
RP11-673C5.4
SLC22A1
SHANK1
CYP11A1
LEP
OBP2A
SNX16
FSHB
IL17B
RP4-734P14.4
CYCSP33
DRD4
MAG
RPLP2P1
PDX1
HIST1H4H
NUS1P2
PAX7
FCF1
STYK1
BMP10
MSX2
SLC9A7
SPRR1B
KCNJ9
MAGEA3
FABP2
PPY
MTAP
ONECUT1
CACNA1E
HIST1H2AE
SPAG11B
MYCNOS
GABRP
RP4-724E16.2
TNNI1
SAA3P
HTR7
MC5R
ATP2B2
PCDHB1
KCNH6
CADM4
TLX2

KRT18P50
ACHE
TCP11
ACSM2A
GPR25
OPRM1
CDC25C
CEACAM7
FGF3
WNT16
FHP1
PAX2
KCNJ1
TTTY1
CENPI
RP13-210D15.1
SLC14A2
CDK5R1
LGALS14
MYF6
KRTAP1-3
KCNH1
FBN2
HMGA2
PIK3C2G
GPR135
KRT3
CRISP1
GNAT1
MYL1
BPY2
PKP2
TCP10
NKX3-2
RP5-916L7.1
EFNA2
SIX3
SLC6A5
S100A7A
GAP43
ACTL7B
CDK5R2
HIST1H2AB
ZFX
KIF14
SLC28A2

CDC6
TCEB1P3
TRPM3
TTTY9B
FSHR
ANXA10
ELAVL4
TTYH1
HRASLS
ST8SIA2
RP11-731F5.1
LINC00563
ADARB2
CYP2R1
ULBP1
APCS
CCR8
GRM3
MAPK4
SERPINA10
PROL1
CD28
VSX1
IL24
NR2C2
FGF5
AP3B2
OR12D3
ZNF750
KRT31
PPEF1
LGALS13
CYP24A1
GNG13
TRPA1
NUP62CL
TEX13A
RBM12B-AS1
TSSK1B
XYLB
RHBDL1
HYAL4
NR2E1
NR0B1
ST20
DMC1

KALP
SMARCE1P2
BFSP2
PSG5
OMP
PDHA2
ATAD2B
SERPINB7
GRM8
NKX6-1
OR2H2
OR7C1
AC004478.2
APOBEC1
AFP
ROPN1
IRX4
TACR3
MAGEB4
CRP
GRIA4
FOXI1
GAD1
HTR1D
OR52A1
POF1B
TPO
POU3F2
DIAPH3
INSL4
ETV2
KIF25-AS1
ALX1
CSH2
IL36RN
KLF1
TUBB2B
GRIK3
DLX6
AC092811.1
PSG2
ATP8B3
CYP19A1
INS
DDX25
AL590822.1

SOX3
MTRF1
EGF
NPHS1
FOXE1
RPGRIP1L
CNGB3
MUC6
KCNJ4
CLCA2
AC112502.1
SCN2A
SERPINB10
IL36G
GNRHR
RPS6KA6
PPP1R2P9
MED18
MMP10
AVPR1A
CRYGB
KRT13
AC104699.1
OR2F1
TSHB
APOC3
MTL5
ZNF81
GABRA1
DOCK3
DCAF17
OCLM
RBPJL
MYH7B
RAB3B
CAPN6
ZNF556
SNTG1
ZIC1
CDH17
PAGE4
RP11-706O15.3
E2F8
UCP1
RBL1
EPHA7

MEP1B
TRHR
TSKS
CCDC15
SLITRK5
FOXE3
PAX3
S100G
PHF14
EGR4
CPB1
GHSR
ST8SIA3
TRAV8-3
FABP7
ADH7
PMCH
KIAA1549L
MC4R
CRYGA
SCN8A
UBQLN3
SERPINB13
CATSPERG
GABBR2
REG3A
CSHL1
SLC2A4
POLH
SNHG3
GPX5
FZD3
SLC39A9
KLK15
TMEM144
IGHV3-72
PPYR1
MPL
TRPM8
HIST3H3
GAD2
ATP1A3
GPR6
SLCO5A1
TRAV21
AC132216.1

NTRK1
SLC45A2
RP1-20N2.2
LRTM1
CHRNA9
FIP1L1
BCMO1
SOX11
HMGB1P12
GPR50
SLC18A1
CTD-3185P2.1
OPN1LW
AC008103.5
ADAM21
KCNA1
CALCB
GFAP
GAPDHS
PAX9
KRT34
HIST1H2BL
TTTY2B
TRBV10-2
SPIN2A
SMPX
DLK1
CNIH3
STAR
SMG7-AS1
NCAN
GALR2
DUSP21
PLSCR2
TSPY13P
AC015871.1
SIM2
ASIP
PHOX2B
MMP3
ZNF391
NEUROG3
WNT1
CTAG2
DSC3
LPA

SLC12A1
AL356585.3
GRM1
DSPP
RP11-158D1.1
GRID2
DEFA5
TAAR3
OR2B6
TP73
RP3-359N14.1
LINC00115
RP11-1007I13.3
PRM2
IFNA7
FGF22
PTHLH
OR7E19P
ZIC3
PKDREJ
OR1A2
FBXO40
HAPLN1
HERC2P5
MAGEC2
RP11-678B3.2
MOBP
DEFB126
FN3K
CALY
CYP2C19
MC3R
ZNF117
SLC35E2
RP11-118B18.1
GPR45
TLX3
IFNA4
CYLC1
TSSK2
SPC25
RAD51B
INSL5
HOXA9
STMN2
KRT37

KCNJ3
IL12RB2
HESX1
GRIP1
GRIK1
AD001527.5
MCF2
IL1RAPL1
SLC6A15
PRTN3
MAGEA12
GPR63
NTN3
RP11-101E3.5
THPO
NPY2R
OR1F2P
MSI1
TEX15
KNG1
LBX1
IGHV3-47
RP11-1084J3.3
HOXD3
DNTT
KIF18A
KRT36
DAZ1
AC004893.11
TUBAL3
LECT2
WDR76
RBMY2FP
POU6F2
POU3F3
GSG1
RP3-334F4.2
IL21
TH
BTN1A1
CTNNA2
PCSK1
TRIM9
SMR3A
TBL1Y
FAM205B

ISL1
FSCN2
GUCY2F
OR51E2
SLC9A2
MLL2
POU3F4
CHRNA5
MAGEC1
RP1-142F18.2
CBLN1
GPR75
AHSG
EMX1
SRY
AC084219.3
CPN2
AL359757.1
RALYL
LHX1
IGLL1
ZNF214
DCHS2
DSCR6
AC007401.1
GIP
ATRNL1
CSN3
RFPL1-AS1
NEFL
REG1B
LY6G6E
AL136531.1
FRK
TBR1
FOXB1
TRBV21-1
FABP3P2
CRCT1
RP11-770J1.4
PA2G4P2
WDHD1
LHX2
FOXD2
ZNF702P
KCNIP2

MYBPC1
ERC2
KRT35
CLGN
COL19A1
MEP1A
SIM1
SIX6
RP11-568A7.1
OR1E1
HEATR3
SOHLH2
ZPBP
RGS20
CRYBA2
IFNB1
B3GALT5
RIMBP2
GLP2R
GBX2
AKAP5
TRAV9-2
SCN11A
CCDC132
ASCL1
BRSK2
AKAP4
KHDC1L
ALDOAP2
ITGB8
BBOX1
CATSPERB
IGHV5-51
MTNR1A
RP11-707M1.1
DNAJC28
IGHV3-73
HIST1H1B
ELOVL4
PRSS1
KRT8P17
NEFM
SERPINC1
AMELY
PLEKHH3
ZIC4

OR3A1
NHLH2
SULT2A1
CDH8
TREH
FAM75A4
NRTN
AICDA
DGKI
ANP32C
COL2A1
OPRK1
HSD3B2
SOX1
GS1-124K5.9
LINC00302
TRAV16
CFHR3
PARPBP
GABRA5
GLRA1
LGSN
NPPC
P2RX3
PPBPP2
EN2
MAGEA1
AC110619.2
ASMT
GPR77
RNF17
PRKAR1AP
KRT24
SPAM1
AC114546.1
SP3P
CSRP3
RLN2
HIST1H4A
TNFSF15
RP11-119F7.5
INSL6
LINC00474
GNAT2
RBBP9
KCNK13

TAS2R4
CYP7B1
IMPG2
SNAP91
LPAL2
ELL2P3
CTAGE11P
NRAP
GKN1
HNRNPA1P31
HOXB8
GABRA3
OR2J1
MMP27
F2RL2
CA10
LRIT1
DRD3
SYCP2
ADAM7
CLIC1P1
PTH2R
AGAP2
LCE2B
POU4F1
AC006293.3
SPRR2C
KCNK12
SHOX2
MLANA
ABCB11
B3GALT1
FMO6P
SLC35F5
HIST1H4G
SCRT1
HIST1H3B
TDRD1
RLN1
TAF7L
HHLA2
DHRS2
HIST1H2BM
KRT18P27
APOA2
JPH3

MYO3A
RP3-522P13.1
AANAT
RXFP3
NOL4
TMEM156
PCDHGA11
TAS2R16
SCGB1D2
ABCG5
NLGN4Y
PRDM9
GIF
GPR22
MYL2
RP11-526J3.3
TRPC7
HRH4
ELAVL2
TM4SF20
GUCY2C
MKRN3
DIAPH2-AS1
HIST1H3C
CNN3P1
ADAM18
KCNJ13
SPOCK3
MAGEC3
RP5-1032F13.1
CYP4F11
OR10H2
PEX5L
RP3-497J21.1
ADCY10
TFAP2B
GRIA3
POU2F3
FOXL2
MAGEA2
PRDM13
RP3-508D13.1
GCM1
FGF8
HIST1H2BB
HIST1H4B

MS4A12
BMP8A
CYP4F8
BRS3
KRT12
KCND2
PCDHB8
HNF4G
BRCA2
BTF3P13
STXBP5L
PRPS1L1
ARPP21
KCNK2
INSM1
AC007359.6
FCN2
EN1
RP11-1220K2.2
TAS2R8
PNLIP
HSD3B1
OR2J3
ZNF192
KCNV1
CLCA4
CNGA3
GAGE1
ZNF492
HIST1H2AG
HIST1H2AL
HIST1H3I
RP11-714L20.1
GREB1
CSN1S1
MAGEA8
PMCHL2
PBOV1
NMBR
IKZF3
LIN28A
HIST1H2BJ
IAPP
RP3-336K20__B.2
AP003471.1
KIAA1024

MYH4
MAGEB2
EDDM3A
BNC1
CDH20
NPBWR2
RP4-630C24.3
IGFBP1
MYH8
MIA2
DSC1
PCDHA6
DDX4
SLCO1B3
HIST1H3A
ABCA12
POU4F2
MAGEB3
AMELX
PTPRJ
RP11-64J4.2
HCRTR1
UTS2
RP5-1116H23.1
NDP
HIST1H2APS4
MMP8
NEUROD1
GLRA3
HMGB3P19
ACSL6
TBC1D22B
AC024940.1
SYCP1
GS1-103B18.1
TSPY7P
OLFM4
NDST3
TAS2R9
GAN
EPHA5
OR2F2
HOXD12
GRIA2
ST18
SUMO1P2

RP1-217P22.2
NEUROG2
ANP32D
SAGE1
FABP1
MAGEA4
MYH6
FEZF2
RGS7
CALB1
GABRR1
SCGB1D1
RFX4
VPREB1
DMRT1
GS1-600G8.3
IL3
SKA1
GABRG3
POU4F3
SOX5P
WISP3
SLCO1B1
OR7A10
ZNF695
PAH
FUT9
ZNF643
HAVCR1
MYF5
UGT2A1
HIST1H2BO
FGF20
LPAR4
PITX2
MLNR
HIST1H4L
KRT20
GABRB1
MAGEB1
HMGB3P30
ARHGAP11A
UNC93A
TRAV13-2
RP1-6P5.2
RP11-15P13.1

KCNV2
AKR1C4
HEXA-AS1
LDHAL6B
TRAV13-1
IL4
ANGPTL3
AL136115.1
STATH
MTMR7
GABRA6
NPVF
MAGEA11
MRPS11P1
IGHV5-78
LRAT
RPL23AP22
TSHR
IFNA6
HIST1H3F
ZNF749
TARDBPP1
SORCS3
DAZL
PIWIL1
FOXD3
PMP2
NKX2-2
AGXT2L1
OR7E24
BRDT
PCK1
ATP8A2P3
ADAMTS20
TRPM1
GC
TPTE
AC021106.1
RBBP4P4
CDH12
RP13-225O21.3
TIGD6
EPYC
LINC00483
GPR88
RP11-69I8.2

ZNF257
PDZK1P1
ARL14
INHBE
ALB
PPIAL4A
FETUB
GCG
RP1-263J7.2
GNAT3
IFNA2
FAM75C1
SLC38A4
GSC2
ACTR3P2
LHCGR
HTR2C
PNLIPRP2
UGT2B17
RP11-430B1.2
TRIM49
EDDM3B
GYS2
ANKRD7
CHRNB4
HTN1
SOX30
DGKB
KIAA0087
MUC7
SLC5A12
MBL2
AC002994.1
DSG1
BTF3P11
OR2J2
ABCD2
VENTXP1
AC064852.4
GNGT1
DMP1
F13B
SCGB2A2
DIRAS2
KERA
SLC25A21

SCG3
ADAM2
ADAM3A
ZNF962P
BX004987.4
FLG
AC007742.7
SSX4
IL2
KRTAP9-9
RNF11B
USH2A
RP11-361M4.1
SEMG2
RFPL1
IL12B
RP6-149D17.1
IMPG1
SALL1
PCDH8
GCM2
RP11-286E11.2
SSX1
HMGB1P17
RP11-61A14.4
IFNA8
AKR1D1
PCNAP3
MORC1
RP1-28C20.1
AC098817.5
CLCA3P
SLC13A1
RPE65
POU1F1

Supplementary Table S4. Pathways/Processes significantly enriched (FDR ≤ 0.05) in Alzheimer's disease, lung cancer and glioblastoma according to GSEA analyses.

AD up
immune response
defense response
response to other organism
immune system process
response to virus
cellular defense response
inflammatory response
regulation of i kappab kinase nf kappab cascade
hematopoietic cell lineage
cytokine cytokine receptor interaction
graft versus host disease
allograft rejection
autoimmune thyroid disease
complement and coagulation cascades
ecm receptor interaction
intestinal immune network for iga production
cell adhesion molecules cams
asthma
nod like receptor signaling pathway
apoptosis
b cell receptor signaling pathway
leishmania infection
notch signaling pathway
viral myocarditis
integrin cell surface interactions
immunoregulatory interactions between a lymphoid and a non lymphoid cell
yap1 and wwtr1 taz stimulated gene expression
interferon alpha beta signaling
interferon gamma signaling
traf6 mediated irf7 activation
traf6 mediated nfkb activation
rig i mda5 mediated induction of ifn alpha beta pathways
cell surface interactions at the vascular wall
chemokine receptors bind chemokines
complement cascade
pre notch transcription and translation
innate immune system
gpvi mediated activation cascade
tak1 activates nfkb by phosphorylation and activation of ikks complex
integrin alphaiib beta3 signaling

AD down
mrna processing go 0006397
trna metabolic process
cofactor metabolic process
protein folding
rna splicing
protein catabolic process
cellular carbohydrate metabolic process
mrna metabolic process
cellular respiration
coenzyme metabolic process
cellular protein catabolic process
rna processing
ribonucleoprotein complex biogenesis and assembly
macromolecule catabolic process
carbohydrate metabolic process
transmission of nerve impulse
protein amino acid lipidation
spliceosome assembly
synaptic transmission
nucleobasenucleoside and nucleotide metabolic process
neurogenesis
rna splicingvia transesterification reactions
biopolymer catabolic process
catabolic process
lipoprotein biosynthetic process
generation of neurons
secretory pathway
neuron differentiation
cellular catabolic process
protein amino acid dephosphorylation
double strand break repair
amino sugar metabolic process
golgi vesicle transport
energy derivation by oxidation of organic compounds
cellular macromolecule catabolic process
protein rna complex assembly
dephosphorylation
amine metabolic process
nervous system development
glucose metabolic process
mitochondrion organization and biogenesis
ubiquitin cycle
oxidative phosphorylation
parkinsons disease
huntingtons disease
alzheimers disease
aminoacyl trna biosynthesis
proteasome

citrate cycle tca cycle
cardiac muscle contraction
vibrio cholerae infection
rna polymerase
alanine aspartate and glutamate metabolism
riboflavin metabolism
purine metabolism
spliceosome
terpenoid backbone biosynthesis
glutathione metabolism
rna degradation
ubiquitin mediated proteolysis
glycosylphosphatidylinositol gpi anchor biosynthesis
oocyte meiosis
nucleotide excision repair
pyruvate metabolism
pyrimidine metabolism
n glycan biosynthesis
protein export
glycosaminoglycan biosynthesis heparan sulfate
tca cycle and respiratory electron transport
respiratory electron transport atp synthesis by chemiosmotic coupling and heat production by uncoupling proteins
respiratory electron transport
mitochondrial protein import
trafficking of ampa receptors
autodegradation of cdh1 by cdh1 apc c
insulin receptor recycling
scf beta trcp mediated degradation of emi1
trna aminoacylation
cytosolic trna aminoacylation
apc c cdc20 mediated degradation of mitotic proteins
trafficking of glur2 containing ampa receptors
pyruvate metabolism and citric acid tca cycle
cdt1 association with the cdc6 orc origin complex
regulation of mitotic cell cycle
antigen processing ubiquitination proteasome degradation
prefoldin mediated transfer of substrate to cct tric
cross presentation of soluble exogenous antigens endosomes
transferrin endocytosis and recycling
transmission across chemical synapses
cdk mediated phosphorylation and removal of cdc6
apc c cdh1 mediated degradation of cdc20 and other apc c cdh1 targeted proteins in late mitosis early g1
host interactions of hiv factors
scfskp2 mediated degradation of p27 p21
p53 independent g1 s dna damage checkpoint
hiv infection
vif mediated degradation of apobec3g
autodegradation of the e3 ubiquitin ligase cop1
assembly of the pre replicative complex

destabilization of mrna by auf1 hnrnp d0
citric acid cycle tca cycle
signaling by wnt
protein folding
neuronal system
cell cycle checkpoints
regulation of apoptosis
rna pol iii chain elongation
nef mediates down modulation of cell surface receptors by recruiting them to clathrin adapters
mrna splicing minor pathway
cyclin e associated events during g1 s transition
activation of nf kappab in b cells
m g1 transition
neurotransmitter receptor binding and downstream transmission in the postsynaptic cell
rna pol iii transcription initiation from type 3 promoter
mrna capping
synthesis of dna
orc1 removal from chromatin
neurotransmitter release cycle
s phase
rna pol iii transcription termination
formation of tubulin folding intermediates by cct tric
the role of nef in hiv1 replication and disease pathogenesis
endosomal sorting complex required for transport escrt
g1 s transition
post chaperonin tubulin folding pathway
p53 dependent g1 dna damage response
late phase of hiv life cycle
cholesterol biosynthesis
mrna processing
voltage gated potassium channels
glutamate neurotransmitter release cycle
formation of the hiv1 early elongation complex
activation of nmda receptor upon glutamate binding and postsynaptic events
mitotic m m g1 phases
asparagine n linked glycosylation
rna pol ii transcription
metabolism of proteins
class i mhc mediated antigen processing presentation
rna pol iii transcription initiation from type 2 promoter
metabolism of non coding rna
pyruvate metabolism
iron uptake and transport
dna replication
interactions of vpr with host cellular proteins
hiv life cycle
regulation of mrna stability by proteins that bind au rich elements
metabolism of amino acids and derivatives
unblocking of nmda receptor glutamate binding and activation

er phagosome pathway
post nmda receptor activation events
rna pol iii transcription
ras activation uopn ca2 infux through nmda receptor
processing of capped intron containing pre mrna
mhc class ii antigen presentation
rna pol ii transcription pre initiation and promoter opening
cell cycle mitotic
latent infection of homo sapiens with mycobacterium tuberculosis
gluconeogenesis
metabolism of vitamins and cofactors
mrna splicing
mitotic g1 g1 s phases
formation of rna pol ii elongation complex
transport of mature transcript to cytoplasm
gaba synthesis release reuptake and degradation
rna pol ii pre transcription events
ligand gated ion channel transport
mitochondrial trna aminoacylation
downstream signaling events of b cell receptor bcr
formation of transcription coupled ner tc ner repair complex
recruitment of mitotic centrosome proteins and complexes
cleavage of growing transcript in the termination region
g alpha z signalling events
membrane trafficking
dna repair
activation of atr in response to replication stress
potassium channels
triglyceride biosynthesis
transcription coupled ner tc ner
biosynthesis of the n glycan precursor dolichol lipid linked oligosaccharide llo and transfer to a nascent protein
darpp 32 events
apc cdc20 mediated degradation of nek2a
ca dependent events
rna pol i transcription termination
nucleotide excision repair
peroxisomal lipid metabolism
hibition of the proteolytic activity of apc c required for the onset of anaphase by mitotic spindle checkpoint componen
phosphorylation of the apc c
opioid signalling
gaba receptor activation

**LC up**
m phase
m phase of mitotic cell cycle
mitosis
cell cycle process
mitotic cell cycle
cell cycle phase
regulation of mitosis
protein folding
dna metabolic process
sister chromatid segregation
dna repair
mitotic sister chromatid segregation
response to dna damage stimulus
mitotic cell cycle checkpoint
meiosis i
base excision repair
mitochondrion organization and biogenesis
response to endogenous stimulus
meiotic cell cycle
dna damage responsesignal transduction
amino acid and derivative metabolic process
dna integrity checkpoint
regulation of cell cycle
microtubule cytoskeleton organization and biogenesis
dna replication
double strand break repair
meiotic recombination
nucleobasenucleoside and nucleotide metabolic process
mitochondrial transport
ectoderm development
regulation of cyclin dependent protein kinase activity
dna recombination
nuclear export
rna processing
amino acid metabolic process
amine catabolic process
dna catabolic process
er to golgi vesicle mediated transport
apoptotic program
dna dependent dna replication
amino acid catabolic process
apoptotic nuclear changes
regulation of mitotic cell cycle
dna replication initiation
nucleotide metabolic process
epidermis development
one carbon compound metabolic process
nitrogen compound catabolic process

cell cycle
dna replication
base excision repair
aminoacyl trna biosynthesis
proteasome
one carbon pool by folate
homologous recombination
o glycan biosynthesis
rna polymerase
glutathione metabolism
cysteine and methionine metabolism
arginine and proline metabolism
alanine aspartate and glutamate metabolism
p53 signaling pathway
n glycan biosynthesis
citrate cycle tca cycle
pentose phosphate pathway
oxidative phosphorylation
mismatch repair
dna replication
cell cycle mitotic
cell cycle
mitotic m m g1 phases
g2 m checkpoints
cell cycle checkpoints
dna strand elongation
mitotic prometaphase
activation of atr in response to replication stress
mitotic g1 g1 s phases
s phase
g1 s transition
m g1 transition
deposition of new cenpa containing nucleosomes at the centromere
synthesis of dna
dna repair
assembly of the pre replicative complex
e2f mediated regulation of dna replication
activation of the pre replicative complex
kinesins
gluconeogenesis
telomere maintenance
g1 s specific transcription
chromosome maintenance
metabolism of non coding rna
apc c cdh1 mediated degradation of cdc20 and other apc c cdh1 targeted proteins in late mitosis early g1
trna aminoacylation
rna pol i promoter opening
rna pol i transcription
transcription coupled ner tc ner

metabolism of nucleotides
glucose metabolism
extracellular matrix organization
hiv life cycle
formation of the hiv1 early elongation complex
resolution of ap sites via the multiple nucleotide patch replacement pathway
p53 independent g1 s dna damage checkpoint
base excision repair
glycolysis
packaging of telomere ends
tca cycle and respiratory electron transport
rna pol i rna pol iii and mitochondrial transcription
double strand break repair
collagen formation
biosynthesis of the n glycan precursor dolichol lipid linked oligosaccharide llo and transfer to a nascent protein
purine metabolism
late phase of hiv life cycle
lagging strand synthesis
autodegradation of the e3 ubiquitin ligase cop1
metabolism of proteins
transcription
destabilization of mrna by auf1 hnrnp d0
transport of ribonucleoproteins into the host nucleus
autodegradation of cdh1 by cdh1 apc c
mitotic g2 g2 m phases
regulation of ornithine decarboxylase odc
nucleotide excision repair
metabolism of amino acids and derivatives
post translational protein modification
cdk mediated phosphorylation and removal of cdc6
interactions of vpr with host cellular proteins
respiratory electron transport
abortive elongation of hiv1 transcript in the absence of tat
cyclin e associated events during g1 s transition
formation of transcription coupled ner tc ner repair complex
asparagine n linked glycosylation
homologous recombination repair of replication independent double strand breaks
respiratory electron transport atp synthesis by chemiosmotic coupling and heat production by uncoupling proteins
p53 dependent g1 dna damage response
scf beta trcp mediated degradation of emi1
scfskp2 mediated degradation of p27 p21
transport of mature mrna derived from an intronless transcript
formation of rna pol ii elongation complex
nep ns2 interacts with the cellular export machinery
rna pol ii pre transcription events
cross presentation of soluble exogenous antigens endosomes
vif mediated degradation of apobec3g
apc cdc20 mediated degradation of nek2a
regulation of glucokinase by glucokinase regulatory protein

hiv infection
mrna splicing minor pathway
meiosis
cytosolic trna aminoacylation
mitochondrial protein import
o linked glycosylation of mucins
mrna capping
amyloids
olfactory signaling pathway
amino acid synthesis and interconversion transamination
mrna processing
metabolism of carbohydrates
metabolism of rna
pyruvate metabolism and citric acid tca cycle
global genomic ner gg ner
rna pol ii transcription pre initiation and promoter opening
processing of capped intronless pre mrna
processing of capped intron containing pre mrna
er phagosome pathway
activation of chaperone genes by xbp1s
microrna mirna biogenesis
influenza life cycle
elongation arrest and recovery
meiotic synapsis
srp dependent cotranslational protein targeting to membrane
intrinsic pathway for apoptosis
rna pol ii transcription
glucose transport
recruitment of mitotic centrosome proteins and complexes
mhc class ii antigen presentation
ncam1 interactions

ts

LC down
defense response
immune response
immune system process
inflammatory response
transforming growth factor beta receptor signaling pathway
response to wounding
cell migration
transmembrane receptor protein serine threonine kinase signaling pathway
response to external stimulus
locomotory behavior
cellular defense response
cytokine and chemokine mediated signaling pathway
hemopoiesis
muscle development
hemopoietic or lymphoid organ development
leukocyte migration
axon guidance
cell recognition
response to other organism
enzyme linked receptor protein signaling pathway
regulation of g protein coupled receptor protein signaling pathway
regulation of cytoskeleton organization and biogenesis
behavior
innate immune response
actin cytoskeleton organization and biogenesis
response to bacterium
protein kinase cascade
cytokine secretion
ras protein signal transduction
vascular smooth muscle contraction
graft versus host disease
hematopoietic cell lineage
leishmania infection
viral myocarditis
chemokine signaling pathway
endocytosis
cytokine cytokine receptor interaction
natural killer cell mediated cytotoxicity
complement and coagulation cascades
allograft rejection
leukocyte transendothelial migration
nod like receptor signaling pathway
mapk signaling pathway
cell adhesion molecules cams
prion diseases
regulation of actin cytoskeleton
adipocytokine signaling pathway
dilated cardiomyopathy

ppar signaling pathway
aldosterone regulated sodium reabsorption
jak stat signaling pathway
hypertrophic cardiomyopathy hcm
smooth muscle contraction
muscle contraction
netrin1 signaling
platelet activation signaling and aggregation
immunoregulatory interactions between a lymphoid and a non lymphoid cell
complement cascade
interferon gamma signaling
signaling by rho gtpases
response to elevated platelet cytosolic ca2
semaphorin interactions
signaling by ils
sema4d in semaphorin signaling
sema4d induced cell migration and growth cone collapse
hemostasis
other semaphorin interactions
effects of pip2 hydrolysis
innate immune system
cell surface interactions at the vascular wall
regulation of kit signaling
g alpha1213 signalling events
growth hormone receptor signaling
il 3 5 and gm csf signaling
nitric oxide stimulates guanylate cyclase
pi 3k cascade
phospholipase c mediated cascade
chemokine receptors bind chemokines
signaling by scf kit
myd88 mal cascade initiated on plasma membrane
pi3k events in erbb4 signaling
toll receptor cascades

**GBM up**
positive regulation of i kappab kinase nf kappab cascade
response to wounding
positive regulation of signal transduction
cell cycle go 0007049
cell cycle phase
response to other organism
m phase
mitotic cell cycle checkpoint
regulation of mitosis
dna metabolic process
m phase of mitotic cell cycle
mitotic cell cycle
response to virus
mitosis
regulation of cell cycle
interphase
chromosome segregation
response to stress
meiosis i
regulation of phosphorylation
dna repair
response to dna damage stimulus
blood coagulation
jak stat cascade
dna integrity checkpoint
response to endogenous stimulus
viral myocarditis
p53 signaling pathway
dna replication
ecm receptor interaction
homologous recombination
glycosaminoglycan degradation
small cell lung cancer
base excision repair
nucleotide excision repair
interferon alpha beta signaling
interferon signaling
dna strand elongation
dna replication
g2 m checkpoints
extracellular matrix organization
cell cycle
deposition of new cenpa containing nucleosomes at the centromere
activation of the pre replicative complex
mitotic m m g1 phases
cell cycle mitotic
mitotic g1 g1 s phases
s phase

cell cycle checkpoints
activation of atr in response to replication stress
g1 s transition
g1 s specific transcription
transport of ribonucleoproteins into the host nucleus
antiviral mechanism by ifn stimulated genes
cell surface interactions at the vascular wall
synthesis of dna
innate immune system
global genomic ner gg ner
meiosis
homologous recombination repair of replication independent double strand breaks
e2f mediated regulation of dna replication
smad2 smad3 smad4 heterotrimer regulates transcription
meiotic recombination
regulation of glucokinase by glucokinase regulatory protein
rig i mda5 mediated induction of ifn alpha beta pathways
double strand break repair
antigen processing cross presentation
nep ns2 interacts with the cellular export machinery
translation
metabolism of nucleotides

GBM down
synaptic transmission
transmission of nerve impulse
potassium ion transport
monovalent inorganic cation transport
metal ion transport
central nervous system development
generation of a signal involved in cell cell signaling
glutamate signaling pathway
system process
g protein signaling coupled to cyclic nucleotide second messenger
cyclic nucleotide mediated signaling
establishment and or maintenance of cell polarity
generation of neurons
neuron differentiation
cell cell signaling
cardiac muscle contraction
oxidative phosphorylation
neuroactive ligand receptor interaction
phosphatidylinositol signaling system
gnrh signaling pathway
neuronal system
transmission across chemical synapses
neurotransmitter receptor binding and downstream transmission in the postsynaptic cell
neurotransmitter release cycle
potassium channels
gaba synthesis release reuptake and degradation
regulation of insulin secretion
darpp 32 events
effects of pip2 hydrolysis
ngf signalling via trka from the plasma membrane
g alpha s signalling events
phospholipase c mediated cascade
g alpha z signalling events
creb phosphorylation through the activation of ras
activation of kainate receptors upon glutamate binding
platelet homeostasis
l1cam interactions

Supplementary Table S5. Numbers of significantly Differentially Expressed Genes (sDEGs) annotated in overlapping pathways in pairwise comparisons.

Disease pairwise comparisons	Number of sDEGs annotated in overlapping pathways	Number of sDEGs in pairwise overlapping pathways	Percentage of sDEGs annotated in pairwise overlapping pathways
LC-/AD+	315	1031	30.55%
LC+/AD-	553	1934	28.59%
GBM+/AD-	34	289	11.76%
GBM-/AD-	208	1003	20.74%
GBM+/AD+	192	807	23.79%
GBM-/LC-	11	692	1.58%
GBM+/LC-	231	896	25.78%
GBM+/LC+	364	1058	34.4%

Supplementary Table S6. Pathways/Processes significantly enriched (FDR ≤ 0.05) in AD-/LC+ brain-specific sDEGs and in AD+/LC- lung-specific sDEGs using gProfileR.

**AD-/LC+ brain-specific genes**
sensory perception of pain
glutamate receptor signaling pathway
neurofilament cytoskeleton organization
neurofilament bundle assembly
dendrite extension
regulation of dendrite extension
positive regulation of dendrite extension
adenylate cyclase-inhibiting G-protein coupled receptor signaling pathway
signaling
regulation of biological quality
regulation of membrane potential
regulation of postsynaptic membrane potential
action potential
regulation of neurotransmitter levels
multicellular organismal process
system process
regulation of system process
neurological system process
cognition
neuromuscular process
vesicle-mediated transport in synapse
behavior
feeding behavior
locomotory behavior
localization
establishment of localization
transport
ion transport
cation transport
metal ion transport
transmembrane transport
ion transmembrane transport
inorganic ion transmembrane transport
inorganic cation transmembrane transport
single-organism process
single-organism localization
single-organism transport
neurotransmitter transport
secretion
single organism signaling
single-multicellular organism process
multicellular organismal signaling
multicellular organismal response to stress
single-organism behavior
adult behavior

learning or memory
memory
learning
associative learning
conditioned taste aversion
single-organism cellular process
cell communication
cell-cell signaling
synaptic signaling
trans-synaptic signaling
anterograde trans-synaptic signaling
chemical synaptic transmission
presynaptic process involved in chemical synaptic transmission
neuron-neuron synaptic transmission
synaptic transmission, glutamatergic
transmission of nerve impulse
neuronal action potential
cell projection organization
synapse organization
secretion by cell
signal release
signal release from synapse
neurotransmitter secretion
glutamate secretion
synaptic vesicle cycle
regulation of localization
regulation of transport
regulation of transmembrane transport
regulation of ion transport
regulation of ion transmembrane transport
regulation of secretion
cellular component morphogenesis
cell morphogenesis
cell part morphogenesis
cell projection morphogenesis
regulation of secretion by cell
regulation of exocytosis
vesicle localization
synaptic vesicle localization
establishment of synaptic vesicle localization
synaptic vesicle transport
head development
system development
nervous system development
central nervous system development
limbic system development

cell development
brain development
hindbrain development
calcium ion regulated exocytosis
regulation of calcium ion-dependent exocytosis
synaptic vesicle exocytosis
calcium ion-regulated exocytosis of neurotransmitter
neurogenesis
generation of neurons
metencephalon development
neuron differentiation
neuron development
neuron projection development
neuron projection morphogenesis
cell morphogenesis involved in neuron differentiation
modulation of synaptic transmission
regulation of synaptic plasticity
regulation of neuronal synaptic plasticity
regulation of synaptic transmission, glutamatergic
chemical synaptic transmission, postsynaptic
excitatory postsynaptic potential
positive regulation of synaptic transmission
G-protein coupled receptor signaling pathway
neuropeptide signaling pathway
cyclic nucleotide metabolic process
regulation of cyclic nucleotide metabolic process
regulation of cyclic nucleotide biosynthetic process
negative regulation of nucleotide metabolic process
negative regulation of purine nucleotide metabolic process
negative regulation of cyclic nucleotide metabolic process
negative regulation of nucleotide biosynthetic process
negative regulation of purine nucleotide biosynthetic process
negative regulation of cyclic nucleotide biosynthetic process
regulation of cAMP metabolic process
negative regulation of cAMP metabolic process
Gastric acid secretion
cAMP signaling pathway
Serotonergic synapse
Cholinergic synapse
Taste transduction
Glutamatergic synapse
Retrograde endocannabinoid signaling
Long-term potentiation
Dopaminergic synapse
Calcium signaling pathway
GABAergic synapse

Amphetamine addiction
Nicotine addiction
Circadian entrainment
Neuroactive ligand-receptor interaction
Morphine addiction
Inhibition of voltage gated Ca2+ channels via Gbeta/gamma subunits
Neuronal System
Potassium Channels
Voltage gated Potassium channels
Inwardly rectifying K+ channels
G protein gated Potassium channels
Activation of G protein gated Potassium channels
Transmission across Chemical Synapses
Neurotransmitter Release Cycle
Glutamate Neurotransmitter Release Cycle
Neurotransmitter Receptor Binding And Downstream Transmission In The Postsynaptic Cell
Glutamate Binding, Activation of AMPA Receptors and Synaptic Plasticity
Trafficking of AMPA receptors
GABA receptor activation
Activation of NMDA receptor upon glutamate binding and postsynaptic events
Unblocking of NMDA receptor, glutamate binding and activation
Muscle contraction
Cardiac conduction
G alpha (i) signalling events
CREB phosphorylation through the activation of CaMKII
Ras activation uopn Ca2+ infux through NMDA receptor
Signaling by GPCR
GPCR ligand binding

**AD+/LC- lung-specific genes**
B cell receptor signaling pathway
immune system process
immune effector process
leukocyte degranulation
immune system development
localization
localization of cell
establishment of localization
biological adhesion
cell adhesion
cell-cell adhesion
signaling
response to stimulus
response to chemical
response to organic substance
response to cytokine
response to lipid
response to external stimulus
response to stress
defense response
inflammatory response
cellular defense response
response to wounding
response to biotic stimulus
response to external biotic stimulus
response to molecule of fungal origin
immune response
adaptive immune response
innate immune response
response to interferon-gamma
multicellular organismal process
locomotion
taxis
chemotaxis
single-organism process
single organism signaling
single organism cell adhesion
single organismal cell-cell adhesion
leukocyte cell-cell adhesion
leukocyte aggregation
lymphocyte aggregation
cell proliferation
leukocyte proliferation
mononuclear cell proliferation
single-multicellular organism process

cytokine production
interleukin-3 production
single-organism localization
single-organism transport
secretion
single-organism cellular process
cell activation
leukocyte activation
myeloid leukocyte activation
mast cell activation
lymphocyte activation
lymphocyte proliferation
T cell aggregation
T cell activation
alpha-beta T cell activation
NK T cell activation
T cell proliferation
alpha-beta T cell proliferation
cell activation involved in immune response
leukocyte activation involved in immune response
myeloid cell activation involved in immune response
mast cell activation involved in immune response
mast cell degranulation
lymphocyte activation involved in immune response
T cell activation involved in immune response
ell activation via T cell receptor contact with antigen bound to MHC molecule on antigen presenting
movement of cell or subcellular component
cell motility
cell migration
leukocyte migration
myeloid leukocyte migration
granulocyte migration
neutrophil migration
cell communication
secretion by cell
multi-organism process
interspecies interaction between organisms
symbiosis, encompassing mutualism through parasitism
response to other organism
response to bacterium
response to molecule of bacterial origin
response to lipopolysaccharide
cellular response to lipopolysaccharide
defense response to other organism
defense response to bacterium
defense response to virus

peptidyl-tyrosine modification
biological regulation
regulation of biological process
regulation of localization
regulation of response to stimulus
regulation of response to external stimulus
regulation of response to stress
regulation of defense response
regulation of inflammatory response
negative regulation of biological process
positive regulation of biological process
positive regulation of response to stimulus
positive regulation of defense response
positive regulation of response to external stimulus
positive regulation of metabolic process
regulation of multicellular organismal process
negative regulation of multicellular organismal process
positive regulation of multicellular organismal process
regulation of cytokine production
positive regulation of cytokine production
regulation of immune system process
regulation of immune effector process
negative regulation of immune system process
positive regulation of immune system process
regulation of immune response
positive regulation of immune response
activation of immune response
regulation of innate immune response
positive regulation of innate immune response
activation of innate immune response
regulation of signaling
positive regulation of signaling
regulation of locomotion
positive regulation of locomotion
regulation of chemotaxis
positive regulation of chemotaxis
regulation of cellular process
positive regulation of cellular process
regulation of cell proliferation
regulation of leukocyte proliferation
regulation of mononuclear cell proliferation
regulation of cell activation
regulation of leukocyte activation
regulation of lymphocyte activation
regulation of lymphocyte proliferation
positive regulation of cell activation

positive regulation of leukocyte activation
regulation of cell communication
positive regulation of cell communication
regulation of cellular component movement
regulation of cell motility
regulation of cell migration
regulation of leukocyte migration
regulation of neutrophil migration
positive regulation of leukocyte migration
positive regulation of neutrophil migration
regulation of cell adhesion
positive regulation of cell adhesion
regulation of cell-cell adhesion
regulation of leukocyte cell-cell adhesion
regulation of T cell activation
regulation of T cell proliferation
regulation of protein modification process
multi-organism cellular process
viral process
cellular response to stimulus
cellular response to chemical stimulus
cellular response to organic substance
cellular response to cytokine stimulus
cellular response to interferon-gamma
cell chemotaxis
leukocyte chemotaxis
regulation of leukocyte chemotaxis
granulocyte chemotaxis
neutrophil chemotaxis
regulation of neutrophil chemotaxis
positive regulation of leukocyte chemotaxis
positive regulation of granulocyte chemotaxis
positive regulation of neutrophil chemotaxis
cellular response to biotic stimulus
cellular response to molecule of fungal origin
signal transduction
cell surface receptor signaling pathway
integrin-mediated signaling pathway
cytokine-mediated signaling pathway
interferon-gamma-mediated signaling pathway
intracellular signal transduction
I-kappaB kinase/NF-kappaB signaling
regulation of signal transduction
regulation of intracellular signal transduction
immune response-regulating signaling pathway
immune response-regulating cell surface receptor signaling pathway

immune response-activating signal transduction
innate immune response-activating signal transduction
pattern recognition receptor signaling pathway
toll-like receptor signaling pathway
MyD88-dependent toll-like receptor signaling pathway
positive regulation of signal transduction
positive regulation of intracellular signal transduction
positive regulation of macromolecule metabolic process
negative regulation of cell activation
negative regulation of leukocyte activation
negative regulation of lymphocyte activation
vesicle-mediated transport
endocytosis
phagocytosis
regulation of developmental process
phosphorylation
protein phosphorylation
peptidyl-tyrosine phosphorylation
signal transduction by protein phosphorylation
MAPK cascade
extracellular structure organization
extracellular matrix organization
cell differentiation
animal organ development
hematopoietic or lymphoid organ development
hemopoiesis
leukocyte differentiation
regulation of leukocyte differentiation
lymphocyte differentiation
T cell differentiation
positive regulation of transport
regulation of phosphorus metabolic process
regulation of phosphate metabolic process
regulation of phosphorylation
regulation of protein phosphorylation
regulation of peptidyl-tyrosine phosphorylation
regulation of MAPK cascade
negative regulation of cell adhesion
negative regulation of cell-cell adhesion
negative regulation of leukocyte cell-cell adhesion
negative regulation of T cell activation
negative regulation of cell proliferation
negative regulation of leukocyte proliferation
negative regulation of mononuclear cell proliferation
negative regulation of lymphocyte proliferation
regulation of interferon-gamma production

positive regulation of cellular metabolic process
positive regulation of phosphorus metabolic process
positive regulation of phosphate metabolic process
positive regulation of phosphorylation
positive regulation of interleukin-8 production
positive regulation of leukocyte cell-cell adhesion
protein secretion
cytokine secretion
regulation of cytokine secretion
positive regulation of protein phosphorylation
positive regulation of MAPK cascade
cardiovascular system development
vasculature development
blood vessel development
blood vessel morphogenesis
angiogenesis
granulocyte macrophage colony-stimulating factor biosynthetic process
acute inflammatory response to antigenic stimulus
calcium-mediated signaling
Tuberculosis
Rheumatoid arthritis
Cell adhesion molecules (CAMs)
Viral myocarditis
Staphylococcus aureus infection
Inflammatory bowel disease (IBD)
Natural killer cell mediated cytotoxicity
NOD-like receptor signaling pathway
Malaria
Legionellosis
Hematopoietic cell lineage
Fc epsilon RI signaling pathway
Cytokine-cytokine receptor interaction
Osteoclast differentiation
B cell receptor signaling pathway
TNF signaling pathway
Immune System
Innate Immune System
Cytokine Signaling in Immune system
Interferon Signaling
Interferon gamma signaling
Chemokine receptors bind chemokines
Hemostasis
Cell surface interactions at the vascular wall
Integrin cell surface interactions

Supplementary Table S7. Overlaps between significantly differentially expressed genes (sDEGs) in Alzheimer's disease (AD) and glioblastoma (GBM) using healthy and epilepsy control samples separately.

Pairwise comparisons of sDEGs identified as significantly up- and down-regulated (FDR≤0.05) after gene expression meta-analyses in AD and GBM using different control samples. Green cells indicate significant overlaps between sDEGs in the same direction (Fisher's exact test, FDR≤0.05). White cells correspond to non-significant overlaps (FDR>0.05).

	**Healthy**	
	**GBM down**	**GBM up**
**AD up**	1	4.07e-180
**AD down**	2.07e-192	1

	**Epilepsy**	
	**GBM down**	**GBM up**
**AD up**	1	6.52e-143
**AD down**	2.15e-115	1

Supplementary Table S8. Genes described to be known bear causative mutations in GBM by PMID:24120142 present in the overlaps between genes significantly differentially expressed in AD-GBM and LC-GBM comparisons.

GBM+/AD+	TP53	EGFR	RB1	STAG2	COL1A2
GBM+/AD-	TMEM147				
GBM-/AD-	CDH18	CDH9	DYNC1I1	GABRA1	
GBM+/LC+	TP53	EGFR	IDH1	COL1A2	TMEM147
GBM+/LC-	RB1				
GBM-/LC+	CDH18	DRD5	DYNC1I1	GABRA1	
GBM-/LC-	GABRB2				

Supplementary Table S9. Datasets used for studying Alzheimer's disease (AD), lung cancer (LC), glioblastoma (GB) and brain control samples necessary for differential expression analyses in GB. Disease name and their corresponding IDs, tissues, platforms, sample numbers and references are indicated for each of the datasets.

**Disease**	**Dataset ID**	**Tissue**	**Platform**	**Online repository**	**Sample numbers (case/control)**	**Reference**
Alzheimer's disease	GSE5281	Entorhinal Cortex (Brain)	HG_U133_Plus2	GEO	**23** (10/13)	Liang *et al*., 2007
		Hippocampus (Brain)			**23** (10/13)	
		Medial Temporal Gyrus (Brain)			**28** (16/12)	
		Posterior Cingulate (Brain)			**22** (9/13)	
		Superior Frontal Gyrus (Brain)			**34** (23/11)	
		Primary Visual Cortex (Brain)			**31** (19/12)	
	GSE48350	Entorhinal Cortex (Brain)			**53** (14/39)	Blair *et al*., 2013
		Hippocampus (Brain)			**62** (19/43)	
		Posterior Cingulate (Brain)			**68** (25/43)	
		Superior Frontal Gyrus (Brain)			**69** (21/48)	
	GSE4757	Brain			**20** (10/10)	Dunckley *et al*., 2006
Glioblastoma	GSE4290	Brain	HG_U133_Plus2	GEO	(26/-)	Sun *et al*., 2006
	GSE15824				(12/-)	Grzmil *et al*., 2011
	GSE13041				(27/-)	Lee et al., 2008
	GSE53733				(70/-)	Reifenberger et al., 2014
	E MTAB 1852			ArrayExpress	(11/-)	Choy et al., 2013
	TCGA		HT_HG_U133A	TCGA	**528** (518/10)	
Brain Controls	GSE54567	Dorsolateral Prefrontal Cortex (frontal lobe)	HG_U133_Plus2	GEO	(-/14)	Chang *et al*., 2014
	GSE54571	Anterior Cingulate Cortex (frontal lobe)			(-/13)	
	GSE11882	Entorhinal Cortex (temporal lobe)			(-/39)	
		Post-central Gyrus (parietal lobe)			(-/43)	
	GSE7307	Occipital-lobe			(-/8)	*Citation missing*
	GSE4290	Brain (Epilepsy)			(-/23)	Sun *et al*., 2006
Lung Cancer	GSE33532	NSCLC	HG_U133_Plus2	GEO	**40** (20/20) x4 regions	Meister *et al*., 2014
	GSE19188				**144** (94/50)	Hou *et al*., 2010
	GSE19804				**120** (60/60)	Lu *et al*., 2010
	GSE7670		HG_U133A		**54** (27/27)	Su et al., 2007
	GSE10072				**107** (58/49)	Landi et al., 2008

Text mining

Abstracts of papers studying AD, GBM and LC in PubMed were analyzed (see queries bellow) looking for genes mentioned on each of them.

Query for PubMed's AD papers:

((((("Alzheimer Disease"[Mesh] OR "Alzheimer's disease antigen"[Supplementary Concept] OR "APP protein, human"[Supplementary Concept] OR "PSEN2 pro- tein, human"[Supplementary Concept] OR "PSEN1 protein, human"[Supplementary Concept]) OR "Amyloid beta-Peptides"[Mesh]) OR "donepezil"[Supplementary Concept]) OR ("gamma-secretase activating protein, human"[Supplementary Concept] OR "gamma-secretase activating protein, mouse"[Supplementary Concept])) OR "amy- loid beta-protein (1-42)"[Supplementary Concept]) OR "Presenilins"[Mesh]) OR "Neu- ro brillary Tangles"[Mesh] OR "Alzheimer's disease"[All Fields] OR "Alzheimer's Disease"[All Fields] OR "Alzheimer s disease"[All Fields] OR "Alzheimers disease"[All Fields] OR "Alzheimer's dementia"[All Fields] OR "Alzheimer dementia"[All Fields] OR "Alzheimer-type dementia"[All Fields] NOT "non-Alzheimer"[All Fields] NOT ("non-AD"[All Fields] AND "dementia"[All Fields]))

Query for PubMed's GBM papers:

"glioblastoma"[Mesh] OR glioblastoma OR Glioblastomas OR "Astrocytoma, Grade IV" OR "Grade IV Astrocytoma" OR "Astrocytomas, Grade IV" OR "Grade IV AstrocytomaS" OR "Grade IV Astrocytoma" OR "Grade IV Astrocytomas" OR "Glioblastoma Multiforme" OR "Giant Cell Glioblastoma" OR "Giant Cell Glioblastomas" OR "Glioblastoma, Giant Cell" OR "Glioblastomas, Giant Cell" OR "glioblastoma multiforme" OR "glioblastoma multiforme" OR "Glioblastoma multiforme" OR "glioblastomas multiforme" OR "glioblastoma multiform" OR "Glioblastoma Multiforme" OR "glioblastoma multiformes" OR "Glioblastoma multiform" OR "Glioblastoma multiforma" OR "Glioblastomas multiforme" OR "glioblastoma multiforma" OR "grade IV astrocytoma" OR "grade four astrocytoma" OR "grade 4 astrocytoma" OR "glial cell tumor" OR "glial cell tumour" OR "malignant neoplasm of glial cells" OR "glioblastom" OR "glioblastome" OR "multiforme glioblastoma"

Query for PubMed's LC papers:

(((["Carcinoma, Non-Small-Cell Lung"[Mesh]] OR ["lung cancer" and "non-small cell"] OR "acinar predominant adenocarcinoma of the lung" OR "Acinar predominant adenocarcinoma of the lung" OR "ACINAR PREDOMINANT ADENOCARCINOMA OF THE LUNG" OR "acinar predominant adenocarcinoma of the lungs" OR "Acinar predominant adenocarcinoma of the lungs" OR "ACINAR PREDOMINANT ADENOCARCINOMA OF THE LUNGs" OR "ACINAR PREDOMINANT ADENOCARCINOMA OF THE LUNGS" OR "adenocarcinoma in situ of the lung" OR "Adenocarcinoma in situ of the lung" OR "ADENOCARCINOMA IN SITU OF THE LUNG" OR "adenocarcinoma in situ of the lungs" OR "adenocarcinoma in situ of the lungS" OR "Adenocarcinoma in situ of the lungs" OR "ADENOCARCINOMA IN SITU OF THE LUNGs" OR "ADENOCARCINOMA IN SITU OF THE LUNGS" OR "adenocarcinoma of the lung" OR "Adenocarcinoma of the lung" OR "ADENOCARCINOMA OF THE LUNG" OR "adenocarcinoma of

the lungs" OR "Adenocarcinoma of the lungs" OR "Adenocarcinoma of the lungS" OR "ADENOCARCINOMA OF THE LUNGs" OR "ADENOCARCINOMA OF THE LUNGS" OR "adenosquamous carcinoma of the lung" OR "Adenosquamous carcinoma of the lung" OR "ADENOSQUAMOUS CARCINOMA OF THE LUNG" OR "adenosquamous carcinoma of the lungs" OR "Adenosquamous carcinoma of the lungs" OR "Adenosquamous carcinoma of the lungS" OR "ADENOSQUAMOUS CARCINOMA OF THE LUNGs" OR "ADENOSQUAMOUS CARCINOMA OF THE LUNGS" OR "bronchioalveolar carcinoma" OR "Bronchioalveolar carcinoma" OR "BRONCHIOALVEOLAR CARCINOMA" OR "bronchioalveolar carcinomas" OR "bronchioalveolar carcinomaS" OR "Bronchioalveolar carcinomas" OR "Bronchioalveolar carcinomaS" OR "BRONCHIOALVEOLAR CARCINOMAs" OR "BRONCHIOALVEOLAR CARCINOMAS" OR "bronchioalveolar lung carcinoma" OR "Bronchioalveolar lung carcinoma" OR "BRONCHIOALVEOLAR LUNG CARCINOMA" OR "bronchioalveolar lung carcinomas" OR "bronchioalveolar lung carcinomaS" OR "Bronchioalveolar lung carcinomas" OR "Bronchioalveolar lung carcinomaS" OR "BRONCHIOALVEOLAR LUNG CARCINOMAs" OR "BRONCHIOALVEOLAR LUNG CARCINOMAS" OR "bronchioloalveolar carcinoma" OR "Bronchioloalveolar carcinoma" OR "BRONCHIOLOALVEOLAR CARCINOMA" OR "bronchioloalveolar carcinomas" OR "bronchioloalveolar carcinomaS" OR "Bronchioloalveolar carcinomas" OR "Bronchioloalveolar carcinomaS" OR "BRONCHIOLOALVEOLAR CARCINOMAs" OR "BRONCHIOLOALVEOLAR CARCINOMAS" OR "carcinoma, non-small cell lung" OR "Carcinoma, non-small cell lung" OR "Carcinoma, Non-Small Cell Lung" OR "CARCINOMA, NON-SMALL CELL LUNG" OR "carcinoma, non-small cell lungs" OR "carcinoma, non-small cell lungS" OR "Carcinoma, non-small cell lungs" OR "Carcinoma, non-small cell lungS" OR "Carcinoma, Non-Small Cell Lungs" OR "Carcinoma, Non-Small Cell LungS" OR "CARCINOMA, NON-SMALL CELL LUNGs" OR "CARCINOMA, NON-SMALL CELL LUNGS" OR "cribriform adenocarcinoma lungs" OR "Cribriform adenocarcinoma lungs" OR "CRIBRIFORM ADENOCARCINOMA LUNGS" OR "cribriform adenocarcinoma of the lung" OR "Cribriform adenocarcinoma of the lung" OR "CRIBRIFORM ADENOCARCINOMA OF THE LUNG" OR "cribriform adenocarcinoma of the lungs" OR "cribriform adenocarcinoma of the lungS" OR "Cribriform adenocarcinoma of the lungs" OR "Cribriform adenocarcinoma of the lungS" OR "CRIBRIFORM ADENOCARCINOMA OF THE LUNGs" OR "CRIBRIFORM ADENOCARCINOMA OF THE LUNGS" OR "enteric adenocarcinoma" OR "Enteric adenocarcinoma" OR "ENTERIC ADENOCARCINOMA" OR "enteric adenocarcinoma of lungs" OR "Enteric adenocarcinoma of lungs" OR "ENTERIC ADENOCARCINOMA OF LUNGS" OR "enteric adenocarcinoma of the lung" OR "Enteric adenocarcinoma of the lung" OR "ENTERIC ADENOCARCINOMA OF THE LUNG" OR "enteric adenocarcinoma of the lungs" OR "enteric adenocarcinoma of the lungS" OR "Enteric adenocarcinoma of the lungs" OR "Enteric adenocarcinoma of the lungS" OR "ENTERIC ADENOCARCINOMA OF THE LUNGs" OR "ENTERIC ADENOCARCINOMA OF THE LUNGS" OR "enteric adenocarcinomas" OR "enteric adenocarcinomaS" OR "Enteric adenocarcinomas" OR "Enteric adenocarcinomaS" OR "ENTERIC ADENOCARCINOMAs" OR "ENTERIC ADENOCARCINOMAS" OR "epidermoid cancers of the lung" OR "Epidermoid cancers of the lung"

OR "EPIDERMOID CANCERS OF THE LUNG" OR "epidermoid cancers of the lungs" OR "epidermoid cancers of the lungS" OR "Epidermoid cancers of the lungs" OR "Epidermoid cancers of the lungS" OR "EPIDERMOID CANCERS OF THE LUNGs" OR "EPIDERMOID CANCERS OF THE LUNGS" OR "epidermoid lung cancer" OR "Epidermoid lung cancer" OR "EPIDERMOID LUNG CANCER" OR "epidermoid lung cancers" OR "epidermoid lung cancerS" OR "Epidermoid lung cancers" OR "Epidermoid lung cancerS" OR "EPIDERMOID LUNG CANCERs" OR "EPIDERMOID LUNG CANCERS" OR "epidermoid lung carcinoma" OR "Epidermoid lung carcinoma" OR "EPIDERMOID LUNG CARCINOMA" OR "epidermoid lung carcinomas" OR "epidermoid lung carcinomaS" OR "Epidermoid lung carcinomas" OR "Epidermoid lung carcinomaS" OR "EPIDERMOID LUNG CARCINOMAs" OR "EPIDERMOID LUNG CARCINOMAS" OR "invasive mucinous adenocarcinoma of the lung" OR "Invasive mucinous adenocarcinoma of the lung" OR "INVASIVE MUCINOUS ADENOCARCINOMA OF THE LUNG" OR "invasive mucinous adenocarcinoma of the lungs" OR "invasive mucinous adenocarcinoma of the lungS" OR "Invasive mucinous adenocarcinoma of the lungs" OR "Invasive mucinous adenocarcinoma of the lungS" OR "INVASIVE MUCINOUS ADENOCARCINOMA OF THE LUNGs" OR "INVASIVE MUCINOUS ADENOCARCINOMA OF THE LUNGS" OR "large cell lung carcinoma" OR "Large cell lung carcinoma" OR "LARGE CELL LUNG CARCINOMA" OR "large cell lung carcinomas" OR "large cell lung carcinomaS" OR "Large cell lung carcinomas" OR "Large cell lung carcinomaS" OR "LARGE CELL LUNG CARCINOMAs" OR "LARGE CELL LUNG CARCINOMAS" OR "lung acinar predominant adenocarcinoma" OR "Lung acinar predominant adenocarcinoma" OR "LUNG ACINAR PREDOMINANT ADENOCARCINOMA" OR "lung acinar predominant adenocarcinomas" OR "lung acinar predominant adenocarcinomaS" OR "Lung acinar predominant adenocarcinomas" OR "Lung acinar predominant adenocarcinomaS" OR "LUNG ACINAR PREDOMINANT ADENOCARCINOMAs" OR "LUNG ACINAR PREDOMINANT ADENOCARCINOMAS" OR "lung adenocarcinoma" OR "Lung adenocarcinoma" OR "LUNG ADENOCARCINOMA" OR "lung adenocarcinoma in situ" OR "Lung adenocarcinoma in situ" OR "LUNG ADENOCARCINOMA IN SITU" OR "lung adenocarcinoma in situs" OR "lung adenocarcinoma in situS" OR "Lung adenocarcinoma in situs" OR "Lung adenocarcinoma in situS" OR "LUNG ADENOCARCINOMA IN SITUs" OR "LUNG ADENOCARCINOMA IN SITUS" OR "lung adenocarcinomas" OR "lung adenocarcinomaS" OR "Lung adenocarcinomas" OR "Lung adenocarcinomaS" OR "LUNG ADENOCARCINOMAs" OR "LUNG ADENOCARCINOMAS" OR "lung adenosquamous carcinoma" OR "Lung adenosquamous carcinoma" OR "LUNG ADENOSQUAMOUS CARCINOMA" OR "lung adenosquamous carcinomas" OR "lung adenosquamous carcinomaS" OR "Lung adenosquamous carcinomas" OR "Lung adenosquamous carcinomaS" OR "LUNG ADENOSQUAMOUS CARCINOMAs" OR "LUNG ADENOSQUAMOUS CARCINOMAS" OR "lung invasive mucinous adenocarcinoma" OR "Lung invasive mucinous adenocarcinoma" OR "LUNG INVASIVE MUCINOUS ADENOCARCINOMA" OR "lung invasive mucinous adenocarcinomas" OR "lung invasive mucinous adenocarcinomaS" OR "Lung invasive mucinous adenocarcinomas" OR "Lung invasive mucinous adenocarcinomaS" OR "LUNG INVASIVE MUCINOUS ADENOCARCINOMAs" OR "LUNG INVASIVE MUCINOUS

ADENOCARCINOMAS" OR "lung micropapillary predominant adenocarcinoma" OR "Lung micropapillary predominant adenocarcinoma" OR "LUNG MICROPAPILLARY PREDOMINANT ADENOCARCINOMA" OR "lung micropapillary predominant adenocarcinomas" OR "lung micropapillary predominant adenocarcinomaS" OR "Lung micropapillary predominant adenocarcinomas" OR "Lung micropapillary predominant adenocarcinomaS" OR "LUNG MICROPAPILLARY PREDOMINANT ADENOCARCINOMAs" OR "LUNG MICROPAPILLARY PREDOMINANT ADENOCARCINOMAS" OR "lung papillary predominant adenocarcinoma" OR "Lung papillary predominant adenocarcinoma" OR "LUNG PAPILLARY PREDOMINANT ADENOCARCINOMA" OR "lung papillary predominant adenocarcinomas" OR "lung papillary predominant adenocarcinomaS" OR "Lung papillary predominant adenocarcinomas" OR "Lung papillary predominant adenocarcinomaS" OR "LUNG PAPILLARY PREDOMINANT ADENOCARCINOMAs" OR "LUNG PAPILLARY PREDOMINANT ADENOCARCINOMAS" OR "lung sarcomatoid carcinoma" OR "Lung sarcomatoid carcinoma" OR "LUNG SARCOMATOID CARCINOMA" OR "lung sarcomatoid carcinomas" OR "lung sarcomatoid carcinomaS" OR "Lung sarcomatoid carcinomas" OR "Lung sarcomatoid carcinomaS" OR "LUNG SARCOMATOID CARCINOMAs" OR "LUNG SARCOMATOID CARCINOMAS" OR "lung scc" OR "lung SCC" OR "Lung scc" OR "LUNG SCC" OR "lung sccs" OR "lung sccS" OR "lung SCCs" OR "lung SCCS" OR "Lung sccs" OR "Lung sccS" OR "LUNG SCCs" OR "LUNG SCCS" OR "lung solid predominant adenocarcinoma" OR "Lung solid predominant adenocarcinoma" OR "LUNG SOLID PREDOMINANT ADENOCARCINOMA" OR "lung solid predominant adenocarcinomas" OR "lung solid predominant adenocarcinomaS" OR "Lung solid predominant adenocarcinomas" OR "Lung solid predominant adenocarcinomaS" OR "LUNG SOLID PREDOMINANT ADENOCARCINOMAs" OR "LUNG SOLID PREDOMINANT ADENOCARCINOMAS" OR "micropapillary predominant adenocarcinoma of the lung" OR "Micropapillary predominant adenocarcinoma of the lung" OR "MICROPAPILLARY PREDOMINANT ADENOCARCINOMA OF THE LUNG" OR "micropapillary predominant adenocarcinoma of the lungs" OR "micropapillary predominant adenocarcinoma of the lungS" OR "Micropapillary predominant adenocarcinoma of the lungs" OR "Micropapillary predominant adenocarcinoma of the lungS" OR "MICROPAPILLARY PREDOMINANT ADENOCARCINOMA OF THE LUNGs" OR "MICROPAPILLARY PREDOMINANT ADENOCARCINOMA OF THE LUNGS" OR "ncslc" OR "Ncslc" OR "NCSLC" OR "ncslcs" OR "ncslcS" OR "Ncslcs" OR "NcslcS" OR "NCSLCs" OR "NCSLCS" OR "non sclc" OR "non-sclc" OR "non SCLC" OR "non-SCLC" OR "Non sclc" OR "Non-sclc" OR "NON SCLC" OR "NON-SCLC" OR "non sclcs" OR "non-sclcs" OR "non sclcS" OR "non-sclcS" OR "non SCLCs" OR "non-SCLCs" OR "non SCLCS" OR "non-SCLCS" OR "Non sclcs" OR "Non-sclcs" OR "Non sclcS" OR "Non-sclcS" OR "NON SCLCs" OR "NON-SCLCs" OR "NON SCLCS" OR "NON-SCLCS" OR "non-small cell bronchial carcinoma" OR "non-small-cell bronchial carcinoma" OR "Non-small cell bronchial carcinoma" OR "Non-small-cell bronchial carcinoma" OR "NON-SMALL CELL BRONCHIAL CARCINOMA" OR "NON-SMALL-CELL BRONCHIAL CARCINOMA" OR "non-small cell bronchial carcinomas" OR "non-small-cell bronchial carcinomas" OR "non-small cell bronchial carcinomaS" OR "non-small-cell bronchial carcinomaS" OR "Non-small

cell bronchial carcinomas" OR "Non-small-cell bronchial carcinomas" OR "Non-small cell bronchial carcinomaS" OR "Non-small-cell bronchial carcinomaS" OR "NON-SMALL CELL BRONCHIAL CARCINOMAs" OR "NON-SMALL-CELL BRONCHIAL CARCINOMAs" OR "NON-SMALL CELL BRONCHIAL CARCINOMAS" OR "NON-SMALL-CELL BRONCHIAL CARCINOMAS" OR "non - small-cell lung cancer" OR "non small cell lung cancer" OR "non small cell-lung cancer" OR "non small-cell lung cancer" OR "non- small cell lung cancer" OR "non--small cell lung cancer" OR "non--small-cell lung cancer" OR "non-small cell lung cancer" OR "non-small cell-lung cancer" OR "non-small cell; lung cancer" OR "non-small-cell lung cancer" OR "non-small-cell lung-cancer" OR "non-small-cell-lung cancer" OR "non-small-cell-lung-cancer" OR "nonsmall cell lung cancer" OR "nonsmall-cell lung cancer" OR "non-small cell lung Cancer" OR "non-small-cell lung Cancer" OR "non-small-cell Lung cancer" OR "non-small-Cell lung cancer" OR "Non - small-cell lung cancer" OR "Non small cell lung cancer" OR "Non small cell-lung cancer" OR "Non small-cell lung cancer" OR "Non- small cell lung cancer" OR "Non--small cell lung cancer" OR "Non--small-cell lung cancer" OR "Non-small cell lung cancer" OR "Non-small cell-lung cancer" OR "Non-small cell; lung cancer" OR "Non-small-cell lung cancer" OR "Non-small-cell lung-cancer" OR "Non-small-cell-lung cancer" OR "Non-small-cell-lung-cancer" OR "Nonsmall cell lung cancer" OR "Nonsmall-cell lung cancer" OR "Non-small Cell Lung Cancer" OR "Nonsmall Cell Lung Cancer" OR "Non-Small cell Lung Cancer" OR "Non Small Cell Lung Cancer" OR "Non-Small Cell Lung Cancer" OR "Non-Small-Cell Lung Cancer" OR "Non-Small-Cell-Lung-Cancer" OR "NON - SMALL-CELL LUNG CANCER" OR "NON SMALL CELL LUNG CANCER" OR "NON SMALL CELL-LUNG CANCER" OR "NON SMALL-CELL LUNG CANCER" OR "NON- SMALL CELL LUNG CANCER" OR "NON--SMALL CELL LUNG CANCER" OR "NON--SMALL-CELL LUNG CANCER" OR "NON-SMALL CELL LUNG CANCER" OR "NON-SMALL CELL-LUNG CANCER" OR "NON-SMALL CELL; LUNG CANCER" OR "NON-SMALL-CELL LUNG CANCER" OR "NON-SMALL-CELL LUNG-CANCER" OR "NON-SMALL-CELL-LUNG CANCER" OR "NON-SMALL-CELL-LUNG-CANCER" OR "NONSMALL CELL LUNG CANCER" OR "NONSMALL-CELL LUNG CANCER" OR "non - small-cell lung cancers" OR "non small cell lung cancers" OR "non small cell-lung cancers" OR "non small-cell lung cancers" OR "non- small cell lung cancers" OR "non--small cell lung cancers" OR "non--small-cell lung cancers" OR "non-small cell lung cancers" OR "non-small cell-lung cancers" OR "non-small cell; lung cancers" OR "non-small-cell lung cancers" OR "non-small-cell lung-cancers" OR "non-small-cell-lung cancers" OR "non-small-cell-lung-cancers" OR "nonsmall cell lung cancers" OR "nonsmall-cell lung cancers" OR "non - small-cell lung cancerS" OR "non small cell lung cancerS" OR "non small cell-lung cancerS" OR "non small-cell lung cancerS" OR "non- small cell lung cancerS" OR "non--small cell lung cancerS" OR "non--small-cell lung cancerS" OR "non-small cell lung cancerS" OR "non-small cell-lung cancerS" OR "non-small cell; lung cancerS" OR "non-small-cell lung cancerS" OR "non-small-cell lung-cancerS" OR "non-small-cell-lung cancerS" OR "non-small-cell-lung-cancerS" OR "nonsmall cell lung cancerS" OR "nonsmall-cell lung cancerS" OR "non-small cell lung Cancers" OR "non-small-cell lung Cancers" OR "non-small cell lung CancerS" OR "non-small-cell lung CancerS" OR "non-small-cell Lung cancers" OR "non-small-cell Lung cancerS" OR "non-small-Cell lung

cancers" OR "non-small-Cell lung cancerS" OR "Non - small-cell lung cancers" OR "Non small cell lung cancers" OR "Non small cell-lung cancers" OR "Non small-cell lung cancers" OR "Non- small cell lung cancers" OR "Non--small cell lung cancers" OR "Non--small-cell lung cancers" OR "Non-small cell lung cancers" OR "Non-small cell-lung cancers" OR "Non-small cell; lung cancers" OR "Non-small-cell lung cancers" OR "Non-small-cell lung-cancers" OR "Non-small-cell-lung cancers" OR "Non-small-cell-lung-cancers" OR "Nonsmall cell lung cancers" OR "Nonsmall-cell lung cancers" OR "Non - small-cell lung cancerS" OR "Non small cell lung cancerS" OR "Non small cell-lung cancerS" OR "Non small-cell lung cancerS" OR "Non- small cell lung cancerS" OR "Non--small cell lung cancerS" OR "Non--small-cell lung cancerS" OR "Non-small cell lung cancerS" OR "Non-small cell-lung cancerS" OR "Non-small cell; lung cancerS" OR "Non-small-cell lung cancerS" OR "Non-small-cell lung-cancerS" OR "Non-small-cell-lung cancerS" OR "Non-small-cell-lung-cancerS" OR "Nonsmall cell lung cancerS" OR "Nonsmall-cell lung cancerS" OR "Non-small Cell Lung Cancers" OR "Nonsmall Cell Lung Cancers" OR "Non-small Cell Lung CancerS" OR "Nonsmall Cell Lung CancerS" OR "Non-Small cell Lung Cancers" OR "Non-Small cell Lung CancerS" OR "Non Small Cell Lung Cancers" OR "Non-Small Cell Lung Cancers" OR "Non-Small-Cell Lung Cancers" OR "Non-Small-Cell-Lung-Cancers" OR "Non Small Cell Lung CancerS" OR "Non-Small Cell Lung CancerS" OR "Non-Small-Cell Lung CancerS" OR "Non-Small-Cell-Lung-CancerS" OR "NON - SMALL-CELL LUNG CANCERs" OR "NON SMALL CELL LUNG CANCERs" OR "NON SMALL CELL-LUNG CANCERs" OR "NON SMALL-CELL LUNG CANCERs" OR "NON- SMALL CELL LUNG CANCERs" OR "NON--SMALL CELL LUNG CANCERs" OR "NON--SMALL-CELL LUNG CANCERs" OR "NON-SMALL CELL LUNG CANCERs" OR "NON-SMALL CELL-LUNG CANCERs" OR "NON-SMALL CELL; LUNG CANCERs" OR "NON-SMALL-CELL LUNG CANCERs" OR "NON-SMALL-CELL LUNG-CANCERs" OR "NON-SMALL-CELL-LUNG CANCERs" OR "NON-SMALL-CELL-LUNG-CANCERs" OR "NONSMALL CELL LUNG CANCERs" OR "NONSMALL-CELL LUNG CANCERs" OR "NON - SMALL-CELL LUNG CANCERS" OR "NON SMALL CELL LUNG CANCERS" OR "NON SMALL CELL-LUNG CANCERS" OR "NON SMALL-CELL LUNG CANCERS" OR "NON-SMALL CELL LUNG CANCERS" OR "NON--SMALL CELL LUNG CANCERS" OR "NON--SMALL-CELL LUNG CANCERS" OR "NON-SMALL CELL LUNG CANCERS" OR "NON-SMALL CELL-LUNG CANCERS" OR "NON-SMALL CELL; LUNG CANCERS" OR "NON-SMALL-CELL LUNG CANCERS" OR "NON-SMALL-CELL LUNG-CANCERS" OR "NON-SMALL-CELL-LUNG CANCERS" OR "NON-SMALL-CELL-LUNG-CANCERS" OR "NONSMALL CELL LUNG CANCERS" OR "NONSMALL-CELL LUNG CANCERS" OR "non small cell lung carcinoma" OR "non- small cell lung carcinoma" OR "non-small cell lung carcinoma" OR "non-small-cell lung carcinoma" OR "non-small-cell-lung-carcinoma" OR "nonsmall cell lung carcinoma" OR "nonsmall-cell lung carcinoma" OR "Non small cell lung carcinoma" OR "Non- small cell lung carcinoma" OR "Non-small cell lung carcinoma" OR "Non-small-cell lung carcinoma" OR "Non-small-cell-lung-carcinoma" OR "Nonsmall cell lung carcinoma" OR "Nonsmall-cell lung carcinoma" OR "Non Small Cell Lung Carcinoma" OR "Non-Small Cell Lung Carcinoma" OR "Non-Small-Cell Lung Carcinoma" OR "Non-Small-Cell-Lung-Carcinoma" OR "NON SMALL CELL LUNG CARCINOMA" OR "NON- SMALL CELL LUNG

CARCINOMA" OR "NON-SMALL CELL LUNG CARCINOMA" OR "NON-SMALL-CELL LUNG CARCINOMA" OR "NON-SMALL-CELL-LUNG-CARCINOMA" OR "NONSMALL CELL LUNG CARCINOMA" OR "NONSMALL-CELL LUNG CARCINOMA" OR "non small cell lung carcinomas" OR "non- small cell lung carcinomas" OR "non-small cell lung carcinomas" OR "non-small- cell lung carcinomas" OR "non-small-cell lung carcinomas" OR "non-small-cell-lung-carcinomas" OR "nonsmall cell lung carcinomas" OR "nonsmall-cell lung carcinomas" OR "non small cell lung carcinomaS" OR "non- small cell lung carcinomaS" OR "non-small cell lung carcinomaS" OR "non-small-cell lung carcinomaS" OR "non-small-cell-lung-carcinomaS" OR "nonsmall cell lung carcinomaS" OR "nonsmall-cell lung carcinomaS" OR "Non small cell lung carcinomas" OR "Non-small cell lung carcinomas" OR "Non-small cell lung carcinomas" OR "Non-small- cell lung carcinomas" OR "Non-small-cell lung carcinomas" OR "Non-small-cell-lung-carcinomas" OR "Nonsmall cell lung carcinomas" OR "Nonsmall-cell lung carcinomas" OR "Non small cell lung carcinomaS" OR "Non- small cell lung carcinomaS" OR "Non-small cell lung carcinomaS" OR "Non-small-cell lung carcinomaS" OR "Non-small-cell-lung-carcinomaS" OR "Nonsmall cell lung carcinomaS" OR "Nonsmall-cell lung carcinomaS" OR "Non Small Cell Lung Carcinomas" OR "Non-Small Cell Lung Carcinomas" OR "Non-Small-Cell Lung Carcinomas" OR "Non-Small-Cell-Lung-Carcinomas" OR "Non Small Cell Lung CarcinomaS" OR "Non-Small Cell Lung CarcinomaS" OR "Non-Small-Cell Lung CarcinomaS" OR "Non-Small-Cell-Lung-CarcinomaS" OR "NON SMALL CELL LUNG CARCINOMAs" OR "NON- SMALL CELL LUNG CARCINOMAs" OR "NON-SMALL CELL LUNG CARCINOMAs" OR "NON-SMALL-CELL LUNG CARCINOMAs" OR "NON-SMALL-CELL-LUNG-CARCINOMAs" OR "NONSMALL CELL LUNG CARCINOMAs" OR "NONSMALL-CELL LUNG CARCINOMAs" OR "NON SMALL CELL LUNG CARCINOMAS" OR "NON- SMALL CELL LUNG CARCINOMAS" OR "NON-SMALL CELL LUNG CARCINOMAS" OR "NON-SMALL- CELL LUNG CARCINOMAS" OR "NON-SMALL-CELL LUNG CARCINOMAS" OR "NON-SMALL-CELL-LUNG-CARCINOMAS" OR "NONSMALL CELL LUNG CARCINOMAS" OR "NONSMALL-CELL LUNG CARCINOMAS" OR "non small cells lung cancer" OR "non-small cells lung cancer" OR "Non small cells lung cancer" OR "Non-small cells lung cancer" OR "NON SMALL CELLS LUNG CANCER" OR "NON-SMALL CELLS LUNG CANCER" OR "non small cells lung cancers" OR "non-small cells lung cancers" OR "non small cells lung cancerS" OR "non-small cells lung cancerS" OR "Non small cells lung cancers" OR "Non-small cells lung cancers" OR "Non small cells lung cancerS" OR "Non-small cells lung cancerS" OR "NON SMALL CELLS LUNG CANCERs" OR "NON-SMALL CELLS LUNG CANCERs" OR "NON SMALL CELLS LUNG CANCERS" OR "NON-SMALL CELLS LUNG CANCERS" OR "nsclc" OR "n-sclc" OR "Nsclc" OR "N-sclc" OR "NSCLC" OR "N-SCLC" OR "nsclcs" OR "n-sclcs" OR "nsclcS" OR "n-sclcS" OR "Nsclcs" OR "N-sclcs" OR "NsclcS" OR "N-sclcS" OR "NSCLCs" OR "N-SCLCs" OR "NSCLCS" OR "N-SCLCS" OR "nslc" OR "Nslc" OR "NSLC" OR "nslcs" OR "nslcS" OR "Nslcs" OR "NslcS" OR "NSLCs" OR "NSLCS" OR "papillary predominant adenocarcinoma of the lung" OR "Papillary predominant adenocarcinoma of the lung" OR "PAPILLARY PREDOMINANT ADENOCARCINOMA OF THE LUNG" OR "papillary predominant adenocarcinoma of the lungs" OR "papillary predominant adenocarcinoma of the lungS" OR

"Papillary predominant adenocarcinoma of the lungs" OR "Papillary predominant adenocarcinoma of the lungS" OR "PAPILLARY PREDOMINANT ADENOCARCINOMA OF THE LUNGs" OR "PAPILLARY PREDOMINANT ADENOCARCINOMA OF THE LUNGS" OR "pulmonary adenocarcinoma" OR "Pulmonary adenocarcinoma" OR "PULMONARY ADENOCARCINOMA" OR "pulmonary adenocarcinomas" OR "pulmonary adenocarcinomaS" OR "Pulmonary adenocarcinomas" OR "Pulmonary adenocarcinomaS" OR "PULMONARY ADENOCARCINOMAs" OR "PULMONARY ADENOCARCINOMAS" OR "pulmonary cribriform adenocarcinoma" OR "Pulmonary cribriform adenocarcinoma" OR "PULMONARY CRIBRIFORM ADENOCARCINOMA" OR "pulmonary cribriform adenocarcinomas" OR "pulmonary cribriform adenocarcinomaS" OR "Pulmonary cribriform adenocarcinomas" OR "Pulmonary cribriform adenocarcinomaS" OR "PULMONARY CRIBRIFORM ADENOCARCINOMAs" OR "PULMONARY CRIBRIFORM ADENOCARCINOMAS" OR "pulmonary enteric adenocarcinoma" OR "Pulmonary enteric adenocarcinoma" OR "PULMONARY ENTERIC ADENOCARCINOMA" OR "pulmonary enteric adenocarcinomas" OR "pulmonary enteric adenocarcinomaS" OR "Pulmonary enteric adenocarcinomas" OR "Pulmonary enteric adenocarcinomaS" OR "PULMONARY ENTERIC ADENOCARCINOMAs" OR "PULMONARY ENTERIC ADENOCARCINOMAS" OR "pulmonary minimally invasive adenocarcinoma" OR "Pulmonary minimally invasive adenocarcinoma" OR "PULMONARY MINIMALLY INVASIVE ADENOCARCINOMA" OR "pulmonary minimally invasive adenocarcinomas" OR "pulmonary minimally invasive adenocarcinomaS" OR "Pulmonary minimally invasive adenocarcinomas" OR "Pulmonary minimally invasive adenocarcinomaS" OR "PULMONARY MINIMALLY INVASIVE ADENOCARCINOMAs" OR "PULMONARY MINIMALLY INVASIVE ADENOCARCINOMAS" OR "pulmonary scc" OR "pulmonary SCC" OR "Pulmonary scc" OR "PULMONARY SCC" OR "pulmonary sccs" OR "pulmonary sccS" OR "pulmonary SCCs" OR "pulmonary SCCS" OR "Pulmonary sccs" OR "Pulmonary sccS" OR "PULMONARY SCCs" OR "PULMONARY SCCS" OR "sarcomatoid carcinoma of the lung" OR "Sarcomatoid carcinoma of the lung" OR "SARCOMATOID CARCINOMA OF THE LUNG" OR "sarcomatoid carcinoma of the lungs" OR "sarcomatoid carcinoma of the lungS" OR "Sarcomatoid carcinoma of the lungs" OR "Sarcomatoid carcinoma of the lungS" OR "SARCOMATOID CARCINOMA OF THE LUNGs" OR "SARCOMATOID CARCINOMA OF THE LUNGS" OR "scc of the lung" OR "Scc of the lung" OR "SCC of the lung" OR "SCC OF THE LUNG" OR "scc of the lungs" OR "scc of the lungS" OR "Scc of the lungs" OR "Scc of the lungS" OR "SCC of the lungs" OR "SCC of the lungS" OR "SCC OF THE LUNGs" OR "SCC OF THE LUNGS" OR "solid predominant adenocarcinoma of the lung" OR "Solid predominant adenocarcinoma of the lung" OR "SOLID PREDOMINANT ADENOCARCINOMA OF THE LUNG" OR "solid predominant adenocarcinoma of the lungs" OR "solid predominant adenocarcinoma of the lungS" OR "Solid predominant adenocarcinoma of the lungs" OR "Solid predominant adenocarcinoma of the lungS" OR "SOLID PREDOMINANT ADENOCARCINOMA OF THE LUNGs" OR "SOLID PREDOMINANT ADENOCARCINOMA OF THE LUNGS" OR "squamous cell carcinoma of lungs" OR "Squamous cell carcinoma of lungs" OR "SQUAMOUS CELL CARCINOMA OF LUNGS" OR "squamous cell carcinoma of the lung" OR "Squamous cell carcinoma

of the lung" OR "SQUAMOUS CELL CARCINOMA OF THE LUNG" OR "squamous cell carcinoma of the lungs" OR "squamous cell carcinoma of the lungS" OR "Squamous cell carcinoma of the lungs" OR "Squamous cell carcinoma of the lungS" OR "SQUAMOUS CELL CARCINOMA OF THE LUNGs" OR "SQUAMOUS CELL CARCINOMA OF THE LUNGS" OR "squamous cell lung carcinoma" OR "Squamous cell lung carcinoma" OR "SQUAMOUS CELL LUNG CARCINOMA" OR "squamous cell lung carcinomas" OR "squamous cell lung carcinomaS" OR "Squamous cell lung carcinomas" OR "Squamous cell lung carcinomaS" OR "SQUAMOUS CELL LUNG CARCINOMAs" OR "SQUAMOUS CELL LUNG CARCINOMAS")) NOT "metastatic to the lung") NOT "lung metastasis"

Then, we looked for those sDEG (FDR<=5*10-6) AD+/GBM+/LC- and AD-/GBM-/LC+ located over the third-quartile of genes mentioned in the abstracts of the three diseases.
